# Proteolysis Targeting Chimera Degraders of the METTL3–14 m^6^A-RNA Methyltransferase

**DOI:** 10.1021/jacsau.4c00040

**Published:** 2024-02-12

**Authors:** Francesco Errani, Annalisa Invernizzi, Marcin Herok, Elena Bochenkova, Fiona Stamm, Ivan Corbeski, Valeria Romanucci, Giovanni Di Fabio, František Zálešák, Amedeo Caflisch

**Affiliations:** # Department of Biochemistry, University of Zurich, Winterthurerstrasse 190, Zurich CH-8057, Switzerland; † Università degli Studi di Napoli Federico II, Via Cintia 4, Napoli I-80126, Italia

**Keywords:** PROTACs, METTL3, METTL14, CRBN, degradation, m^6^A-RNA, AML, prostate cancer

## Abstract

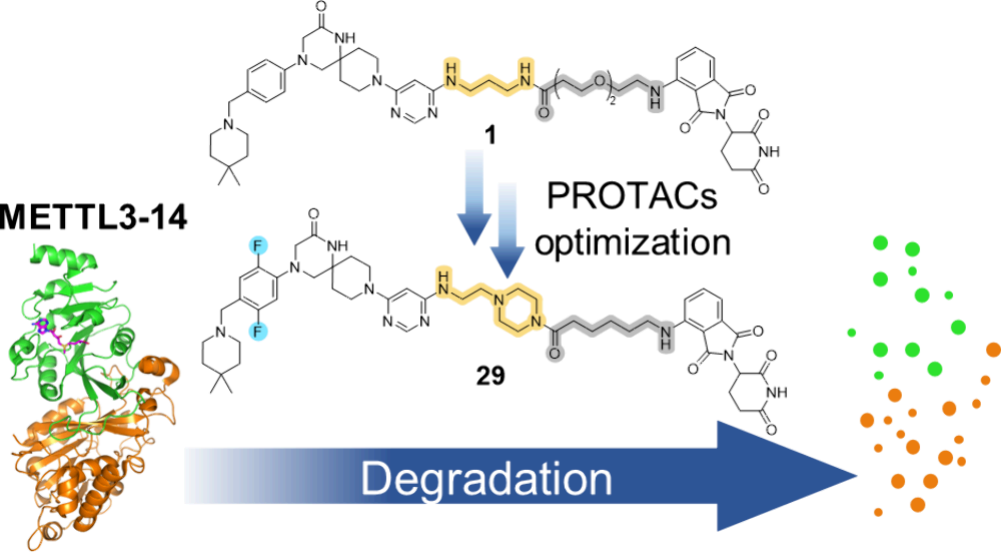

Methylation of adenine N6 (m^6^A) is the most frequent RNA modification. On mRNA, it is catalyzed by the METTL3–14 heterodimer complex, which plays a key role in acute myeloid leukemia (AML) and other types of blood cancers and solid tumors. Here, we disclose the first proteolysis targeting chimeras (PROTACs) for an epitranscriptomics protein. For designing the PROTACs, we made use of the crystal structure of the complex of METTL3–14 with a potent and selective small-molecule inhibitor (called UZH2). The optimization of the linker started from a desfluoro precursor of UZH2 whose synthesis is more efficient than that of UZH2. The first nine PROTAC molecules featured PEG- or alkyl-based linkers, but only the latter showed cell penetration. With this information in hand, we synthesized 26 PROTACs based on UZH2 and alkyl linkers of different lengths and rigidity. The formation of the ternary complex was validated by a FRET-based biochemical assay and an *in vitro* ubiquitination assay. The PROTACs **14**, **20**, **22**, **24**, and **30**, featuring different linker types and lengths, showed 50% or higher degradation of METTL3 and/or METTL14 measured by Western blot in MOLM-13 cells. They also showed substantial degradation on three other AML cell lines and prostate cancer cell line PC3.

## Introduction

Post-transcriptional (epitranscriptomic) modifications of RNA have a key role in gene expression and cell homeostasis regulation.^1,2^ The N6-adenosine methylation (m^6^A) is the most abundant among over 150 reported modifications.^3^ It has been found on mRNA, tRNA, rRNA, and several noncoding RNAs.^4^ The m^6^A is a dynamic and reversible modification deposited by proteins defined as “writers” and removed by “eraser” proteins. A third family of epitranscriptomic proteins (“readers”) recognize the methylated RNA, leading to splicing, nuclear export, translation, altered stability, and degradation of transcripts.^5−9^ In this way, the m^6^A modification can mediate the expression or silencing of specific genes.^2^

This epitranscriptomic machinery enables processes such as stem cell differentiation,^10^ cell response to stress,^11^ and regulation of the circadian cycle^12^ under physiological conditions. Its dysregulation has been linked to a growing number of pathological conditions. In particular, abnormal m^6^A levels have been connected to different kinds of cancer including leukemia, prostate cancer, breast cancer, liver cancer, colorectal cancer, and others.^13−20^

Methyltransferase-like 3 (METTL3) and METTL14 form the heterodimeric protein complex that catalyzes the deposition of the m^6^A modification (writer). METTL3 is the catalytic subunit that binds the cosubstrate *S*-adenosyl-l-methionine (SAM) while METTL14 facilitates RNA binding and stabilization of the complex.^21,22^ Many studies show that increased m^6^A levels can lead to enhanced cell proliferation, antiapoptotic effects, promotion of migration, and invasion.^23^ Moreover, METTL3 has been reported to promote other cancerogenic processes independently of its catalytic activity.^24^ Mouse knockout studies have revealed that the depletion of m^6^A modification leads to early embryonic lethality. Mouse embryonic stem cells (mESCs) could survive the METTL3 gene knockout and continue to proliferate but lost the ability to differentiate.^25^ In human hematopoietic stem/progenitor cells (HSPCs), m^6^A modification controls myeloid differentiation. Short hairpin RNA (shRNA)-mediated silencing of METTL3 in HSPCs promotes cell differentiation and reduces cell proliferation.^26^ These two examples demonstrate the relevance of m^6^A modification in normal cell differentiation processes, but its effects are largely dependent on the cellular context. Importantly, METTL3 mRNA and protein are expressed at higher levels in AML cells than in healthy HSPCs, which can result in a therapeutic window to target the protein with inhibitors and degraders.

To date, only three series of SAM-competitive, potent, and selective inhibitors of METTL3 have been reported, two of them originating from medicinal chemistry campaigns carried out in our group at the University of Zurich (UZH).^27−30^ The low nanomolar inhibitors UZH2 and the compound published by Storm Therapeutics (STM2457) have shown antiproliferative effects in acute myeloid leukemia (AML) cell lines, strengthening the therapeutic potential of targeting the METTL3–14 complex.^27,29^ A small molecule inhibitor of METTL3 called STC-15 (SAM-competitive, developed by Storm Therapeutics) is currently in phase 1 clinical trials (https://clinicaltrials.gov/study/NCT05584111). A total of 66 patients have been enrolled and dosed (since Nov. 15, 2022) to evaluate the safety, pharmacokinetic, pharmacodynamic, and clinical activity of STC-15 in subjects with advanced malignancies. Serious side effects after dosing STC-15 do not seem to have emerged as the clinical trials are ongoing for nearly 14 months. A recent in vivo study using the METTL3 inhibitor STM2457 (a predecessor of the compound STC-15 currently in clinical trials) reported milder, more nuanced, and manageable effects of pharmacological METTL3 inhibition on normal hematopoiesis than those observed in METTL3 knockout studies. The observed lineage bias in the earliest hematopoietic progenitors included an increase in neutrophils and a decrease in erythroids, indicating anemia as a potential side effect of catalytic METTL3 inhibition.^31^ Available data show that targeting METTL3 by knockout or inhibition affects normal cells, but the effect depends on the cellular and systemic context. However, the high cellular concentration of SAM (60 to 160 μM as measured in rat liver)^32^ can limit the scope of SAM-competitive inhibitors.

Proteolysis targeting chimeras (PROTACs) are a valid alternative to small-molecule inhibitors.^33−36^ PROTACs are heterobifunctional molecules bearing a protein of interest (POI) ligand covalently linked to an E3 ligase ligand. Upon binding to both targets, PROTACs promote ubiquitination of the POI and its subsequent degradation by the 26S-proteasome. This is a promising approach already applied to a variety of targets, in particular in the epigenetic field.^37^ Their catalytic-like mechanism of action results in the recycling/reuse of the PROTAC molecules upon protein degradation. Moreover, because of the degradation of the whole protein, PROTACs eliminate both its enzymatic and scaffolding functions, acting as a chemical knockout of the protein.

Here, we report a medicinal chemistry campaign aimed at the development of PROTAC molecules against METTL3–14, the human m^6^A-RNA writer complex.

## Results and Discussion

### Protein Structure-Based Design

**Scheme 1:**
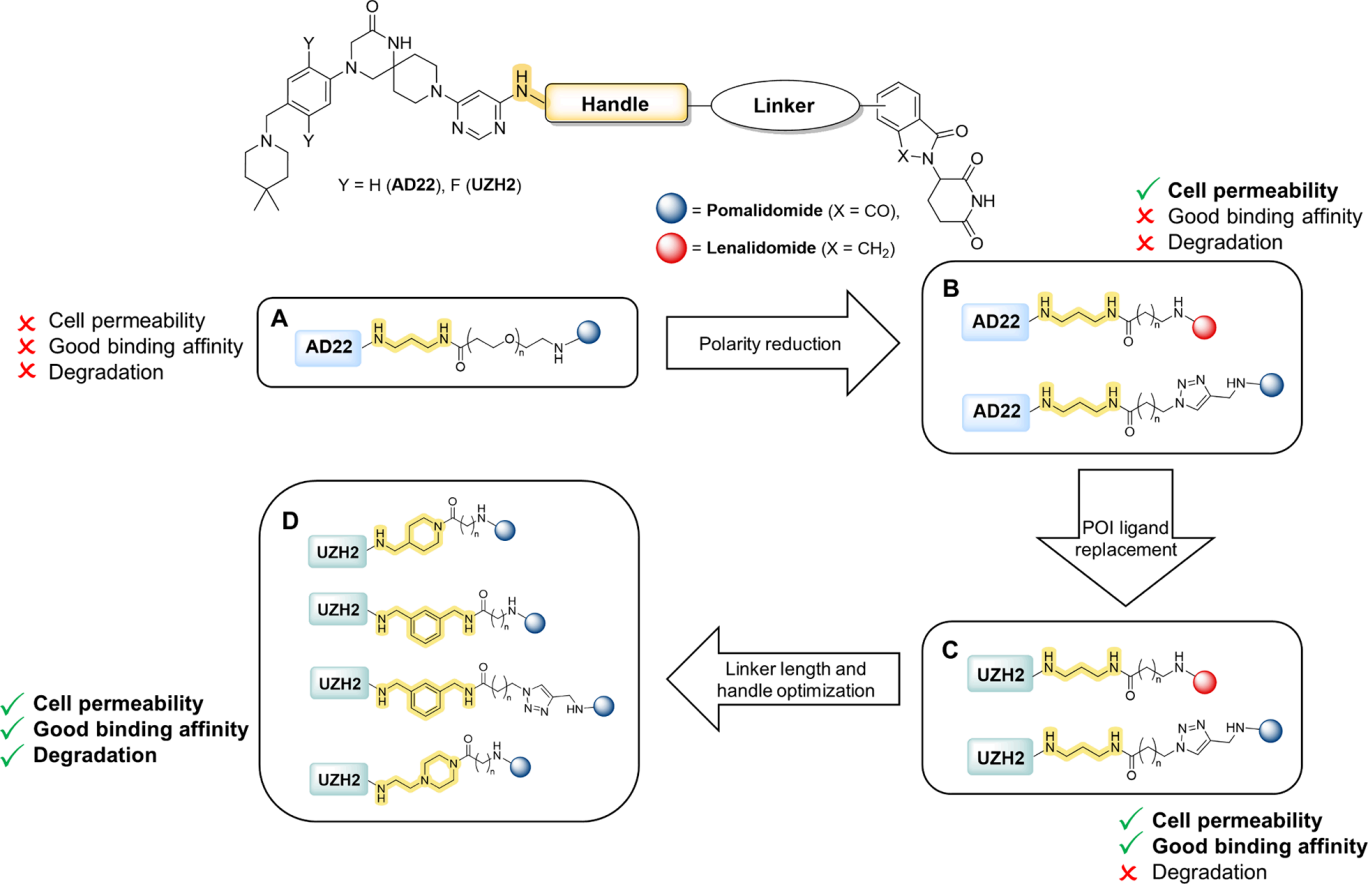
General Structure of PROTACs and Optimization Strategy

**Figure 1:**
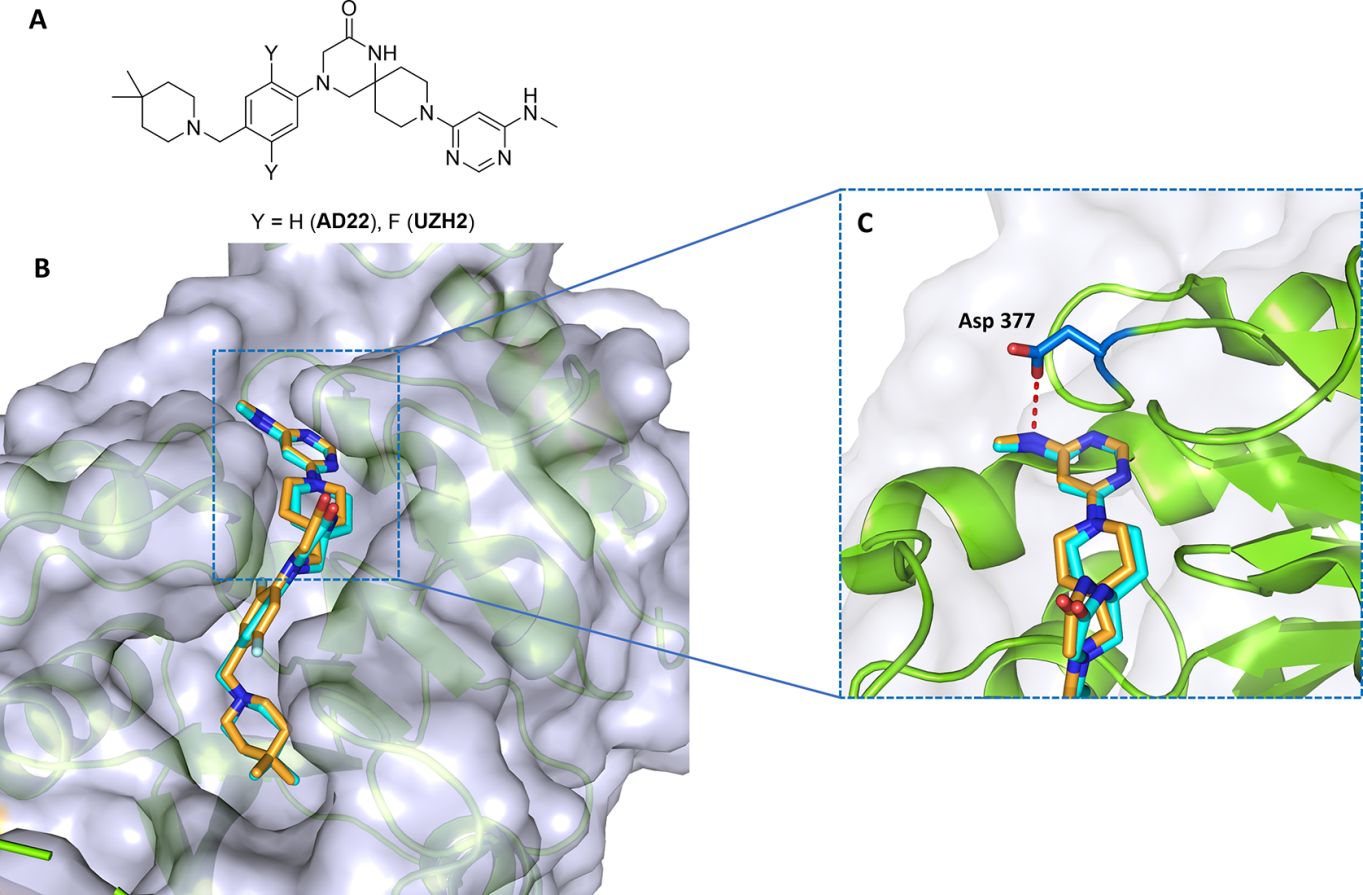
Protein structure-based design of PROTACs. (a) 2D structures of UZH2 and its desfluoro precursor AD22. (b) Overlap of the crystal structures of METTL3 bound to AD22 (carbon atoms in cyan, PDB 7O0P) and UZH2 (carbon atoms in orange, PDB 7O2F). (c) Zoom in on the hydrogen bond between the side chain of Asp377 and the methylamine of AD22 and UZH2.

The medicinal chemistry campaign builds upon our previous results obtained during the development of small molecule inhibitors for METTL3–14.^27^ Here, we start from two potent and selective METTL3–14 inhibitors, UZH2 (IC_50_ = 5 nM, selectivity data in Table S1) and its desfluoro derivative AD22 (IC_50_ = 89 nM, compound **10** in ref.^27^) (Figure 1A). As E3 ubiquitin ligase, we selected Cereblon (CRBN), for which the most common ligands are 4-amino thalidomide (pomalidomide) and lenalidomide.^38,39^ The general structure of the synthesized PROTACs is represented in Scheme 1. The UZH2/AD22 atom for the covalent bond with the linker was identified by crystallographic analysis. The binding poses of UZH2 (PDB 7O2F) and AD22 (PDB 7O0P) in the METTL3–14 complex (Figure 1B) provide an exit vector from the pyrimidine ring into the solvent-exposed area. Replacing the methylamino moiety with a propyl diamino motif (handle) allowed a convenient connection to the CRBN ligand via a linker. The amino group directly connected to the pyrimidine ring was intended to maintain a favorable hydrogen bond interaction with the side chain of Asp377 in METTL3 (Figure 1C). The terminal amino functionality allowed the final amide bond formation, thus connecting the POI ligand with pomalidomide through the linker (Scheme 1). The propyl diamino moiety is formally considered to be part of the linker. Nevertheless, it affects the affinity of the PROTACs for METTL3–14 as measured in our time-resolved FRET assay (hereafter referred to as binary assay).^40^ For this reason, this portion is called handle to clearly distinguish it from the rest of the linker. The structures of all the synthesized PROTACs (compounds **1**–**35**) are reported in Table 1. The first set of PROTAC molecules (compounds **1**–**4**) consists of AD22 as the POI ligand, propyl diamine handle, polyethylene glycol (PEG) linker, and pomalidomide as the E3 ligase binder (Scheme 1A). Their synthesis was achieved by a final amide bond formation, as described in further detail in Scheme 4 in the Synthesis section.

**Table 1:**
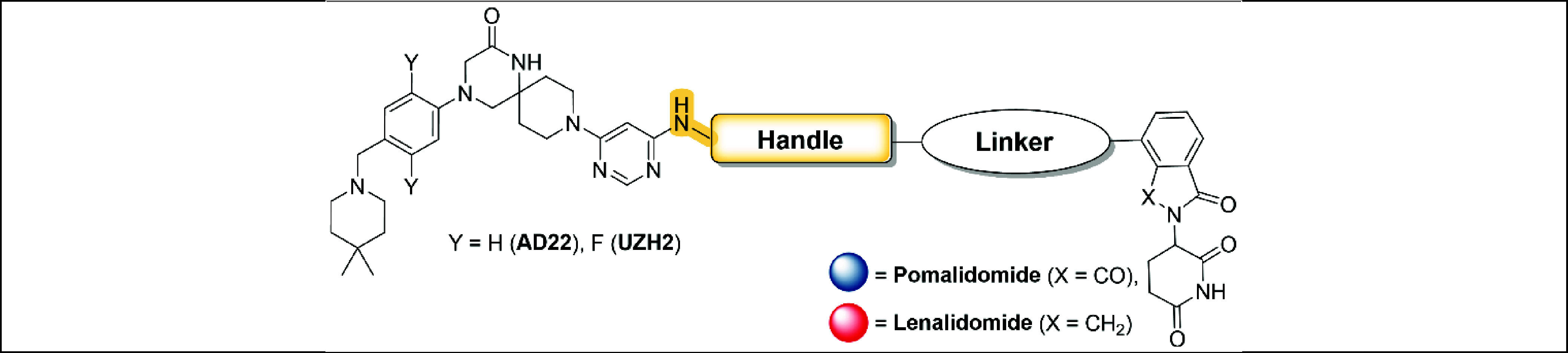

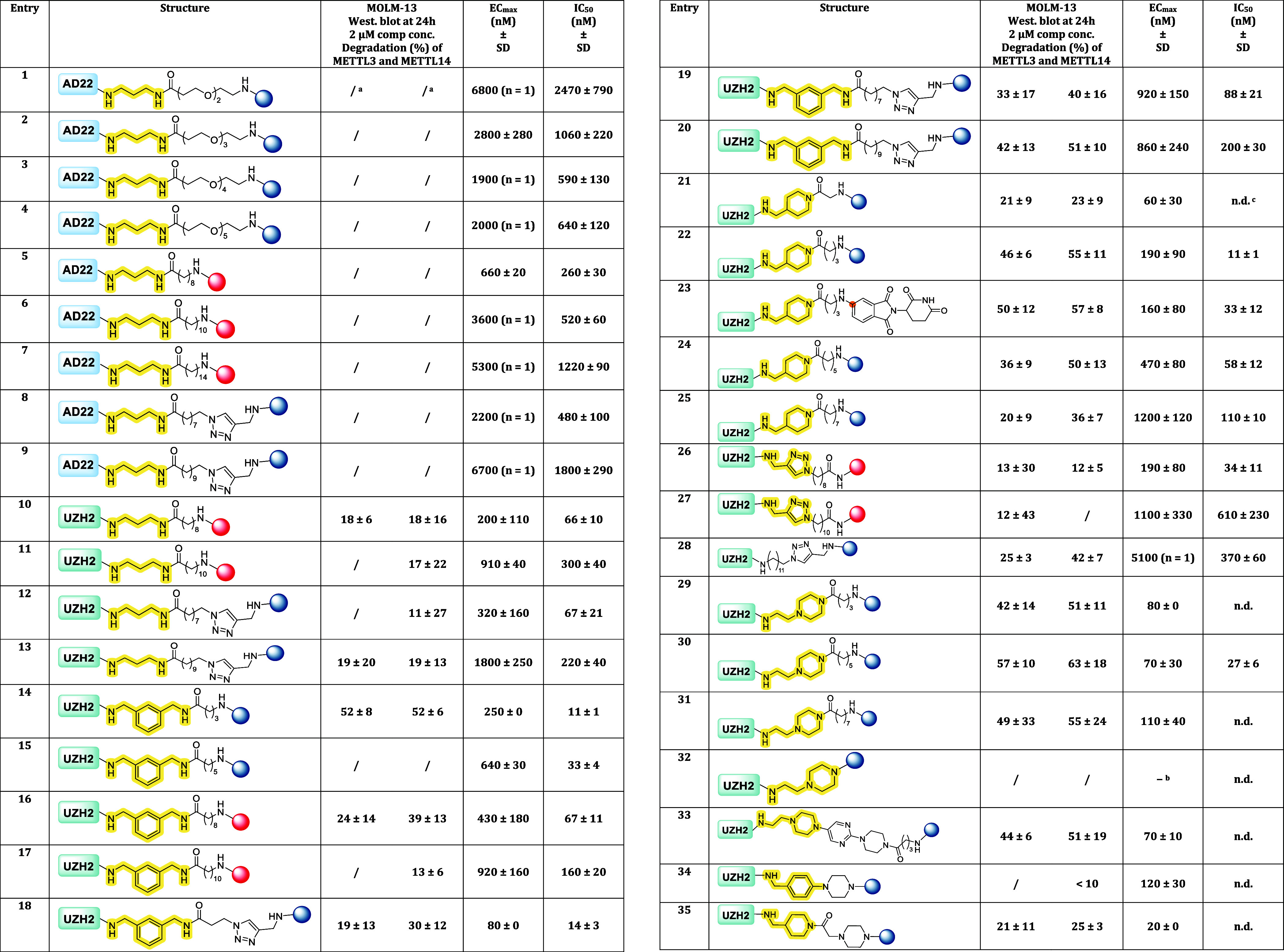
Synthesized PROTACs and Activity Data

The EC_max_ value is the PROTAC concentration at the maximum of the signal in the ternary complex formation assay (TCFA). The IC_50_ value is the PROTAC concentration required to inhibit 50% of the catalytic activity of METTL3-14, as measured by the binary FRET assay.

a Degradation <10% or stabilization.

b The compound was inactive in the TCFA.

c Not determined.

**Figure 2:**
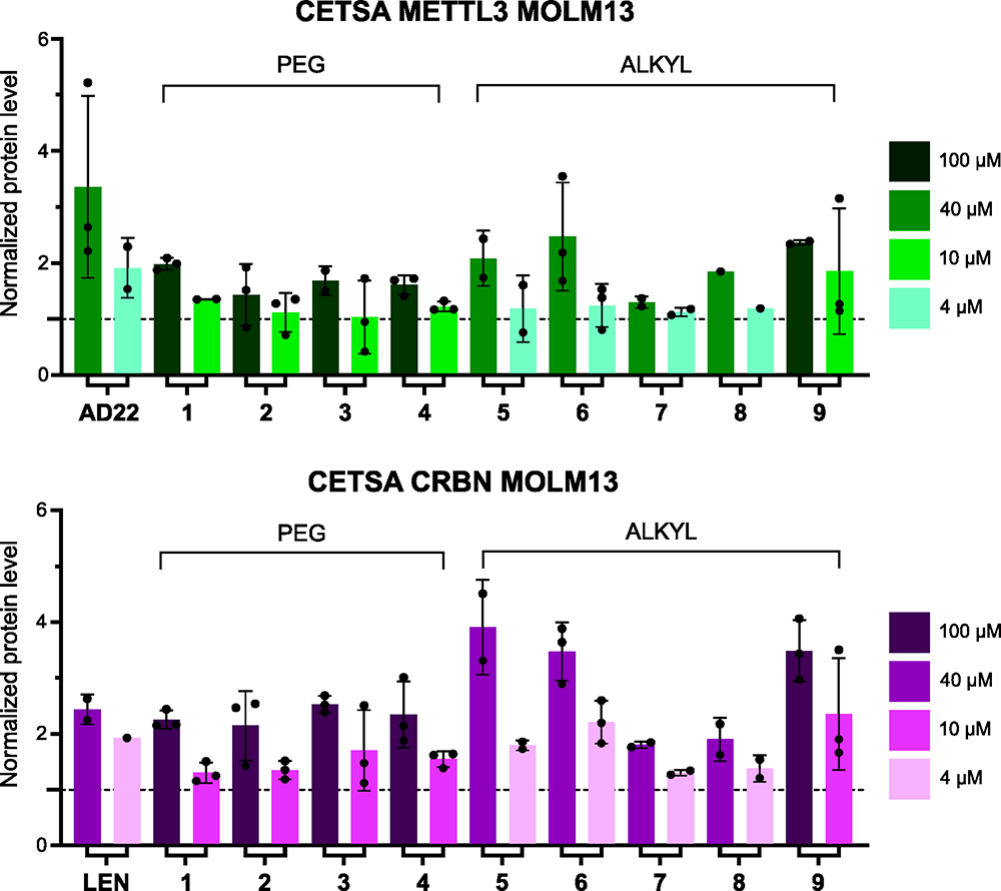
Evaluation of AD22-based PROTACS **1**-**9** in the MOLM-13 (AML) cell line. The stabilization of METTL3 (top) and Cereblon (CRBN, bottom) was quantified by CETSA at 54 °C. The SAM-competitive inhibitor AD22 was employed as a control for METTL3 (top, left), while lenalidomide (LEN) was used as a control for Cereblon (bottom, left). The dashed line represents the protein level of the DMSO control used for normalization (*y* = 1).

The rationale behind the use of PEG linkers in the first generation was the commercial availability of PEG chains with different numbers of PEG subunits.^41^ This allowed us to cover different distances between the PROTAC moieties for POI and CRBN. This is useful for investigating the optimal range for the formation of the ternary complex CRBN/PROTAC/METTL3–14. Moreover, the PEG chain is widely used in cross-linking for bioconjugation and biolabeling, due to its favorable physicochemical properties.^42−44^ The degradation of METTL3 and METTL14 was measured individually by Western blot at various PROTAC concentrations (10, 5, 1, 0.1, 0.01 μM) at the 16 h time point in MOLM-13, which is an AML cell line. However, none of the four first-generation PROTACs (compounds **1**–**4**) showed degradation activity (Table 1). Compounds **1**, **2**, **3**, and **4** were also tested in a biochemical ternary complex formation assay (TCFA).^45^ Relatively high effective concentrations at the peak of the Hook curve (E*C*_max_: 6.8, 2.8, 1.9, and 2.0 μM) reflect low affinity toward both CRBN and METTL3–14 (Table 1). In addition, the amplitude of the Hook curve (Table S2) is low compared to those of other compounds in this paper. This can be an indication of weak cooperativity and less stable ternary complex formation, which is needed for successful degradation activity.^46^ As PROTACs can suffer from low cellular permeability due to their high molecular weight and/or high hydrophilicity,^47^ we decided to assess permeability and target engagement in cells using the cellular thermal shift assay (CETSA).^48^ The first set of PROTACs (compounds **1**–**4**) exhibited low protein stabilization and only at the very high concentration of 100 μM. Considering the CETSA (Figure 2) and TCFA results, the lack of degradation might be a result of low cell permeability and/or inability to form a stable ternary complex.

### Optimization of Cell Permeability and POI Affinity

To address the low permeability, we synthesized a second set of AD22-based PROTACs (compounds **5**–**9**), aiming to increase the lipophilicity of the molecules. The PEG linker was replaced by an alkyl chain (Scheme 1B).^49−51^ The positive effect of the increased lipophilicity on cell membrane permeation was confirmed through CETSA experiments, as indicated by more pronounced stabilization effects (Figure 2). To improve the stability of the ternary complex, we decided to change the POI ligand by replacing AD22 with the ∼20-fold more potent inhibitor UZH2 (Scheme 1C). As expected, the resulting PROTACs (compounds **10**–**13**) showed a much higher affinity for METTL3–14 in comparison to their AD22 analogues (Table 1). Furthermore, the EC_max_ measured in TCFA was substantially improved. Compounds **10**, **11**, and **12** showed an EC_max_ below 1 μM, with a 3- to 6-fold improvement compared to the nonfluorinated analogues (Table 1). Despite the increased cellular permeability and binding affinity to the target protein, none of the tested compounds showed degradation of METTL3–14, as measured by Western blot at 0.2, 2, and 20 μM PROTAC concentration at multiple time points (6, 16, 36 h). As the PROTACs synthesized so far featured the same handle motif (propyl diamine), we questioned its impact on ternary complex formation and protein degradation. From previous studies, we knew that the replacement of the methyl amino group in UZH2 with an aryl or aliphatic ring can provide favorable lipophilic interaction with the edge of the METTL3–14 binding site.^30^ Moreover, a rigid and bulky feature in the handle or the linker could lead to a PROTAC conformation more prone to cell permeation and/or ternary complex formation.^41,52−54^ With this in mind, we moved on to the synthesis of PROTACs bearing a more rigid handle/linker (Scheme 1D).

### Optimization of Length and Rigidity of the Linker

**Figure 3:**
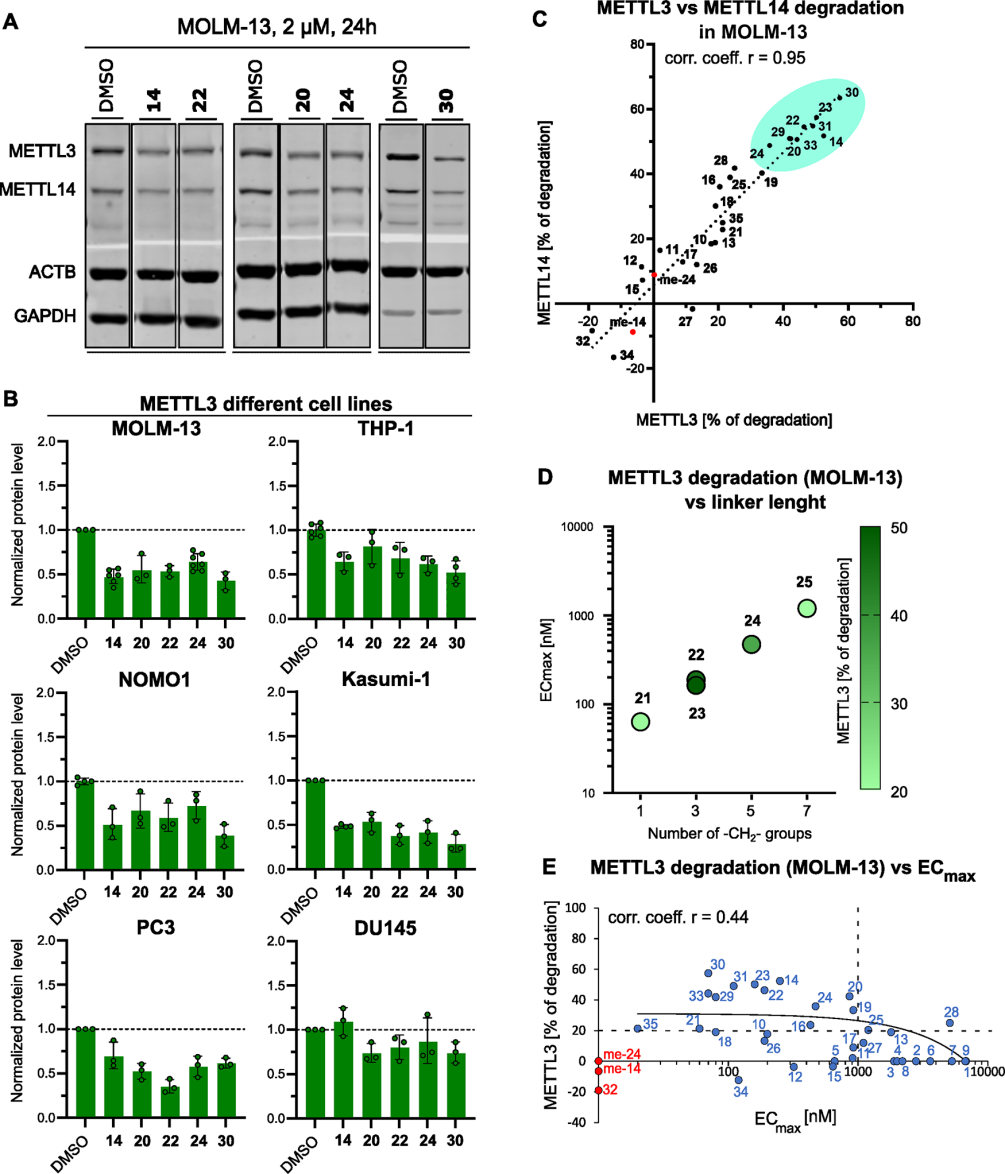
(a) Representative Western blots from a cellular degradation assay with PROTACs **14**, **20**, **22**, **24**, and **30** in the MOLM-13 cell line. Full membranes are shown in Figure S2. (b) METTL3 Western blot quantification by densitometry from a cellular degradation assay at PROTAC concentration of 2 μMin the AML cell lines MOLM-13, THP-1, NOMO-1, and KASUMI-1 and in the prostate cancer cell lines PC3 and DU145 (representative Western blots are shown in Figure S3). The dashed line represents the protein level of the DMSO control, used for normalization (*y* = 1). (c) Correlation of the degradation of METTL3 and METTL14 in MOLM-13 at PROTAC concentration of 2 μM; the dotted black line is a linear regression of all the compounds (corr. coeff. *r* = 0.95). The nine most active PROTACs are highlighted in turquoise. **me-14** and **me-24** (in red) are the methylated negative controls of PROTACs **14** and **24** (for structures, see Figure 4B). (d) Correlation between the number of −CH_2_– groups in the linker and the EC_max_ value measured in the TCFA. The color of the data points reflects the degradation of METTL3 as measured by Western blot in MOLM-13 (legend on the right). While the EC_max_ improves until the shortest length of the linker (one −CH_2_−), the highest degradation is observed at intermediate lengths, i.e., (−CH_2_−)_3_. (e) Scatter plot of the degradation of METTL3 in MOLM-13 as a function of the EC_max_ values measured in the TCFA. The data points on the *y*-axis (red) did not show any detectable ternary complex formation in the TCFA. The linear regression for the compounds with both degradation and EC_max_ values (blue) is shown (black continuous line). The horizonal dashed line marks a METTL3 degradation level of 20%, and the vertical dashed line marks EC_max_ = 1 μM.

The next set of seven PROTAC molecules contained a benzyl diamine handle instead of a propyl diamine. In addition, we varied the length of the linear portion of the linker, retaining the alkyl (compounds **14**–**17**) and alkyl-triazole (compounds **18**–**20**) motifs from previous optimization steps. The presence of the aromatic ring significantly improved the affinity for METTL3–14 (measured by the FRET-based binary assay) as well as the values of EC_max_ (Table 1). Substantial degradation of both METTL3 and METTL14 proteins after 24 h incubation was observed with 2 μM PROTAC concentration in MOLM-13 cells. It is important to note that for SAM-competitive PROTACs the cellular activity at low micromolar concentration is in line with the low micromolar activity measured previously for UZH2 in cellular assays^25,29^ which is due to the aforementioned high concentration of SAM. Compounds **14**, **19**, and **20** reduced the level of both METTL3 (by 52, 33, and 42%, respectively) and METTL14 (by 52, 40, and 51%, respectively). The most promising derivative, PROTAC **14**, contained a shorter linker in comparison with the other PROTACs of this set. Furthermore, we synthesized another set of 11 PROTACs that contained lipophilic and rigid handles such as piperidine (compounds **21**–**25**), piperazine (compounds **29**–**32**), and triazole (compounds **26** and **27**) in combination with different alkyl linker lengths. After a round of protein degradation screening in cells and quantification by Western blot analysis (2 μM, 24 h MOLM-13) compounds **22**, **23**, **24**, **29**, **30**, and **31**, featuring piperidine or piperazine handle, showed a 50% or higher degradation of METTL3 and/or METTL14 (Table 1, Figure 3A,B).^55,56^ The correlated degradation of the two proteins of the heterodimeric complex METTL3–14 provides evidence that a PROTAC binding at the SAM-pocket of METTL3 can degrade both proteins (Figure 3C, Figure S1). PROTAC **30** displayed the most significant degradation activity, achieving a reduction of both METTL3 and METTL14 by about 60%. In contrast, the PROTACs with a triazole ring as a handle (compounds **26** and **27**) performed worse, showing degradation efficacies of 13 and 12%, respectively.

The highest degradation (50–60%) was achieved with compounds featuring a linker length spanning between three and five methylene groups, i.e., PROTACs **14**, **22**, **23**, and **30**. The TCFA shows gradual EC_max_ improvement when reducing the linker length, as we can see for the piperidine handle series PROTACs **25**, **24**, **23**, **22**, and **21** (Figure 3D). Among them, the shortest PROTAC, compound **21**, has the best EC_max_ (0.06 μM), but in terms of degradation, it is worse than its slightly longer analogues **22** and **24**, with 21, 46, and 36% METTL3 reduction, respectively. A possible explanation for the discrepancy between the biochemical TCFA and the cellular degradation assay is that the TCFA employs truncated protein constructs. PROTAC **21** might be too short to form a stable ternary complex with full-length proteins, and some steric clashes can cause a nonoptimal conformation of the ternary complex. This EC_max_ improvement tendency is not observed at even shorter linker lengths, as shown with PROTAC **32** (no linker), which is inactive in both TCFA and cell degradation assays. Thus, there seems to be an optimal range for the linker length (three to five methylene groups).

Figure 3E shows the distribution of the percentage of protein degradation in MOLM-13 as a function of the EC_max_ measured in TCFA (Table 1). Even though the correlation is not strong (correlation coefficient *r* = 0.44), only PROTAC **28** shows degradation higher than 20% and EC_max_ > 1 μM. The biochemical TCFA can be considered a useful screening technique for prioritizing the PROTACs for biological characterization.

To further increase the rigidity of the handle/linker part, we synthesized three PROTACs (compounds **33**–**35**) with reduced linker flexibility. Among them, PROTAC **33** turned out to be the best, showing 44% METTL3 reduction, while compound **34** displayed a not significant protein reduction and **35** only 21% METTL3 reduction. Considering that compounds **34** and **35** contain a much shorter linker than that of PROTAC **33**, these results further highlight the importance of linker length.

Once the most promising handles and ideal length to achieve protein degradation were defined, we tried to modify the connection to the CRBN ligand.^57,58^ PROTAC **23** is linked to thalidomide at position 5. This modification is well tolerated, and the compound causes 50% degradation of METTL3. Nonetheless, compared to its 4-substituted analogue (PROTAC **22**), the difference in both degradation (50% vs 46%) and EC_max_ (0.16 μM vs 0.19 μM) is negligible (Table 1, Figure 3D).

At this point, we decided to select a subset of PROTACs for further validation in multiple AML cell lines. We first focused on the eight PROTACs that showed submicromolar EC_max_ and at least 50% degradation of METTL3 and/or METTL14 in MOLM-13 (**14**, **20**, **22**, **23**, **24**, **29**, **30**, **31**, highlighted in Figure 3C). Note that PROTAC **33** (also highlighted in Figure 3C) had not yet been prepared when we decided to focus on a small subset of PROTACs. We further restricted the selection to only five PROTACs as PROTAC **23** is the meta-substituted equivalent of **22**, and PROTACs **29**–**31** differ only in the number of methylene groups. Thus, the degradation activity of compounds **14**, **20**, **22**, **24**, and **30** was investigated in different AML cell lines (THP-1, NOMO-1, and KASUMI-1) (Figure 3B, Figures S1 and S3). While the degradation levels observed in THP-1 and NOMO-1 cell lines were comparable to those in MOLM-13, a higher degradation of both METTL3 and METTL14 was measured in KASUMI-1. The treatment of KASUMI-1 cells with 2 μM of PROTAC **30** for 24 h caused a 70% degradation of the POI.

To further validate the top PROTACs, we tested some of them against cell lines of solid tumors. We decided to focus on two prostate cancer cell lines (DU145 and PC3) because of recent evidence for the importance of METTL3 in prostate cancer.^59,60^ The selected PROTACs **14**, **20**, **22**, **24**, and **30** showed only a minor effect in the DU145 cell line (Figure 3B, Figure S3). In contrast, the METTL3 levels were reduced substantially in PC3 after 24 h, with PROTACs **20** and **22** showing the highest degradation values, 48 and 64% reduction of METTL3, respectively (Figure 3B, Figure S3). This result indicates that the degradation activity of our PROTAC molecules is not limited to leukemia cell lines, but they have a good potential also against prostate cancer. Moreover, the correlated degradation of METTL3 and METTL14 was observed not only in MOLM-13 (Figure 3C) but also in all of the other cell lines tested (Figure 3B, Figure S1).

### Validation of PROTACs Cellular Activity

**Figure 4:**
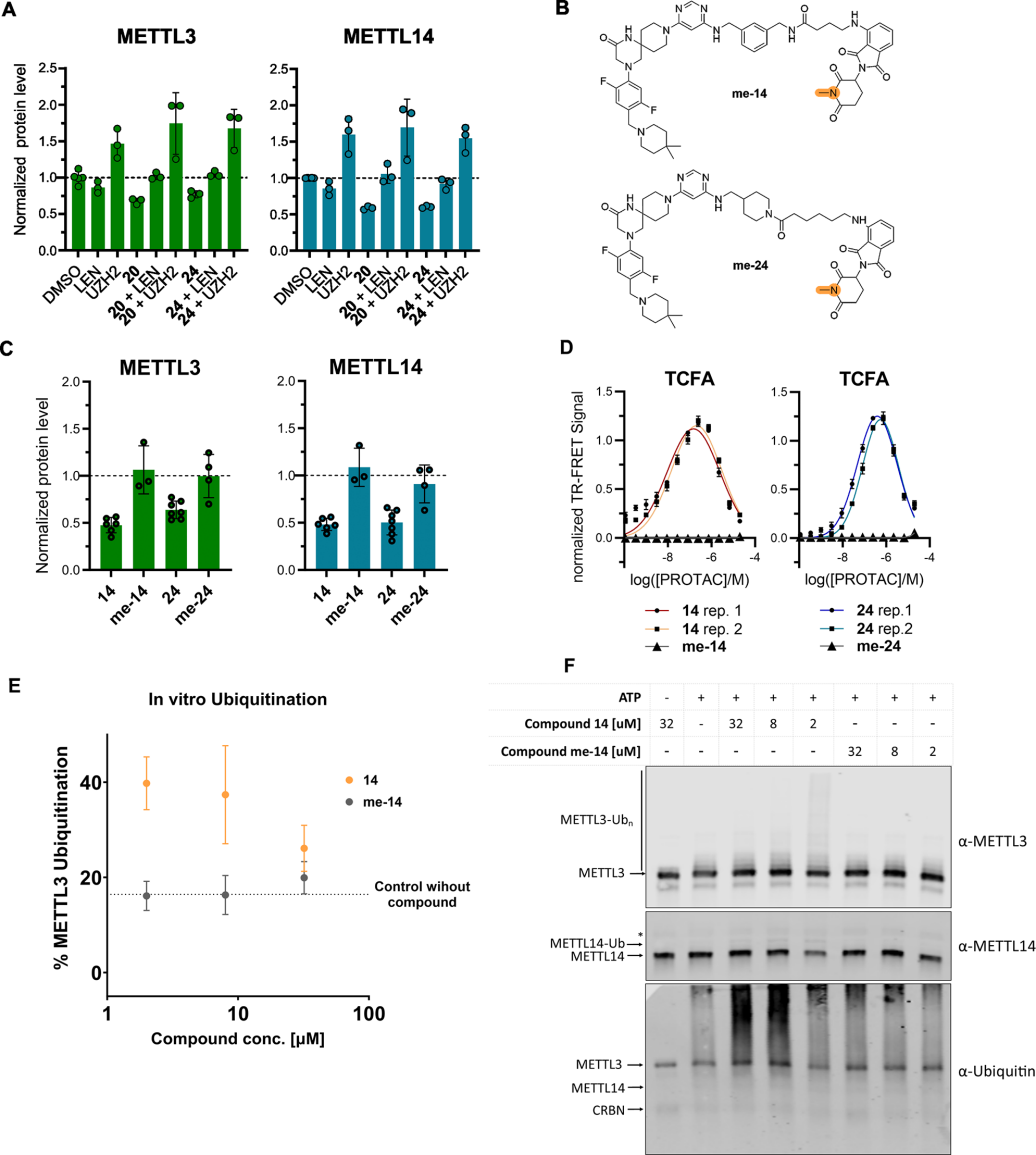
Control experiments, TCFA and in vitro ubiquitination. (a) METTL3 and METTL14 levels in the presence of lenalidomide or UZH2 in MOLM-13. The dashed line represents the protein level of the DMSO control, used for normalization (*y* = 1). (b) Chemical structures of PROTAC negative controls **me-14** and **me-24**. (c) METTL3 and METTL14 degradation by PROTACs **14** and **24** in comparison with their negative controls **me-14** and **me-24** in MOLM-13. The dashed line represents the protein level of the DMSO control, used for normalization (*y* = 1). (d) Biochemical FRET-based ternary complex formation assay (TCFA) with PROTACs **14** and **24** and their methylated negative controls **me-14** and **me-24**. (e) In vitro ubiquitination assay results with compound **14** and its negative control **me-14**. All data originate from biological and biochemical duplicates or more. (f) Western blot analysis of the *in vitro* ubiquitination assay. The ubiquitination reaction mixture (E1, E2, CUL4A-RBX1, CRBN-DDB1, and METTL3-METTL14 in reaction buffer) was incubated with or without ATP and at different concentrations of compounds **14** and **me-14** as indicated at 30 °C for 2 h. The proteins were separated by SDS-PAGE followed by Western blot analysis with α-METTL3, α-METTL14, and α-Ubiquitin antibodies. Shown here is one representative Western blot of three biological replicates of the experiment. For panel (e), densitometry was performed using the α-METTL3 blots of all three replicates. In the α-METTL14 blot, a weak band appears above the METTL14 band at 32, 8, and 2 μM of compound **14**, presumably indicating monoubiquitination of METTL14. However, the ubiquitination of METTL3 is clearly more efficient. The band indicated with an asterisk (*) originates from the unspecific detection of METTL3 with the α-METTL14 antibody.

At this stage, we evaluated the METTL3–14 protein levels after PROTAC treatment in combination with high concentrations of the small-molecule inhibitor lenalidomide or UZH2 (Figure 4A). These controls consist of saturating the binding pockets of CRBN or METTL3–14, respectively, thus preventing the formation of the ternary complex. Cells were treated with PROTACs **20** and **24** under three different conditions: 2 μM PROTAC, 2 μM PROTAC + 10 μM lenalidomide, and 2 μM PROTAC + 10 μM UZH2. As expected, we did not observe any degradation when applying a high concentration of lenalidomide. Interestingly, the presence of a high UZH2 concentration led to elevated levels of METTL3–14 both in the control as well as in combination with the PROTAC. This observation indicates a possible cellular compensatory mechanism aimed at preserving the protein catalytic activity in the presence of an inhibitor.^61^ It also offers a potential explanation for the difficulties in reaching degradation levels of METTL3–14 above 50%.

To confirm that our compounds lead to protein degradation by hijacking the Ubiquitin–proteasome system (UPS), we synthesized negative controls based on a single methylation, which results in inactive lenalidomide/pomalidomide derivatives.^62−64^ Thus, we prepared the methylated versions of PROTACs **14** and **24** (**me-14** and **me-24**) (Figure 4B). These methylated compounds did not show any METTL3–14 degradation at standard testing conditions (2 μM, 24 h) (Figure 4C).

Both control PROTACs gave no signal in the biochemical ternary complex formation assay (Figure 4D). The methylated derivatives are inactive, indicating their inability to form a ternary complex and further confirming the specificity of our PROTACs. Compound **me-14** was also tested in the CETSA on both METTL3 and CRBN to provide evidence that it is cell permeable and engages the POI and not CRBN (i.e., validation of the negative control). As expected, the compound showed stabilization of METTL3 but not of CRBN (Figure S4).

Furthermore, we set up an *in vitro* ubiquitination assay to quantify the ubiquitination of METTL3 and METTL14 in the presence of different concentrations of PROTACs. Compounds **14** and **me-14** were tested at 2, 8, and 32 μM (Figure 4E). While **me-14** does not increase the ubiquitination of METTL3 compared to the control without compound, PROTAC **14** raises the ubiquitination level of METTL3 to ∼40% at 2 μM. The decrease of ubiquitination at higher concentrations of compound **14** is consistent with the Hook effect. The ubiquitination of METTL14 is not as pronounced (Figure 4F). This can indicate that the ubiquitination site(s) is (are) mainly on METTL3. Interestingly, Zeng et al. showed that METTL14 can get ubiquitinated by STUB1 in the METTL3–14 interface and METTL3 therefore seems to prevent METTL14 ubiquitination.^65^ The simultaneous degradation of METTL3 and METTL14 caused by the PROTACs (Figure 3C) could be explained either by both proteins being subjected to the proteasome as a complex or by the reduced stability of METTL14 without METTL3.

**Figure 5:**
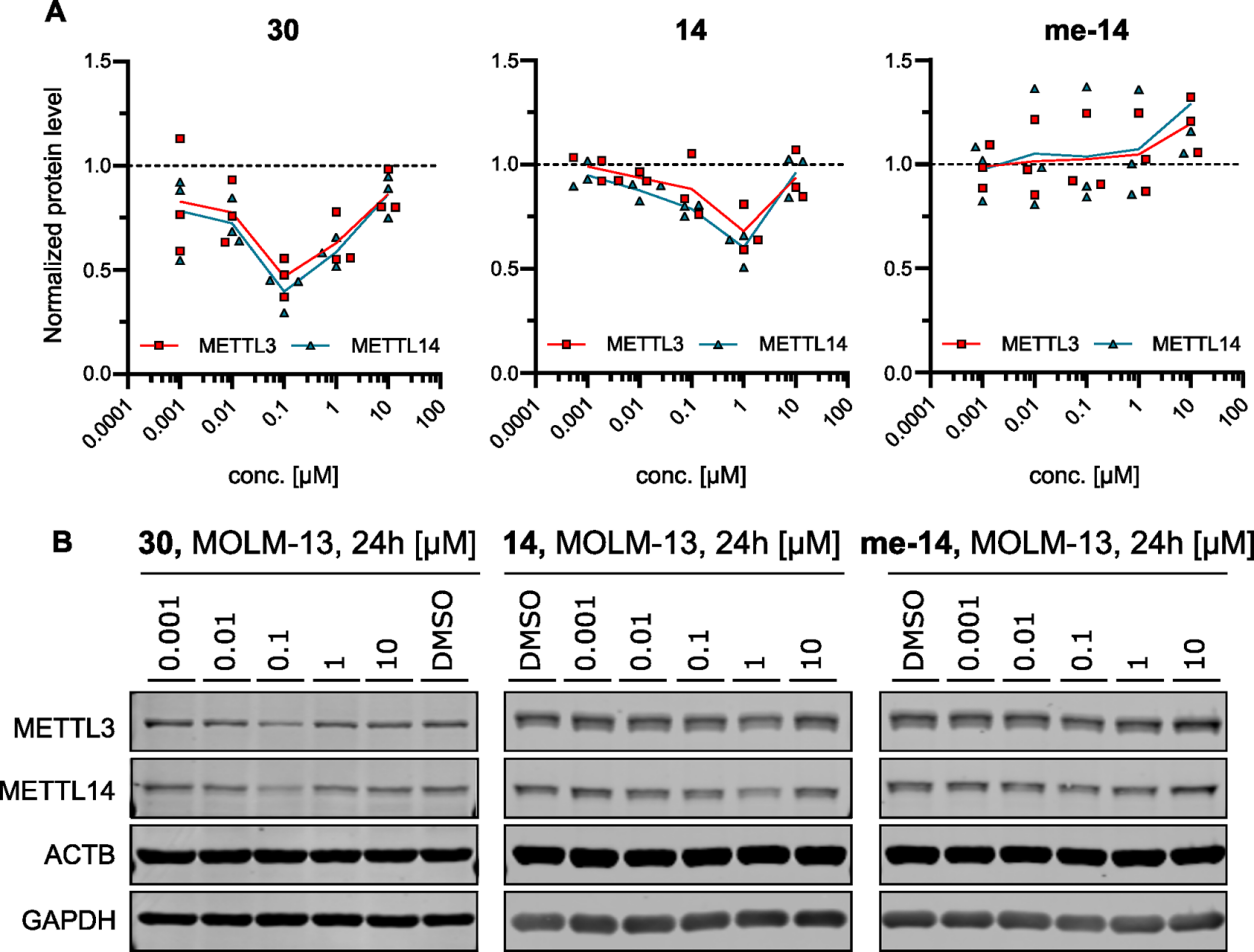
Cellular characterization. (a) Concentration dependence of METTL3 (red) and METTL14 (blue) degradation by PROTACs **30** and **14** and the methylated negative control of 14 (**me-14**) in MOLM-13 cells. The dashed line represents the protein level of the DMSO control, used for normalization (*y* = 1). (b) Representative Western blots. Full membranes are shown in Figure S5.

Lastly, we analyzed the concentration dependence of degradation of METTL3 and METTL14 in the MOLM-13 cell line. We selected PROTACs **14** and **30**, which have the highest METTL3 degradation in MOLM-13 (52 and 57%, respectively). The activity of PROTAC **14** was also compared to the one of its negative control **me-14.** We treated MOLM-13 cells with PROTACs **14**, **30**, and **me-14** at five different concentrations (Figure 5). As expected, the concentration dependence shows the so-called Hook effect,^66,67^ resulting from a saturation of CRBN and METTL3–14 at high PROTAC concentrations, where the PROTAC/CRBN and PROTAC/METTL3–14 binary complexes prevent the ternary complex formation and thus degradation. This result confirms that our compounds behave according to the principle of the PROTAC mechanism of action. For PROTAC **30**, the highest degradation was observed at 0.1 μM concentration, reaching up to 60% degradation, and for PROTAC **14**, the highest effect was seen at 1 μM, with a METTL3 reduction of around 50%. Compound **me-14** showed no degradation and even increased METTL3 levels at 10 μM, similar to that of the UZH2 inhibitor alone.

Overall, among the 35 PROTAC compounds synthesized in this study, nine (**14**, **20**, **22**, **23**, **24**, **29**, **30**, **31**, and **33**) caused at least 50% degradation of METTL3 and/or METTL14 in MOLM-13. Five of them (**14**, **20**, **22**, **24**, and **30**) were tested in different cell lines (AML and prostate cancer) and showed significant activity with METTL3–14 degradation up to 70% of the endogenous level (Figure 3B). The concentration-dependent degradation activity (Hook effect, Figure 5A), correlation in the degradation of the two proteins of the METTL3–14 heterodimeric complex (Figure 3C), methylated PROTAC controls (Figure 4), and the additional validation experiments with competitive small-molecule ligands (Figure 4A) provide strong evidence of cellular target engagement and selectivity of our PROTACs.

**Figure 6:**
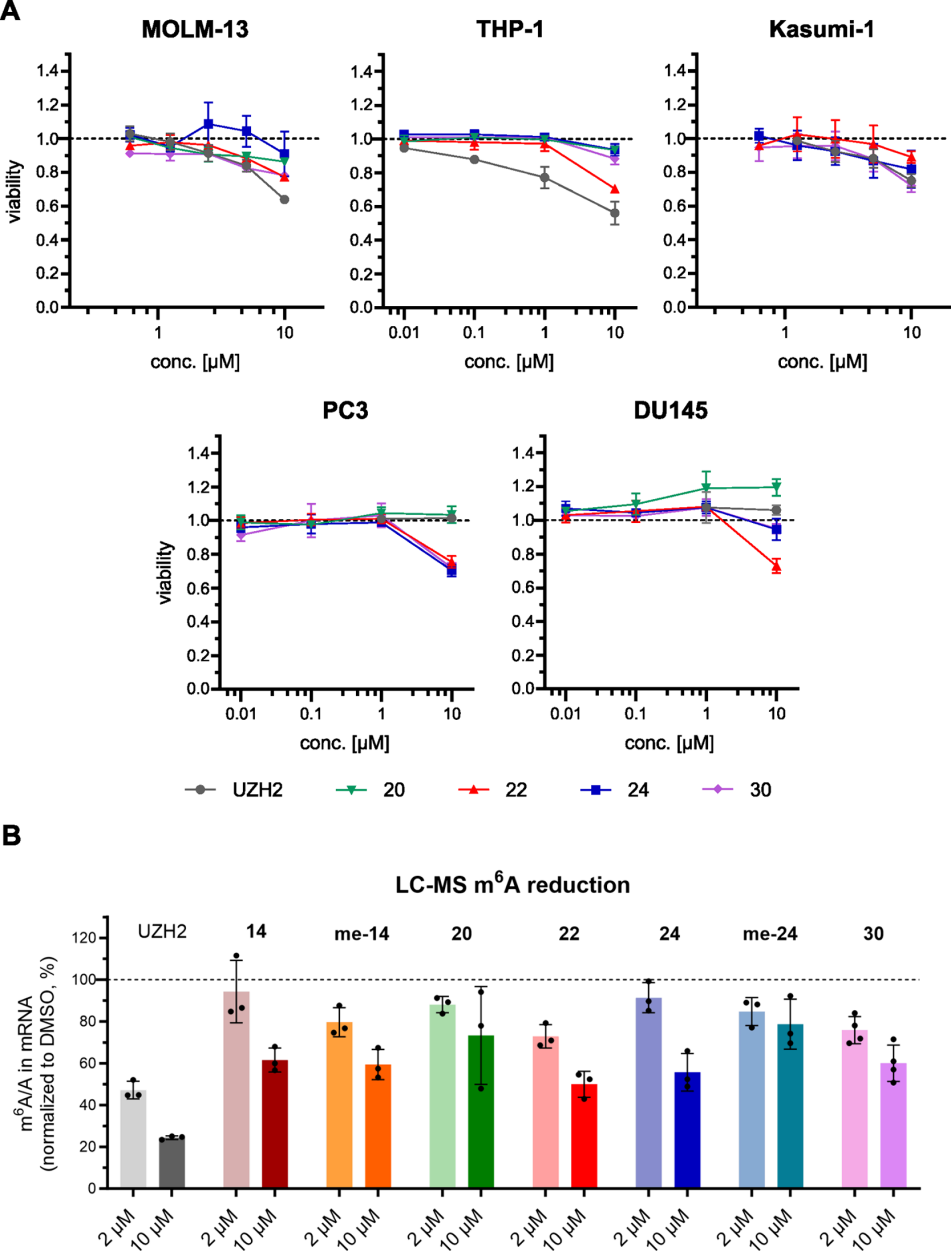
Cellular characterization. (a) Cell viability assay for PROTACs **20**, **22**, **24**,and **30** and the METTL3 catalytic inhibitor **UZH2** in AML cell lines (top) and prostate cancer cell lines (bottom). The dashed line represents the protein level of the DMSO control, used for normalization (*y* = 1). (b) LC-MS quantification of m^6^A/A levels in polyadenylated RNA in MOLM-13. The dashed line represents the protein level of the DMSO control, used for normalization (*y* = 1).

To assess whether degradation induced by our compounds translated into enhanced cell death, we performed cell viability assays on MOLM-13 with all of the UZH2-based PROTACs (Table S3). Compounds **20**, **22**, **24**, and **30** were also tested in the other AML cell lines THP-1 and Kasumi-1 and in the prostate cancer cell lines PC3 and DU145 (Figure 6A). Significant effects on the cell viability of some PROTACs were observed only at the highest concentration tested (10 μM). This concentration is higher than the EC_max_ values measured in the biochemical assay because, as mentioned before, the PROTACs (and UZH2) compete with the micromolar concentration of SAM in the cellular assays. Interestingly, on PC3 cell line only PROTACs (compounds **22**, **24**, and **30**) and not UZH2 showed an antiproliferative effect.

To better understand cell viability results in the MOLM-13 cell line, we measured the changes in cellular m^6^A/A levels (by LC-MS quantification) after PROTAC treatment (Figure 6B). At 2 μM (concentration used for degradation screening), we did not observe significant effects on the levels of m^6^A/A (except for a slight reduction for compound **22**). Measurable reduction of m^6^A/A was observed at a 10 μM concentration. Taking into consideration the concentration-dependent activity of PROTAC molecules (Hook effect), it is more likely that the observed reduction in m^6^A modification is due to inhibition of the catalytic activity of METTL3 by the UZH2-based warhead rather than protein degradation. Partial inhibition of the catalytic activity might also explain the observed cytotoxic effect at the highest tested concentrations of PROTACs. In conclusion, the modest cytotoxicity and reduction of m^6^A/A suggest that degradation levels higher than 50–70% are required to observe phenotypic effects specific to the PROTAC-induced METTL3–14 degradation.

### Synthesis

**Scheme 2:**
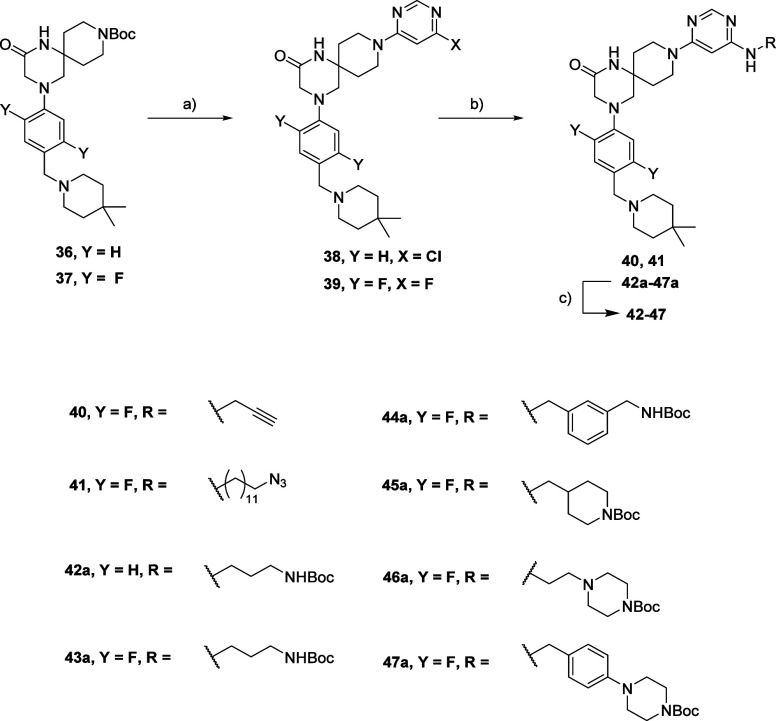
Synthesis Route for Compounds **40**–**47** Reagents and conditions: (a) (i) HCl aq. 37%, MeOH; (ii) for **38** and **39**: 4,6-dichloro pyrimidine (**38**)/4,6-difluoro pyrimidine (**39**), TEA, iPrOH; (b) RNH_2_, TEA, DMSO (**40**, **41**)/EtOH (**42a**-**47a**); (c) for **42**–**47**: TFA, DCM.

Starting from spiro compounds **36** and **37**, the preparation of POI ligands bearing the handle moiety was conducted following the general strategy reported in Scheme 2:

After Boc deprotection followed by an S_N_Ar reaction with 4,6-dichloro or difluoro pyrimidine, compounds **38** and **39** were obtained. A second S_N_Ar was needed to afford compounds **40**, **41**, and **42a**–**47a**. Interestingly, due to the poor reactivity of chloro-pyrimidine **38** toward S_N_Ar, we were only able to prepare compounds **42a** and **43a**. Switching to its fluorinated analogue **39** was necessary to synthesize compounds **40**, **41**, and **44a**–**47a** in good yield (from 50 to 70%). Compounds **42**–**47** were then obtained upon removal of the protecting group from precursors **42a**–**47a**.

**Scheme 3:**
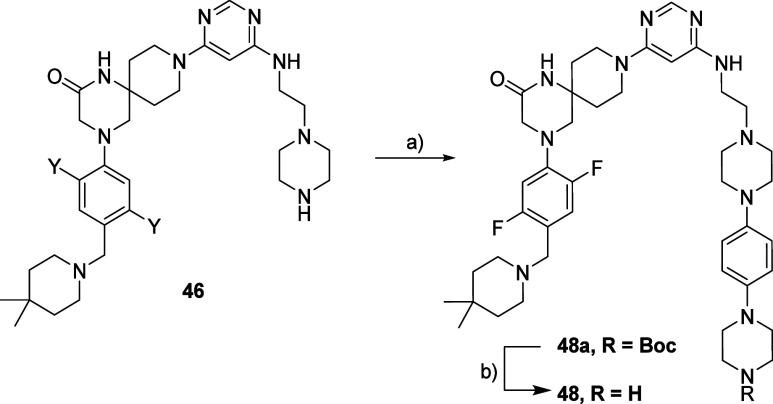
Synthesis Route for Compound **48** Reagents and conditions: (a) *tert*-butyl 4-(5-bromopyrimidin-2-yl) piperazine-1-carboxylate, CuI, (L)-proline, K_2_CO3, DMSO; (b) HCl 4 M in dioxane, MeOH.

Through an Ullmann-type reaction, we combined compound **46** with Boc-protected 4-(5-bromopyrimidin-2-yl) piperazine.^68^ The desired intermediate **48a** was obtained in low yield and upon Boc deprotection afforded compound **48** (Scheme 3).

**Scheme 4:**
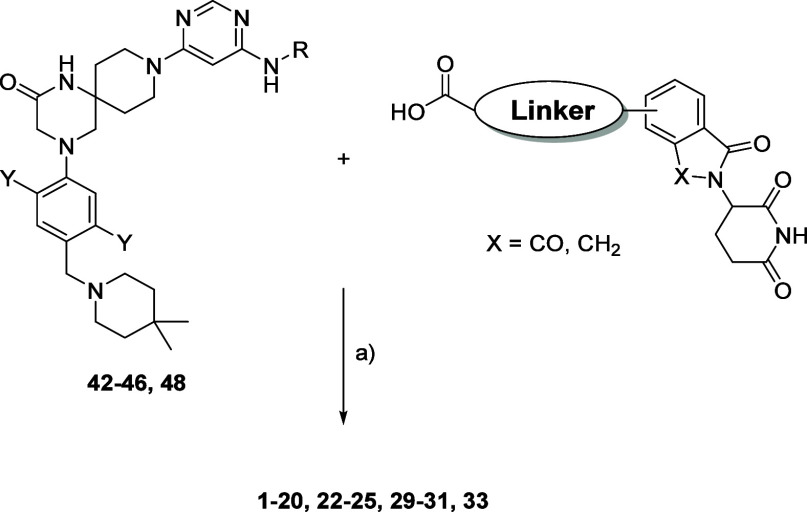
Synthesis Route for Compounds **1**–**20**, **22**–**25**, **29**–**31**, and **33** Reagents and conditions: (a) HATU (**1–4, 8, 9, 29–31**)/COMU (**5–7, 10–28, 32–35**), DIPEA, DMF.

Compounds **1**–**20**, **22**–**25**, **29**–**31**, and **33** were synthesized via an amide coupling reaction between compounds **42**–**46** and **48** and the corresponding pomalidomide/lenalidomide carboxylic acids. HATU coupling agent provided decent yields only for compounds **1**–**4**, **8**, **9**,and **29**–**31**. For the other molecules, COMU performed better and provided the desired products with yields of up to 60% (Scheme 4).

**Scheme 5:**
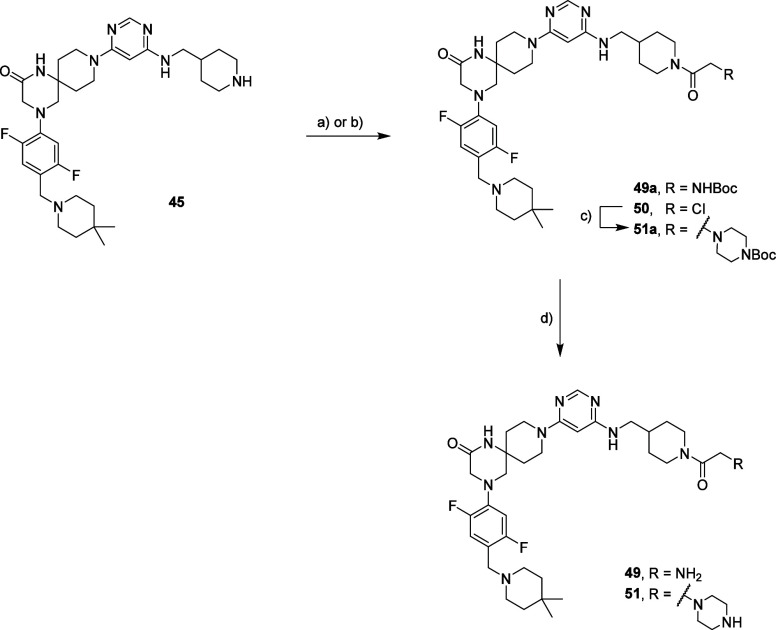
Synthesis Route for Compounds **49** and **51** Reagents and conditions: (a) For **49a**: Boc-glycine, COMU, DIPEA, DMF; (b) for **50**: 2-chloroacetyl chloride, DIPEA, dry THF; (c) for **51a**: Boc-piperazine, DMSO, 50 °C; (d) for **49** and **51**: TFA, DCM.

The final amide coupling (Scheme 4) did not work for compounds **21** and **35**. Therefore, the synthetic route was slightly modified. Both intermediates **49** and **51** were prepared starting from **45**. An amide coupling between the latter and Boc-glycine, followed by amino group deprotection, yielded compound **49**. For the synthesis of compound **51**, the acetylation of **43** using 2-chloroacetyl chloride resulted in the formation of **50**. Afterward, we converted **50** into **51** through an S_N_2 reaction with Boc-piperazine, followed by the removal of the protecting group (Scheme 5).

**Scheme 6:**
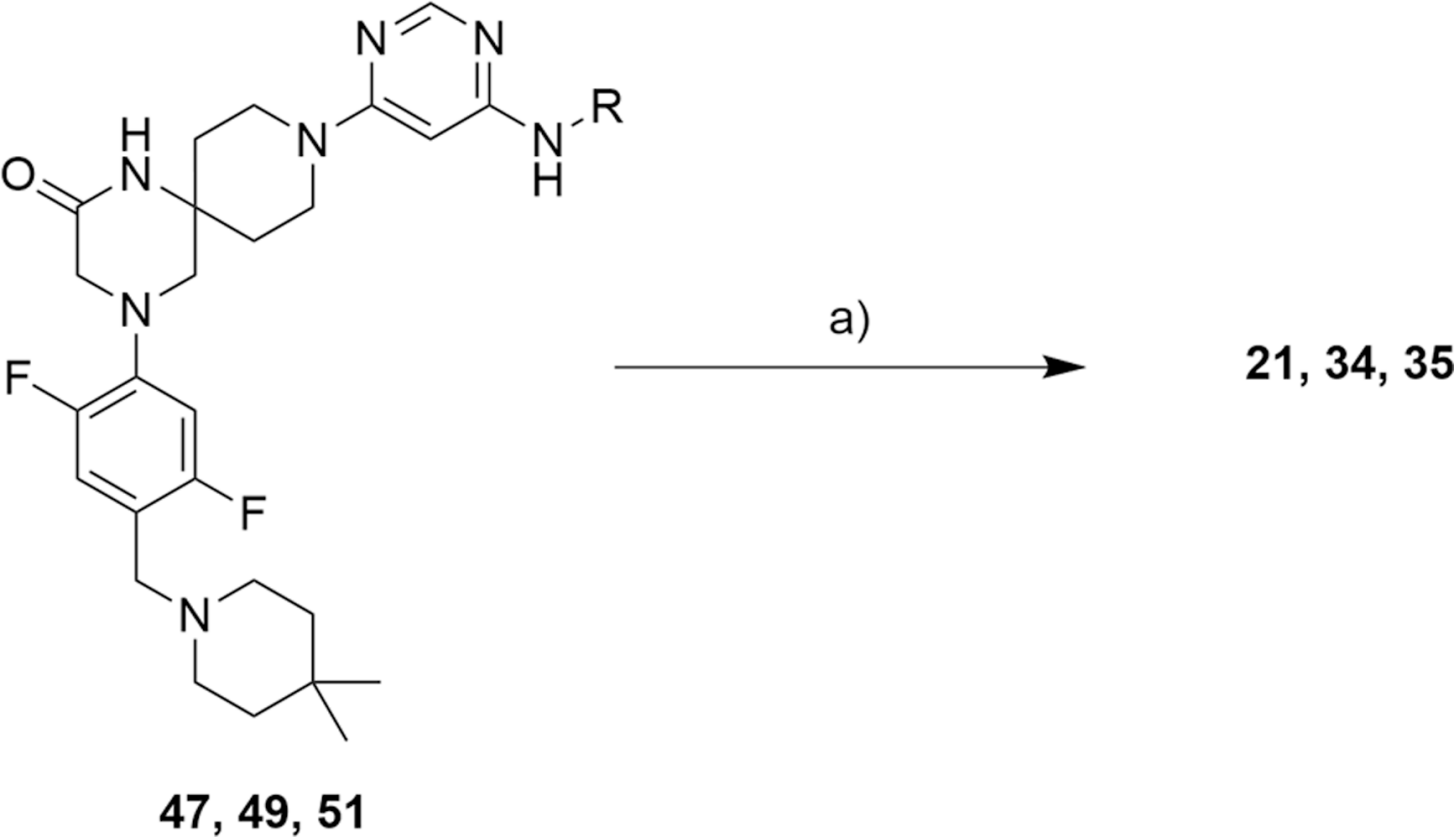
Synthesis Route for Compounds **21**, **34**, and **35** Reagents and conditions: (a) For **21**, **34**, and **35**: 4-fluoro thalidomide, TEA, and DMSO.

With intermediates **49** and **51** in our hands, we were finally able to obtain PROTACs **21** and **35** using S_N_Ar and 4-fluoro thalidomide. Using the same reaction as for the final step, we prepared PROTAC **34** from compound **47** (Scheme 6).

**Scheme 7:**
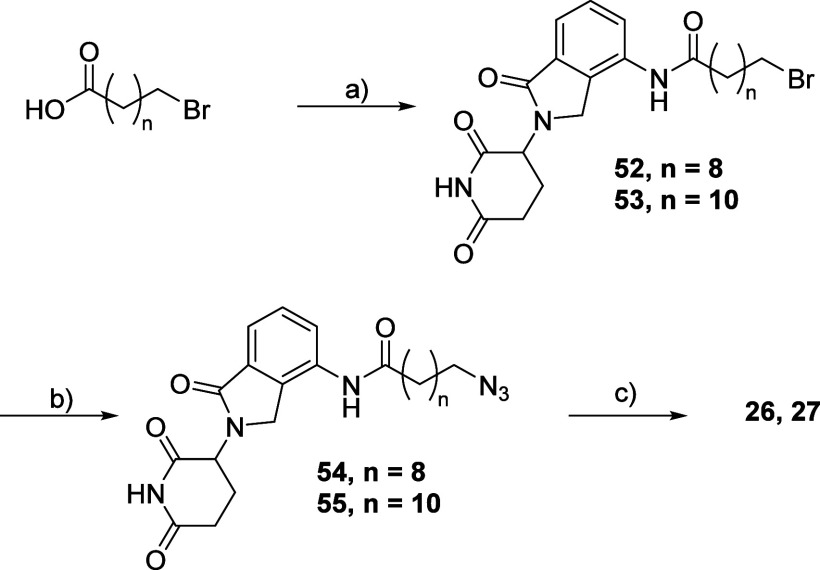
Synthesis Route for Compounds **26** and **27** Reagents and conditions: (a) (i) SOCl_2_; (ii) lenalidomide, THF; (b) NaN_3_, DMF; (c) **40**, CuSO_4_, Na ascorbate, THF.

To synthesize **26** and **27**, compounds **52** and **53** were first prepared starting with lenalidomide, which was reacted with the corresponding carboxylic acids.^69^ A following S_N_2 reaction with NaN_3_ allowed us to prepare intermediates **54** and **55**.^70^ PROTACs **26** and **27** were finally obtained through the click reaction (Scheme 7).

**Scheme 8:**
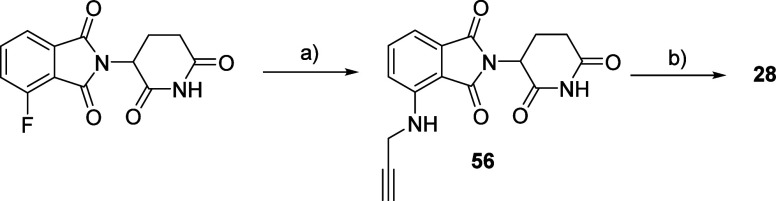
Synthesis Route for Compound **28** Reagents and conditions: (a) propargyl amine, TEA, DMSO; (b) **41**, CuSO_4_, Na ascorbate, THF.

Compound **56** was obtained from a S_N_Ar reaction between 4-fluoro thalidomide and propargyl amine.^71^ Similarly to **26** and **27** (Scheme 7), the click reaction between **56** and **41** was employed to synthesize PROTAC **28** (Scheme 8).

**Scheme 9:**
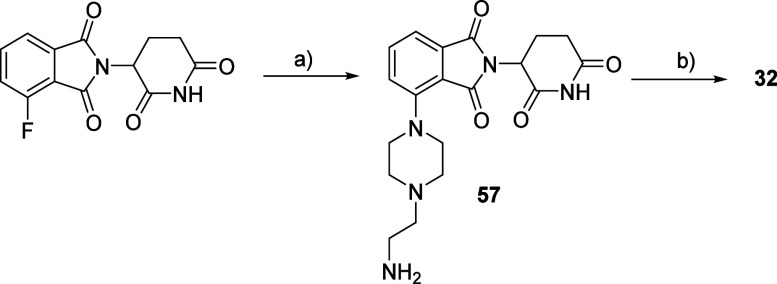
Synthesis Route for Compound **32** Reagents and conditions: (a) (i) *tert*-butyl (2-(piperazin-1-yl) ethyl) carbamate, DIPEA; DMSO; (ii) HCl 4 M in dioxane, MeOH; b) **39**, DIPEA, DMSO.

Following the same procedure used for **56** (Scheme 8), we prepared the protected version of intermediate **57**. After Boc removal, PROTAC **32** was obtained via the S_N_Ar reaction between compounds **57** and **39** (Scheme 9).

**Scheme 10:**
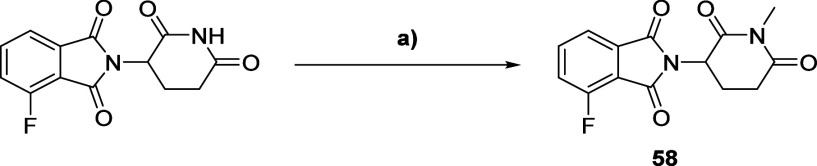
Synthesis Route for Compound **58** Reagents and conditions: (a) CH_3_I, K_2_CO_3_, DMF.

For the synthesis of **me-14** and **me-24** (Scheme 10), 4-fluoro thalidomide was methylated using CH_3_I.^72^ The two PROTACs were then synthesized from **58**, following the synthetic route used for the nonmethylated analogues PROTACs **14** and **24** (Scheme 4).

## Conclusions

Here, we report a medicinal chemistry design of PROTACs against m^6^A-RNA writer METTL3–14. We used as starting information the crystal structure of METTL3–14 in complex with the potent (IC_50_ = 5 nM) and selective inhibitor UZH2. We efficiently optimized the PROTAC linker by first employing the desfluoro derivative of UZH2 as a moiety for METTL3–14. While PEG- and alkyl-based linkers were considered initially, only the PROTACs with alkyl-based linkers demonstrated cell penetration. Subsequently, we synthesized 26 PROTACs based on UZH2 with alkyl linkers of varying lengths. The formation of the ternary complex and ubiquitination of METTL3 were confirmed by a FRET-based assay and an *in vitro* ubiquitination assay. The cellular characterization of the PROTACs is still highly necessary, but biochemical TCFA emerges as a valuable instrument for efficiently screening PROTACs for further validation (Figure 3E). Notably, five PROTACs (**14**, **20**, **22**, **24**, and **30**) with distinct rigid extensions of UZH2 achieved substantial METTL3–14 degradation (50% or higher) in multiple AML cell lines and the prostate cancer cell line PC3, showcasing their potential as valuable tools in targeted protein degradation research. In comparison with the catalytic inhibitor UZH2, the PROTACs **22**, **24**, and **30** show higher antiproliferative activity on prostate cancer PC3 but not on AML cell lines. The elevated level of METTL3–14 in the presence of UZH2 and the lack of reduction of the m^6^A/A level of polyadenylated RNA (measured by LC/MS) suggest that a substantially higher degradation of METTL3–14 (probably above 90%) is required for a strong antiproliferative effect. Similarly, compounds with higher activity will be needed to characterize the potential side effects of METTL3 PROTACs in future *in vivo* studies.

In investigating the features of PROTACs for successful protein degradation, this study adds a valuable contribution to the understanding of the crucial role of the linker. The biochemical and cellular characterizations provide evidence that a minimum linker length is required to achieve the formation of the tripartite complex between PROTAC, E3 ligase CRBN, and POI(s) METTL3–14. In this regard, the linkers of PROTACs **32** and **34** are too short for the formation of a ternary complex that allows for degradation of METTL3–14. The PROTACs that promote a more significant degradation (**14**, **20**, **22**, **23**, **24**, **29**, **30**, **31**, and **33**) are characterized by a rigid “handle” (benzyl, piperidine, and piperazine) and a longer linker. The structure of these PROTACs presents only minimal differences compared to other molecules synthesized during this study (**18**, **19**, **21**, and **25**), which shows how small differences in geometry, length, and/or rigidity of the linker can have a major impact on the formation and stabilization of the ternary complex, and the eventual protein degradation.

## Material and Methods

### METTL3–14 Expression and Purification

For determining the half maximal inhibitory concentration (IC_50_) with the full-length complex, the recombinant complex construct pFastBacDual-StrepII-GFP-TEV-METTL3-His-TEV-METTL14 was expressed using the baculovirus/Sf9 insect cell expression system and purified as described previously.^21^

For ternary complex formation assays, genes containing the methyltransferase domains (MTD) of METTL3 (residues 354–580) and METTL14 (residues 107–395) were cloned into the MacroBac vector 438-GST.^73^ A histidine tag (His) was inserted at the N-terminus of GST by site-directed mutagenesis to yield the construct 438-His-GST-METTL3^MTD^-METTL14^MTD^. Recombinant baculovirus to express the complex of His-GST-METTL3^MTD^ and METTL14^MTD^ was generated by using the Bac-to-Bac system. For protein expression, suspension cultures of Sf9 cells in Sf-90 II SFM medium (Thermo Fisher) were infected at a density of 2 × 10^6^ mL^–1^. Cells were harvested 72 h postinfection, resuspended in buffer A (50 mM Tris-HCl pH 8.0, 500 mM NaCl) supplemented with protease inhibitor cocktail (Roche Diagnostics GmbH, Germany), phenylmethylsulfonyl fluoride (PMSF), salt active nuclease (Merck), and lysed by sonication. The protein complex was purified by Ni-affinity chromatography on a 5 mL HisTrap HP column (Cytiva), equilibrated, washed with buffer A, and eluted with 250 mM imidazole. The complex was further purified by size exclusion chromatography using a Superdex 200 Increase 10/300 GL column (Cytiva) in 20 mM Tris-Cl, pH 8.0, and 200 mM KCl. The complex was aliquoted and flash-frozen in liquid nitrogen and stored at −80 °C until further use.

### Reader-Based TR-FRET Assay

The inhibitory potencies of the PROTACs for METTL3 were quantified by a homogeneous time-resolved fluorescence (HTRF)-based enzyme assay as previously described.^40^ Briefly, the level of m^6^A in an RNA substrate after the reaction catalyzed by METTL3–14 was quantified by measuring specific binding to the m^6^A reader domain of YTHDC1 (residues 345–509) by HTRF. PROTACs that inhibit METTL3 decrease the m^6^A level and, thus, reduce the HTRF signal. Dose–response curves of titrations with the PROTACs were plotted in OriginLab 2018 and fitted with nonlinear regression “log(inhibitor) vs normalized response with variable slope” from which IC_50_ values were determined. Each PROTAC was measured in triplicates on a Corning 384 U bottom white polystyrene plate.

### Biotinylated CRBNTBD Expression and Purification

His-TEV-CRBN^TBD^-Avi was created by inserting a tobacco etch virus (TEV) cleavage site at the N-terminus and an Avi-tag at the C-terminus of the CRBN thalidomide binding domain (TBD, CRBN residues 318–442) and the construct was cloned into the pETDuet-1 vector between SacI and *Hin*dIII restriction sites together with full-length enzyme BirA, which was cloned into the same vector between NdeI and XhoI restriction sites.

The construct was overexpressed in Rosetta (DE3) cells upon induction with 100 μM isopropyl thio-beta-D-galactoside (IPTG) for 20 h at 18 °C. The expression medium was supplemented with 50 μM d-biotin. Harvested cells were resuspended in lysis buffer containing 100 mM Tris-HCl, pH 8.0, 500 mM NaCl, 1 mM 1,4-dithiothreitol (DTT), 1 mM phenylmethylsulfonyl fluoride (PMSF), and 1 mM ethylenediaminetetraacetic acid (EDTA) and lysed by sonication. The lysates were centrifuged at 18,000 rpm at 4 °C for 1 h in an SS-34 rotor. The soluble fraction was then loaded onto a HisTrap FF crude column (GE Healthcare) and washed with lysis buffer supplemented with 50 mM imidazole. The protein was eluted with a buffer containing 250 mM imidazole, 100 mM Tris-HCl, pH 8.0, and 500 mM NaCl. Recombinant TEV protease cleaved the His_6_ tag during overnight dialysis at 4 °C against 100 mM Tris-HCl, pH 8.0, and 500 mM NaCl buffer. The dialyzed sample was passed through the HisTrap FF crude column to remove the His_6_-tagged TEV protease and uncleaved protein. The protein was further purified by size-exclusion chromatography using a HiLoad 16/600 Superdex 200 pg column (GE Healthcare) in 50 mM HEPES, pH 7.5 and 150 mM NaCl buffer. The protein was aliquoted and stored at −80 °C until further use. Biotinylation was confirmed by an avidin shift assay, where a final CRBN-Avi of 5 μM was mixed with different amounts of NeutrAvidin (10, 20, 40 μM) (Thermo Fisher # 31000) and the proteins were analyzed by SDS-PAGE (data not shown).

### Ternary Complex Formation Assay

Ternary complex formation between the PROTACs, METTL3, and CRBN was quantified by a homogeneous time-resolved fluorescence (HTRF)-based enzyme assay. The HTRF signal of a titration series with PROTACs at constant METTL3 and CRBN concentrations underlies the hook effect, leading to a characteristic bell-shaped curve where the concentration of the ternary complex decreases at high PROTAC concentrations. Curves of titrations with the PROTACs were plotted in GraphPad Prism 9.5.1 and fitted with a Gaussian function, if appropriate. His-GST-METTL3^MTD^-METTL14^MTD^ was used at a final concentration of 15 nM. CRBN^TBD^-Avi(biotin) was used at a final concentration of 10 nM. XL665-conjugated streptavidin (Cisbio, 610SAXLB) was used at a final concentration of 1.25 nM. Anti-GST Eu^3+^-labeled antibody (Cisbio, 61GSTKLB) was used at a final concentration of 0.8 nM. The final reaction volume was 20 μL in 50 mM HEPES (pH 7.5), 150 mM NaCl, 0.1% BSA, 100 mM KF. The assays were carried out in triplicate on a Corning 384 U bottom white polystyrene plate (20 ul working volume). The reaction was incubated for at least 3 h at room temperature (RT) in the dark before the HTRF signal was measured using a Tecan Spark plate reader (Tecan). The plate reader recorded with a delay of 100 μs the emission at 620 and 665 nm after excitation of the HTRF donor with UV light at 320 nm. The ratio of the emissions
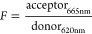
was considered for further analysis. The maximal control contained compound **16**; the blank contained no compounds–this was replaced by the appropriate buffer (with DMSO). The Hook curves were determined by normalization with the maximal control (compound 16) where the maximum of the Hook curve is determined as the fraction of the maximum of compound 16 for each PROTAC. The concentrations resulting in the maximum signal (EC_max_) and the amplitudes of the Hook curves were determined from the parameters of the Gaussian fit (if appropriate) or as the coordinates of the data-point with the highest TR-FRET signal.

For the ternary complex formation assay (TCFA), one of the protein partners must have a GST-tag and the other a biotin-tag. We chose to have the biotin-tag on the CRBN-TBD as it worked well in a previous project (unpublished data). Hence, we chose to put the GST-tag on either METTL3 or METTL14. We cloned, expressed, purified, and tested four N-terminally GST-tagged METTL3–14 constructs for the TCFA: two full-length complexes with either METTL3 or METTL14 GST-tagged and two MTD-only complexes with either METTL3-MTD or METTL14-MTD GST-tagged. The full-length complexes were either prone to aggregation or gave low signal in the TCFA. We attribute this to either folding problems due to the GST-tag or due to too long distances for efficient TR-FRET when the GST-tag is attached to the long unstructured N-terminal tails of the proteins. Hence, we conducted the TCFA with the MTD constructs, which behaved well and gave good TR-FRET signals.

### In Vitro Ubiquitination Assay

For the cell-free in vitro ubiquitination of METTL3-METTL14 purified E1, E2, ubiquitin, CUL4A-RBX1, Cereblon-DDB1, and METTL3-METTL14 were used. Human full-length METTL3-METTL14 was expressed and purified as described above. Human full-length cereblon-DDB1 and 6xHis-CUL4A-6xHis-RBX1 were coexpressed using the baculovirus/Sf9 insect cell expression system and purified by nickel affinity chromatography on a 5 mL HisTrap HP column (Cytiva) followed by anion exchange chromatography on a 5 mL HiTrap Q HP column (Cytiva) and a final gel-filtration step on a HiLoad 16/600 Superdex 200 pg column. Purified recombinant human UBE1 E1 (E-305–025) was purchased from R&D Systems, UbcH5a E2 (23–029) from Merck and ubiquitin (SBB-UP0013) from South Bay Bio. For the ubiquitination reaction, components were mixed to final concentrations of 0.06 μM UBE1, 1.96 μM UbcH5a, 39 μM ubiquitin, 0.33 μM CUL4A-RBX1, 0.33 μM Cereblon-DDB1, 0.5 μM METTL3-METTL14, and different concentrations of compound **14** or **me-14** (0, 2, 8, or 32 μM) in reaction buffer (50 mM Tris-HCl pH 7.6, 5 mM MgCl_2_, 0.2 mM CaCl_2_, 1 mM DTT, 100 mM NaCl, 0.01% BSA, 0.01% Triton X-100). The final reaction volume was 15 μL. After the addition of 2 mM ATP (or the equal volume of water for the control without ATP), the reaction mixture was incubated for 2 h at 30 °C. The reaction was stopped by adding SDS-PAGE loading buffer (final concentration: 60 mM Tris, 1.5% SDS, 10% glycerol, 5% beta-mercaptoethanol, 0.02% bromphenolblue). The samples were then subjected to Western blot analysis for METTL3, METTL14, and ubiquitin. The signals were quantified by using the Image Studio Lite software. To determine the percentage of ubiquitinated METTL3, the fraction of unmodified METTL3 was calculated by dividing the signal of the band assigned to unmodified METTL3 by the signal of the area containing both the unmodified and (poly)ubiquitinated METTL3 and normalizing to the control without ATP. The fraction of ubiquitinated METTL3 is 1 minus the fraction of unmodified METTL3.

### Cell Culture

MOLM-13, NOMO-1, THP-1, Kasumi-1, PC3, and DU145 cell lines were obtained from DSMZ-German Collection of Microorganisms and Cell Cultures GmbH. Cells were cultured in RPMI 1640 medium (11875093, Thermo Fisher Scientific) containing 10% FBS (16140071, Thermo Fisher Scientific) and 1% penicillin-streptomycin (15140122, Thermo Fisher Scientific) in 5% CO_2_ at 37 °C in a humidified incubator, with maintained cell densities at 0.5–1 × 10^6^ cells/mL. All cell lines were tested negative for mycoplasma contamination (PCR-based assay by Microsynth, Switzerland).

### Cell Viability Assay

Cells were seeded in white clear-bottom 96-well plates at a density of 6–20 × 10^3^ cells/well in 50 μL of the complete RPMI medium and treated with 50 μL of increasing concentrations of the indicated compounds dissolved in DMSO (final concentration of compounds 0.01–10 μM) or DMSO only as a negative control (0.01% (v/v)) and incubated for 72 h at 37 °C with 5% CO_2_. Cell viability was determined using a CellTiter-Glo luminescent cell viability assay (Promega) based on the detection of ATP according to the manufacturer’s instructions. 100 μL of the reagent was added to each well and incubated for 10 min at room temperature. The luminescence was recorded using a Tecan Infinite 3046 M1000 microplate reader from the top. Background luminescence value was obtained from wells containing the CellTiter-Glo reagent and medium without cells. The resulting data was analyzed in GraphPad Prism 9.

### Cellular Thermal Shift Assay (CETSA)

One million of MOLM-13 cells were suspended in 100 μL of PBS (10010023, Thermo Fisher Scientific) containing 2× protease inhibitor cocktail (11697498001, Roche), for each condition tested. Cells were incubated with compounds or DMSO control (1% (v/v)) for 1 h at 37 °C. They were then heat treated at 54 °C in a thermoblock for 3 min, followed by cooling to room temperature (3 min). Next, samples were lysed by threefreeze–thaw cycles in liquid nitrogen and centrifuge at 16000 g for 30 min, 4 °C. Equal volumes of control and tested samples (12 μL) were analyzed by Western blot. The changes in the amount of METTL3 protein (after normalization for β-actin and/or GAPDH) were monitored by performing densitometry in Image Studio Lite software and analyzed in GraphPad Prism 9.

### Cellular Degradation Assay

METTL3 (and METTL14) protein degradation was monitored by Western blot. Cells were treated with the indicated concentration of PROTACs or DMSO control (0.1% (v/v)) for 24h, 37 °C with 5% CO_2_. Samples were then collected and lysed with RIPA buffer with added protease inhibitors (11697498001, Roche). After SDS-PAGE, proteins were transferred to a nitrocellulose membrane, blocked (with 5% milk, 0.5% BSA in TBST buffer), and incubated overnight with primary antibodies. The following antibodies were used: GAPDH (no. 2118, Cell Signaling, 1:4000), β-actin (ab8226, Abcam, 1:2000), METTL3 (ab195352, Abcam, 1:1000), and METTL14 (ab220031, Abcam, 1:1000). Membranes were scanned using an LI-COR Odyssey DLx imager after incubation with appropriate secondary antibodies (antimouse IgG IRDye 680RD (926–68072, LI-COR, 1:10000) and goat anti-Rabbit IgG IRDye 800CW (926–32211, LI-COR, 1:10000)). Densitometry was performed in Image Studio Lite software and analyzed in GraphPad Prism 9.

### Quantification of m6A/A Ratio in Polyadenylated RNA by UPLC-MS/MS Analysis

UPLC-MS/MS was performed as previously described.^28^

Briefly, MOLM-13 cells were seeded into 6 well plates at a density of 1 × 10^6^ cells/mL in 2 mL of complete RPMI medium. Cells were treated with the indicated concentrations of compounds or DMSO control (final concentration 0.5% (v/v)) for 24 h. Following the incubation, cells were collected by centrifugation and washed once with PBS, and total RNA was extracted using 0.5 mL of GENEzol reagent according to the manufacturer’s instructions. The final volume of 50 μL of total RNA eluate was subjected to two rounds of purification using 25 μL of Sera-Mag magnetic oligo(dT) particles (Cytiva) per sample. The polyadenylated RNA was eluted with nuclease-free water in a final volume of 25 μL, and its concentration was determined using NanoDrop. One hundred nanograms of mRNA were digested to nucleosides and dephosphorylated in a one-pot reaction using 0.5 μL of nucleoside digestion mix (M0649S, NEB) in 25 μL of total reaction volume for 4 h at 37 °C. The samples were used for UPLC-MS/MS analysis without further purification steps. The data were plotted by using GraphPad Prism 9.

## Supporting Information Available

Supplementary figures, tables, materials, synthetic procedures, characterization data, ^1^H and ^13^C NMR spectra, and HPLC traces for compounds **1**–**35**, **me-14**, and **me-24** (PDF)

## References

[ref1] Kumar S.; Mohapatra T. Deciphering Epitranscriptome: Modification of mRNA Bases Provides a New Perspective for Post-Transcriptional Regulation of Gene Expression. Front. Cell Dev. Biol. 2021, 9, 628415 10.3389/fcell.2021.628415. 33816473 PMC8010680

[ref2] Roignant J. Y.; Soller M. m^6^A in mRNA: An Ancient Mechanism for Fine-Tuning Gene Expression. Trends Genet. 2017, 33 (6), 380–390. 10.1016/j.tig.2017.04.003. 28499622

[ref3] Boccaletto P.; Stefaniak F.; Ray A.; Cappannini A.; Mukherjee S.; Purta E.; Kurkowska M.; Shirvanizadeh N.; Destefanis E.; Groza P.; Avşar G.; Romitelli A.; Pir P.; Dassi E.; Conticello S. G.; Aguilo F.; Bujnicki J. M. MODOMICS: A Database of RNA Modification Pathways. 2021 Update. Nucleic Acids Res. 2021, 50. 10.1093/nar/gkab1083. PMC8728126 34893873

[ref4] Dominissini D.; Moshitch-Moshkovitz S.; Schwartz S.; Salmon-Divon M.; Ungar L.; Osenberg S.; Cesarkas K.; Jacob-Hirsch J.; Amariglio N.; Kupiec M.; Sorek R.; Rechavi G. Topology of the Human and Mouse m^6^A RNA Methylomes Revealed by m^6^A-Seq. 2012, 485 ( (7397), ), 201–206. 10.1038/nature11112. 22575960

[ref5] Alarcón C. R.; Goodarzi H.; Lee H.; Liu X.; Tavazoie S.; Tavazoie S. F. HNRNPA2B1 Is a Mediator of m^6^A-Dependent Nuclear RNA Processing Events. Cell 2015, 162 (6), 1299–1308. 10.1016/j.cell.2015.08.011. 26321680 PMC4673968

[ref6] Li A.; Chen Y. S.; Ping X. L.; Yang X.; Xiao W.; Yang Y.; Sun H. Y.; Zhu Q.; Baidya P.; Wang X.; Bhattarai D. P.; Zhao Y. L.; Sun B. F.; Yang Y. G. Cytoplasmic m^6^A Reader YTHDF3 Promotes mRNA Translation. Cell Res. 2017, 27 (3), 444–447. 10.1038/cr.2017.10. 28106076 PMC5339832

[ref7] Wang X.; Lu Z.; Gomez A.; Hon G. C.; Yue Y.; Han D.; Fu Y.; Parisien M.; Dai Q.; Jia G.; Ren B.; Pan T.; He C. N^6^-Methyladenosine-Dependent Regulation of Messenger RNA Stability. Nature 2014, 505 (7481), 117–120. 10.1038/nature12730. 24284625 PMC3877715

[ref8] Xiao W.; Adhikari S.; Dahal U.; Chen Y. S.; Hao Y. J.; Sun B. F.; Sun H. Y.; Li A.; Ping X. L.; Lai W. Y.; Wang X.; Ma H. L.; Huang C. M.; Yang Y.; Huang N.; Jiang G. B.; Wang H. L.; Zhou Q.; Wang X. J.; Zhao Y. L.; Yang Y. G. Nuclear m^6^A Reader YTHDC1 Regulates mRNA Splicing. Molecular Cell 2016, 61 (4), 507–519. 10.1016/j.molcel.2016.01.012. 26876937

[ref9] Shi H.; Wang X.; Lu Z.; Zhao B. S.; Ma H.; Hsu P. J.; Liu C.; He C. YTHDF3 Facilitates Translation and Decay of N^6^-Methyladenosine-Modified RNA. Cell. Res. 2017, 27 (3), 315–328. 10.1038/cr.2017.15. 28106072 PMC5339834

[ref10] Batista P. J.; Molinie B.; Wang J.; Qu K.; Zhang J.; Li L.; Bouley D. M.; Lujan E.; Haddad B.; Daneshvar K.; Carter A. C.; Flynn R. A.; Zhou C.; Lim K. S.; Dedon P.; Wernig M.; Mullen A. C.; Xing Y.; Giallourakis C. C.; Chang H. Y. m^6^A RNA Modification Controls Cell Fate Transition in Mammalian Embryonic Stem Cells. Cell Stem Cell 2014, 15 (6), 707–719. 10.1016/j.stem.2014.09.019. 25456834 PMC4278749

[ref11] Zhou J.; Wan J.; Gao X.; Zhang X.; Jaffrey S. R.; Qian S. B. Dynamic m^6^A mRNA Methylation Directs Translational Control of Heat Shock Response. Nature 2015, 526 (7574), 591–594. 10.1038/nature15377. 26458103 PMC4851248

[ref12] Fustin J. M.; Doi M.; Yamaguchi Y.; Hida H.; Nishimura S.; Yoshida M.; Isagawa T.; Morioka M. S.; Kakeya H.; Manabe I.; Okamura H. RNA-Methylation-Dependent RNA Processing Controls the Speed of the Circadian Clock. Cell 2013, 155 (4), 793–806. 10.1016/j.cell.2013.10.026. 24209618

[ref13] Barbieri I.; Tzelepis K.; Pandolfini L.; Shi J.; Millán-Zambrano G.; Robson S. C.; Aspris D.; Migliori V.; Bannister A. J.; Han N.; De Braekeleer E.; Ponstingl H.; Hendrick A.; Vakoc C. R.; Vassiliou G. S.; Kouzarides T. Promoter-Bound METTL3Maintains Myeloid Leukaemia by m^6^A-Dependent Translation Control. Nature 2017, 552 (7683), 126–131. 10.1038/nature24678. 29186125 PMC6217924

[ref14] Choe J.; Lin S.; Zhang W.; Liu Q.; Wang L.; Ramirez-Moya J.; Du P.; Kim W.; Tang S.; Sliz P.; Santisteban P.; George R. E.; Richards W. G.; Wong K. K.; Locker N.; Slack F. J.; Gregory R. I. mRNA Circularization by METTL3–EIF3h Enhances Translation and Promotes Oncogenesis. Nature 2018, 561 (7724), 556–560. 10.1038/s41586-018-0538-8. 30232453 PMC6234840

[ref15] Li J.; Xie H.; Ying Y.; Chen H.; Yan H.; He L.; Xu M.; Xu X.; Liang Z.; Liu B.; Wang X.; Zheng X.; Xie L. YTHDF2Mediates the mRNA Degradation of the Tumor Suppressors to Induce AKT Phosphorylation in N6-Methyladenosine-Dependent Way in Prostate Cancer. Mol. Cancer 2020, 19 ( (152), ). 10.1186/s12943-020-01267-6. PMC7599101 33121495

[ref16] Cai X.; Wang X.; Cao C.; Gao Y.; Zhang S.; Yang Z.; Liu Y.; Zhang X.; Zhang W.; Ye L. HBXIP-Elevated Methyltransferase METTL3 Promotes the Progression of Breast Cancer via Inhibiting Tumor Suppressor Let-7g. Cancer Lett. 2018, 415, 11–19. 10.1016/j.canlet.2017.11.018. 29174803

[ref17] Chen M.; Wei L.; Law C. T.; Tsang F. H.-C.; Shen J.; Cheng C. L.-H.; Tsang L.-H.; Ho D. W.-H.; Chiu D. K.-C.; Lee J. M.-F.; Wong C. C.-L.; Ng I. O.-L.; Wong C.-M. RNA N6-Methyladenosine Methyltransferase-like 3 Promotes Liver Cancer Progression through YTHDF2-Dependent Posttranscriptional Silencing of SOCS2. Hepatology 2018, 67 (6), 2254–2270. 10.1002/hep.29683. 29171881

[ref18] Zuo X.; Chen Z.; Gao W.; Zhang Y.; Wang J.; Wang J.; Cao M.; Cai J.; Wu J.; Wang X. M6A-Mediated Upregulation of LINC00958 Increases Lipogenesis and Acts as a Nanotherapeutic Target in Hepatocellular Carcinoma. J. Hematol. Oncol. 2020, 13 ( (5), ). 10.1186/s13045-019-0839-x. PMC6951025 31915027

[ref19] Shen C.; Xuan B.; Yan T.; Ma Y.; Xu P.; Tian X.; Zhang X.; Cao Y.; Ma D.; Zhu X.; Zhang Y.; Fang J. Y.; Chen H.; Hong J. m^6^A-Dependent Glycolysis Enhances Colorectal Cancer Progression. Mol. Cancer 2020, 19 ( (72), ). 10.1186/s12943-020-01190-w. PMC7118901 32245489

[ref20] Li T.; Hu P. S.; Zuo Z.; Lin J. F.; Li X.; Wu Q. N.; Chen Z. H.; Zeng Z. L.; Wang F.; Zheng J.; Chen D.; Li B.; Kang T. B.; Xie D.; Lin D.; Ju H. Q.; Xu R. H. METTL3 Facilitates Tumor Progression via an m^6^A-IGF2BP2-Dependent Mechanism in Colorectal Carcinoma. Mol. Cancer 2019, 18 ( (112), ). 10.1186/s12943-019-1038-7. PMC6589893 31230592

[ref21] Śledź P.; Jinek M. Structural Insights into the Molecular Mechanism of the m^6^A Writer Complex. Elife 2016, 5, e18434 10.7554/eLife.18434. 27627798 PMC5023411

[ref22] Wang P.; Doxtader K. A.; Nam Y. Structural Basis for Cooperative Function of Mettl3 and Mettl14 Methyltransferases. Mol. Cell 2016, 63 (2), 306–317. 10.1016/j.molcel.2016.05.041. 27373337 PMC4958592

[ref23] Sun T.; Wu R.; Ming L. The Role of m6A RNA Methylation in Cancer. Biomed. Pharmacother. 2019, 112, 108613 10.1016/j.biopha.2019.108613. 30784918

[ref24] Lin S.; Choe J.; Du P.; Triboulet R.; Gregory R. I. The m^6^A Methyltransferase METTL3 Promotes Translation in Human Cancer Cells. Mol. Cell 2016, 62 (3), 335–345. 10.1016/j.molcel.2016.03.021. 27117702 PMC4860043

[ref25] Geula S.; Moshitch-Moshkovitz S.; Dominissini D.; Mansour A. A. F.; Kol N.; Salmon-Divon M.; Hershkovitz V.; Peer E.; Mor N.; Manor Y. S.; Ben-Haim M. S.; Eyal E.; Yunger S.; Pinto Y.; Jaitin D. A.; Viukov S.; Rais Y.; Krupalnik V.; Chomsky E.; Zerbib M.; Maza I.; Rechavi Y.; Massarwa R.; Hanna S.; Amit I.; Levanon E. Y.; Amariglio N.; Stern-Ginossar N.; Novershtern N.; Rechavi G.; Hanna J. H. m^6^A mRNA Methylation Facilitates Resolution of Naïve Pluripotency toward Differentiation. Science 2015, 347 (6225), 1002–1006. 10.1126/science.1261417. 25569111

[ref26] Vu L. P.; Pickering B. F.; Cheng Y.; Zaccara S.; Nguyen D.; Minuesa G.; Chou T.; Chow A.; Saletore Y.; Mackay M.; Schulman J.; Famulare C.; Patel M.; Klimek V. M.; Garrett-Bakelman F. E.; Melnick A.; Carroll M.; Mason C. E.; Jaffrey S. R.; Kharas M. G. The N^6^-Methyladenosine (m^6^A)-Forming Enzyme METTL3 Controls Myeloid Differentiation of Normal Hematopoietic and Leukemia Cells. Nat. Med. 2017, 23 (11), 1369–1376. 10.1038/nm.4416. 28920958 PMC5677536

[ref27] Dolbois A.; Bedi R. K.; Bochenkova E.; Müller A.; Moroz-Omori E. V.; Huang D.; Caflisch A. 1,4,9-Triazaspiro[5.5]Undecan-2-One Derivatives as Potent and Selective METTL3 Inhibitors. J. Med. Chem. 2021, 64 (17), 12738–12760. 10.1021/acs.jmedchem.1c00773. 34431664

[ref28] Moroz-Omori E. V.; Huang D.; Kumar Bedi R.; Cheriyamkunnel S. J.; Bochenkova E.; Dolbois A.; Rzeczkowski M. D.; Li Y.; Wiedmer L.; Caflisch A. METTL3 Inhibitors for Epitranscriptomic Modulation of Cellular Processes. ChemMedChem. 2021, 16 (19), 3035–3043. 10.1002/cmdc.202100291. 34237194 PMC8518639

[ref29] Yankova E.; Blackaby W.; Albertella M.; Rak J.; De Braekeleer E.; Tsagkogeorga G.; Pilka E. S.; Aspris D.; Leggate D.; Hendrick A. G.; Webster N. A.; Andrews B.; Fosbeary R.; Guest P.; Irigoyen N.; Eleftheriou M.; Gozdecka M.; Dias J. M. L.; Bannister A. J.; Vick B.; Jeremias I.; Vassiliou G. S.; Rausch O.; Tzelepis K.; Kouzarides T. Small-Molecule Inhibition of METTL3 as a Strategy against Myeloid Leukaemia. Nature 2021, 593 (7860), 597–601. 10.1038/s41586-021-03536-w. 33902106 PMC7613134

[ref30] Bedi R. K.; Huang D.; Li Y.; Caflisch A. Structure-Based Design of Inhibitors of the m(6)A-RNA Writer Enzyme METTL3. ACS Bio & Med. Chem. Au 2023, 3 (4), 359–370. 10.1021/acsbiomedchemau.3c00023. PMC10436262 37599794

[ref31] Sturgess K.; Yankova E.; Vijayabaskar M. S.; Isobe T.; Rak J.; Kucinski I.; Barile M.; Webster N. A.; Eleftheriou M.; Hannah R.; Gozdecka M.; Vassiliou G.; Rausch O.; Wilson N. K.; Göttgens B.; Tzelepis K. Pharmacological Inhibition of METTL3 Impacts Specific Haematopoietic Lineages. Leukemia 2023, 37, 2133–2137. 10.1038/s41375-023-01965-2. 37464070 PMC10539174

[ref32] Finkelstein J. D.; Martin J. J. Methionine Metabolism in Mammals. Distribution of Homocysteine between Competing Pathways. J. Biol. Chem. 1984, 259 (15), 9508–9513. 10.1016/S0021-9258(17)42728-1. 6746658

[ref33] Pei H.; Peng Y.; Zhao Q.; Chen Y. Small Molecule PROTACs: An Emerging Technology for Targeted Therapy in Drug Discovery. RSC Adv. 2019, 9 (30), 16967 10.1039/C9RA03423D. 35519875 PMC9064693

[ref34] Békés M.; Langley D. R.; Crews C. M. PROTAC Targeted Protein Degraders: The Past Is Prologue. Nature Reviews Drug Discovery 2022 21:3 2022, 21 (3), 181–200. 10.1038/s41573-021-00371-6. PMC8765495 35042991

[ref35] Zou Y.; Ma D.; Wang Y. The PROTAC Technology in Drug Development. Cell. Biochem. Funct. 2019, 37 (1), 21–30. 10.1002/cbf.3369. 30604499 PMC6590639

[ref36] Bond M. J.; Crews C. M. Proteolysis Targeting Chimeras (PROTACs) Come of Age: Entering the Third Decade of Targeted Protein Degradation. RSC Chem. Biol. 2021, 2 (3), 725–742. 10.1039/D1CB00011J. 34212149 PMC8190915

[ref37] Webb T.; Craigon C.; Ciulli A. Targeting Epigenetic Modulators Using PROTAC Degraders: Current Status and Future Perspective. Bioorg. Med. Chem. Lett. 2022, 63, 128653 10.1016/j.bmcl.2022.128653. 35257896

[ref38] Winter G. E.; Buckley D. L.; Paulk J.; Roberts J. M.; Souza A.; Dhe-Paganon S.; Bradner J. E. Phthalimide Conjugation as a Strategy for in Vivo Target Protein Degradation. Science 2015, 348 (6241), 1376–1381. 10.1126/science.aab1433. 25999370 PMC4937790

[ref39] Han X.; Zhao L.; Xiang W.; Qin C.; Miao B.; McEachern D.; Wang Y.; Metwally H.; Wang L.; Matvekas A.; Wen B.; Sun D.; Wang S. Strategies toward Discovery of Potent and Orally Bioavailable Proteolysis Targeting Chimera Degraders of Androgen Receptor for the Treatment of Prostate Cancer. J. Med. Chem. 2021, 64 (17), 12831–12854. 10.1021/acs.jmedchem.1c00882. 34431670 PMC8880306

[ref40] Wiedmer L.; Eberle S. A.; Bedi R. K.; Śledź P.; Caflisch A. A Reader-Based Assay for m^6^A Writers and Erasers. Anal. Chem. 2019, 91 (4), 3078–3084. 10.1021/acs.analchem.8b05500. 30715855

[ref41] Bemis T. A.; La Clair J. J.; Burkart M. D. Unraveling the Role of Linker Design in Proteolysis Targeting Chimeras. J. Med. Chem. 2021, 64 (12), 8042–8052. 10.1021/acs.jmedchem.1c00482. 34106704 PMC10790565

[ref42] Shi Y.; Shi B.; Dass A.; et al. Stable Upconversion Nanohybrid Particles for Specific Prostate Cancer Cell Immunodetection. Sci. Rep. 2016, 6, 37533 10.1038/srep37533. 27874051 PMC5118722

[ref43] Zhang P.; Jain P.; Tsao C.; Wu K.; Jiang S. Proactively Reducing Anti-Drug Antibodies via Immunomodulatory Bioconjugation. Angew. Chem., Int. Ed. 2019, 58 (8), 2433–2436. 10.1002/anie.201814275. 30632270

[ref44] Jones M. W.; Strickland R. A.; Schumacher F. F.; Caddick S.; Baker J. R.; Gibson M. I.; Haddleton D. M. Polymeric Dibromomaleimides as Extremely Efficient Disulfide Bridging Bioconjugation and Pegylation Agents. J. Am. Chem. Soc. 2012, 134 (3), 1847–1852. 10.1021/ja210335f. 22188166

[ref45] Han B. A Suite of Mathematical Solutions to Describe Ternary Complex Formation and Their Application to Targeted Protein Degradation by Heterobifunctional Ligands. J. Biol. Chem. 2020, 295 (45), 15280–15291. 10.1074/jbc.RA120.014715. 32859748 PMC7650257

[ref46] Gadd M.; Testa A.; Lucas X.; et al. Structural basis of PROTAC cooperative recognition for selective protein degradation. Nat. Chem. Biol. 2017, 13, 514–521. 10.1038/nchembio.2329. 28288108 PMC5392356

[ref47] Sun X.; Gao H.; Yang Y.; He M.; Wu Y.; Song Y.; Tong Y.; Rao Y. PROTACs: Great Opportunities for Academia and Industry. Signal Transduct. Target. Ther. 2019, 4 ( (64), ). 10.1038/s41392-019-0101-6. PMC6927964 31885879

[ref48] Alshareef A.; Zhang H. F.; Huang Y. H.; et al. The use of cellular thermal shift assay (CETSA) to study Crizotinib resistance in ALK-expressing human cancers. Sci. Rep. 2016, 6, 33710 10.1038/srep33710. 27641368 PMC5027386

[ref49] Borsari C.; Trader D. J.; Tait A.; Costi M. P. Designing Chimeric Molecules for Drug Discovery by Leveraging Chemical Biology. J. Med. Chem. 2020, 63 (5), 1908–1928. 10.1021/acs.jmedchem.9b01456. 32023055 PMC7997565

[ref50] Hendrick C. E.; Jorgensen J. R.; Chaudhry C.; Strambeanu I. I.; Brazeau J. F.; Schiffer J.; Shi Z.; Venable J. D.; Wolkenberg S. E. Direct-to-Biology Accelerates PROTAC Synthesis and the Evaluation of Linker Effects on Permeability and Degradation. ACS Med. Chem. Lett. 2022, 13 (7), 1182–1190. 10.1021/acsmedchemlett.2c00124. 35859867 PMC9290060

[ref51] Donoghue C.; Cubillos-Rojas M.; Gutierrez-Prat N.; Sanchez-Zarzalejo C.; Verdaguer X.; Riera A.; Nebreda A. R. Optimal Linker Length for Small Molecule PROTACs That Selectively Target P38a and P38b for Degradation. Eur. J. Med. Chem. 2020, 201, 112451 10.1016/j.ejmech.2020.112451. 32634680

[ref52] Liu X.; Kalogeropulou A. F.; Domingos S.; Makukhin N.; Nirujogi R. S.; Singh F.; Shpiro N.; Saalfrank A.; Sammler E.; Ganley I. G.; Moreira R.; Alessi D. R.; Ciulli A. Discovery of XL01126: A Potent, Fast, Cooperative, Selective, Orally Bioavailable, and Blood-Brain Barrier Penetrant PROTAC Degrader of Leucine-Rich Repeat Kinase 2. J. Am. Chem. Soc. 2022, 144 (37), 16930–16952. 10.1021/jacs.2c05499. 36007011 PMC9501899

[ref53] Xiang W.; Zhao L.; Han X.; Xu T.; Kregel S.; Wang M.; Miao B.; Qin C.; Wang M.; McEachern D.; Lu J.; Bai L.; Yang C. Y.; Kirchhoff P. D.; Takyi-Williams J.; Wang L.; Wen B.; Sun D.; Ator M.; Mckean R.; Chinnaiyan A. M.; Wang S. Discovery of ARD-1676 as a Highly Potent and Orally Efficacious AR PROTAC Degrader with a Broad Activity against AR Mutants for the Treatment of AR + Human Prostate Cancer. J. Med. Chem. 2023, 66 (18), 13280–13303. 10.1021/acs.jmedchem.3c01264. 37683104

[ref54] García Jiménez D.; Rossi Sebastiano M.; Vallaro M.; Mileo V.; Pizzirani D.; Moretti E.; Ermondi G.; Caron G. Designing Soluble PROTACs: Strategies and Preliminary Guidelines. J. Med. Chem. 2022, 65 (19), 12639–12649. 10.1021/acs.jmedchem.2c00201. 35469399 PMC9574862

[ref55] Khan S.; He Y.; Zhang X.; Yuan Y.; Pu S.; Kong Q.; Zheng G.; Zhou D. Proteolysis TArgeting Chimeras (PROTACs) as Emerging Anticancer Therapeutics. Oncogene 2020, 39, 4909–4924. 10.1038/s41388-020-1336-y. 32475992 PMC7319888

[ref56] Desantis J.; Mammoli A.; Eleuteri M.; Coletti A.; Croci F.; Macchiarulo A.; Goracci L. PROTACs bearing piperazine-containing linkers: what effect on their protonation state?. RSC Adv. 2022, 12, 21968–21977. 10.1039/D2RA03761K. 36043064 PMC9361468

[ref57] Han X.; Zhao L.; Xiang W.; Miao B.; Qin C.; Wang M.; Xu T.; McEachern D.; Lu J.; Wang Y.; Metwally H.; Yang C. Y.; Kirchhoff P. D.; Wang L.; Matvekas A.; Takyi-Williams J.; Wen B.; Sun D.; Ator M.; Mckean R.; Wang S. Discovery of ARD-2051 as a Potent and Orally Efficacious Proteolysis Targeting Chimera (PROTAC) Degrader of Androgen Receptor for the Treatment of Advanced Prostate Cancer. J. Med. Chem. 2023, 66 (13), 8822–8843. 10.1021/acs.jmedchem.3c00405. 37382562 PMC10568492

[ref58] Li K.; Crews C. M. PROTACs: Past, Present and Future. Chem. Soc. Rev. 2022, 51 (12), 5214–5236. 10.1039/D2CS00193D. 35671157 PMC10237031

[ref59] Li Y.; He X.; Lu X.; Gong Z.; Li Q.; Zhang L.; Yang R.; Wu C.; Huang J.; Ding J.; He Y.; Liu W.; Chen C.; Cao B.; Zhou D.; Shi Y.; Chen J.; Wang C.; Zhang S.; Zhang J.; Ye J.; You H. METTL3 Acetylation Impedes Cancer Metastasis via Fine-Tuning Its Nuclear and Cytosolic Functions. Nat. Commun. 2022, 13 ( (6350), ). 10.1038/s41467-022-34209-5. PMC9605963 36289222

[ref60] Yuan Y.; Du Y.; Wang L.; Liu X. The M6A Methyltransferase METTL3 Promotes the Development and Progression of Prostate Carcinoma via Mediating MYC Methylation. J. Cancer 2020, 11 (12), 3588–3595. 10.7150/jca.42338. 32284755 PMC7150444

[ref61] Xiao H.; Zhao R.; Meng W.; Liao Y. Effects and Translatomics Characteristics of a Small-Molecule Inhibitor of METTL3 against Non-Small Cell Lung Cancer. J. Pharm. Anal. 2023, 13 (6), 625–639. 10.1016/j.jpha.2023.04.009. 37440912 PMC10334285

[ref62] Fischer E. S.; Böhm K.; Lydeard J. R.; Yang H.; Stadler M. B.; Cavadini S.; Nagel J.; Serluca F.; Acker V.; Lingaraju G. M.; Tichkule R. B.; Schebesta M.; Forrester W. C.; Schirle M.; Hassiepen U.; Ottl J.; Hild M.; Beckwith R. E. J.; Harper J. W.; Jenkins J. L.; Thomä N. H. Structure of the DDB1-CRBN E3 Ubiquitin Ligase in Complex with Thalidomide. Nature 2014, 512, 49–53. 10.1038/nature13527. 25043012 PMC4423819

[ref63] Xie S.; Sun Y.; Liu Y.; Li X.; Li X.; Zhong W.; Zhan F.; Zhu J.; Yao H.; Yang D. H.; Chen Z. S.; Xu J.; Xu S. Development of Alectinib-Based PROTACs as Novel Potent Degraders of Anaplastic Lymphoma Kinase (ALK). J. Med. Chem. 2021, 64 (13), 9120–9140. 10.1021/acs.jmedchem.1c00270. 34176264

[ref64] Toure M.; Crews C. M. Small-Molecule PROTACS: New Approaches to Protein Degradation. Angew. Chem., Int. Ed. 2016, 55 (6), 1966–1973. 10.1002/anie.201507978. 26756721

[ref65] Zeng Z.-C.; Pan Q.; Sun Y.-M.; Huang H.-J.; Chen X.-T.; Chen T.-Q.; He B.; Ye H.; Zhu S.-X.; Pu K.-J.; Fang K.; Huang W.; Chen Y.-Q.; Wang W.-T. METTL3 Protects METTL14 from STUB1-Mediated Degradation to Maintain m^6^A Homeostasis. EMBO Rep. 2023, 24 (3), e55762 10.15252/embr.202255762. 36597993 PMC9986817

[ref66] Zhang N.; Hou D.; Hu X.; Liang J.; Wang M.; Song Z.; Yi L.; Wang Z.; An H.; Xu W.; Wang H. Nano Proteolysis Targeting Chimeras (PROTACs) with Anti-Hook Effect for Tumor Therapy. Angew. Chem., Int. Ed. 2023, 62 (37), e202308049 10.1002/anie.202308049. 37486792

[ref67] Casement R.; Bond A.; Craigon C.; Ciulli A. Mechanistic and Structural Features of PROTAC Ternary Complexes. Methods Mol. Biol. 2021, 2365, 79–113. 10.1007/978-1-0716-1665-9_5. 34432240

[ref68] Hanthorn J. J.; Valgimigli L.; Pratt D. A. Preparation of Highly Reactive Pyridine-and Pyrimidine-Containing Diarylamine Antioxidants. J. Org. Chem. 2012, 77 (16), 6908–6916. 10.1021/jo301013c. 22788575

[ref69] Zhang W.; Li P.; Sun S.; Jia C.; Yang N.; Zhuang X.; Zheng Z.; Li S. Discovery of Highly Potent and Selective CRBN-Recruiting EGFR L858R/T790M Degraders in Vivo. Eur. J. Med. Chem. 2022, 238, 114509 10.1016/j.ejmech.2022.114509. 35691176

[ref70] Zhang H.; Zhao H. Y.; Xi X. X.; Liu Y. J.; Xin M.; Mao S.; Zhang J. J.; Lu A. X.; Zhang S. Q. Discovery of Potent Epidermal Growth Factor Receptor (EGFR) Degraders by Proteolysis Targeting Chimera (PROTAC). European Journal of Medicinal Chemistry 2020, 208, 112061 10.1016/j.ejmech.2020.112061. 31951960

[ref71] Brownsey D. K.; Rowley B. C.; Gorobets E.; Gelfand B. S.; Derksen D. J. Rapid Synthesis of Pomalidomide-Conjugates for the Development of Protein Degrader Libraries. Chem. Sci. 2021, 12, 4519–4525. 10.1039/D0SC05442A. 34163717 PMC8179520

[ref72] Liu J.; Yuan L.; Ruan Y.; Deng B.; Yang Z.; Ren Y.; Li L.; Liu T.; Zhao H.; Mai R.; Chen J. Novel CRBN-Recruiting Proteolysis-Targeting Chimeras as Degraders of Stimulator of Interferon Genes with In Vivo Anti-Inflammatory Efficacy. J. Med. Chem. 2022, 65 (9), 6593–6611. 10.1021/acs.jmedchem.1c01948. 35452223

[ref73] Gradia S. D.; Ishida J. P.; Tsai M.-S.; Jeans C.; Tainer J. A.; Fuss J. O. MacroBac: New Technologies for Robust and Efficient Large-Scale Production of Recombinant Multiprotein Complexes. Meth. Enzymol. 2017, 592, 1–26. 10.1016/bs.mie.2017.03.008. PMC6028233 28668116

