# Supplementary material for: Proteolysis Targeting Chimera Degraders of the METTL3–14 m6A-RNA Methyltransferase
Source: JACS Au. 2024 Feb 12;4(2):713–29. doi: 10.1021/jacsau.4c00040 (PMC10900215; doi:10.1021/jacsau.4c00040)

Supporting information for

PROTAC degraders of the METTL3-14 m^6A-RNA methyltransferase

Francesco Errani,[#§] Annalisa Invernizzi,[#§] Marcin Herok,[#] Elena Bochenkova,[#] Fiona Stamm,[#] Ivan Corbeski,[#] Valeria Romanucci,[†] Giovanni Di Fabio,[†] František Zálešák,[#]* Amedeo Caflisch[#]*

[#] Department of Biochemistry, University of Zurich, Winterthurerstrasse 190, CH-8057, Zurich, Switzerland

[†] Università degli Studi di Napoli Federico II, Via Cintia 4, I-80126, Napoli, Italia

[§] Co-first author

* Corresponding Authors

Amedeo Caflisch – **Email:** caflisch@bioc.uzh.ch

František Zálešák – **Email :** f.zalesak@bioc.uzh.ch

Table of Contents

Supplementary Figures 3
Supplementary Tables 9
Chemistry 11
Materials and Methods 11
Compounds purity 11
Experimental section 12
References 68
^{1}H and ^{13}C NMR spectra of target compounds **1-35**, **me-14** and **me-24** 69
HPLC traces of target compounds **1-35**, **me-14** and **me-24**. 106

Supplementary Figures

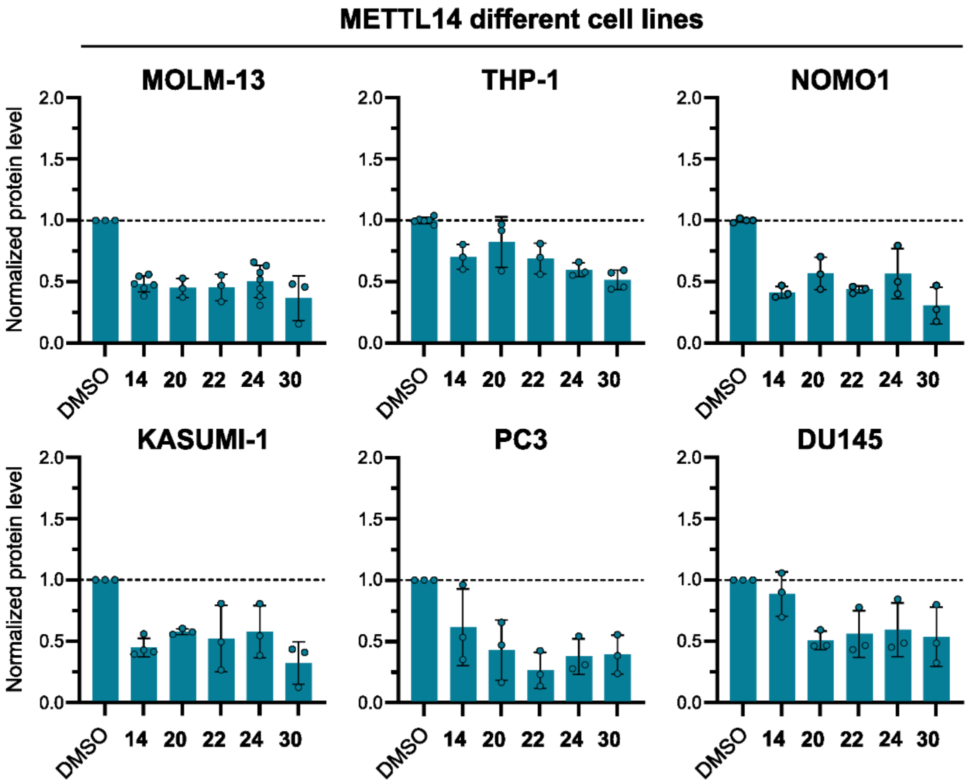

Figure S1. METTL14 Western blot quantification by densitometry from a cellular degradation assay with PROTACs **14**, **20**, **22**, **24** and **30** in the AML cell lines MOLM-13, THP-1, NOMO1, KASUMI-1 and in the prostate cancer cell lines PC3, DU145. The dashed line represents the protein level of the DMSO control, used for normalization (y = 1).

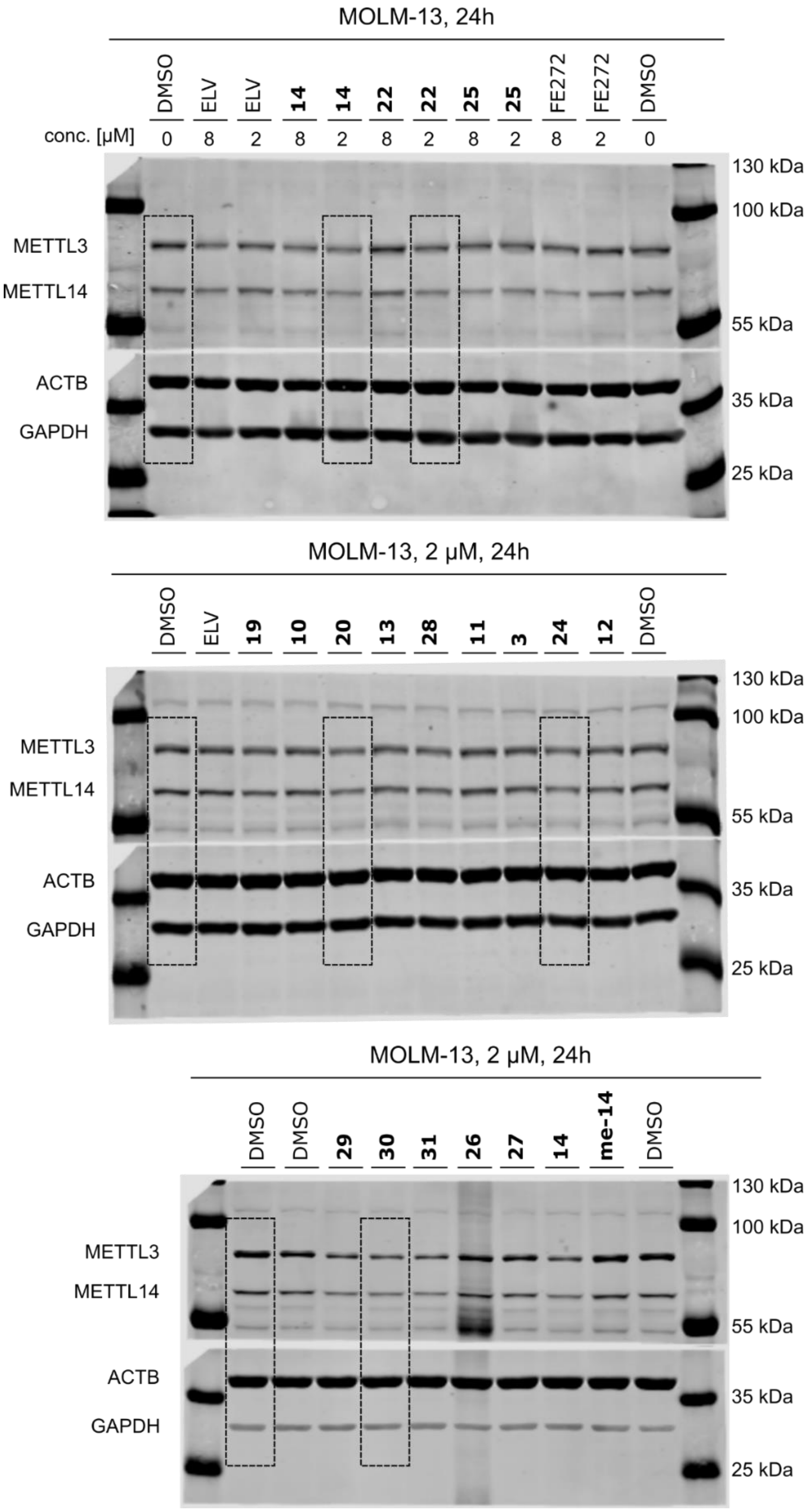

Figure S2. Full Western blot membranes for Figure 3A. Representative Western blots for data in Figure 3B (MOLM-13). Degradation of METTL3 and METTL14 by selected PROTACs **14**, **20**, **22**, **24** and **30** in MOLM-13 cells after 24h and 2 µM compounds concentration.

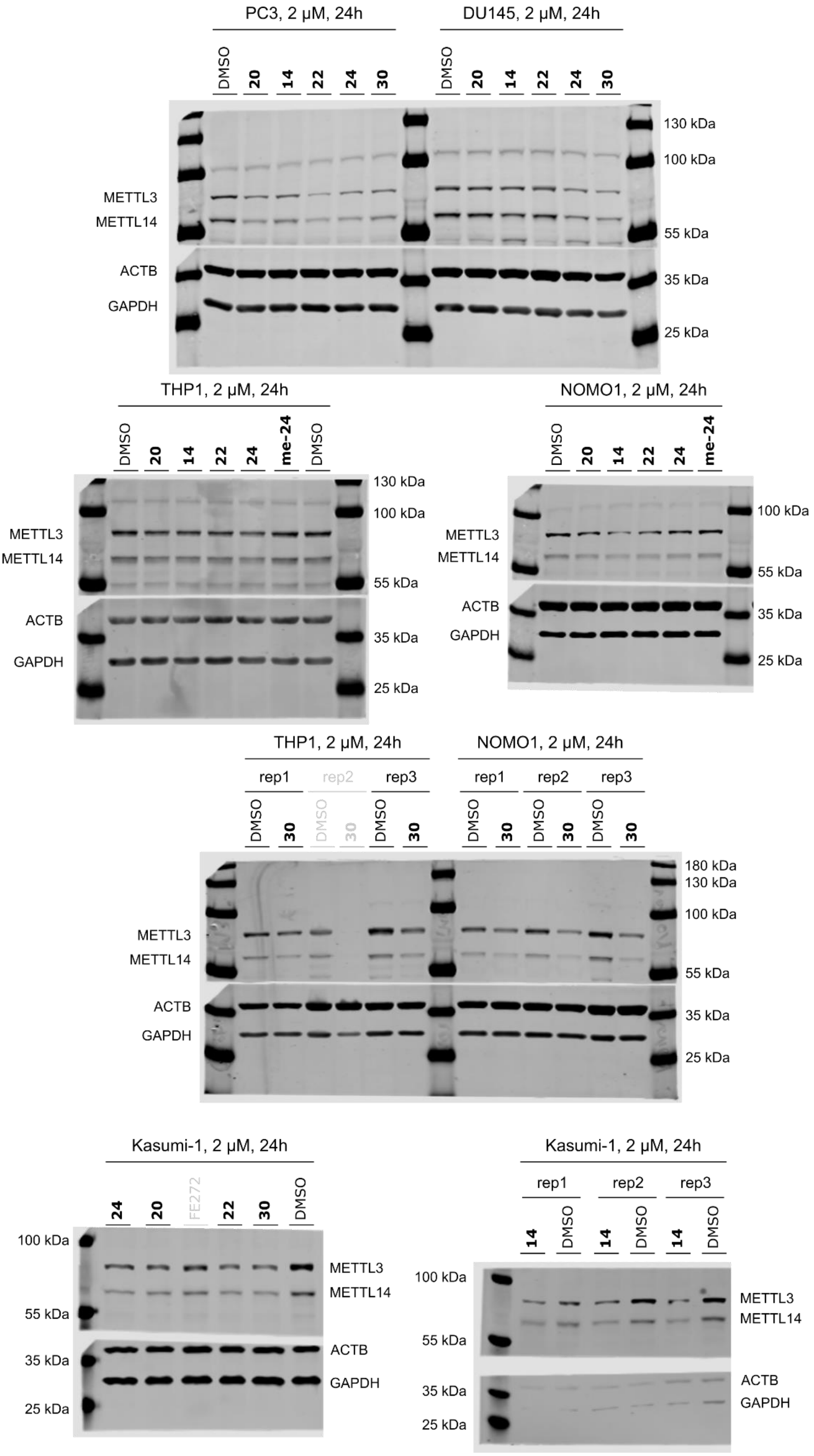

Figure S3. Representative Western blots for data in Figure 3B. Degradation of METTL3 and METTL14 by selected PROTACs **14**, **20**, **22**, **24** and **30** in the prostate cancer cell lines PC3, DU145 and in the AML cell lines THP-1, NOMO-1, KASUMI-1 after 24h and 2 µM compounds concentration.

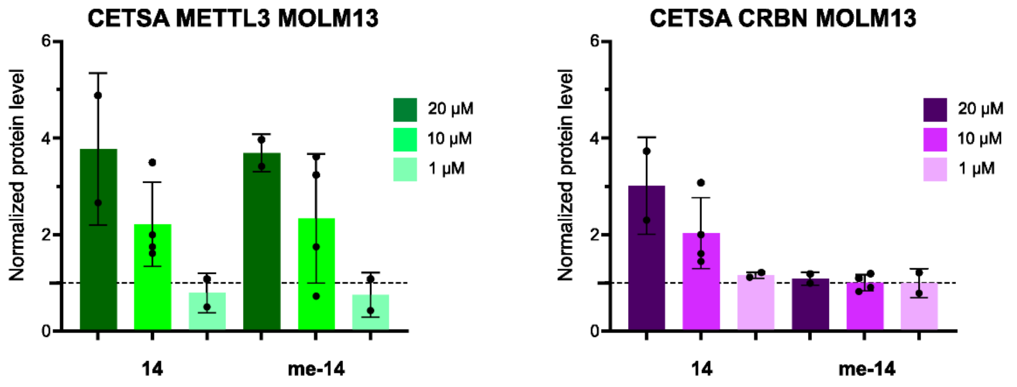

Figure S4. Evaluation of compound **14** and its negative control **me-14** in MOLM-13 AML cell line. The stabilization of METTL3 (left) and Cereblon (CRBN, right) was quantified by CETSA at 54°C. Both **14** and **me-14** stabilize METTL3 in a concentration-dependent manner. The stabilization of CRBN is visible only with compound **14**. The dashed line represents the protein level of the DMSO control, used for normalization (y = 1).

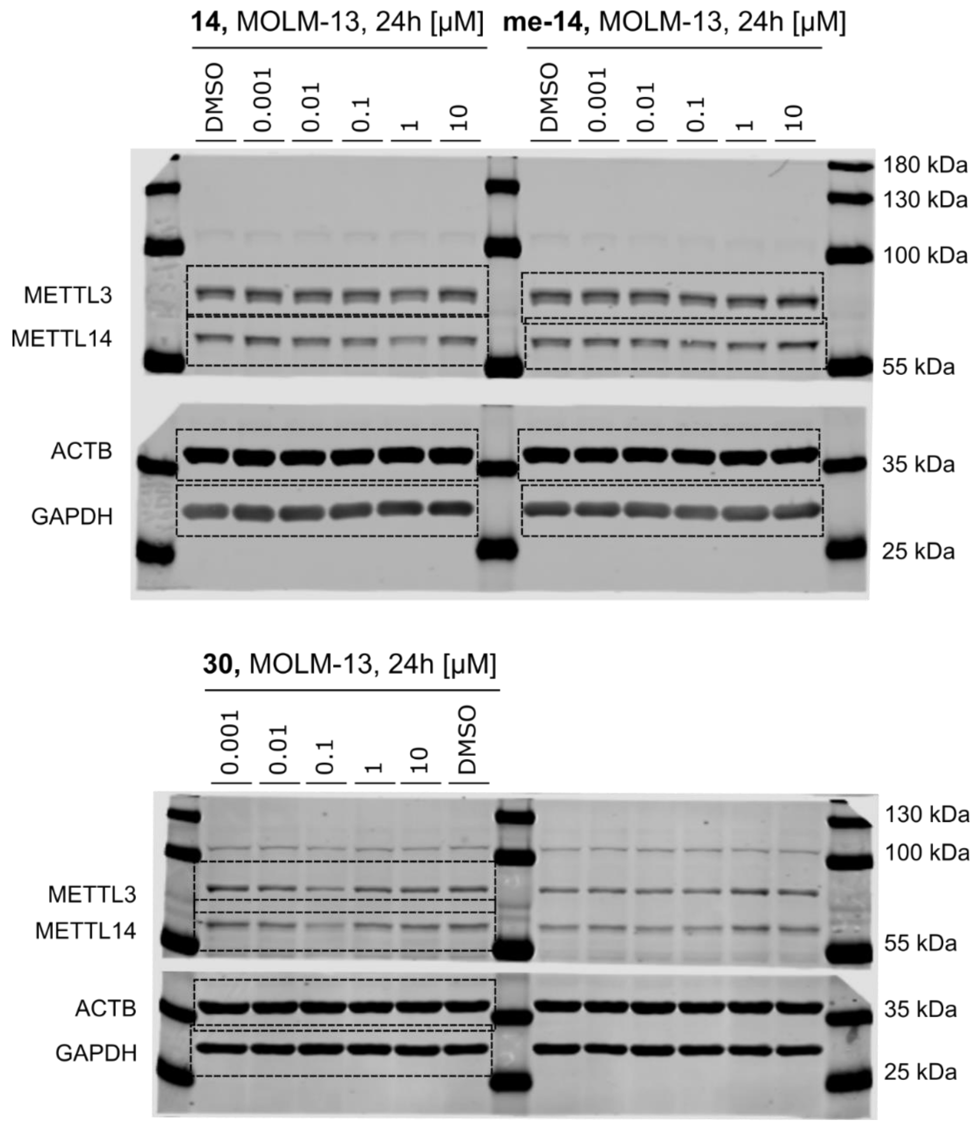

Figure S5. Full Western blot membranes for Figure 5B. Concentration dependence of METTL3 and METTL14 degradation by PROTACs **14**, **me-14** and **30** in MOLM-13 cells.

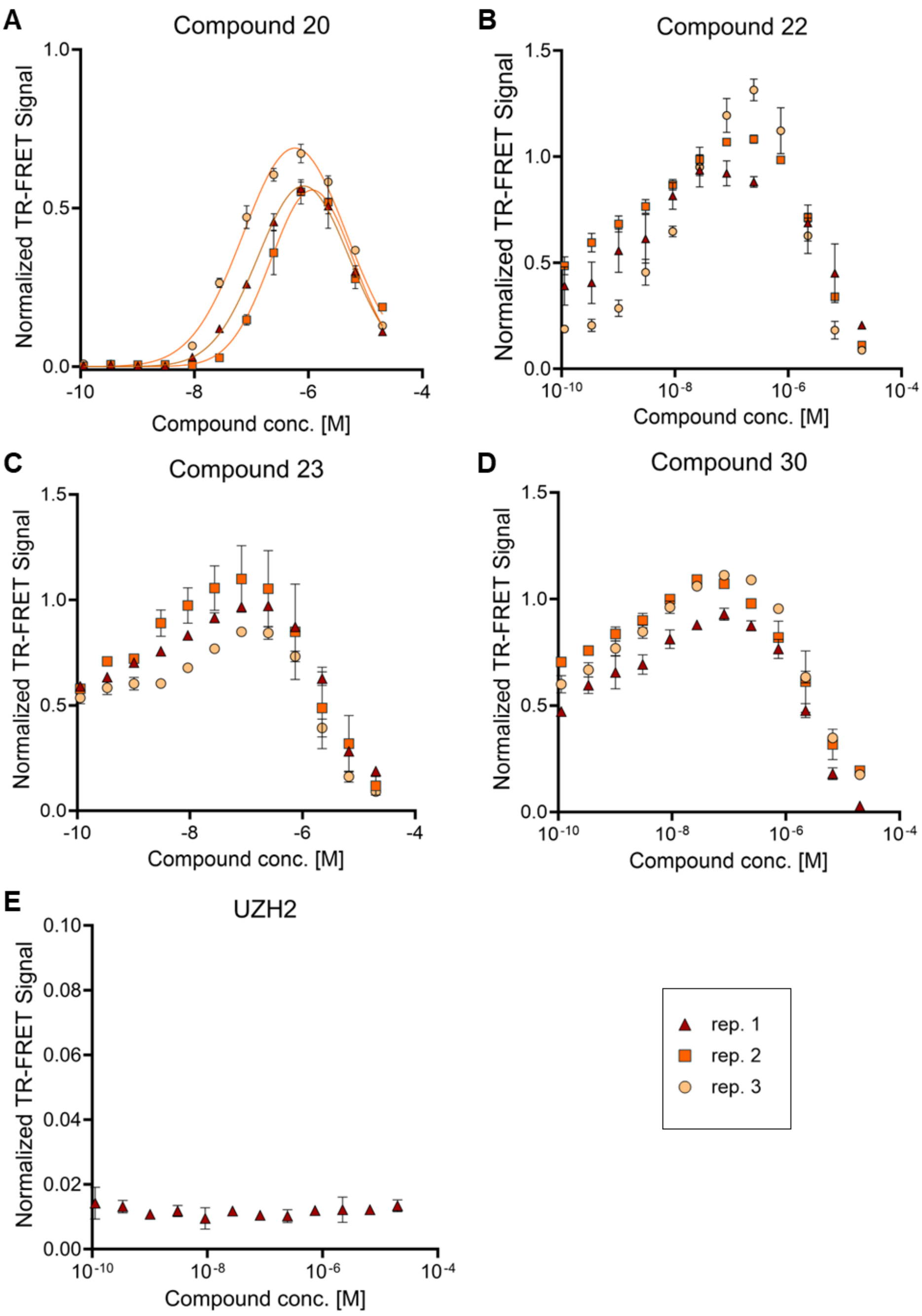

Figure S6. Dose-response curves derived from the TR-FRET ternary complex formation assay between METTL3-14, CRBN and 4 PROTACs (a-d) or UZH2 as negative control (e) (mean ± SD, n = 3 technical replicates). 3 biological replicates from independent measurements are shown for compounds **20**, **22**, **23** and **30** and 1 for UZH2. The Hook effect is apparent in all curves in panels a-d. The curve in panel a was fitted with the Gaussian function, which was not appropriate for the curves in panels b-d. EC_{max} and the amplitude of the curves were either derived from the fitting parameters or from the coordinates of the data point with the highest signal. UZH2 (e) showed no activity in the ternary complex formation assay since it is only able to bind to METTL3 and not to CRBN.

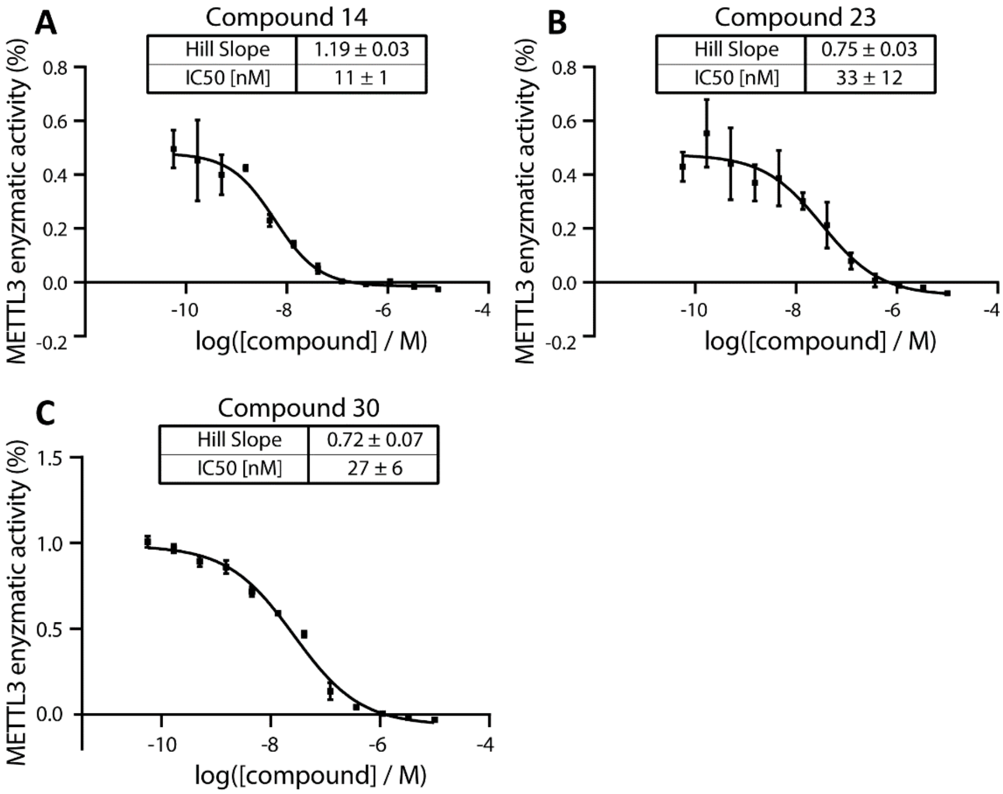

Figure S7. Dose-response curves derived from the reader-based TR-FRET inhibition assay on METTL3-14 (mean ± SD, n = 3 technical replicates) measured for three PROTACs: compound **14** (a), **23** (b), and **30** (c). IC_{50} and Hill Slope values were obtained from fits with nonlinear regression "log(inhibitor) vs. normalized response with variable slope". Note that depending on the placement of a compound on the 384-well plate used, the maximal signal can deviate from the expected 100% maximum enzymatic activity.

Supplementary Tables

Table S1. Protein methyltransferases selectivity profile of UZH2 at 10μM.

Methyltransferase	Remaining activity [%][a]		Control IC_{50} (M)	Control compound[b]
	Replicate 1	Replicate 2		
DOT1L	84	83	1.05E-07	SAH
G9a	85	88	7.08E-07	SAH
MLL4 Complex	88	87	8.16E-07	SAH
PRDM9	97	102	9.05E-07	Chaetocin
PRMT1	100	97	2.19E-07	SAH
SETD2	92	87	1.38E-06	SAH
SMYD3	84	90	8.48E-06	SAH
METTL3-14	0.02	0.02	1.53E-07	SAH

[a] The remaining activity is the percentage of enzymatic activity in the presence of 10 μM UZH2 concerning the buffer solution containing DMSO. The closer to 100% these values are, the weaker the inhibitory potency of UZH2.
[b] SAH = S-(5'-adenosyl)-L-homocysteine.

Table S2. Hook curve amplitudes derived from the TR-FRET ternary complex formation assay with all compounds in the paper. n = number of independent measurements (biological replicates).

Compound	TCFA amplitude (mean ± SEM)	n
**1**	0.03	1
**2**	0.3 ± 0.1	2
**3**	0.3 ± 0.2	2
**4**	0.4	1
**5**	0.7 ± 0.1	2
**6**	0.4	1
**7**	0.1	1
**8**	0.6	1
**9**	0.4	1
**10**	1.0 ± 0.0	3
**11**	0.3 ± 0.0	2
**12**	0.7 ± 0.1	2
**13**	0.8 ± 0.0	2
**14**	1.2 ± 0.0	3
**15**	0.8 ± 0.1	2
**16**	1.0 ± 0.0	13
**17**	1.0 ± 0.0	2
**18**	0.6 ± 0.1	3

Compound	TCFA amplitude (mean ± SEM)	n
**19**	0.9 ± 0.1	2
**20**	0.6 ± 0.0	3
**21**	1.3 ± 0.1	3
**22**	1.1 ± 0.1	3
**23**	1.0 ± 0.1	3
**24**	1.2 ± 0.0	2
**25**	1.2 ± 0.1	2
**26**	0.8 ± 0.0	3
**27**	0.7 ± 0.0	2
**28**	0.0 ± 0.0	2
**29**	1.0 ± 0.0	2
**30**	1.0 ± 0.1	3
**31**	0.9 ± 0.0	2
**32**	inactive	2
**33**	0.8 ± 0.1	2
**34**	0.2 ± 0.1	2
**35**	0.7 ± 0.2	2

Table S3. Cell viability data of PROTACs tested on MOLM-13 cells at different concentration.

	MOLM-13, cell viability (%) at 10-0.6 µM (average of two biological replicates)				
**Compound**	10 µM	5 µM	2.5 µM	1.25 µM	0.6 µM
**10**	86.6	n.d.	n.d.	n.d.	n.d.
**11**	84.6	n.d.	n.d.	n.d.	n.d.
**12**	94.3	n.d.	n.d.	n.d.	n.d.
**13**	n.d.	n.d.	n.d.	n.d.	n.d.
**14**	94.1	102.2	102.7	97.5	95.5
**15**	84.6	89.4	97.2	94.8	91.2
**16**	102.9	n.d.	n.d.	n.d.	n.d.
**17**	105.8	105.9	111.9	108.8	106.0
**18**	105.2	102.5	104.1	105.4	101.2
**19**	87.6	97.1	101.7	104.4	103.8
**20**	86.4	89.5	90.7	94.6	101.1
**21**	102.1	109.5	110.5	105.4	104.5
**22**	77.4	88.4	96.2	97.8	95.8
**23**	78.2	87.3	95.2	93.3	95.3
**24**	91.0	104.4	108.8	95.2	102.4
**25**	83.2	87.0	92.1	94.8	102.0
**26**	97.3	96.6	91.2	92.6	97.9
**27**	90.9	95.5	92.6	88.7	95.2
**28**	72.0	80.5	86.8	93.0	96.5
**29**	87.4	94.5	93.4	93.5	93.7
**30**	78.4	82.5	91.1	90.9	91.3
**31**	80.2	85.2	88.0	88.3	95.4
**32**	74.6	91.5	103.1	105.9	105.7
**33**	89.6	97.7	95.3	90.9	92.1
**34**	101.4	99.2	96.3	97.3	95.8
**35**	104.6	n.d.	n.d.	n.d.	n.d.
**UZH2**	64.1	84.3	91.0	96.6	101.4

Chemistry

Materials and Methods

All reagents were purchased from commercial suppliers and used as received. Reactions run at elevated temperatures were carried out in the oil bath. All reactions were monitored by thin-layer chromatography (Aluminium plates coated with silica gel 60 F_{254}). Flash column chromatography was carried out over silica gel (0.040-0.063 mm) or aluminium oxide (0.050-0.200 mm). SiliaMetS® TAAcONa (or SiliaMetS® Triaminetetraacetate, sodium salt) is a silica-bound metal scavenger for Pd(II), Ni(II) and Cu. It is the supported version of EDTA in its sodium salt form. ^{1}H and ^{13}C {^{1}H} NMR spectra were recorded on AV2-400 MHz and AV-600 Bruker spectrometers (400 MHz, 101 MHz and 600 MHz, 150 MHz, respectively) in DMSO-d_6, $CDCl_3$ or MeOD-d_4. Chemical shifts are given in ppm and their calibration was performed to the residual ^{1}H and ^{13}C signals of the deuterated solvents. Multiplicities are abbreviated as follows: singlet (s), doublet (d), triplet (t), multiplet (m), and broad signal (bs). The purity was acquired by Liquid chromatography high-resolution electrospray ionization mass spectrometry (LC-HR-ESI-MS): *Acquity UPLC* (*Waters*, Milford, USA) connected to an *Acquity* eλ diode array detector and a *Synapt G2* HR-ESI-QTOF-MS (*Waters*, Milford, USA); injection of 1 µL sample (c = ca. 10-100 µg/mL in the indicated solvent); *Acquity BEH C18* HPLC column (1.7 µm particle size, 2.1 × 50 mm, *Waters*) kept at 30°C; elution at a flow rate of 400 µL/min with A: H_2O + 0.02% TFA and B: CH_3CN + 0.02% TFA, linear gradient from 10–95% B within 3 min, then isocratic 95% B for 2 min; UV spectra recorded from 190–300 nm at 1.2 nm resolution and 20 points s^{-1}; ESI: positive ionization mode, capillary voltage 3.0 kV, sampling cone 40V, extraction cone 4V, N_2 cone gas 4 L/h, N_2 desolvation gas 800 L/min, source temperature 120°C; mass analyzer in resolution mode: mass range 100–2’000 *m/z* with a scan rate of 1 Hz; mass calibration to <2 ppm within 50–2’500 m/z with a 5mM aq. soln. of HCO_2Na, lockmasses: *m/z* 195.0882 (caffein, 0.7 ng/mL) and 556.2771 (Leucine-enkephalin, 2 ng/mL). The HPLC analyses were performed on a Shimadzu LC − 9A HPLC system equipped with a Shimadzu SPD − 6A VP UV − Vis detector; Phenomenex RP-HPLC on a Phenomenex InertClone (5 µm particle size, 4.6 mm × 150 mm i.d.). HPLC purifications were performed on a Shimadzu LC − 8A HPLC system equipped with a Shimadzu SCL − 10A VP System control and a Shimadzu SPD − 10A VP UV − Vis detector on a Phenomenex Gemini C18-110A preparative column (10-µm particle size, 250 mm × 21.2 mm i.d.).

Compounds purity

The final compounds **1-35**, **me-14** and **me-24** have a purity ≥ 95% assessed by HPLC, HPLC traces can be found further on in the Supplemental Information.

Experimental section

General procedure 1: SNAr from compound 39 (synthesis of compounds 40, 41, 42a-47a)

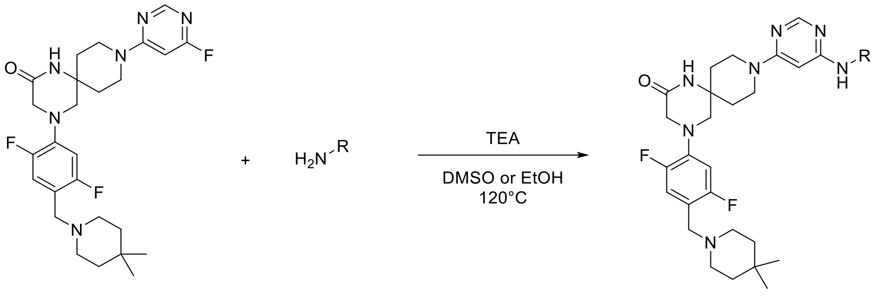

39

40, 41,
42a-47a

To a solution of compound **39** (1 eq) in DMSO (for **40, 41, 46a**) or EtOH (for **42a-45a, 47a**) (0.5 M) in a pressure vial were subsequently added amine (1.5 eq) and TEA (4 eq). The resulting reaction mixture was stirred at 120°C (oil bath temperature) until completion (Monitored by TLC).

General procedure 2: Boc-protected amines deprotection (synthesis of compounds 42-47, 49, 51)

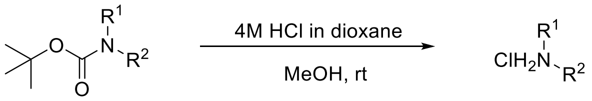

42a-47a,
49a, 51a

42-47,
49, 51

To a stirred solution of corresponding Boc-protected amine (1 eq) in MeOH (0.5 M) was added 4M HCl in dioxane (10 eq). The resulting reaction mixture was stirred at rt until full completion (Monitored by TLC). The volatiles were then removed *in vacuo* and the product was used in the following synthetic step without further purification.

General procedure 3: amide coupling (synthesis of compounds 49a, 1-20, 22-25, 29-31, 33, me-14, me-24)

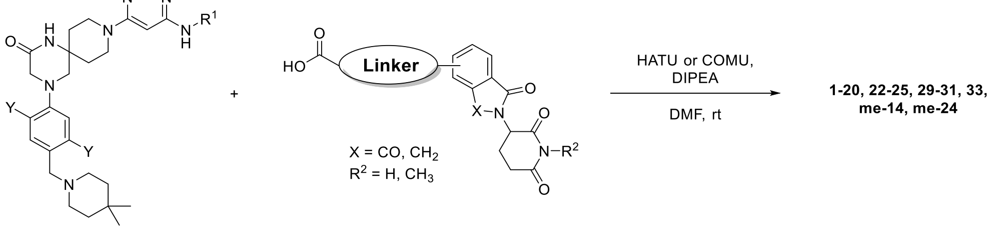

42-46, 48

S16-S30

To a cooled solution (water-ice bath) of carboxylic acid (1 eq) in DMF (0.5 M) was added DIPEA (4 eq), and the reaction mixture was stirred at the same temperature for 10 minutes. After the addition of 1.1 eq of HATU (for compounds **1-4, 8, 9, 29-31**) or COMU (for compounds **5-7, 10-28, 32-35**), the solution was stirred for an additional 30 minutes after which the amine (1 eq) was added. The resulting reaction mixture was stirred at rt until completion (Monitored by TLC), concentrated *in vacuo*, and purified using flash column chromatography.

General procedure 4 (synthesis of compounds S1-S4)

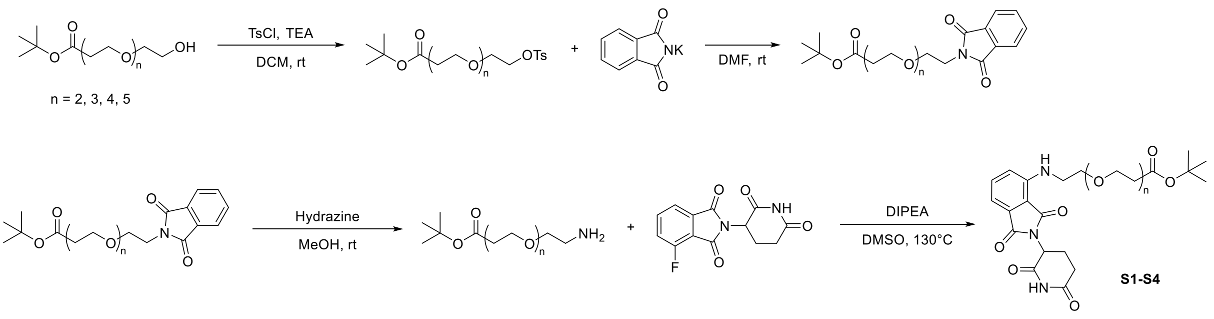

To a stirred solution of corresponding *tert*-butyl ester (1 eq) in DCM (0.5 M) was subsequently added TsCl (1.6 eq) and TEA (5 eq). The resulting reaction mixture was stirred at rt until full completion (Monitored by TLC). The volatiles were removed *in vacuo* and the product was involved in the next step without further purification.

To a stirred solution of corresponding tosylated *tert*-butyl ester in DMF (0.5 M), was added potassium phthalimide (2 eq). The resulting reaction mixture was stirred at rt until full completion (Monitored by TLC). The volatiles were then removed *in vacuo* and the product was used in the following step without further purification.

The compound was then dissolved in methanol (0.5 M), and hydrazine hydrate 50-60% (3 eq). The resulting reaction mixture was stirred at rt until full completion (Monitored by TLC). To the resulting mixture was added DCM and the formed precipitate was removed by filtration. The filtrate was concentrated *in vacuo* and the resulting product was used in the following step without any further purification.

To a stirred solution of 4-fluoro-thalidomide (1 eq) in DMSO (0.5 M) was subsequently added the corresponding *tert*-butyl ester (1 eq) and DIPEA (2 eq). The resulting reaction mixture was stirred at 130°C (oil bath temperature) until completion (Monitored by TLC). The volatiles were then removed *in vacuo* and the crude product was purified using flash column chromatography.

General procedure 5 (synthesis of compounds S5-S7)

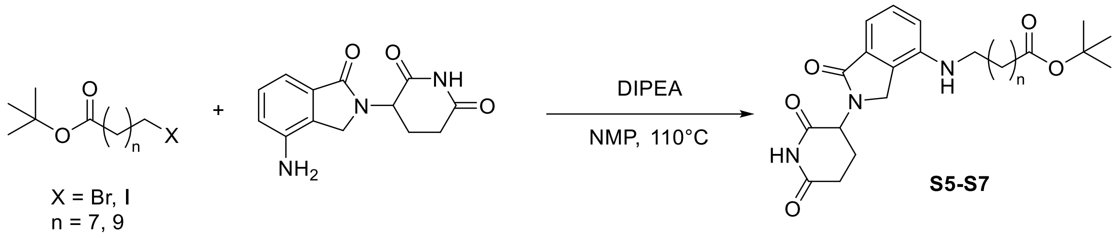

S5-S7

To a stirred solution of lenalidomide (1 eq) in NMP (0.5 M) were subsequently added the corresponding *tert*-butyl ester (1 eq) and DIPEA (10 eq). The resulting reaction mixture was stirred at 110°C (oil bath temperature) until full completion (Monitored by TLC). The volatiles were then removed *in vacuo* and the crude product was purified using flash column chromatography.

General procedure 6 (synthesis of compounds 56, 57, S8-S12)

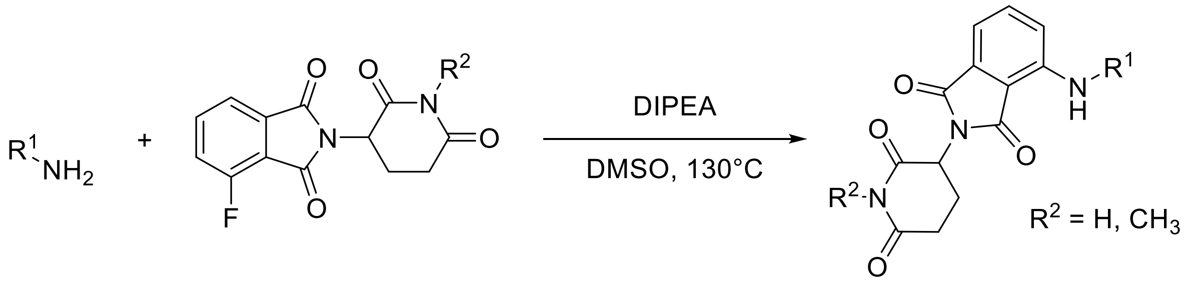

56, 57, S8-S12

To a stirred solution of 4-fluoro-thalidomide (1 eq) in DMSO (0.5 M) was subsequently added the corresponding amine (1 eq) and DIPEA (3 eq). The resulting reaction mixture was stirred at 130°C (if not stated otherwise) until full completion (Monitored by TLC). The volatiles were then removed *in vacuo* and the crude product was purified using flash column chromatography.

General procedure 7 (synthesis of compounds S13-S15)

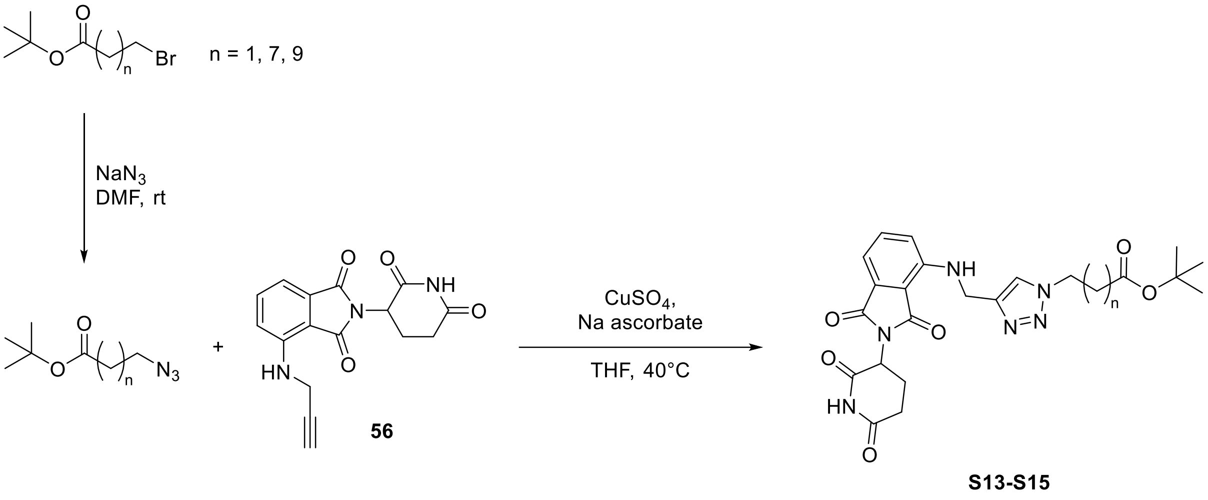

To a stirred solution of *tert*-butyl ester (1 eq) in DMF (0.5 M) was added sodium azide (1.2 eq). The resulting reaction mixture was stirred at rt until full completion (Monitored by ^{1}H NMR). The volatiles were then removed *in vacuo*, taken up with DCM, and filtered through filter paper. The product was involved in the next step without further purification.

Compound **56** (1 eq) was dissolved in THF (0.5 M) followed by the addition of the corresponding *tert*-butyl ester (1 eq), anhydrous $CuSO_4$ (0.5 eq), and sodium ascorbate (1.1 eq). The resulting reaction mixture was stirred at 40°C (oil bath temperature) until full completion (Monitored by TLC). The volatiles were then removed *in vacuo* and the crude product was purified using flash column chromatography.

General procedure 8 (synthesis of compounds 52 and 53)

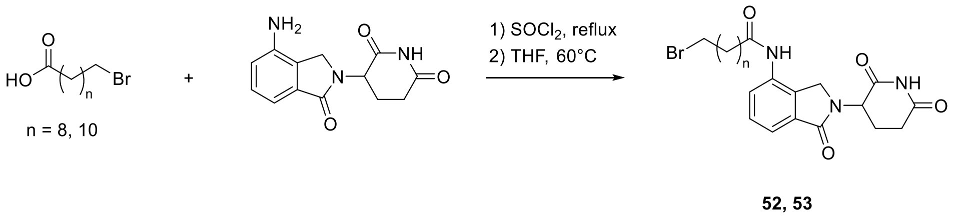

In a flame-dried round-bottomed flask equipped with a magnetic stirring bar and under N_2-atm, 200 mg of acid (1 eq) were dissolved in 0.5 mL of $SOCl_2$. The mixture was stirred at reflux for 5h and then the solvent was evaporated *in vacuo*. The residue was dissolved in 1 mL of dry THF and 1 eq of Lenalidomide was added to the flask. The mixture was stirred overnight at reflux under N_2-atm. Then the solvent was evaporated under reduced pressure and the residue was treated with water and extracted with EtOAc. The combined organic layers were washed

with water, dried ($MgSO_4$), filtered, and evaporated. The crude product was purified using flash column chromatography.

General procedure 9 (synthesis of compounds 54 and 55)

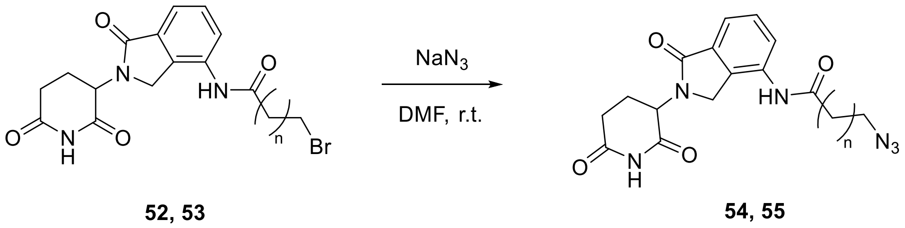

The Bromo compound (1 eq) was dissolved in DMF (0.5 M), to a stirred solution of it was added sodium azide (1.2 eq). The resulting reaction mixture was stirred at rt until completion (Monitored by ^{1}H-NMR). The reaction mixture was evaporated and extracted in EtOAc (3x). The combined organic layers were dried over $MgSO_4$, filtrated, and evaporated.

General procedure 10: *tert*-butyl esters deprotection (synthesis of compounds S16-S30)

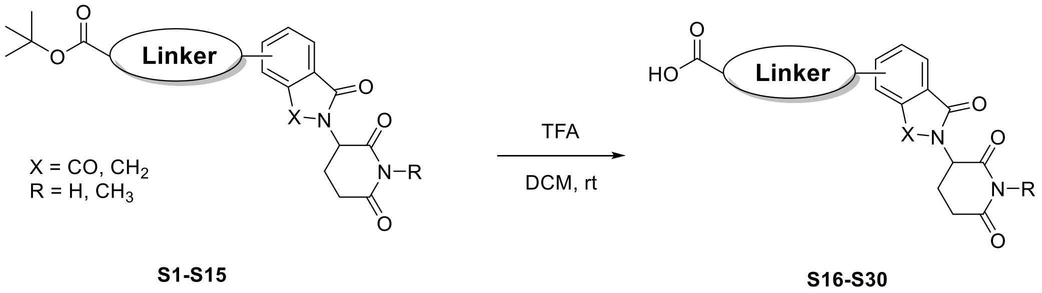

To a stirred solution of corresponding *tert*-butyl esters **S1-S15** (1 eq) in DCM (0.5 M) was added TFA (10 eq). The resulting reaction mixture was stirred at rt until full completion (Monitored by TLC). The volatiles were then removed *in vacuo* and the obtained products (**S16-S30**) were used in the following synthetic step without further purification.

Preparation of 4-(4-((4,4-dimethylpiperidin-1-yl)methyl)-2,5-difluorophenyl)- 9-(6-fluoropyrimidin-4-yl)-1,4,9-triazaspiro[5.5]undecan-2-one (39)

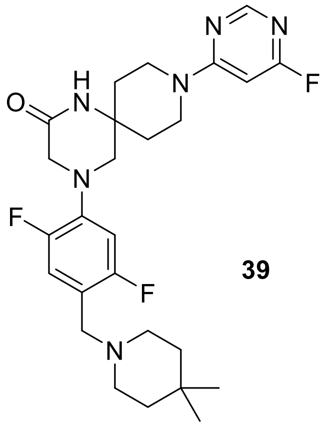

39

4-(4-((4,4-dimethylpiperidin-1-yl)methyl)-2,5-difluorophenyl)-1,4,9-triazaspiro[5.5]undecan-2-one **37** (Ref.[1]) (100 mg, 0.27 mmol) was dissolved in *i*PrOH (0.5 M). To the stirred solution were subsequently added 4,6-difluoro pyrimidine (27 µL, 0.32 mmol) and TEA (150 µL, 1.01 mmol). The resulting reaction mixture was stirred at 80°C (oil bath temperature) until full completion (Monitored by TLC). The volatiles were removed *in vacuo* and the crude product was purified using flash column chromatography (SiO_2; DCM/MeOH = 9 : 1) providing 112 mg of the desired product (89% yield). ^1H NMR (400 MHz, DMSO-d_6) δ 8.30 (d, *J* = 2.8 Hz, 1H), 8.22 (s, 1H), 7.15 (dd, *J* = 13.5, 6.6 Hz, 1H), 6.93 (dd, *J* = 11.5, 7.3 Hz, 1H), 6.58 (s, 1H), 4.00 (s, 2H), 3.60 (s, 2H), 3.58 – 3.47 (m, 2H), 3.42 (s, 2H), 3.28 (s, 2H), 2.33 (s, 3H), 1.83 (m, 2H), 1.68 (m, 2H), 1.30 (t, *J* = 5.3 Hz, 4H), 0.87 (s, 6H). ^{13}C NMR (101 MHz, DMSO-d_6) δ 172.5, 170.1, 167.1, 164.9, 164.8, 158.6, 158.4, 156.0, 152.0, 149.7, 107.1, 106.8, 85.9, 85.6, 55.4, 54.5, 53.3, 53.0, 49.5, 46.1, 38.7, 35.1, 28.6. LRMS (ESI): m/z 503.276 $[M + H]^+$, (calcd for $C_{26}H_{34}F_3N_6O^+$, 503.270).

Preparation of 9-(6-((3-aminopropyl)amino)pyrimidin-4-yl)-4-(4-((4,4-dimethyl piperidin-1-yl)methyl)phenyl)-1,4,9-triazaspiro[5.5]undecan-2-one (42)

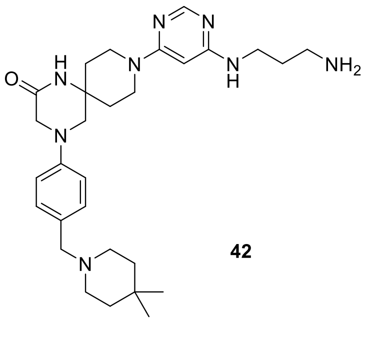

42

To a stirred solution of compound **38** (Ref.[1]) (100 mg, 0.21 mmol) in EtOH (0.5 M) were subsequently added *N*-Boc-1,3-propanediamine (108 mg, 0.62 mmol) and TEA (86.6 µL, 0.62 mmol). The resulting reaction mixture was refluxed until full completion (Monitored by TLC). The volatiles were removed *in vacuo* and the crude product was purified using flash column chromatography (SiO_2; DCM/MeOH = 9 : 1) providing117 mg of **42a** (91% yield).

The impure product (117 mg, 0.19 mmol) was dissolved in MeOH (0.5 M) followed by the addition of 37% HCl (57.7 µL, 1.9 mmol). The reaction mixture was stirred at rt until full completion (Monitored by TLC). The volatiles were removed *in vacuo* and the residue was extracted into *n*BuOH. The organic layer was washed with saturated aq. solution of Na_2CO_3. The combined organic layers were dried over $MgSO_4$, filtered, and concentrated providing 80 mg of the desired product **42** (81% yield). ^{1}H NMR (400 MHz, MeOD-*d₄*) δ 8.06 (d, *J* = 0.9 Hz, 1H), 7.38 (d, *J* = 8.2 Hz, 2H), 7.01 (d, *J* = 8.5 Hz, 2H), 5.77 (d, *J* = 1.0 Hz, 1H), 4.01 (s, 2H), 3.86 (s, 2H), 3.83 (s, 2H), 3.63 – 3.56 (m, 2H), 3.53 (s, 2H), 3.43 (t, *J* = 6.5 Hz, 2H), 3.17 (d, *J* = 0.8 Hz, 1H), 2.98 (t, *J* = 7.0 Hz, 6H), 1.95 – 1.87 (m, 4H), 1.81 (ddd, *J* = 13.3, 8.6, 4.1 Hz, 2H), 1.58 (d, *J* = 5.9 Hz, 4H), 1.29 (d, *J* = 2.7 Hz, 3H), 1.02 (s, 6H). ^{13}C NMR (101 MHz, DMSO-d_6) δ 167.6, 163.7, 162.1, 157.8, 148.6, 130.1, 129.2, 114.7, 65.4, 62.4, 53.3, 52.9, 51.9, 49.7, 38.8, 38.0, 37.9, 34.8, 29.6, 28.8, 28.6, 15.7. LRMS (ESI): m/z 521.371 $[M + H]^+$, (calcd for $C_{29}H_{44}N_8O^+$, 521.360).

Preparation of 4-(4-((4,4-dimethylpiperidin-1-yl)methyl)-2,5-difluorophenyl)-9-(6-(prop-2-yn-1-ylamino)pyrimidin-4-yl)-1,4,9-triazaspiro[5.5]undecan-2-one (40)

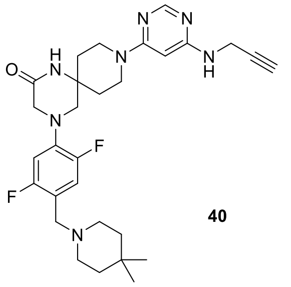

40

Compound **40** was prepared according to General procedure 1 using compound **39** (200 mg, 0.4 mmol) and propargylamine (76.46 µL, 3 eq) in DMSO. The reaction mixture was stirred at 100°C (oil bath temperature) for 14 h and then concentrated under reduced pressure. The crude product was purified using flash column chromatography (SiO_2; EtOAc/MeOH = 90 : 10) providing 150 mg of the desired product (70% yield). ^{1}H NMR (400 MHz, DMSO-d_6) δ 8.18 (s, 1H), 8.04 (s, 1H), 7.15 (dd, *J* = 13.0, 6.7 Hz, 1H), 6.98 (t, *J* = 6.0 Hz, 1H), 6.93 (dd, *J* = 11.7, 7.5 Hz, 1H), 5.71 (s, 1H), 4.02 (dd, *J* = 5.9, 2.5 Hz, 2H), 3.91 – 3.81 (m, 2H), 3.59 (s, 2H), 3.44

(s, 2H), 3.39 – 3.29 (m, 2H), 3.27 (s, 2H), 3.06 (t, *J* = 2.4 Hz, 1H), 2.35 (s, 4H), 1.83 – 1.73 (m, 2H), 1.72 – 1.61 (m, 2H), 1.31 (t, *J* = 5.5 Hz, 4H), 0.87 (s, 6H). ^{13}C NMR (101 MHz, DMSO-d_6) δ 166.5, 162.7, 161.6, 158.0, 157.3, 155.6, 151.5, 149.2, 138.1, 117.8, 106.6, 106.3, 81.9, 72.7, 54.8, 54.0, 52.8, 52.6, 49.1, 38.2, 34.6, 29.7, 28.1. LRMS (ESI): m/z 538.310 [M + H]$^+$, (calcd for $C_{29}H_{38}F_2N_7O^+$, 538.310).

Preparation of tert-butyl (3-((6-(4-(4-((4,4-dimethylpiperidin-1-yl)methyl)- 2,5-difluorophenyl)-2-oxo-1,4,9-triazaspiro[5.5]undecan-9-yl)pyrimidin-4-yl)amino)propyl)carbamate (43a)

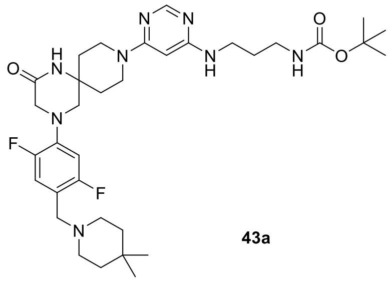

43a

Compound **43a** was prepared according to General procedure 1 using compound **39** (300 mg, 0.6 mmol) and *tert*-butyl (3-aminopropyl)carbamate (156 mg, 0.9 mmol) in EtOH. The volatiles were removed *in vacuo* and the crude product was purified using flash column chromatography (SiO_2; DCM/MeOH = 90 : 10) providing 280 mg of the desired product (71% yield). ^{1}H NMR (400 MHz, DMSO-d_6) δ 8.18 (s, 1H), 7.97 (s, 1H), 7.17 (s, 1H), 6.94 (dd, *J* = 11.5, 7.3 Hz, 1H), 6.82 (t, *J* = 5.7 Hz, 1H), 6.61 (t, *J* = 5.7 Hz, 1H), 5.60 (s, 1H), 3.86 (d, *J* = 13.5 Hz, 2H), 3.60 (s, 2H), 3.27 (s, 2H), 3.22 – 3.12 (m, 2H), 2.96 (q, *J* = 6.6 Hz, 2H), 2.37 (s, 4H), 1.77 (dt, *J* = 14.2, 4.2 Hz, 2H), 1.70 – 1.52 (m, 5H), 1.37 (s, 9H), 1.34 – 1.28 (m, 4H), 0.88 (s, 6H). ^{13}C NMR (101 MHz, DMSO-d_6) δ 166.5, 163.2, 161.6, 157.3, 155.7, 117.9, 106.5, 106.3, 77.5, 54.7, 52.8, 52.7, 49.0, 38.0, 37.7, 34.6, 29.5, 28.3, 28.1. LRMS (ESI): m/z 657.404 [M + H]$^+$, (calcd for $C_{34}H_{51}F_2N_8O_3^+$, 657.405).

Preparation of tert-butyl (3-(((6-(4-(4-((4,4-dimethylpiperidin-1-yl)methyl)- 2,5-difluorophenyl)-2-oxo-1,4,9-triazaspiro[5.5]undecan-9-yl)pyrimidin-4-yl)amino)methyl) benzyl)carbamate (44a)

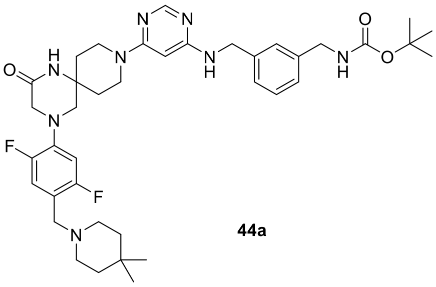

44a

Compound **44a** was prepared according to General procedure 1 using compound **39** (50 mg, 0.1 mmol) and 1-(*N*-Boc-aminomethyl)-3-(aminomethyl)benzene (35 mg, 0.15 mmol) in EtOH. The volatiles were removed *in vacuo* and the crude product was purified using flash column chromatography (SiO_2; DCM/MeOH = 90 : 10) providing 51 mg of the desired product (71% yield). ^{1}H NMR (400 MHz, $CDCl_3$) δ 8.16 (s, 1H), 7.30 (t, *J* = 7.5 Hz, 1H), 7.23 – 7.18 (m, 3H), 7.12 (dd, *J* = 12.8, 6.6 Hz, 1H), 6.60 – 6.56 (m, 2H), 5.43 (s , 1H), 5.20 (s, 1H), 4.89 (s, 1H), 4.53 (d, *J* = 5.8 Hz, 2H), 4.30 (d, *J* = 6.0 Hz, 2H), 3.73 – 3.66 (m, 4H), 3.54 (m, 2H), 3.50 (s, 2H), 3.27 (s, 2H), 2.41 (s, 3H), 1.93 (m, 2H), 1.77 (m, 2H), 1.44 (s, 8H), 1.40 (t, *J* = 7.4, 6.5 Hz, 4H), 0.90 (s, 6H). ^{13}C NMR (101 MHz, $CDCl_3$) δ 167.7, 163.2, 162.5, 157.9, 155.9, 139.7, 138.6, 129.1, 126.5, 126.2, 126.1, 105.8, 105.5, 81.3, 56.4, 54.7, 53.5, 53.0, 49.7, 45.7, 44.5, 40.4, 38.5, 35.2, 29.7, 28.4, 28.4. LRMS (ESI): m/z 719.422 $[M + H]^+$, (calcd for $C_{39}H_{53}F_2N_8O_3^+$, 719.420).

Preparation of tert-butyl 4-(((6-(4-(4-((4,4-dimethylpiperidin-1-yl)methyl)- 2,5-difluorophenyl)-2-oxo-1,4,9-triazaspiro[5.5]undecan-9-yl)pyrimidin-4-yl)amino)methyl)piperidine-1-carboxylate (45a)

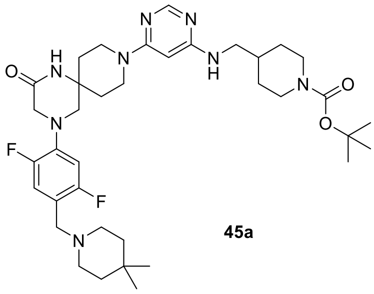

45a

Compound **45a** was prepared according to General procedure 1 using compound **39** (50 mg, 0.1 mmol) and *tert*-butyl 4-(aminomethyl)piperidine-1-carboxylate (42 mg, 0.15 mmol) in EtOH. The volatiles were removed *in vacuo* and the crude product was purified using flash

column chromatography (SiO_2; DCM/MeOH = 90 : 10) providing 55 mg of the desired product (79% yield). ^1H NMR (400 MHz, $CDCl_3$) δ 8.14 (s, 1H), 7.11 (dd, *J* = 12.9, 6.6 Hz, 1H), 6.67 (m , 1H), 6.58 (dd, *J* = 10.9, 7.1 Hz, 1H), 5.43 (s, 1H), 4.87 (s, 1H), 4.13 (s, 2H), 3.78 – 3.68 (m, 4H), 3.60 (m, 2H), 3.48 (s, 2H), 3.28 (s, 2H), 3.14 (t, *J* = 6.1 Hz, 2H), 2.70 (t, *J* = 12.7 Hz, 2H), 2.44 – 2.37 (m, 4H), 1.97 (m, 2H), 1.82 (m, 2H), 1.77 – 1.69 (m, 3H), 1.45 (s, 9H), 1.39 (t, *J* = 5.6 Hz, 4H), 1.28 – 1.11 (m, 2H), 0.91 (s, 6H).^{13}C NMR (101 MHz, $CDCl_3$) δ 167.8, 163.4, 162.4, 157.8, 156.0, 154.8, 152.2, 137.3, 118.4, 118.2, 105.9, 105.6, 80.7, 79.5, 56.5, 54.7, 53.4, 53.0, 49.8, 47.2, 40.2, 38.6, 36.4, 35.4, 30.0, 28.5, 28.4. LRMS (ESI): m/z 697.438 $[M + H]^+$, (calcd for $C_{37}H_{55}F_2N_8O_3^+$, 697.440).

Preparation of tert-butyl (3-(((6-(4-(4-((4,4-dimethylpiperidin-1-yl)methyl)- 2,5-difluorophenyl)-2-oxo-1,4,9-triazaspiro[5.5]undecan-9-yl)pyrimidin-4-yl)4-(2-aminoethyl)piperazin)carbamate (46a)

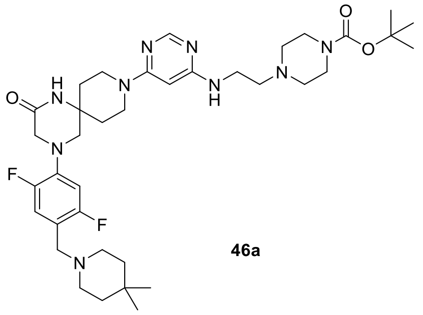

46a

Compound **46a** was prepared according to General procedure 1 using compound **39** (50 mg, 0.1 mmol) and *tert*-butyl 4-(2-aminoethyl)piperazine-1-carboxylate (34 mg, 0.15 mmol) in DMSO. After reaction completion, the volatiles were removed *in vacuo* and the crude mixture was purified using flash column chromatography (SiO_2; DCM/MeOH = 90 : 10) providing 91 mg of the desired product (86% yield). ^1H NMR (400 MHz, $CDCl_3$) δ 8.16 (s, 1H), 7.26 (s, 1H), 7.14 (dd, *J* = 12.7, 6.5 Hz, 1H), 6.78 (s, 1H), 6.58 (dd, *J* = 10.8, 7.2 Hz, 1H), 5.45 (s, 1H), 5.36 (s, 1H), 3.77–3.68 (m, 4H), 3.67-3.58 (m, 2H), 3.53 (s, 2H), 3.44 (t, *J* = 4.6 Hz, 4H), 3.35-3.24 (m, 4H), 2.51-2.37 (m, 8H), 1.96 (m, 2H), 1.84 (m, 2H), 1.77 (m, 2H), 1.46 (s, 9H), 1.41 (t, *J* = 5.3 Hz, 4H), 0.92 (s, 6H). ^{13}C NMR (125 MHz, $CDCl_3$) δ 172.6, 166.8, 166.1, 162.5, 161.0, 160.5, 159.0, 155.7, 153.8, 142.2, 142.1, 122.9, 122.7, 84.1, 60.3, 59.3, 58.1, 57.2, 56.5, 56.4, 53.2, 44.4, 43.7, 41.8, 41.7, 38.7, 31.9. LRMS (ESI): m/z 712.902 $[M + H]^+$, (calcd for $C_{37}H_{56}F_2N_9O_3^+$, 712.447).

Preparation of tert-butyl 4-(4-(((6-(4-(4-((4,4-dimethylpiperidin-1-yl)methyl)-2,5-difluorophenyl)-2-oxo-1,4,9-triazaspiro[5.5]undecan-9-yl)pyrimidin-4-yl)amino)methyl)phenyl)piperazine-1-carboxylate (47a)

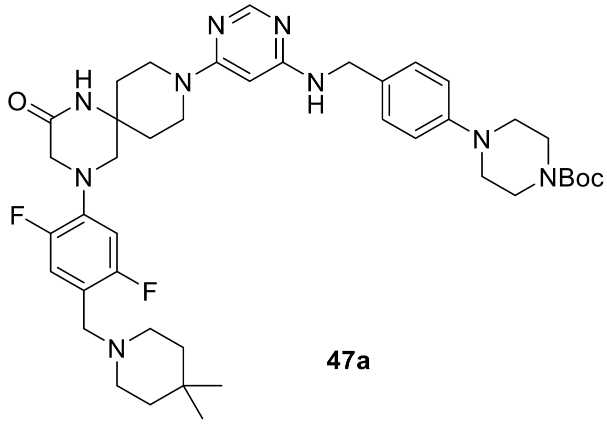

47a

Compound **47a** was prepared according to General procedure 1 using compound **39** (200 mg, 0.397 mmol) and *tert*-butyl 4-(4-(aminomethyl)phenyl)piperazine-1-carboxylate (115 mg, 0.397 mmol) in EtOH. The volatiles were removed *in vacuo* and the crude product was purified using flash column chromatography (SiO_2; DCM/MeOH = 90 : 10) providing 181 mg of the desired product (58% yield). ^1H NMR (400 MHz, $CDCl_3$) δ 8.18 (s, 1H), 7.23 (d, *J* = 8.6 Hz, 1H), 7.12 (dd, *J* = 12.9, 6.6 Hz, 1H), 6.92 – 6.88 (m, 2H), 6.58 (dd, *J* = 10.8, 7.2 Hz, 1H), 6.42 – 6.28 (m, 1H), 5.46 (s, 1H), 5.07 – 4.98 (m, 1H), 4.36 (d, *J* = 5.6 Hz, 1H), 3.74 – 3.65 (m, 4H), 3.61 – 3.53 (m, 6H), 3.49 (s, 2H), 3.27 (s, 2H), 3.15 – 3.09 (m, 4H), 2.43 – 2.37 (m, 4H), 1.99 – 1.91 (m, 2H), 1.83 – 1.75 (m, 2H), 1.73 – 1.62 (m, 4H), 1.48 (s, 9H), 1.39 (t, *J* = 5.6 Hz, 2H), 0.91 (s, 6H), 0.87 – 0.76 (m, 2H). ^{13}C NMR (151 MHz, $CDCl_3$) δ 171.2, 168.4, 167.4, 166.9, 162.4, 150.8, 150.2, 150.1, 135.9, 134.3, 129.0, 128.5, 123.5, 116.8, 116.2, 105.6, 81.0, 56.4, 53.6, 52.9, 51.2, 49.6, 49.3, 45.5, 41.1, 40.4, 35.4, 32.1, 31.6, 29.9, 29.8, 29.5, 28.2, 27.4, 22.9, 14.3, 1.2. LRMS (ESI): m/z 774.462 $[M + H]^+$, (calcd for $C_{42}H_{58}F_2N_9O_3^+$, 774.462).

Preparation of 4-(4-((4,4-dimethylpiperidin-1-yl)methyl)-2,5-difluorophenyl)-9-(6-((2-(4-(4-(piperazin-1-yl)phenyl)piperazin-1-yl)ethyl)amino)pyrimidin-4-yl)-1,4,9-triazaspiro[5.5]undecan-2-one (48a)

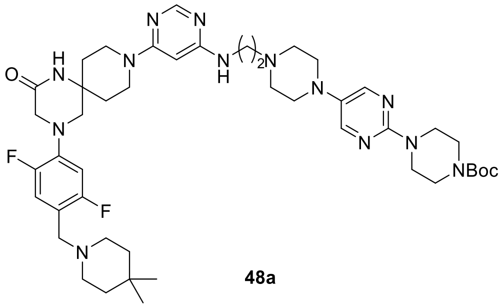

48a

To a stirred solution of *tert*-butyl 4-(5-bromopyrimidin-2-yl)piperazine-1-carboxylate (170 mg, 0.5 mmol) in DMSO (0.5 M) was subsequently added CuI (19 mg,0.1 mmol), L-proline (23 mg, 0.2 mmol), and K_2CO_3 (276 mg, 2 mmol). After a few minutes, compound **46** (376 mg, 0.6 mmol) was added. The resulting reaction mixture was stirred at 85°C until completion (Monitored by TLC). The reaction was cooled down to rt, quenched with water and extracted into DCM. The combined organic layers were washed twice with water, once with brine and dried over Na_2SO_4. The volatiles were then removed *in vacuo* and the crude product was purified using flash column chromatography (SiO_2; DCM/MeOH = 90 : 10) affording 43 mg of the desired product (10% yield). ^{1}H NMR (500 MHz, $CDCl_3$) δ 8.13 (s, 3H), 7.19 (m, 1H), 7.08 (m, 1H), 6.99 (dd, *J* = 10.3, 7.4 Hz, 1H), 5.46 (m, 2H), 3.73 (m, 9H), 3.64 (m, 3H), 3.59-3.47 (m, 9H), 3.35 (s, 2H), 3.30 (s, 2H), 3.07 (s, 3H), 3.00 (s, 3H), 2.67 (m, 4H), 2.63 (s, 4H), 2.48 (s, 4H), 1.96 (m, 2H), 1.86 (m, 2H), 1.50 (s, 9H), 1.44 (s, 2H), 0.93 (s, 6H). ^{13}C NMR (101 MHz, DMSO-d_6) δ170.1, 167.0, 164.8, 158.4, 157.8, 156.0, 154.9, 152.0, 149.6, 148.1, 138.4, 137.1, 118.3, 118.1, 107.7, 106.7, 80.2, 79.8, 57.2, 55.4, 54.4, 53.3, 52.7, 52.9, 50.4, 49.5, 46.1, 44.5, 38.6, 37.0, 35.1, 28.5, 28.2. LRMS (ESI): m/z 874.652 [M + H]$^+$, (calcd for $C_{45}H_{65}F_2N_{13}O_3^+$, 874.533).

Preparation of tert-butyl (2-(4-(((6-(4-(4-((4,4-dimethylpiperidin-1-yl)methyl)-2,5-difluorophenyl)-2-oxo-1,4,9-triazaspiro[5.5]undecan-9-yl)pyrimidin-4-yl)amino)methyl)piperidin-1-yl)-2-oxoethyl)carbamate (49a)

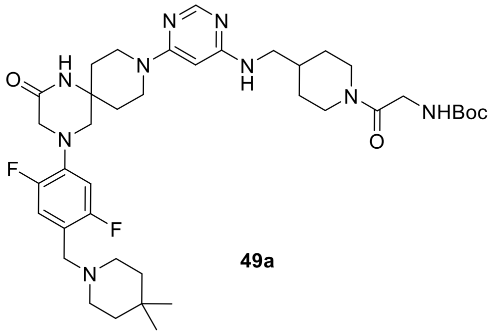

49a

Compound **49a** was prepared according to General procedure 3 (COMU) from compound **45** (108 mg, 0.17 mmol) and *N*-Boc-glycine (30 mg, 0.17 mmol). After reaction completion, the mixture was concentrated *in vacuo* and the crude product was purified using flash column chromatography (SiO_2; DCM/MeOH = 95:5) providing 75 mg of the desired product (58% yield). ^{1}H NMR (400 MHz, MeOD-*d4*) δ 7.99 (d, J = 0.8 Hz, 1H), 7.26 (dd, J = 12.9, 6.7 Hz, 1H), 6.93 (dd, J = 11.3, 7.3 Hz, 1H), 5.72 – 5.68 (m, 1H), 4.49 (d, J = 13.2 Hz, 1H), 3.95 – 3.82 (m, 6H), 3.75 (s, 2H), 3.41 (s, 3H), 3.22 – 3.15 (m, 2H), 3.06 (t, J = 12.9 Hz, 1H), 2.79 (s, 4H), 2.71 – 2.61 (m, 1H), 2.03 – 1.92 (m, 2H), 1.89 – 1.76 (m, 5H), 1.52 (t, J = 5.8 Hz, 4H), 1.45 (s, 9H), 1.32 – 1.09 (m, 4H), 0.98 (s, 6H). ^{13}C NMR (101 MHz, MeOD-*d4*) δ 168.7, 167.9, 163.4, 162.0, 157.0, 156.9, 156.7, 119.1, 118.8, 106.1, 105.8, 79.2, 54.6, 54.4, 53.4, 53.2, 52.2, 49.0, 48.45, 45.9, 44.4, 42.4, 42.0, 41.6, 40.3, 36.8, 36.1, 34.4, 29.9, 29.3, 27.5, 27.3, 11.8. LRMS (ESI): m/z 754.458 $[M + H]^+$, (calcd for $C_{39}H_{58}F_2N_9O_4^+$, 754.457).

Preparation of 9-(6-(((1-(2-chloroacetyl)piperidin-4-yl)methyl)amino)pyrimidin-4-yl)-4-(4-((4,4-dimethylpiperidin-1-yl)methyl)-2,5-difluorophenyl)-1,4,9-triazaspiro[5.5]undecan-2-one (50)

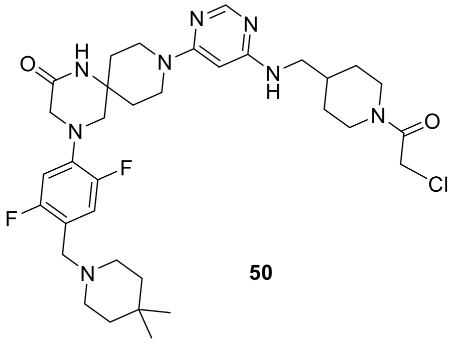

50

In a flame-dried flask under N_2-atmosphere, 150 mg of **45** (0.237 mmol, 1 eq) were suspended in 1 mL of dry-THF with 82 µL of DIPEA (0.47 mmol, 2 eq). 19 µL of 2-chloroacetyl chloride (0.237 mmol, 1 eq) were added dropwise at 0°C and the reaction was stirred at room temperature under N_2-atmosphere overnight. The mixture was then dried under reduced pressure and purified using flash column chromatography (SiO_2; DMC/MeOH = 90 : 10), affording 140 mg of impure product that was directly used for the following step. LRMS (ESI): m/z 673.356 $[M + H]^+$, (calcd for $C_{34}H_{48}ClF_2N_8O_2^+$, 673.355).

Preparation of tert-butyl 3-(2-(2-((2-(2,6-dioxopiperidin-3-yl)- 1,3-dioxoisoindolin-4-yl) amino)ethoxy)ethoxy)propanoate (S1)

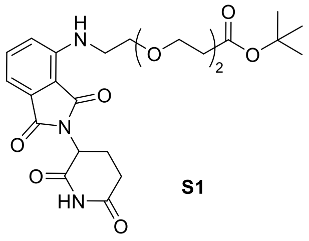

S1

Compound **S1** was prepared according to General procedure 4 using 4-fluoro-thalidomide (82 mg, 0.3 mmol) and the corresponding *tert*-butyl ester (70 mg, 0.3 mmol). The volatiles were then removed *in vacuo* and the crude product was purified using flash column chromatography (SiO_2; EtOAc/Hept = from 80 : 20 to 90 : 10) providing 64 mg of desired product (57% yield). The analytical results were consistent with data reported in the literature [(2)]. ^1H NMR (400 MHz, $CDCl_3$) δ 8.1 (s, 1H), 7.52 (m, *J* = 8.4, 7.1 Hz, 1H), 7.12 (d, *J* = 7.1 Hz, 1H), 6.97 (d, *J* = 8.5 Hz, 1H), 6.47 (t, *J* = 5.6 Hz, 1H), 4.85 (dd, *J* = 12.4, 5.3 Hz, 1H), 3.74 (m, 4H), 3.65 (m, 4H), 3.46 (m, 2H), 2.89 (m, 1H),2.86 (m, 2H), 2.53 (t, *J* = 6.6 Hz, 2H), 2.18 – 2.10 (m, 1H), 1.40 (s, 9H).

Preparation of tert-butyl 3-(2-(2-(2-((2-(2,6-dioxopiperidin-3-yl)- 1,3-dioxoisoindolin-4-yl) amino)ethoxy)ethoxy)ethoxy)propanoate (S2)

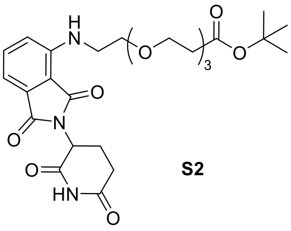

S2

Compound **S2** was prepared according to General procedure 4 using 4-fluoro-thalidomide (68 mg, 0.25 mmol) and the corresponding *tert*-butyl ester (69 mg, 0.25 mmol). The volatiles were then removed *in vacuo and* the crude product was purified using flash column chromatography (SiO_2; EtOAc/Hept = from 80 : 20 to 90 : 10) providing 52 mg of desired product (39% yield). The analytical results were consistent with data reported in the literature [(3)]. ^{1}H NMR (400 MHz, $CDCl_3$) δ 8.12 (s, 1H), 7.55 (t, *J* = 7.6 Hz, 1H), 7.13 (d, *J* = 6.8 Hz, 1H), 6.94 (d, *J* =8.3 Hz, 1H), 6.51 (s, 1H), 4.94 (m, 1H), 3.73 (m, 13H), 3.50 (m, 2H), 2.92 – 2.70 (m, 3H), 2.50 (t, *J* = 6.2 Hz, 2H), 2.15 (m, 1H), 1.44 (s, 9H).

Preparation of tert-butyl 1-((2-(2,6-dioxopiperidin-3-yl)- 1,3-dioxoisoindolin -4-yl)amino)-3,6,9,12-tetraoxapentadecan-15-oate (S3)

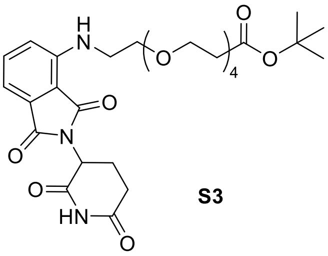

S3

Compound **S3** was prepared according to General procedure 4 using 4-fluoro-thalidomide (53 mg, 0.2 mmol) and the corresponding *tert*-butyl ester (62 mg, 0.2 mmol). The volatiles were then removed *in vacuo* and the crude product was purified using flash column chromatography (SiO_2; EtOAc/Hept = from 80 : 20 to 90 : 10) affording 64 mg of desired product (57% yield). The analytical results were consistent with data reported in the literature [(3)]. ^{1}H NMR (400 MHz, $CDCl_3$) δ 8.11 (s, 1H), 7.44 (dd, *J* = 7.32, 8.34 Hz, 1H),7.04 (d, *J* = 7.11 Hz, 1H), 6.83 (d, *J* = 8.54 Hz, 1H), 6.42 (m, 1H), 4.8 (s, 1H), 3.58 (m, 18H), 3.42 – 3.35 (m, 2H), 2.72 (s, 3H), 2.45 – 2.39 (m, 2H), 1.98 (m, 1 H), 1.43 (m, 9H).

Preparation of tert-butyl 1-((2-(2,6-dioxopiperidin-3-yl)- 1,3-dioxoisoindolin-4-yl) amino)-3,6,9,12,15-pentaoxaoctadecan-18-oate (S4)

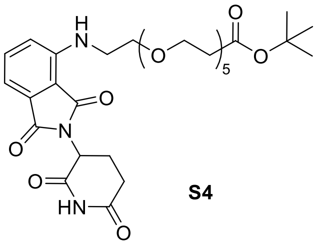

S4

Compound **S4** was prepared according to General procedure 4 using 4-fluoro-thalidomide (56 mg, 0.2 mmol) and the corresponding *tert*-butyl ester (74 mg, 0.2 mmol). The volatiles were then removed *in vacuo* and the crude product was purified using flash column chromatography (SiO_2; EtOAc/Hept = from 80 : 20 to 90 : 10) affording 61 mg of desired product (49% yield). The analytical results were consistent with data reported in the literature (3). ^{1}H NMR (400 MHz, $CDCl_3$) δ 8.19 (s, 1H), 7.46 (t, J = 7.7 Hz, 1H), 7.13 (d, J = 7.0 Hz, 1H), 6.90 (d, J = 8.4 Hz, 1H), 6.50 (m, 1H), 4.92 (m, 1H), 3.76 – 3.58 (m, 18H), 3.46 (m, 2H), 2.94 – 2.73 (m, 3H), 2.52 (t, J = 6.4 Hz, 2H), 2.14 (m, 1H), 1.43 (m, 9H).

Preparation of tert-butyl 9-((2-(2,6-dioxopiperidin-3-yl)-1-oxoisoindolin-4-yl) amino) nonanoate (S5)

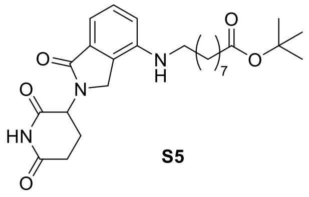

S5

Compound **S5** was prepared according to General procedure 5 using lenalidomide (267 mg, 1.03 mmol) and the corresponding *tert*-butyl ester (303 mg,1.03 mmol). The volatiles were then removed *in vacuo* and the crude product was purified using flash column chromatography (SiO_2; EtOAc/Hept = 70 : 30) providing 200 mg of the desired product (41% yield). ^{1}H NMR (400 MHz, DMSO-d_6) δ 11.00 (s, 1H), 7.27 (t, J = 7.7 Hz, 1H), 6.92 (d, J = 7.3 Hz, 1H), 6.73 (d, J = 8.0 Hz, 1H), 5.55 (t, J = 5.5 Hz, 1H), 5.10 (dd, J = 13.3, 5.1 Hz, 1H), 4.26 – 4.08 (m, 2H), 3.10 (q, J = 6.6 Hz, 2H), 2.92 (ddd, J = 18.3, 13.6, 5.4 Hz, 1H), 2.73 – 2.57 (m, 2H), 2.36 – 2.22 (m, 2H), 2.16 (td, J = 7.3, 3.7 Hz, 2H), 2.08 – 1.97 (m, 1H), 1.56 (m, 2H), 1.47 (m, 2H), 1.38 (s, 9H), 1.25 (m, 8H). ^{13}C NMR (101 MHz, $CDCl_3$) δ 173.9, 172.2, 170.2, 166.3, 147.5, 131.6, 129.8, 125.72, 116.7, 116.2, 80.3, 55.4, 48.2, 43.6, 35.2, 31.1, 29.3, 28.4, 27.7, 27.2, 26.4, 24.3. LRMS (ESI): m/z 416.26 $[M + H]^+$, (calcd for $C_{22}H_{30}N_3O_5^+$, 416.22).

Preparation of tert-butyl 11-((2-(2,6-dioxopiperidin-3-yl)- 1-oxoisoindolin-4-yl) amino)undecanoate (S6)

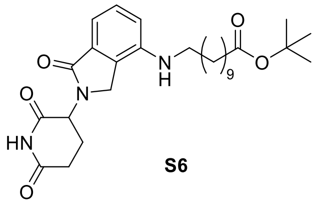

S6

Compound **S6** was prepared according to General procedure 5 using lenalidomide (245 mg, 0.95 mmol) and the corresponding *tert*-butyl ester (304 mg, 0.95 mmol). The volatiles were then removed *in vacuo* and the crude product was purified using flash column chromatography (SiO_2; EtOAc/Hept = 70 : 30) providing 125 mg of the desired product (26% yield). ^{1}H NMR (400 MHz, DMSO-d_6) δ 11.00 (s, 1H), 7.27 (t, *J* = 7.7 Hz, 1H), 6.95 – 6.88 (m, 1H), 6.73 (d, *J* = 8.0 Hz, 1H), 5.55 (t, *J* = 5.5 Hz, 1H), 5.10 (dd, *J* = 13.2, 5.1 Hz, 1H), 4.28 – 4.07 (m, 2H), 3.10 (q, *J* = 6.6 Hz, 2H), 2.92 (ddd, *J* = 18.0, 13.5, 5.3 Hz, 1H), 2.72 – 2.58 (m, 2H), 2.36 – 2.23 (m, 2H), 2.16 (t, *J* = 7.3 Hz, 2H), 2.07 – 1.98 (m, 1H), 1.57 (m, 2H), 1.46 (m, 2H), 1.38 (s, 9H), 1.24 (m, 14H). ^{13}C NMR (101 MHz, $CDCl_3$) δ 173.7, 172.5, 170.3, 165.9, 148.1, 131.7, 130.1, 125.6, 117.2, 116.4, 80.5, 56.3, 49.1, 43.6, 35.4, 31.3, 30.8, 29.7, 29.1, 28.2, 27.7, 27.3, 26.7, 23.3. LRMS (ESI): m/z 500.34 $[M + H]^+$, (calcd for $C_{28}H_{42}N_3O_5^+$, 500.31).

Preparation of tert-butyl 15-((2-(2,6-dioxopiperidin-3-yl)- 1-oxoisoindolin-4-yl) amino)pentadecanoate (S7)

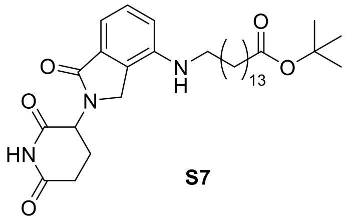

S7

Compound **S7** was prepared according to General procedure 5 using lenalidomide (264 mg, 1.02 mmol) and the corresponding *tert*-butyl ester (432 mg, 1.02 mmol). The volatiles were then removed *in vacuo* and the crude product was purified using flash column chromatography (SiO_2; EtOAc/Hept = 70 : 30) affording 125 mg of the desired product (22% yield). ^{1}H NMR (400 MHz, $CDCl_3$) δ 7.98 (s, 1H), 7.36 (t, *J* = 7.7 Hz, 1H), 6.80 (dd, *J* = 8.0, 0.9 Hz, 1H), 5.25 (dd, *J* = 13.2, 5.2 Hz, 1H), 4.36 – 4.09 (m, 2H), 4.05 (t, *J* = 6.8 Hz, 1H), 3.20 (t, *J* = 7.3 Hz, 2H), 2.92 – 2.78 (m, 2H), 2.36 – 2.26 (m, 2H), 2.20 (t, *J* = 7.5 Hz, 2H), 1.69 – 1.53 (m, 6H), 1.44 (s, 9H), 1.31 – 1.22 (m, 18H). ^{13}C NMR (101 MHz, DMSO-d_6) δ 174.4, 173.1, 170.5,

165.6, 149.7, 131.2, 129.5, 126.2, 117.1, 116.3, 79.8, 56.1, 48.4, 43.8, 36.7, 33.4, 30.7, 29.5, 29.5, 29.2, 28.4, 28.1, 27.7, 27.2, 26.9, 26.7, 25.5, 24.8. LRMS (ESI): m/z 556.41 $[M + H]^+$, (calcd for $C_{32}H_{50}N_3O_5^+$, 556.38).

Preparation of 2-(2,6-dioxopiperidin-3-yl)-4-(prop-2-yn-1-ylamino) isoindoline-1,3-dione (56)

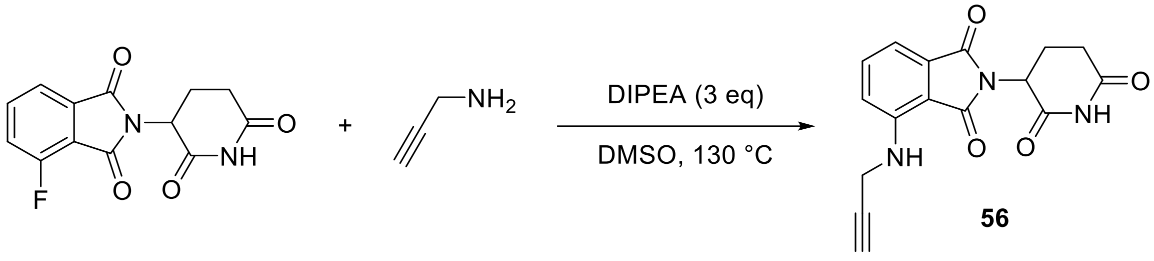

56

Compound **56** was prepared according to General procedure 6 using 4-fluoro-thalidomide (320 mg, 1.16 mmol) and propargylamine (64 mg, 1.16 mmol). The resulting reaction mixture was stirred at 130°C (oil bath temperature) until full completion (Monitored by TLC). The reaction mixture was quenched by the addition of a saturated aq. solution of $NaHCO_3$ and extracted into EtOAc (3 x 15ml). The combined organic layers were dried over $MgSO_4$, filtrated, and evaporated providing 280 mg of desired product **56** (78% yield). ^{1}H NMR (400 MHz, $CDCl_3$) δ 8.00 (s, 1H), 7.57 (dd, J = 8.5, 7.1 Hz, 1H), 7.20 (d, J = 7.1 Hz, 1H), 7.03 (d, J = 8.6 Hz, 1H), 6.44 (t, J = 6.5 Hz, 1H), 4.92 (dd, J = 12.2, 5.4 Hz, 1H), 4.09 (dd, J = 6.1, 2.5 Hz, 2H), 2.95 – 2.67 (m, 3H), 2.27 (t, J = 2.4 Hz, 1H), 2.20 – 2.07 (m, 1H). ^{13}C NMR (101 MHz, $CDCl_3$) δ 170.8, 169.2, 168.1, 167.4, 145.6, 136.2, 132.4, 117.2, 112.8, 111.4, 79.1, 72.2, 49.0, 32.3, 31.4, 22.8. LRMS (ESI): m/z 312.099 $[M + H]^+$, (calcd for $C_{16}H_{14}N_3O_4^+$, 312.090).

Preparation of tert-Butyl 4-(2-(2,6-dioxopiperidin-3-yl)-1,3-dioxoisoindolin-4-yl) -(2-(piperazin-1-yl)ethyl)carbamate (57)

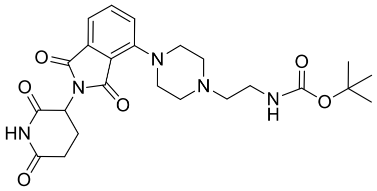

Compound **57** was prepared according to General procedure 6 using 4-fluoro-2-(1-methyl-2,6-dioxopiperidin-3-yl)isoindoline-1,3-dione (150 mg, 0.55 mmol) and *tert*-butyl (2-(piperazin-1-yl)ethyl)carbamate (138 mg, 0.60 mmol). The reaction was heated to 95°C until completion, as determined by TLC. After cooling to room temperature, the mixture was partitioned twice between H_2O and DCM; the combined organic layers were dried over Na_2SO_4, filtered and

evaporated. The crude product was purified using flash column chromatography (SiO_2; DCM/MeOH = 95 : 5) providing 232 mg of the desired product (87% yield). ^{1}H NMR (400 MHz, $CDCl_3$) δ 8.32 (s, 1H), 7.59 (dd, *J* = 8.1 Hz, 1H), 7.16 (d, *J* = 8.3 Hz, 1H), 4.96 (dd, *J* =12.1, 5.1 Hz, 1H), 3.39 (s, 4H), 3.30 (s, 2H), 2.88 (m, 1H), 2.81 (m, 1H), 2.78 – 2.66 (m, 5H), 2.61 (s, 2H), 2.11 (m, 1H), 1.46 (s, 9H). ^{13}C NMR (101 MHz, $CDCl_3$) δ 172.5, 169.7, 167.3, 166.6, 156.0, 150.1, 135.7, 134.2, 123.5, 117.2, 115.2, 57.4, 52.9, 50.9, 49.2, 37.5, 31.5, 28.6, 22.6. LRMS (ESI): m/z 486.154 $[M + H]^+$, (calcd for $C_{24}H_{32}N_5O_6^+$, 486.227).

Preparation of tert-butyl 8-((2-(2,6-dioxopiperidin-3-yl)-1,3-dioxoisoindolin-4-yl)amino) octanoate (S8)

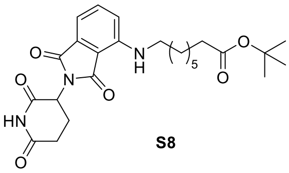

S8

Compound **S8** was prepared according to General procedure 6 using 4-fluoro-thalidomide (258 mg, 0.93 mmol) and the corresponding *tert*-butyl ester (201 mg, 0.93 mmol). The volatiles were then removed *in vacuo* and the crude product was purified using flash column chromatography (SiO_2; EtOAc/Hept = 2 : 3) providing 251 mg of the desired product (57% yield). The analytical results were consistent with data reported in the literature [(2)]. ^{1}H NMR (400 MHz, $CDCl_3$) δ 8.07 (s, 1H), 7.49 (dd, *J* = 8.5, 7.1 Hz, 1H), 7.08 (d, *J* = 7.0 Hz, 1H), 6.87 (d, *J* = 8.5 Hz, 1H), 6.22 (t, *J* = 5.7 Hz, 1H), 4.91 (dd, *J* = 12.1, 5.3 Hz, 1H), 3.25 (td, *J* = 7.1, 5.6 Hz, 2H), 2.96 – 2.67 (m, 3H), 2.20 (t, *J* = 7.5 Hz, 2H), 2.17 – 2.09 (m, 1H), 1.70 – 1.53 (m, 4H), 1.43 (s, 9H), 1.37 – 1.30 (m, 5H).

Preparation of tert-butyl 6-((2-(2,6-dioxopiperidin-3-yl)-1,3-dioxoisoindolin-4-yl)amino) hexanoate (S9)

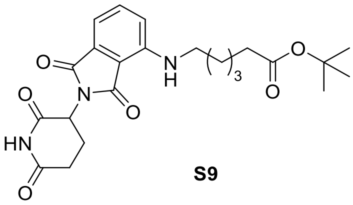

S9

Compound **S9** was prepared according to General procedure 6 using 4-fluoro-thalidomide (516 mg, 1.9 mmol) and the corresponding *tert*-butyl ester (350 mg, 1.9 mmol). The volatiles were then removed *in vacuo* and the crude product was purified using flash column chromatography

(SiO_2; EtOAc/Hept = 2 : 3) providing 623 mg of desired product (75% yield). The analytical results were consistent with data reported in the literature [(4)]. ^{1}H NMR (400 MHz, $CDCl_3$) δ 7.89 (s, 1H), 7.42 (dd, *J* = 8.5, 7.1 Hz, 1H), 7.02 (d, *J* = 7.0 Hz, 1H), 6.81 (d, *J* = 8.5 Hz, 1H), 6.16 (t, *J* = 5.7 Hz, 1H), 4.84 (dd, *J* = 12.1, 5.4 Hz, 1H), 3.20 (td, *J* = 7.1, 5.6 Hz, 2H), 2.89 – 2.59 (m, 3H), 2.17 (t, *J* = 7.4 Hz, 2H), 2.10 – 2.04 (m, 1H), 1.68 – 1.53 (m, 4H), 1.37 (s, 9H).

Preparation of tert-butyl 4-((2-(2,6-dioxopiperidin-3-yl)-1,3-dioxoisoindolin- 4-yl)amino) butanoate (S10)

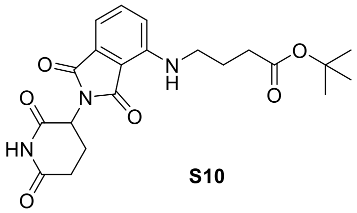

S10

Compound **S10** was prepared according to General procedure 6 using 4-fluoro-thalidomide (55 mg, 0.2 mmol) and the corresponding *tert*-butyl ester (34 mg, 0.2 mmol). The volatiles were then removed *in vacuo* and the crude product was purified using flash column chromatography (SiO_2; EtOAc/Hept = 2 : 3) providing 80 mg of the desired product (96% yield). ^{1}H NMR (400 MHz, $CDCl_3$) δ 8.02 (s, 1H), 7.50 (dd, *J* = 8.6, 7.1 Hz, 1H), 7.10 (d, *J* = 7.0 Hz, 1H), 6.93 (d, *J* = 8.5 Hz, 1H), 6.29 (t, *J* = 5.8 Hz, 1H), 4.96 – 4.84 (m, 1H), 3.33 (q, *J* = 6.6 Hz, 2H), 2.93 – 2.69 (m, 3H), 2.35 (t, *J* = 7.1 Hz, 2H), 2.17 – 2.10 (m, 1H), 1.94 (p, *J* = 7.1 Hz, 2H), 1.45 (s, 9H). ^{13}C NMR (101 MHz, $CDCl_3$) δ 172.4, 171.1, 169.6, 168.4, 167.7, 147.0, 136.3, 132.7, 116.8, 111.8, 110.2, 80.8, 49.0, 42.0, 32.7, 31.6, 28.3, 24.8, 22.9. LRMS (ESI): m/z 416.182 $[M + H]^+$, (calcd for $C_{21}H_{26}N_3O_6^+$, 416.181).

Preparation of tert-butyl 4-((2-(1-methyl-2,6-dioxopiperidin-3-yl)-1,3-dioxoisoindolin-4-yl) amino)butanoate (S11)

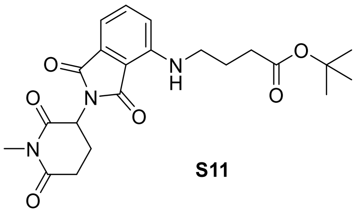

S11

Compound **S11** was prepared according to General procedure 6 using 4-fluoro-2-(1-methyl-2,6-dioxopiperidin-3-yl)isoindoline-1,3-dione (70 mg, 0.24 mmol) and the corresponding *tert*-butyl ester (38 mg, 0.24 mmol). The volatiles were then removed *in vacuo* and the crude product was purified using flash column chromatography (SiO_2; EtOAc/Hept = 2 : 3) providing 53 mg

of the desired product (51% yield). ^{1}H NMR (400 MHz, $CDCl_3$) δ 7.49 (dd, *J* = 8.5, 7.1 Hz, 1H), 7.09 (d, *J* = 7.1 Hz, 1H), 6.92 (d, *J* = 8.5 Hz, 1H), 6.28 (bs, 1H), 4.95 – 4.88 (m, 1H), 3.33 (t, *J* = 7.1 Hz, 2H), 3.21 (s, 3H), 3.01 – 2.90 (m, 1H), 2.84 – 2.72 (m, 2H), 2.34 (t, *J* = 7.1 Hz, 2H), 2.13 – 2.05 (m, 1H), 1.93 (p, *J* = 7.1 Hz, 2H), 1.45 (s, 9H). ^{13}C NMR (101 MHz, $CDCl_3$) δ 172.3, 171.4, 169.8, 169.1, 167.9, 147.0, 136.3, 132.7, 116.7, 111.7, 110.3, 80.8, 49.8, 42.0, 32.7, 32.1, 28.2, 27.4, 24.8, 22.3. LRMS (ESI): m/z 452.180 [M + Na]$^+$, (calcd for $C_{22}H_{27}N_3O_6Na^+$, 452.180).

Preparation of tert-butyl 6-((2-(1-methyl-2,6-dioxopiperidin-3-yl)-1,3-dioxoisoindolin-4-yl)amino)hexanoate (S12)

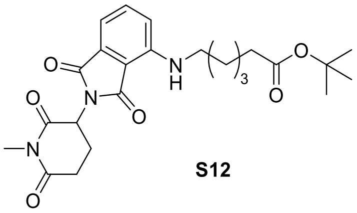

S12

Compound **S12** was prepared according to General procedure 6 using 4-fluoro-2-(1-methyl-2,6-dioxopiperidin-3-yl)isoindoline-1,3-dione (70 mg, 0.24 mmol) and the corresponding *tert*-butyl ester (45 mg, 0.24 mmol). The volatiles were then removed *in vacuo* and the crude product was purified using flash column chromatography (SiO_2; EtOAc/Hept = 2 : 3) providing 41 mg of the desired product (37% yield). ^{1}H NMR (400 MHz, CDCl3) δ 7.48 (t, *J* = 8.4, 7.3 Hz, 1H), 7.08 (d, *J* = 7.1 Hz, 1H), 6.87 (d, *J* = 8.5 Hz, 1H), 6.22 (t, *J* = 5.7 Hz, 1H), 4.90 (dd, *J* = 12.6, 5.3 Hz, 1H), 3.27 (td, *J* = 7.1, 5.6 Hz, 2H), 3.21 (s, 3H), 3.01 – 2.90 (m, 1H), 2.79 – 2.71 (m, 2H), 2.24 (t, *J* = 7.4 Hz, 2H), 2.16 – 2.04 (m, 1H), 1.66 (ddt, *J* = 17.1, 15.1, 7.4 Hz, 4H), 1.57 (s, 2H), 1.44 (s, 9H). ^{13}C NMR (101 MHz, $CDCl_3$) δ 173.0, 171.4, 169.8, 169.2, 167.9, 147.1, 136.2, 132.7, 116.7, 111.5, 110.1, 80.3, 49.8, 42.6, 35.5, 32.1, 29.2, 28.3, 27.4, 26.6, 24.9, 22.3. LRMS (ESI): m/z 458.231 [M + H]$^+$, (calcd for $C_{24}H_{32}N_3O_6^+$, 458.229).

Preparation of tert-butyl 11-(4-(((2-(2,6-dioxopiperidin-3-yl)- 1,3-dioxoisoindolin-4-yl) amino) methyl)-1H-1,2,3-triazol-1-yl)undecanoate (S13)

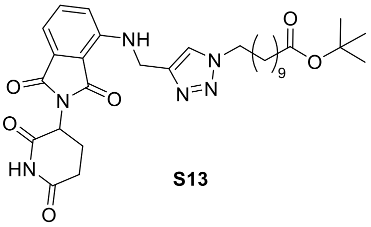

S13

Compound **S13** was prepared according to General procedure 4 using compound **56** (80 mg, 0.26 mmol) and the corresponding *tert*-butyl ester (73 mg, 0.26 mmol). The volatiles were then removed *in vacuo* and the crude product was purified using flash column chromatography (SiO_2; EtOAc/Hept = from 40 : 60 to 50 : 50) providing 85 mg of desired product (55% yield). ^1H NMR (400 MHz, $CDCl_3$) δ 7.99 (s, 1H), 7.49 (dd, *J* = 8.5, 7.1 Hz, 1H), 7.45 (s, 1H), 7.14 (d, *J* = 7.1 Hz, 1H), 7.00 (d, *J* = 8.5 Hz, 1H), 6.68 (t, *J* = 6.0 Hz, 1H), 4.92 (dd, *J* = 12.2, 5.4 Hz, 1H), 4.64 (d, *J* = 6.0 Hz, 2H), 4.31 (t, *J* = 7.3 Hz, 2H), 2.95 – 2.67 (m, 3H), 2.19 (t, *J* = 7.5 Hz, 2H), 2.13 (m, 1H), 1.88 (t, *J* = 7.2 Hz, 2H), 1.44 (s, 9H), 1.33 – 1.21 (m, 14H). ^{13}C NMR (101 MHz, $CDCl_3$) δ 173.2, 172.5, 171.0, 164.9, 163.4, 142.7, 140.8, 132.5, 131.6, 122.7, 121.8, 119.8, 113.4, 82.3, 52.5, 49.8, 38.3, 35.1, 30.3, 29.7, 29.2, 28.6, 27.7, 26.8, 26.1, 23.2. LRMS (ESI): m/z 595.35 $[M + H]^+$, (calcd for $C_{31}H_{43}N_6O_6^+$, 595.32).

Preparation of tert-butyl 9-(4-(((2-(2,6-dioxopiperidin-3-yl)-1,3-dioxoisoindolin-4-yl)amino) methyl)-1H-1,2,3-triazol-1-yl)nonanoate (S14)

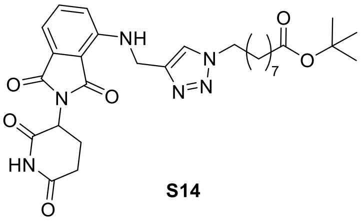

S14

Compound **S14** was prepared according to General procedure 4 using compound **56** (85 mg, 0.27 mmol) and the corresponding *tert*-butyl ester (70 mg, 0.27 mmol). The volatiles were removed *in vacuo* and the crude product was purified using flash column chromatography (SiO_2; EtOAc/Hept = from 40 : 60 to 50 : 50) affording 73 mg of desired product (47% yield). ^1H NMR (400 MHz, $CDCl_3$) δ 8.06 (s, 1H), 7.49 (dd, *J* = 8.5, 7.2 Hz, 1H), 7.44 (s, 1H), 7.14 (d, *J* = 7.2, 0.6 Hz, 1H), 7.00 (d, *J* = 8.5 Hz, 1H), 6.67 (t, *J* = 5.9 Hz, 1H), 4.92 (dd, *J* = 12.1, 5.4 Hz, 1H), 4.64 (d, *J* = 6.0 Hz, 2H), 4.31 (t, *J* = 7.9, 6.5 Hz, 2H), 2.94 – 2.67 (m, 3H), 2.21 – 2.15 (m, 2H), 2.15 – 2.09 (m, 1H), 1.92 – 1.83 (m, 1H), 1.43 (s, 9H), 1.30 – 1.22 (m, 11H). ^{13}C NMR (101 MHz, $CDCl_3$) δ 173.8, 172.3, 170.3, 165.5, 164.8, 143.4, 141.0, 131.4, 130.3, 123.1, 121.4, 120.6, 111.5, 80.25, 51.7, 50.2, 37.3, 35.1, 30.5, 29.2, 28.6, 27.7, 26.4, 24.3. LRMS (ESI): m/z 567.30 $[M + H]^+$, (calcd for $C_{29}H_{39}N_6O_6^+$, 567.29).

Preparation of tert-butyl 3-(4-(((2-(2,6-dioxopiperidin-3-yl)-1,3-dioxoisoindolin-4-yl)amino)methyl)-1H-1,2,3-triazol-1-yl)propanoate (S15)

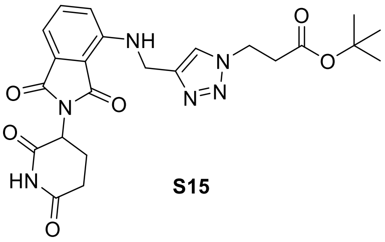

S15

Compound **S15** was prepared according to General procedure 4 using compound **56** (147 mg, 0.47 mmol) and the corresponding *tert*-butyl ester (81 mg, 0.47 mmol). The volatiles were then removed *in vacuo* and the crude product was purified using flash column chromatography (SiO2; EtOAc/Hept = from 40 : 60 to 50 : 50) affording 106 mg of desired product (46% yield). ^{1}H NMR (400 MHz, CDCl3) δ 7.94 (s, 1H), 7.56 (s, 1H), 7.49 (dd, *J* = 7.3, 0.8 Hz, 1H), 7.14 (d, *J* = 6.6 Hz, 1H), 7.00 (d, *J* = 8.5 Hz, 1H), 6.68 (t, *J* = 6.0 Hz, 1H), 4.91 (dd, *J* = 12.1, 5.4 Hz, 1H), 4.62 (d, *J* = 6.0 Hz, 2H), 4.58 (t, *J* = 6.5 Hz, 2H), 2.99 – 2.68 (m, 6H), 1.38 (s, 9H). ^{13}C NMR (101 MHz, $CDCl_3$) δ 173.3, 170.8, 170.4, 166.2, 165.1, 144.0, 141.5, 132.2, 130.8, 122.7, 121.1, 120.2, 112.7, 80.8, 52.3, 44.9, 37.7, 34.1, 31.4, 27.4, 25.9. LRMS (ESI): m/z 483.22 $[M + H]^+$, (calcd for $C_{23}H_{27}N_6O_6^+$, 483.20).

Preparation of 9-bromo-N-(2-(2,6-dioxopiperidin-3-yl)-1-oxoisoindolin-4-yl)nonanamide (52)

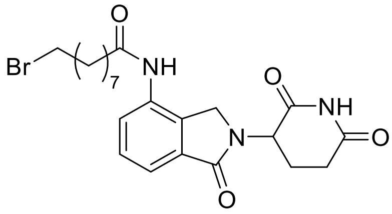

52

Compound **52** was prepared according to General procedure 8 starting from 9-bromononanoic acid (200 mg, 0.8 mmol, 1 eq). The crude product was purified using flash column chromatography (SiO_2; EtOAc) affording 110 mg of the desired product (27% yield). ^{1}H NMR (400 MHz, DMSO-d_6) δ 11.02 (s, 1H), 9.76 (s, 1H), 7.81 (dd, *J* = 6.9, 2.0 Hz, 1H), 7.53 – 7.38 (m, 2H), 5.15 (dd, *J* = 13.3, 5.1 Hz, 1H), 4.35 (dd, *J* = 25.6, 17.5 Hz, 2H), 3.53 (t, *J* = 6.7 Hz, 2H), 2.92 (ddd, *J* = 18.1, 13.5, 5.4 Hz, 1H), 2.68 – 2.56 (m, 1H), 2.41 – 2.30 (m, 3H), 2.11 – 1.99 (m, 1H), 1.80 (p, *J* = 6.7 Hz, 2H), 1.61 (t, *J* = 7.2 Hz, 2H), 1.42 – 1.25 (m, 8H). ^{13}C NMR (101 MHz, DMSO-d_6) δ 174.5, 172.9, 171.4, 171.1, 167.8, 133.8, 133.7, 132.7, 128.6, 125.2,

119.0, 51.5, 46.5, 35.8, 35.2, 33.6, 32.2, 31.2, 28.6, 28.6, 28.4, 28.0, 27.5, 25.0, 24.4, 22.7. LRMS (ESI): m/z 478.102, 480.099 $[M + H]^+$, (calcd for $C_{22}H_{29}BrN_3O_4^+$, 478.133, 480.131).

Preparation of 11-bromo-N-(2-(2,6-dioxopiperidin-3-yl)-1-oxoisoindolin-4-yl)undecanamide (53)

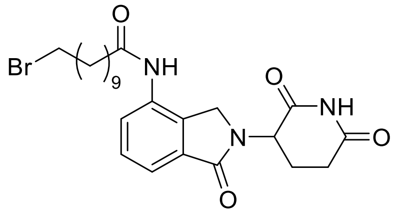

53

Compound **53** was prepared according to General procedure 8 starting from 11-bromoundecanoic acid (200 mg, 0.8 mmol, 1 eq). The crude product was purified using flash column chromatography (SiO_2; EtOAc/Hept 7 : 3) affording 125 mg of the desired product (31% yield). ^{1}H NMR (400 MHz, DMSO-d_6) δ 11.02 (s, 1H), 9.75 (s, 1H), 7.80 (dd, J = 6.9, 2.1 Hz, 1H), 7.60 – 7.38 (m, 2H), 5.14 (dd, J = 13.3, 5.1 Hz, 1H), 4.35 (dd, J = 25.8, 17.6 Hz, 2H), 3.51 (t, J = 6.7 Hz, 2H), 2.92 (ddd, J = 18.1, 13.5, 5.4 Hz, 1H), 2.68 – 2.57 (m, 1H), 2.39 – 2.30 (m, 3H), 2.10 – 1.97 (m, 1H), 1.78 (p, J = 6.9 Hz, 2H), 1.60 (t, J = 7.0 Hz, 2H), 1.36 – 1.23 (m, 12H). ^{13}C NMR (101 MHz, DMSO-d_6) δ 175.0, 172.9, 171.3, 171.1, 168.6, 167.8, 133.8, 132.7, 128.4, 125.1, 122.2, 119.0, 109.1, 51.5, 46.4, 35.8, 35.2, 34.4, 32.2, 31.2, 28.8, 28.7, 28.1, 27.5, 25.1, 22.7. LRMS (ESI): m/z 506.165, 508.164 $[M + H]^+$, (calcd for $C_{24}H_{33}BrN_3O_4^+$, 506.164, 508.162).

Preparation of N-(3-((6-(4-(4-((4,4-dimethylpiperidin-1-yl)methyl)phenyl)-2-oxo-1,4,9-triazaspiro[5.5]undecan-9-yl)pyrimidin-4-yl)amino)propyl)-3-(2-(2-((2-(2,6-dioxopiperidin-3-yl)-1,3-dioxoisoindolin-4-yl)amino)ethoxy)ethoxy)propenamide (1)

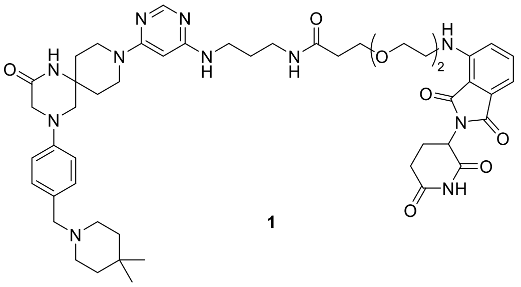

1

Compound **1** was prepared according to General procedure 3 (HATU) using the corresponding amine (159 mg, 0.306 mmol) and carboxylic acid (132 mg, 0.306 mmol). The crude product was purified first using flash column chromatography (Al_2O_3; DCM/MeOH = from 90 : 10 to

80 : 20), then using semipreparative HPLC providing 15 mg of the desired product (5% yield). ^{1}H NMR (500 MHz, DMSO-d_6) δ 11.09 (s, 1H), 8.25 (s, 1H), 7.98 (s, 1H), 7.86 – 7.80 (m, 1H), 7.57 (t, J = 7.8 Hz, 1H), 7.15 – 7.09 (m, 3H), 7.03 (d, J = 7.0 Hz, 1H), 6.89 (d, J = 8.4 Hz, 2H), 6.64 – 6.56 (m, 2H), 5.61 (s, 1H), 5.05 (dd, J = 12.7, 5.4 Hz, 1H), 3.81 – 3.74 (m, 2H), 3.65 (s, 2H), 3.59 (t, J = 6.0 Hz, 4H), 3.55 – 3.52 (m, 2H), 3.50 – 3.47 (m, 2H), 3.44 (q, J = 5.6 Hz, 4H), 3.20 – 3.15 (m, 2H), 3.08 (q, J = 6.6 Hz, 2H), 2.87 (ddd, J = 16.7, 13.7, 5.4 Hz, 2H), 2.65 – 2.55 (m, 2H), 2.33 – 2.25 (m, 4H), 2.06 – 1.97 (m, 1H), 1.90 (s, 1H), 1.76 – 1.68 (m, 2H), 1.67 – 1.55 (m, 4H), 1.28 (t, J = 5.6 Hz, 3H), 1.23 (s, 1H), 0.87 (s, 6H). ^{13}C NMR (126 MHz, DMSO-d_6) δ 172.8, 172.1, 170.1, 170.1, 169.0, 167.3, 167.1, 163.3, 161.7, 157.4, 148.2, 146.4, 136.3, 132.1, 129.7, 128.6, 117.5, 114.3, 110.7, 109.3, 69.7, 69.6, 68.9, 66.9, 61.9, 52.8, 52.4, 51.4, 49.2, 48.6, 41.7, 38.3, 38.0, 36.3, 36.2, 34.3, 31.0, 29.1, 28.3, 22.2, 22.1, 21.1. LRMS (ESI): m/z 936.512 [M + H]$^+$, (calcd for $C_{49}H_{66}N_{11}O_8^+$, 936.500).

Preparation of N-(3-((6-(4-(4-((4,4-dimethylpiperidin-1-yl)methyl)phenyl)- 2-oxo-1,4,9-triazaspiro[5.5]undecan-9-yl)pyrimidin-4-yl)amino)propyl)-3-(2-(2-(2-((2-(2,6-dioxopiperidin-3-yl)-1,3-dioxoisoindolin-4-yl)amino)ethoxy)ethoxy)ethoxy)propenamide (2)

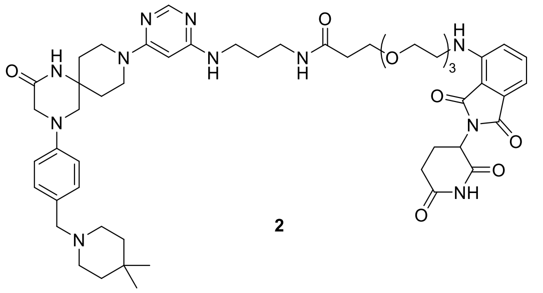

2

Compound **2** was prepared according to General procedure 3 (HATU) using the corresponding amine (69 mg, 0.13 mmol) and carboxylic acid (64 mg, 0.13 mmol). The crude product was purified first using flash column chromatography (Al_2O_3; DCM/MeOH = from 90 : 10 to 80 : 20), then using semipreparative HPLC providing 28 mg of the desired product (22% yield). ^{1}H NMR (500 MHz, DMSO-d_6) δ 11.09 (s, 1H), 8.25 (s, 1H), 7.98 (s, 1H), 7.84 (t, J = 5.6 Hz, 1H), 7.57 (t, J = 7.8 Hz, 1H), 7.16 – 7.09 (m, 3H), 7.03 (d, J = 7.0 Hz, 1H), 6.89 (d, J = 8.2 Hz, 2H), 6.60 (dt, J = 11.5, 5.9 Hz, 2H), 5.61 (s, 1H), 5.05 (dd, J = 12.8, 5.5 Hz, 1H), 3.78 (d, J = 13.8 Hz, 2H), 3.65 (s, 2H), 3.59 (dt, J = 17.1, 6.0 Hz, 4H), 3.56 – 3.49 (m, 4H), 3.47 (h, J = 5.7 Hz, 8H), 3.21 – 3.15 (m, 2H), 3.08 (q, J = 6.6 Hz, 2H), 2.87 (ddd, J = 16.8, 13.7, 5.4 Hz, 2H), 2.65 – 2.54 (m, 2H), 2.28 (q, J = 5.9, 5.2 Hz, 4H), 2.02 (ddd, J = 12.2, 6.9, 4.4 Hz, 1H), 1.90 (s, 1H), 1.72 (dd, J = 14.2, 5.1 Hz, 2H), 1.61 (qd, J = 13.9, 11.3, 5.5 Hz, 4H), 1.28 (t, J = 5.6 Hz, 3H), 1.23 (s, 1H), 0.87 (s, 6H). ^{13}C NMR (126 MHz, DMSO-d_6) δ 172.8, 170.1, 170.1, 169.0, 167.3,

167.1, 163.3, 161.7, 157.4, 148.1, 146.4, 136.3, 132.1, 129.7, 128.7, 117.5, 114.3, 110.7, 109.3, 69.8, 69.7, 69.6, 68.9, 66.9, 62.0, 52.8, 52.4, 51.4, 49.3, 48.6, 41.7, 38.4, 36.3, 36.2, 34.3, 31.0, 29.0, 28.3, 22.2, 21.2. LRMS (ESI): m/z 980.537 $[M + H]^+$, (calcd for $C_{51}H_{70}N_{11}O_9^+$, 980.530).

Preparation of N-(3-((6-(4-(4-((4,4-dimethylpiperidin-1-yl)methyl)phenyl)- 2-oxo-1,4,9-triazaspiro[5.5]undecan-9-yl)pyrimidin-4-yl)amino)propyl)-1-((2-(2,6-dioxopiperidin-3-yl)-1,3-dioxoisoindolin-4-yl)amino)-3,6,9,12-tetraoxapentadecan-15-amide (3)

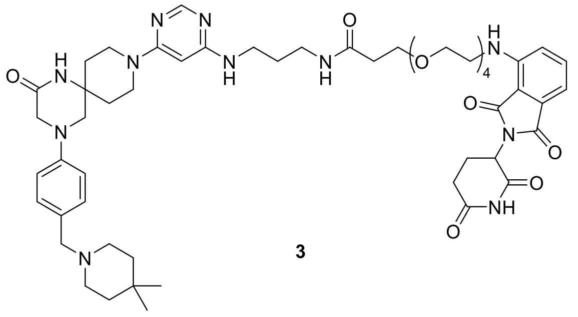

3

Compound **3** was prepared according to General procedure 3 (HATU) using the corresponding amine (50 mg, 0.096 mmol) and carboxylic acid (50 mg, 0.096 mmol). The crude product was purified first using flash column chromatography (Al_2O_3; DCM/MeOH = from 90 : 10 to 80 : 20), then using semipreparative HPLC providing 20 mg of the desired product (20% yield). ^{1}H NMR (500 MHz, DMSO-*d*$_6$) δ 11.09 (s, 1H), 8.25 (s, 1H), 7.98 (s, 1H), 7.84 (t, *J* = 5.7 Hz, 1H), 7.58 (d, *J* = 7.4 Hz, 1H), 7.15 – 7.10 (m, 3H), 7.04 (d, *J* = 7.1 Hz, 1H), 6.89 (d, *J* = 8.4 Hz, 2H), 6.64 – 6.57 (m, 2H), 5.61 (s, 1H), 5.05 (dd, *J* = 12.8, 5.5 Hz, 1H), 3.81 – 3.75 (m, 2H), 3.65 (s, 2H), 3.63 – 3.53 (m, 5H), 3.53 – 3.41 (m, 12H), 3.19 (h, *J* = 4.9 Hz, 2H), 3.08 (q, *J* = 6.5 Hz, 2H), 2.87 (ddd, *J* = 16.8, 13.8, 5.4 Hz, 2H), 2.65 – 2.54 (m, 2H), 2.33 – 2.25 (m, 4H), 2.06 – 1.97 (m, 1H), 1.90 (s, 1H), 1.76 – 1.68 (m, 2H), 1.67 – 1.56 (m, 4H), 1.28 (t, *J* = 5.6 Hz, 3H), 1.24 (s, 1H), 0.87 (s, 6H). ^{13}C NMR (126 MHz, DMSO-*d*$_6$) δ 172.8, 172.1, 170.1, 170.1, 169.0, 167.3, 167.1, 163.3, 161.7, 157.4, 149.8, 148.2, 146.4, 136.3, 134.7, 132.1, 132.0, 129.8, 123.4, 122.1, 117.5, 114.3, 110.7, 109.3, 69.8, 69.8, 69.8, 69.7, 69.5, 68.9, 66.9, 61.9, 52.8, 52.4, 51.4, 49.2, 48.6, 41.7, 38.3, 38.0, 36.3, 36.2, 34.3, 31.0, 29.1, 28.3, 22.2. LRMS (ESI): m/z 1024.564 $[M + H]^+$, (calcd for $C_{53}H_{74}N_{11}O_{10}^+$, 1024.550).

Preparation of N-(3-((6-(4-(4-((4,4-dimethylpiperidin-1-yl)methyl)phenyl)- 2-oxo-1,4,9-triazaspiro[5.5]undecan-9-yl)pyrimidin-4-yl)amino)propyl)-1-((2-(2,6-dioxopiperidin-3-yl)-1,3-dioxoisoindolin-4-yl)amino)-3,6,9,12,15-pentaoxaoctadecan-18-amide (4)

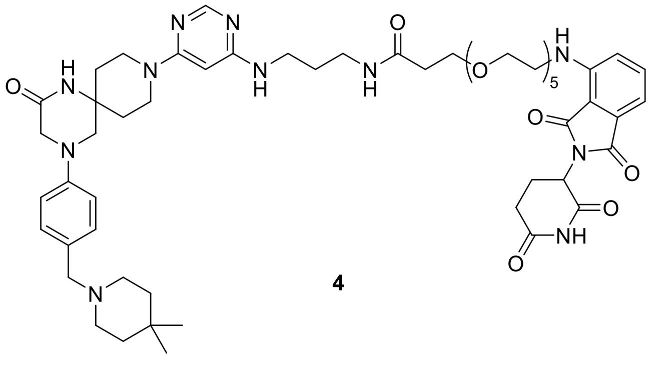

4

Compound **4** was prepared according to General procedure 3 (HATU) using the corresponding amine (51 mg, 0.098 mmol) and carboxylic acid (56 mg, 0.096 mmol). The crude product was purified first using flash column chromatography (Al_2O_3; DCM/MeOH = from 90 : 10 to 80 : 20), then using semipreparative HPLC providing 13 mg of the desired product (12% yield). ^{1}H NMR (500 MHz, DMSO-*d*$_6$) δ 11.09 (s, 1H), 8.25 (s, 1H), 7.98 (s, 1H), 7.89 – 7.80 (m, 1H), 7.58 (t, *J* = 7.9 Hz, 1H), 7.16 – 7.09 (m, 3H), 7.04 (d, *J* = 7.0 Hz, 1H), 6.89 (d, *J* = 8.3 Hz, 2H), 6.64 – 6.57 (m, 2H), 5.61 (s, 1H), 5.05 (dd, *J* = 12.7, 5.4 Hz, 1H), 3.81 – 3.73 (m, 2H), 3.65 (s, 2H), 3.63 – 3.54 (m, 6H), 3.54 – 3.43 (m, 17H), 3.21 – 3.15 (m, 2H), 3.08 (q, *J* = 6.6 Hz, 2H), 2.87 (ddd, *J* = 16.9, 13.8, 5.4 Hz, 2H), 2.65 – 2.54 (m, 2H), 2.32 – 2.24 (m, 4H), 2.06 – 1.97 (m, 1H), 1.90 (s, 1H), 1.76 – 1.68 (m, 2H), 1.67 – 1.55 (m, 4H), 1.28 (t, *J* = 5.6 Hz, 3H), 1.23 (s, 1H), 0.87 (s, 6H). ^{13}C NMR (126 MHz, DMSO-*d*$_6$) δ 172.8, 172.1, 170.1, 170.1, 169.0, 167.3, 167.1, 163.3, 161.7, 157.4, 148.1, 146.4, 136.3, 134.5, 132.1, 131.6, 129.7, 128.7, 123.1, 117.5, 114.3, 110.7, 109.3, 69.9, 69.8, 69.8, 69.7, 69.5, 68.9, 66.9, 62.0, 52.9, 52.4, 51.4, 49.3, 48.6, 48.6, 41.7, 38.4, 38.0, 36.3, 36.2, 34.3, 31.0, 29.1, 28.3, 22.2, 21.2. LRMS (ESI): m/z 1068.591 [M + H]$^+$, (calcd for $C_{55}H_{78}N_{11}O_{11}^+$, 1068.580).

Preparation of N-(3-((6-(4-(4-((4,4-dimethylpiperidin-1-yl)methyl)phenyl)-2-oxo-1,4,9-triazaspiro[5.5]undecan-9-yl)pyrimidin-4-yl)amino)propyl)-9-((2-(2,6-dioxopiperidin-3-yl)-1-oxoisoindolin-4-yl)amino)nonanamide (5)

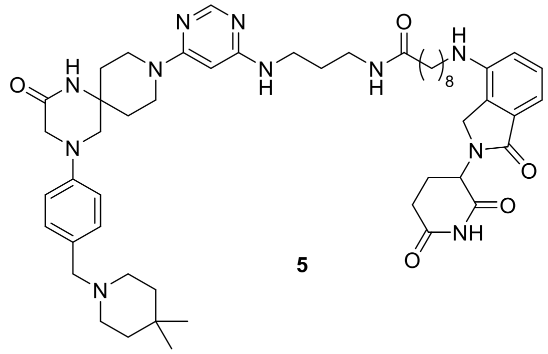

Compound **5** was prepared according to General procedure 3 (COMU) using the corresponding amine (63 mg, 0.12 mmol) and carboxylic acid (50 mg, 0.12 mmol). The reaction mixture was concentrated *in vacuo* and the crude product was purified using two consecutive preparative thin layer chromatography (SiO_2; DCM/MeOH = 84 : 16) providing 6 mg of the desired product (5% yield). ^{1}H NMR (400 MHz, DMSO-d_6) δ 11.00 (s, 1H), 8.27 (s, 1H), 7.98 (s, 1H), 7.78 (t, J = 5.6 Hz, 1H), 7.27 (t, J = 7.7 Hz, 1H), 7.14 (d, J = 8.2 Hz, 2H), 6.91 (m, 3H), 6.72 (d, J = 8.0 Hz, 1H), 6.64 (m, 1H), 5.61 (s, 1H), 5.54 (t, J = 5.5 Hz, 1H), 5.10 (dd, J = 13.2, 5.1 Hz, 1H), 4.22 (d, J = 17.3 Hz, 1H), 4.12 (d, J = 17.2 Hz, 1H), 3.77 (m, 2H), 3.66 (s, 2H), 3.17 (m, 2H), 3.08 (p, J = 6.6 Hz, 4H), 2.92 (ddd, J = 18.0, 13.4, 5.6 Hz, 2H), 2.69 – 2.57 (m, 2 H), 2.36 – 2.23 (m, 2H), 2.04 (t, J = 7.4 Hz, 2H), 1.72 (m, 2H), 1.58 (m, 6H), 1.47 (t, J = 7.1 Hz, 2H), 1.31 (m, 4H), 1.25 (m, 8H), 0.88 (s, 6H). ^{13}C NMR (151 MHz, MeOD-d_4) δ 176.4, 174.7, 172.4, 172.3, 170.7, 164.5, 163.3, 163.2, 162.9, 158.2, 150.7, 145.1, 132.8, 132.8, 130.5, 127.9, 115.6, 113.6, 111.7, 62.3, 54.3, 54.1, 53.4, 52.1, 50.1, 47.2, 44.4, 41.5, 39.7, 39.5, 39.4, 39.3, 39.1, 37.8, 37.8, 37.1, 35.4, 32.3, 30.7, 30.2, 30.2, 30.1, 30.0, 28.9, 28.0, 26.9, 26.9, 24.1, 23.6, 14.3. LRMS (ESI): m/z 918.574 $[M + H]^+$, (calcd for $C_{51}H_{73}N_{11}O_5^+$, 918.560).

Preparation of N-(3-((6-(4-(4-((4,4-dimethylpiperidin-1-yl)methyl)phenyl)-2-oxo -1,4,9-triazaspiro[5.5]undecan-9-yl)pyrimidin-4-yl)amino)propyl)-11-((2-(2,6-dioxopiperidin-3-yl)-1-oxoisoindolin-4-yl)amino)undecanamide (6)

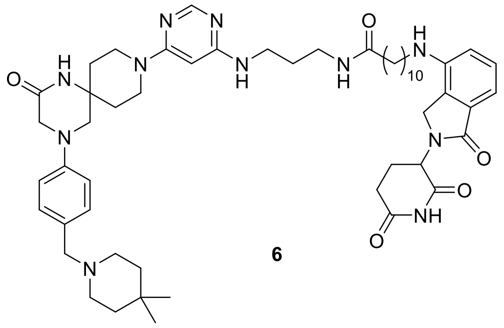

6

Compound **6** was prepared according to General procedure 3 (COMU) using the corresponding amine (47 mg, 0.090 mmol) and carboxylic acid (40 mg, 0.090 mmol). The reaction mixture was evaporated and the crude product was purified using flash column chromatography (SiO_2; DCM/MeOH = from 90 : 10 to 80 : 20) providing 36 mg of the desired product (42% yield). ^{1}H NMR (500 MHz, DMSO-d_6) δ 11.00 (s, 1 H), 8.32 (s, 1H), 7.98 (s, 1H), 7.84 (t, *J* = 5.7 Hz, 1H), 7.43 – 7.34 (m, 2H), 7.27 (t, *J* = 7.7 Hz, 1H), 6.97 (d, *J* = 8.3 Hz, 2H), 6.91 (d, *J* = 7.4 Hz, 1H), 6.72 (d, *J* = 8.1 Hz, 1H), 6.67 (m, 1H), 5.62 (s, 1H), 5.57 (t, *J* = 5.5 Hz, 1H), 5.10 (dd, *J* = 13.3, 5.1 Hz, 1H), 4.23 (d, *J* = 17.2 Hz, 1H), 4.12 (d, *J* = 17.2 Hz, 1H), 3.81 – 3.74 (m, 2 H), 3.72 (s, 2H), 3.59 (m, 2H), 3.51 (m, 2H), 3.45 (s, 2H), 3.17 (m, 2H), 3.08 (dq, *J* = 13.0, 6.5 Hz, 4H), 2.91 (ddd, *J* = 15.1, 9.8, 5.2, 4.7 Hz, 2H), 2.61 (m, 2H), 2.29 (m, 2H), 2.03 (t, *J* = 7.0 Hz, 2H), 1.70 (m, 2H), 1.59 (m, 6H), 1.46 (m, 4H), 1.34 (m, 2H), 1.25 (m, 14 H), 0.94 (s, 6H). ^{13}C NMR (126 MHz, DMSO-d_6) δ 173.0, 172.2, 171.3, 169.0, 166.9, 163.2, 161.7, 158.0, 157.8, 157.3, 143.8, 132.1, 129.2, 126.5, 118.6, 116.2, 113.8, 111.7, 109.9, 69.8, 53.3, 52.4, 51.9, 50.7, 47.7, 45.8, 42.8, 41.6, 38.0, 36.2, 35.5, 34.2, 31.3, 29.1, 29.0, 28.9, 28.8, 28.7, 28.6, 27.8, 26.7, 25.4, 22.8, 22.1, 18.6, 18.0, 16.8, 12.3. LRMS (ESI): m/z 946.605 $[M + H]^+$, (calcd for $C_{53}H_{77}N_{11}O_5^+$, 946.600).

Preparation of N-(3-((6-(4-(4-((4,4-dimethylpiperidin-1-yl)methyl)phenyl)-2-oxo-1,4,9-triaza- spiro[5.5]undecan-9-yl)pyrimidin-4-yl)amino)propyl)-15-((2-(2,6-dioxopiperidin-3-yl)-1-oxoisoindolin-4-yl)amino)pentadecanamide (7)

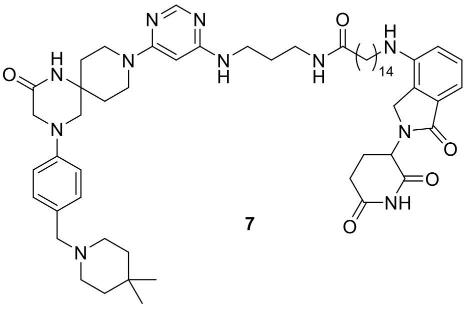

7

Compound **7** was prepared according to General procedure 3 (COMU) using the corresponding amine (117 mg, 0.224 mmol) and carboxylic acid (112 mg, 0.224 mmol). The reaction mixture was concentrated *in vacuo* and the crude product was purified using flash column chromatography (SiO_2; DCM/MeOH = from 90 : 10 to 80 : 20) providing 120 mg of the desired product (53% yield). ^1H NMR (400 MHz, MeOD-d_4) δ 8.11 (s, 1H), 7.42 (d, *J* = 8.7 Hz, 2H), 7.31 (t, *J* = 7.8 Hz, 1H), 7.08 – 6.99 (m, 3H), 6.81 (d, *J* = 8.0 Hz, 1H), 5.81 (s, 1H), 5.15 (dd, *J* = 13.3, 5.1 Hz, 1H), 4.32 (d, *J* = 16.9 Hz, 1H), 4.26 (d, *J* = 17.1 Hz, 1H), 4.22 (s, 2H), 3.96 – 3.86 (m, 4H), 3.80 – 3.71 (m, 2H), 3.55 (s, 2H), 3.28 – 3.25 (m, 2H), 3.20 (t, *J* = 7.2 Hz, 2H), 3.15 – 3.04 (m, 2H), 2.91 (ddd, *J* = 18.4, 13.4, 5.3 Hz, 1H), 2.48 (qd, *J* = 13.2, 4.7 Hz, 1H), 2.19 (t, *J* = 7.4 Hz, 2H), 1.98 – 1.90 (m, 2H), 1.88 – 1.78 (m, 4H), 1.70 – 1.56 (m, 8H), 1.35 – 1.26 (m, 20H), 1.05 (d, *J* = 15.1 Hz, 6H). ^{13}C NMR (101 MHz, MeOD-d_4) δ 176.6, 174.7, 172.5, 172.4, 170.4, 151.6, 145.2, 133.7, 133.0, 130.6, 128.0, 120.4, 115.7, 113.8, 111.8, 61.0, 54.0, 53.8, 53.6, 51.8, 47.5, 44.5, 42.0, 40.1, 37.6, 37.2, 36.6, 35.5, 32.4, 31.5, 30.6, 30.5, 30.4, 30.3, 30.2, 29.8, 28.8, 28.2, 27.0, 24.3, 23.5. LRMS (ESI): m/z 1002.664 $[M + H]^+$, (calcd for $C_{57}H_{84}N_{11}O_5^+$, 1002.670).

Preparation of N-(3-((6-(4-(4-((4,4-dimethylpiperidin-1-yl)methyl)phenyl)-2-oxo-1,4,9-triazaspiro[5.5]undecan-9-yl)pyrimidin-4-yl)amino)propyl)-11-(4-(((2-(2,6-dioxopiperidin-3-yl)-1,3-dioxoisoindolin-4-yl)amino)methyl)-1H-1,2,3-triazol-1-yl)undecanamide (8)

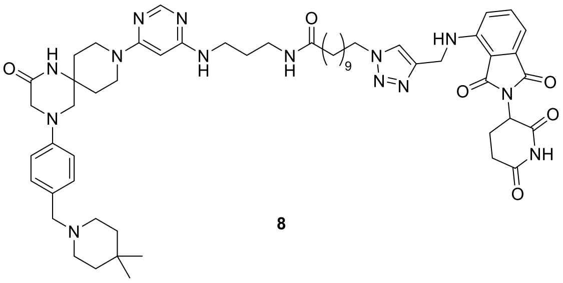

8

Compound **8** was prepared according to General procedure 3 (HATU) using the corresponding amine (80 mg, 0.15 mmol) and carboxylic acid (83 mg, 0.15 mmol). The reaction mixture was concentrated *in vacuo* and the crude product was purified using flash column chromatography (SiO_2; DCM/MeOH = from 90 : 10 to 80 : 20) providing 7 mg of the desired product (5% yield). ^{1}H NMR (600 MHz, MeOD-*d₄*) δ 8.04 (t, *J* = 5.6 Hz, 1H), 7.98 (s, 1H), 7.90 (s, 1H), 7.51 (dd, *J* = 8.5, 7.1 Hz, 1H), 7.27 (d, *J* = 8.7 Hz, 2H), 7.06 (dd, *J* = 7.8, 2.4 Hz, 2H), 6.98 (t, *J* = 6.1 Hz, 1H), 6.95 (d, *J* = 8.7 Hz, 1H), 5.66 (s, 1H), 5.04 (dd, *J* = 12.5, 5.5 Hz, 1H), 4.63 (d, *J* = 6.1 Hz, 2H), 4.34 (t, *J* = 7.0 Hz, 2H), 3.81 (s, 2H), 3.72 – 3.67 (m, 2H), 3.56 – 3.50 (m, 2H), 3.48 (s, 2H), 3.29 – 3.22 (m, 4H), 2.87 – 2.78 (m, 2H), 2.75 – 2.72 (m, 1H), 2.70 (s, 1H), 2.69 – 2.64 (m, 2H), 2.16 (t, *J* = 7.4 Hz, 2H), 2.10 – 2.02 (m, 2H), 1.92 – 1.86 (m, 2H), 1.85 – 1.81 (m, 2H), 1.77 – 1.73 (m, 2H), 1.62 – 1.55 (m, 4H), 1.47 (t, *J* = 5.7 Hz, 4H), 1.32 – 1.27 (m, 16H), 0.95 (s, 6H). ^{13}C NMR (151 MHz, MeOD-*d₄*) δ 176.4, 174.6, 171.6, 170.8, 170.4, 169.1, 164.5, 163.3, 163.2, 162.9, 162.7, 158.2, 150.4, 147.4, 147.3, 146.5, 137.0, 133.8, 132.6, 124.0, 118.2, 115.7, 112.4, 111.9, 54.3, 53.2, 52.3, 51.2, 50.5, 50.1, 41.5, 39.6, 38.9, 38.2, 37.9, 37.1, 33.0, 32.1, 31.6, 31.5, 31.0, 30.7, 30.6, 30.2, 30.2, 30.1, 30.1, 30.0, 29.8, 29.8, 29.0, 27.2, 26.9, 23.7, 18.4. LRMS (ESI): m/z 1041.615 [M + H]$^+$, (calcd for $C_{56}H_{78}N_{14}O_6^+$, 1041.610).

Preparation of N-(3-((6-(4-(4-((4,4-dimethylpiperidin-1-yl)methyl)phenyl)-2-oxo-1,4,9-triazaspiro[5.5]undecan-9-yl)pyrimidin-4-yl)amino)propyl)-9-(4-(((2-(2,6-dioxopiperidin-3-yl)-1,3-dioxoisoindolin-4-yl)amino)methyl)-1H-1,2,3-triazol-1-yl)nonanamide (9)

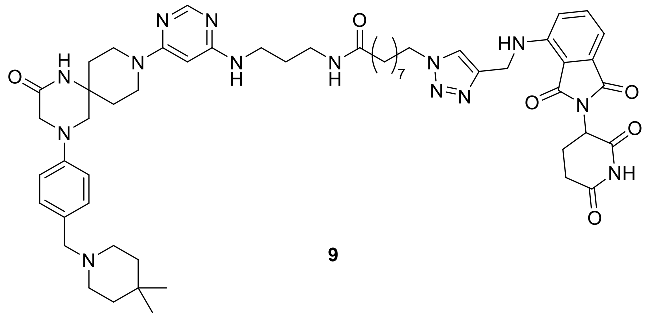

9

Compound **9** was prepared according to General procedure 3 (HATU) using the corresponding amine (67 mg, 0.13 mmol) and carboxylic acid (66 mg, 0.13 mmol). The reaction mixture was concentrated *in vacuo* and the crude product was purified using flash column chromatography (SiO_2; DCM/MeOH = from 90 : 10 to 80 : 20) providing 100 mg of the desired product (76% yield). ^{1}H NMR (400 MHz, MeOD-*d₄*) δ 7.98 (s, 1H), 7.91 (s, 1H), 7.51 (dd, *J* = 8.5, 7.1 Hz, 1H), 7.38 (d, *J* = 8.7 Hz, 2H), 7.07 (t, *J* = 7.9 Hz, 2H), 7.00 (d, *J* = 8.8 Hz, 2H), 5.68 (s, 1H), 5.04 (dd, *J* = 12.5, 5.4 Hz, 1H), 4.62 (s, 2H), 4.34 (t, *J* = 7.0 Hz, 2H), 4.11 (s, 2H), 3.86 (s, 2H), 3.83 – 3.77 (m, 2H), 3.59 – 3.54 (m, 2H), 3.52 (s, 2H), 3.29 – 3.22 (m, 4H), 3.11 – 3.06 (m,

4H), 2.75 – 2.66 (m, 2H), 2.16 (t, *J* = 7.4 Hz, 2H), 1.89 – 1.73 (m, 8H), 1.61 (t, *J* = 5.8 Hz, 4H), 1.56 (t, *J* = 7.3 Hz, 2H), 1.40 – 1.36 (m, 4H), 1.27 – 1.23 (m, 6H), 1.03 (s, 6H). ^{13}C NMR (101 MHz, MeOD-d_4) δ 176.4, 174.6, 171.6, 170.6, 170.5, 169.2, 164.6, 163.4, 158.3, 151.4, 147.4, 146.5, 137.2, 133.9, 133.5, 124.1, 118.4, 115.6, 112.5, 112.0, 61.4, 55.8, 54.3, 53.9, 51.9, 51.3, 50.2, 41.6, 39.6, 38.9, 37.9, 37.1, 36.9, 35.5, 32.2, 31.1, 30.2, 30.1, 29.9, 29.7, 28.9, 27.2, 26.9, 23.8, 13.2. LRMS (ESI): m/z 1013.584 [M + H]$^+$, (calcd for $C_{54}H_{73}N_{14}O_6^+$, 1013.580).

Preparation of N-(3-((6-(4-(4-((4,4-dimethylpiperidin-1-yl)methyl)-2,5-difluorophenyl)- 2-oxo-1,4,9-triazaspiro[5.5]undecan-9-yl)pyrimidin-4-yl)amino)propyl)-9-((2-(2,6-dioxopiperidin-3-yl)-1-oxoisoindolin-4-yl)amino)nonanamide (10)

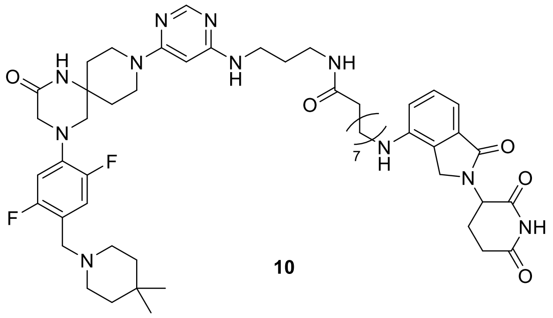

10

Compound **10** was prepared according to General procedure 3 (COMU) using the corresponding amine (85 mg, 0.14 mmol) and carboxylic acid (60 mg, 0.24 mmol). The reaction mixture was concentrated *in vacuo* and the crude product was purified using flash column chromatography (SiO_2; DCM/MeOH = from 95 : 5 to 90 : 10), followed by a second flash column chromatography (SiO_2; EtOAc/MeOH = from 90 : 10 to 80 : 20) providing 100 mg of the desired product (72% yield). ^{1}H NMR (400 MHz, $CDCl_3$) δ 10.31 (bs, 1H), 8.14 (s, 1H), 7.32 (t, *J* = 7.7 Hz, 1H), 7.20 (d, *J* = 7.5 Hz, 1H), 7.11 (dd, *J* = 12.9, 6.6 Hz, 1H), 7.02 (s, 1H), 6.76 (d, *J* = 8.1 Hz, 1H), 6.57 (dd, *J* = 10.9, 7.1 Hz, 1H), 6.36 (s, 1H), 5.74 (s, 1H), 5.43 (s, 1H), 5.20 (dd, *J* = 13.2, 5.2 Hz, 1H), 4.29 (d, *J* = 15.6 Hz, 1H), 4.12 (d, *J* = 15.6 Hz, 1H), 3.71 (m, 4H), 3.60 – 3.53 (m, 2H), 3.50 (s, 2H), 3.29 (m, 4H), 3.25 (s, 2H), 3.18 (q, *J* = 7.1, 6.6 Hz, 2H), 2.85 – 2.75 (m, 2H), 2.47 – 2.38 (m, 4H), 2.33 – 2.19 (m, 2H), 2.19 – 2.07 (m, 4H), 1.98 – 1.86 (m, 2H), 1.84 – 1.69 (m, 4H), 1.66 – 1.53 (m, 4H), 1.44 – 1.36 (m, 4H), 1.35 – 1.21 (m, 7H), 0.90 (s, 6H). ^{13}C NMR (101 MHz, $CDCl_3$) δ 173.8, 172.2, 171.1, 170.3, 168.3, 163.1, 162.3, 157.4, 156.2, 152.0, 149.9, 143.4, 137.6, 132.0, 129.8, 126.3, 118.6, 113.3, 112.5, 105.9, 105.7, 56.6, 56.5, 54.7, 53.5, 53.1, 51.9, 51.0, 49.8, 45.2, 43.7, 40.3, 38.9, 38.6, 38.3, 38.3, 36.9, 36.5, 35.3, 31.8, 29.8, 29.3, 29.2, 29.0, 28.9, 28.5, 26.8, 25.7, 23.7. LRMS (ESI): m/z 954.552 [M + H]$^+$, (calcd for $C_{51}H_{70}F_2N_{11}O_5^+$, 954.552).

Preparation of N-(3-((6-(4-(4-((4,4-dimethylpiperidin-1-yl)methyl)-2,5-difluorophenyl)- 2-oxo-1,4,9-triazaspiro[5.5]undecan-9-yl)pyrimidin-4-yl)amino)propyl)-11-((2-(2,6-dioxopiperidin-3-yl)-1-oxoisoindolin-4-yl)amino)undecanamide (11)

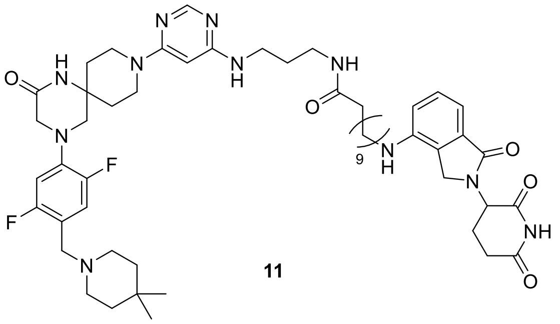

11

Compound **11** was prepared according to General procedure 3 (COMU) using the corresponding amine (40 mg, 0.07 mmol) and carboxylic acid (30 mg, 0.07 mmol). The reaction mixture was concentrated *in vacuo* and the crude product was purified using flash column chromatography (SiO_2; EtOAc/MeOH = from 90 : 10 to 80 : 20) providing 37 mg of the desired product (56% yield). ^{1}H NMR (400 MHz, DMSO) δ 11.00 (s, 1H), 10.87 (s, 1H), 8.36 (s, 1H), 8.29 (s, 1H), 7.99 (t, *J* = 5.5 Hz, 1H), 7.73 (dd, *J* = 13.4, 6.8 Hz, 1H), 7.27 (t, *J* = 7.7 Hz, 1H), 7.05 (dd, *J* = 11.7, 7.4 Hz, 1H), 6.91 (d, *J* = 7.4 Hz, 1H), 6.72 (d, *J* = 8.0 Hz, 1H), 5.94 (s, 1H), 5.10 (dd, *J* = 13.2, 5.1 Hz, 1H), 4.27 – 4.20 (m, 2H), 4.12 (d, *J* = 17.2 Hz, 1H), 3.69 (s, 2H), 3.40 – 3.33 (m, 3H), 3.34 – 3.27 (m, 2H), 3.21 – 3.01 (m, 8H), 2.92 (ddd, *J* = 18.0, 13.4, 5.2 Hz, 1H), 2.68 – 2.57 (m, 1H), 2.29 (qd, *J* = 13.4, 4.9 Hz, 1H), 2.13 – 1.97 (m, 3H), 1.91 – 1.70 (m, 5H), 1.66 (t, *J* = 6.8 Hz, 2H), 1.60 – 1.51 (m, 2H), 1.51 – 1.39 (m, 4H), 1.38 – 1.16 (m, 17H), 0.96 (d, *J* = 26.8 Hz, 6H). ^{13}C NMR (101 MHz, DMSO-d_6) δ 172.9, 172.3, 171.3, 168.9, 166.4, 158.3, 158.0, 156.5, 143.8, 140.5, 132.0, 129.2, 126.5, 120.5, 118.4, 115.5, 112.5, 111.7, 109.9, 54.6, 52.8, 52.2, 51.5, 50.9, 47.7, 45.8, 42.8, 35.9, 35.4, 34.6, 31.2, 31.1, 29.0, 28.9, 28.9, 28.8, 28.7, 28.5, 27.5, 26.7, 25.3, 23.1, 22.8. LRMS (ESI): m/z 982.584 [M + H]$^+$, (calcd for $C_{53}H_{74}F_2N_{11}O_5^+$, 982.583).

Preparation of N-(3-((6-(4-(4-((4,4-dimethylpiperidin-1-yl)methyl)-2,5-difluorophenyl)-2-oxo-1,4,9-triazaspiro[5.5]undecan-9-yl)pyrimidin-4-yl)amino)propyl)-9-(4-(((2-(2,6-dioxopiperidin-3-yl)-1,3-dioxoisoindolin-4-yl)amino)methyl)-1H-1,2,3-triazol-1-yl)nonanamide (12)

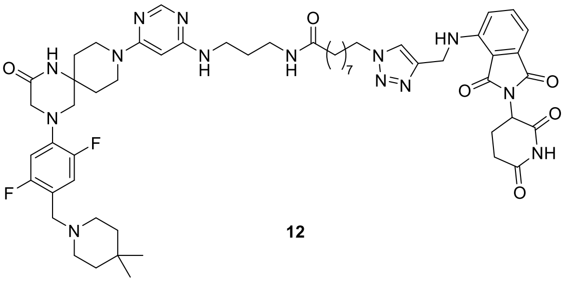

12

Compound **12** was prepared according to General procedure 3 (COMU) using the corresponding amine (34.9 mg, 0.063 mmol) and carboxylic acid (32.0 mg, 0.063 mmol). The reaction mixture was concentrated *in vacuo* and the crude product was purified using flash column chromatography (SiO_2; DCM/MeOH = from 100 : 5 to 100 : 20) providing 15 mg of the desired product (22% yield). ^{1}H NMR (400 MHz, $CDCl_3$) δ 10.38 (bs, 1H), 8.14 (s, 1H), 7.49 (s, 1H), 7.47 (dd, *J* = 8.5, 7.1 Hz, 1H), 7.17 – 7.12 (m, 1H), 7.11 (d, *J* = 7.1 Hz, 1H), 6.98 (d, *J* = 8.5 Hz, 1H), 6.90 (s, 1H), 6.70 (t, *J* = 5.9 Hz, 1H), 6.58 (dd, *J* = 10.9, 7.2 Hz, 1H), 6.28 (bs, 1H), 5.79 (bs, 1H), 5.44 (s, 1H), 4.91 (dd, *J* = 12.1, 5.4 Hz, 1H), 4.63 (d, *J* = 5.9 Hz, 2H), 4.32 (t, *J* = 7.0 Hz, 2H), 3.75 – 3.49 (m, 8H), 3.35 – 3.26 (m, 5H), 2.87 – 2.81 (m, 3H), 2.49 (bs, 4H), 2.17 – 2.05 (m, 2H), 1.95 – 1.91 (m, 8H), 1.60 – 1.51 (m, 2H), 1.45 – 1.38 (m, 4H), 1.28 – 1.17 (m, 10H), 0.92 (s, 6H). ^{13}C NMR (101 MHz, $CDCl_3$) δ 173.8, 171.8, 169.8, 169.6, 168.1, 167.7, 162.5, 162.3, 158.7, 157.2, 156.3, 152.3, 146.3, 145.2, 136.4, 132.5, 121.8, 118.9, 117.3, 112.4, 110.9, 105.9, 105.6, 63.9, 56.7, 54.5, 53.5, 53.0, 50.5, 49.7, 49.1, 40.3, 38.9, 38.3, 36.8, 36.5, 35.4, 32.1, 31.6, 31.1, 30.1, 29.8, 29.5, 29.3, 28.8, 28.4, 28.4, 26.2, 25.6, 23.0. LRMS (ESI): m/z 1049.565 $[M + H]^+$, (calcd for $C_{54}H_{71}F_2N_{14}O_6^+$, 1049.564).

Preparation of N-(3-((6-(4-(4-((4,4-dimethylpiperidin-1-yl)methyl)-2,5-difluorophenyl)-2-oxo-1,4,9-triazaspiro[5.5]undecan-9-yl)pyrimidin-4-yl)amino)propyl)-11-(4-(((2-(2,6-dioxopiperidin-3-yl)-1,3-dioxoisoindolin-4-yl)amino)methyl)-1H-1,2,3-triazol-1-yl)undecanamide (13)

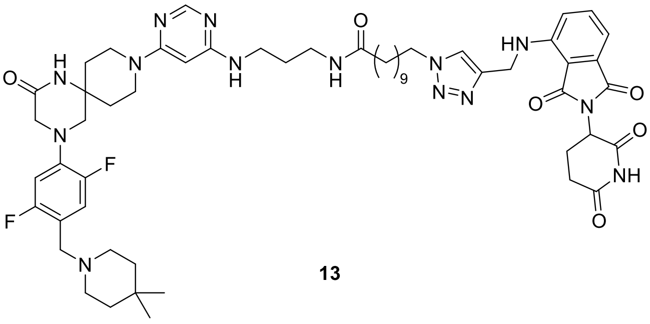

13

Compound **13** was prepared according to General procedure 3 (COMU) using the corresponding amine (33.08 mg, 0.059 mmol) and carboxylic acid (32.0 mg, 0.059 mmol). The reaction mixture was concentrated *in vacuo* and the crude product was purified using flash column chromatography (SiO_2; DCM/MeOH = from 100 : 10 to 100 : 20) providing 14 mg of the desired product (21% yield). ^{1}H NMR (400 MHz, $CDCl_3$) δ 10.38 (bs, 1H), 8.14 (s, 1H), 7.49 (s, 1H), 7.46 (dd, J = 8.5, 7.1 Hz, 1H), 7.15 – 7.12 (m, 1H), 7.10 (d, J = 7.1 Hz, 1H), 7.05 (s, 1H), 6.98 (d, J = 8.5 Hz, 1H), 6.70 (t, J = 5.9 Hz, 1H), 6.58 (dd, J = 10.9, 7.2 Hz, 1H), 6.28 (bs, 1H), 5.79 (bs, 1H), 5.44 (s, 1H), 4.91 (dd, J = 12.1, 5.4 Hz, 1H), 4.63 (d, J = 5.9 Hz, 2H), 4.32 (t, J = 7.0 Hz, 2H), 3.75 – 3.49 (m, 8H), 3.35 – 3.23 (m, 5H), 2.87 – 2.81 (m, 3H), 2.49 (bs, 4H), 2.17 – 2.05 (m, 2H), 1.95 – 1.91 (m, 8H), 1.60 – 1.51 (m, 2H), 1.45 – 1.38 (m, 4H), 1.28 – 1.17 (m, 14H), 0.92 (s, 6H). ^{13}C NMR (101 MHz, $CDCl_3$) δ 173.9, 171.9, 169.5, 168.2, 167.7, 162.9, 162.3, 158.7, 157.2, 156.3, 152.3, 149.9, 146.3, 145.1, 137.8, 136.3, 132.5, 121.7, 118.6, 117.3, 112.4, 110.9, 105.9, 105.6, 63.9, 56.6, 54.6, 53.5, 53.0, 50.6, 49.7, 49.1, 40.3, 38.9, 38.4, 36.9, 36.5, 35.3, 31.6, 30.2, 29.8, 29.5, 29.4, 29.1, 29.1, 29.1, 28.7, 28.4, 26.3, 25.8, 22.9, 14.3. LRMS (ESI): m/z 1077.593 [M + H]$^+$, (calcd for $C_{56}H_{75}F_2N_{14}O_6^+$, 1077.596).

Preparation of N-(3-(((6-(4-(4-((4,4-dimethylpiperidin-1-yl)methyl)-2,5-difluorophenyl)- 2-oxo-1,4,9-triazaspiro[5.5]undecan-9-yl)pyrimidin-4-yl)amino)methyl)benzyl)-4-((2-(2,6-dioxopiperidin-3-yl)-1,3-dioxoisoindolin-4-yl)amino)butanamide (14)

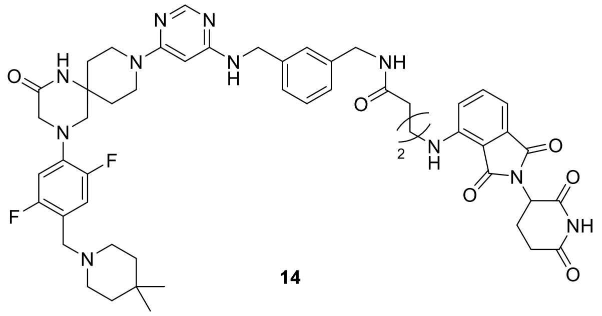

14

Compound **14** was prepared according to General procedure 3 (COMU) using the corresponding amine (55 mg, 0.08 mmol) and carboxylic acid (30 mg, 0.08 mmol). The reaction mixture was concentrated *in vacuo* and the crude product was purified using flash column chromatography (SiO_2; EtOAc/MeOH = 90 : 10) providing 47 mg of the desired product (59% yield). ^{1}H NMR (400 MHz, $CDCl_3$) δ 10.85 (bs, 1H), 8.16 (s, 1H), 7.43 (dd, J = 8.5, 7.1 Hz, 1H), 7.25 – 7.17 (m, 3H), 7.15 – 7.07 (m, 2H), 7.04 (d, J = 7.1 Hz, 1H), 6.92 – 6.84 (m, 2H), 6.57 (dd, J = 10.9, 7.1 Hz, 1H), 6.26 (t, J = 5.9 Hz, 1H), 6.09 – 6.04 (m, 1H), 5.77 (bs, 1H), 5.34 (s, 1H), 4.89 (dd, J = 12.2, 5.3 Hz, 1H), 4.38 (dd, J = 13.1, 5.7 Hz, 4H), 3.71 (s, 2H), 3.66

– 3.50 (m, 4H), 3.33 (q, *J* = 6.5 Hz, 2H), 3.25 (s, 2H), 2.90 – 2.69 (m, 3H), 2.46 (bs, 4H), 2.31 (t, *J* = 6.9 Hz, 2H), 2.15 – 2.06 (m, 1H), 2.05 – 1.96 (m, 2H), 1.94 – 1.83 (m, 2H), 1.81 – 1.70 (m, 4H), 1.45 – 1.38 (m, 4H), 0.92 (s, 6H). ^{13}C NMR (101 MHz, $CDCl_3$) δ 172.0, 171.9, 170.4, 169.8, 168.2, 167.8, 162.5, 157.6, 147.0, 139.1, 138.8, 136.4, 132.6, 129.1, 127.0, 126.7, 126.3, 116.9, 111.8, 110.3, 56.7, 54.3, 53.5, 53.0, 49.8, 49.1, 45.7, 43.6, 41.8, 40.3, 38.5, 35.2, 33.3, 31.7, 29.9, 28.5, 24.8, 23.0. LRMS (ESI): m/z 960.469 $[M + H]^+$, (calcd for $C_{51}H_{60}F_2N_{11}O_6^+$, 960.469).

Preparation of N-(3-(((6-(4-(4-((4,4-dimethylpiperidin-1-yl)methyl)-2,5-difluorophenyl)-2-oxo-1,4,9-triazaspiro[5.5]undecan-9-yl)pyrimidin-4-yl)amino)methyl)benzyl)-6-((2-(2,6-dioxopiperidin-3-yl)-1,3-dioxoisoindolin-4-yl)amino)hexanamide (15)

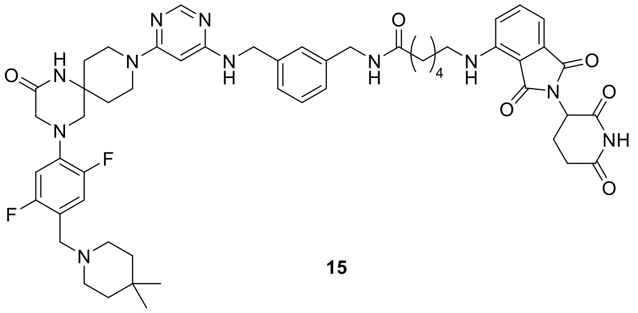

15

Compound **15** was prepared according to General procedure 3 using the corresponding amine (124 mg, 0.19 mmol) and carboxylic acid (95 mg, 0.19 mmol). The reaction mixture was evaporated and the crude product was purified using flash column chromatography (SiO_2; EtOAc/MeOH = from 85 : 15 to 80 : 20) providing 113 mg of the desired product (60% yield). ^{1}H NMR (400 MHz, MeOD-*d_4*) δ 7.98 (s, 1H), 7.51 (t, 1H), 7.29 – 7.20 (m, 3H), 7.18 (d, *J* = 6.7 Hz, 1H), 7.15 (d, *J* = 7.2 Hz, 1H), 7.03 – 6.97 (m, 2H), 6.86 (dd, *J* = 11.2, 7.3 Hz, 1H), 5.62 (s, 1H), 5.03 (dd, *J* = 12.4, 5.5 Hz, 1H), 4.45 (s, 2H), 4.34 (s, 2H), 3.89 – 3.81 (m, 2H), 3.70 (s, 2H), 3.60 (s, 2H), 2.90 – 2.62 (m, 3H), 2.54 (bs, 3H), 2.24 (t, *J* = 7.3 Hz, 2H), 2.13 – 2.03 (m, 1H), 1.97 – 1.87 (m, 2H), 1.82 – 1.71 (m, 2H), 1.71 – 1.60 (m, 4H), 1.43 (t, *J* = 4.9, 3.7 Hz, 6H), 1.36 – 1.27 (m, 1H), 0.93 (s, 6H). ^{13}C NMR (101 MHz, DMSO-*d_6*) δ 173.4, 172.7, 170.6, 169.4, 167.8, 167.2, 163.6, 162.0, 158.5, 157.8, 156.1, 146.9, 140.1, 136.8, 132.6, 128.7, 126.4, 126.1, 126.0, 117.7, 110.9, 109.4, 54.3, 53.1, 49.4, 49.0, 44.1, 42.4, 42.2, 38.4, 35.7, 35.0, 31.4, 28.9, 28.5, 26.4, 25.5, 22.6. LRMS (ESI): m/z 988.56 $[M + H]^+$, (calcd for $C_{58}H_{79}F_2N_{12}O_6^+$, 988.50).

Preparation of N-(3-(((6-(4-(4-((4,4-dimethylpiperidin-1-yl)methyl)-2,5-difluorophenyl)-2-oxo-1,4,9-triazaspiro[5.5]undecan-9-yl)pyrimidin-4-yl)amino)methyl)benzyl)-9-((2-(2,6-dioxopiperidin-3-yl)-1-oxoisoindolin-4-yl)amino)nonanamide (16)

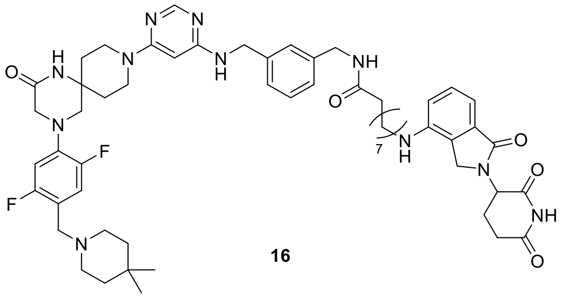

16

Compound **16** was prepared according to General procedure 3 (COMU) using the corresponding amine (25 mg, 0.04 mmol) and carboxylic acid (17 mg, 0.04 mmol). The reaction mixture was concentrated *in vacuo* and the crude product was purified using flash column chromatography (SiO_2; EtOAc/MeOH = from 90 : 10 to 80 : 20) providing 25 mg of the desired product (61% yield). ^{1}H NMR (400 MHz, DMSO-d_6) δ 11.01 (s, 1H), 8.35 (t, *J* = 6.0 Hz, 1H), 8.18 (s, 1H), 7.98 (s, 1H), 7.31 – 7.20 (m, 3H), 7.20 – 7.12 (m, 3H), 7.08 (d, *J* = 7.5 Hz, 1H), 6.97 – 6.86 (m, 2H), 6.71 (d, *J* = 8.0 Hz, 1H), 5.69 (s, 1H), 5.60 (t, *J* = 5.6 Hz, 1H), 5.10 (dd, *J* = 13.3, 5.1 Hz, 1H), 4.41 (d, *J* = 6.2 Hz, 2H), 4.28 – 4.09 (m, 4H), 3.83 (d, *J* = 13.3 Hz, 2H), 3.58 (s, 2H), 3.47 – 3.37 (m, 4H), 3.25 (s, 2H), 3.09 (q, *J* = 6.6 Hz, 2H), 2.92 (ddd, *J* = 18.1, 13.5, 5.4 Hz, 1H), 2.67 – 2.57 (m, 1H), 2.40 – 2.22 (m, 4H), 2.11 (t, *J* = 7.4 Hz, 2H), 2.06 – 1.97 (m, 1H), 1.81 – 1.72 (m, 2H), 1.69 – 1.60 (m, 1H), 1.60 – 1.44 (m, 4H), 1.38 – 1.20 (m, 14H), 0.87 (s, 6H). ^{13}C NMR (101 MHz, DMSO-d_6) δ 172.9, 172.1, 172.0, 171.3, 168.9, 166.5, 163.2, 161.6, 157.4, 156.2, 143.8, 139.8, 132.0, 129.2, 128.1, 126.5, 126.0, 125.5, 125.5, 111.7, 109.8, 106.3, 69.8, 62.8, 59.8, 54.7, 54.2, 52.8, 52.6, 51.5, 49.0, 45.8, 43.6, 42.7, 41.9, 38.1, 35.3, 34.6, 31.2, 28.9, 28.8, 28.7, 28.5, 28.1, 26.7, 25.3, 22.8, 21.1, 20.8, 14.1. LRMS (ESI): m/z 1016.566 $[M + H]^+$, (calcd for $C_{56}H_{72}F_2N_{11}O_5^+$, 1016.568).

Preparation of N-(3-(((6-(4-(4-((4,4-dimethylpiperidin-1-yl)methyl)-2,5-difluorophenyl)-2-oxo-1,4,9-triazaspiro[5.5]undecan-9-yl)pyrimidin-4-yl)amino)methyl)benzyl)-11-((2-(2,6-dioxopiperidin-3-yl)-1-oxoisoindolin-4-yl)amino)undecanamide (17)

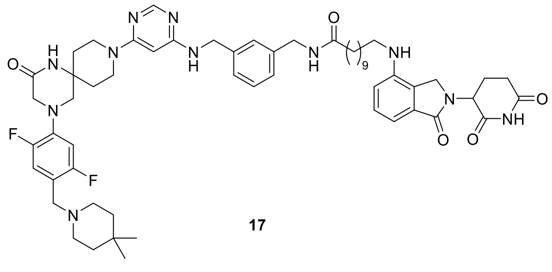

17

Compound **17** was prepared according to General procedure 3 (COMU) using the corresponding amine (18.6 mg, 0.04 mmol) and carboxylic acid (26 mg, 0.04 mmol). The reaction mixture was concentrated *in vacuo* and the crude product was purified using flash column chromatography (SiO_2; DCM/MeOH = from 90 : 10 to 80 : 20) providing 32 mg of the desired product (73% yield). ^{1}H NMR (400 MHz, MeOD-d_4) δ 8.06 (s, 1H), 7.47 – 7.38 (m, 1H), 7.33 – 7.25 (m, 3H), 7.22 (d, J = 7.8 Hz, 1H), 7.17 (d, J = 7.5 Hz, 1H), 7.03 (d, J = 7.5 Hz, 1H), 6.98 (dd, J = 11.4, 7.2 Hz, 1H), 6.79 (d, J = 8.0 Hz, 1H), 5.69 (s, 1H), 5.13 (dd, J = 13.3, 5.1 Hz, 1H), 4.48 (s, 2H), 4.34 (s, 2H), 4.30 – 4.26 (m, 3H), 3.94 – 3.86 (m, 2H), 3.76 (s, 2H), 3.72 (t, J = 6.7 Hz, 1H), 3.64 (s, 1H), 3.57 (dd, J = 10.8, 8.1 Hz, 2H), 3.49 – 3.43 (m, 1H), 3.41 (s, 2H), 3.26 – 3.16 (m, 6H), 2.96 – 2.84 (m, 1H), 2.81 – 2.73 (m, 1H), 2.46 (qd, J = 13.1, 4.7 Hz, 1H), 2.23 – 2.14 (m, 3H), 1.97 – 1.89 (m, 2H), 1.78 (m, 2H), 1.70 – 1.57 (m, 6H), 1.39 – 1.36 (m, 14H), 1.05 (s, 5H). ^{13}C NMR (101 MHz, MeOD-d_4) δ 176.2, 174.7, 172.5, 172.4, 169.8, 162.6, 160.9, 145.2, 142.5, 140.8, 140.1, 133.0, 130.6, 129.9, 128.0, 127.5, 127.3, 127.2, 121.3, 113.8, 111.8, 110.3, 107.6, 55.8, 54.4, 53.6, 53.3, 50.0, 47.5, 46.0, 44.4, 43.9, 43.8, 41.9, 37.1, 36.5, 35.7, 32.4, 30.8, 30.6, 30.5, 30.3, 30.2, 30.2, 28.7, 28.2, 27.0, 24.2, 18.7, 17.3, 13.2. LRMS (ESI): m/z 1044.600 [M + H]$^+$, (calcd for $C_{58}H_{76}F_2N_{11}O^+$, 1044.600).

Preparation of N-(3-(((6-(4-(4-((4,4-dimethylpiperidin-1-yl)methyl)-2,5-difluorophenyl)-2-oxo-1,4,9-triazaspiro[5.5]undecan-9-yl)pyrimidin-4-yl)amino)methyl)benzyl)-3-(4-(((2-(2,6-dioxopiperidin-3-yl)-1,3-dioxoisoindolin-4-yl)amino)methyl)-1H-1,2,3-triazol-1-yl)propenamide (18)

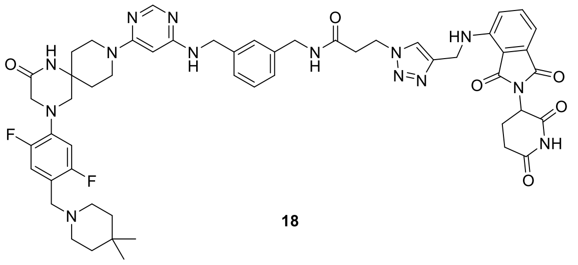

18

Compound **18** was prepared according to General procedure 3 (COMU) using the corresponding amine (48.5 mg, 0.07 mmol) and carboxylic acid (40 mg, 0.07 mmol). The reaction mixture was concentrated *in vacuo* and the crude product was purified using flash column chromatography (SiO_2; DCM/MeOH = from 90 : 10 to 80 : 20) providing 26 mg of the desired product (34% yield). ^{1}H NMR (600 MHz, MeOD-d_4) δ 7.96 (s, 1H), 7.80 (s, 1H), 7.47 (dd, *J* = 8.5, 7.1 Hz, 1H), 7.24 – 7.14 (m, 4H), 7.06 – 6.98 (m, 3H), 6.88 (dd, *J* = 11.2, 7.2 Hz, 1H), 5.61 (s, 1H), 5.03 (dd, *J* = 12.8, 5.5 Hz, 1H), 4.65 (t, *J* = 6.4 Hz, 2H), 4.56 (s, 2H), 4.42 (s, 2H), 4.24 (s, 2H), 3.85 – 3.78 (m, 2H), 3.71 (s, 2H), 3.68 (s, 2H), 3.34 (s, 2H), 2.83 (t, *J* = 6.4 Hz, 2H), 2.75 – 2.66 (m, 2H), 2.66 – 2.58 (m, 4H), 2.10 – 2.05 (m, 1H), 1.92 – 1.87 (m, 2H), 1.78 – 1.71 (m, 2H), 1.46 (t, *J* = 5.7 Hz, 4H), 1.31 – 1.28 (m, 6H), 0.95 (s, 6H). ^{13}C NMR (151 MHz, MeOD-d_4) δ 174.7, 171.9, 171.6, 170.5, 170.3, 169.2, 164.4, 163.4, 159.9, 158.3, 153.1, 151.5, 147.4, 146.4, 140.8, 140.4, 140.1, 137.2, 133.9, 129.7, 127.1, 127.1, 124.6, 120.2, 120.0, 118.3, 112.5, 111.9, 107.4, 107.2, 71.5, 61.6, 56.0, 55.1, 54.6, 53.7, 50.4, 50.2, 47.6, 45.7, 43.9, 41.7, 38.8, 38.7, 37.0, 35.7, 33.1, 32.2, 31.8, 30.8, 29.0, 23.8, 14.5. LRMS (ESI): m/z 1028.509 $[M + H]^+$, (calcd for $C_{53}H_{61}F_2N_{14}O_6^+$, 1028.159).

Preparation of N-(3-(((6-(4-(4-((4,4-dimethylpiperidin-1-yl)methyl)-2,5-difluorophenyl)-2-oxo-1,4,9-triazaspiro[5.5]undecan-9-yl)pyrimidin-4-yl)amino)methyl)benzyl)-9-(4-(((2-(2,6-dioxopiperidin-3-yl)-1,3-dioxoisoindolin-4-yl)amino)methyl)-1H-1,2,3-triazol-1-yl)nonanamide (19)

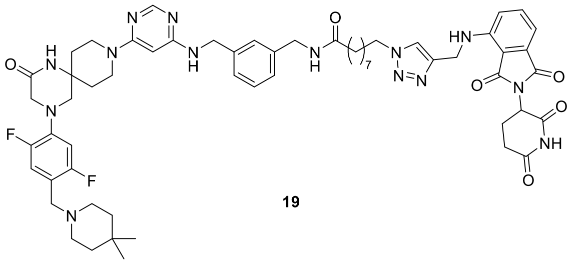

19

Compound **19** was prepared according to General procedure 3 (COMU) using the corresponding amine (18.2 mg, 0.03 mmol) and carboxylic acid (15 mg, 0.03 mmol). The reaction mixture was concentrated *in vacuo* and the crude product was purified using flash column chromatography (SiO_2; DCM/MeOH = from 90 : 10 to 80 : 20) providing 10 mg of desired product (31% yield). ^{1}H NMR (500 MHz, $CDCl_3$) δ 10.24 (bs, 1H), 8.16 (s, 1H), 7.51 – 7.41 (m, 2H), 7.30 – 7.26 (m, 1H), 7.20 (s, 1H), 7.17 (t, *J* = 7.4 Hz, 2H), 7.11 (d, *J* = 7.1 Hz, 1H), 6.98 (d, *J* = 8.5 Hz, 1H), 6.81 (s, 1H), 6.67 (t, *J* = 6.0 Hz, 1H), 6.58 (dd, *J* = 10.9, 7.0 Hz, 1H), 6.03 (t, *J* = 6.0 Hz, 1H), 5.37 (s, 1H), 4.89 (dd, *J* = 12.2, 5.4 Hz, 1H), 4.62 (d, *J* = 5.9 Hz, 2H), 4.47 – 4.35 (m, 3H), 4.31 (t, *J* = 6.9, 2.4 Hz, 2H), 3.71 (s, 2H), 3.70 – 3.62 (m, 2H), 3.54 – 3.45 (m, 2H), 3.28 (s, 2H), 2.90 – 2.69 (m, 3H), 2.56 (bs, 2H), 2.12 (t, *J* = 7.1, 6.5 Hz, 2H), 1.91 – 1.81 (m, 4H), 1.79 – 1.71 (m, 3H), 1.57 (t, *J* = 7.8, 7.1 Hz, 2H), 1.50 – 1.43 (m, 2H), 1.26 (m, 16H), 0.94 (s, 6H). ^{13}C NMR (126 MHz, $CDCl_3$) δ 173.2, 171.9, 169.6, 169.6, 167.8, 162.8, 162.5, 157.2, 156.6, 152.0, 150.1, 146.3, 145.2, 139.4, 138.7, 136.4, 132.5, 129.1, 126.9, 126.3, 126.0, 121.8, 118.9, 117.3, 112.4, 110.9, 105.8, 105.6, 81.1, 56.3, 53.6, 53.0, 50.5, 49.1, 45.7, 43.4, 40.6, 38.9, 36.7, 35.1, 32.1, 31.6, 30.1, 29.9, 28.9, 28.9, 28.5, 28.3, 26.2, 25.6, 23.0, 22.8, 14.3. LRMS (ESI): m/z 1111.581 $[M + H]^+$, (calcd for $C_{59}H_{73}F_2N_{14}O_6^+$, 1111.580).

Preparation of N-(3-(((6-(4-(4-((4,4-dimethylpiperidin-1-yl)methyl)-2,5-difluorophenyl)- 2-oxo-1,4,9-triazaspiro[5.5]undecan-9-yl)pyrimidin-4-yl)amino)methyl)benzyl)-11-(4-(((2-(2,6-dioxopiperidin-3-yl)-1,3-dioxoisoindolin-4-yl)amino)methyl)-1H-1,2,3-triazol-1-yl)undecanamide (20)

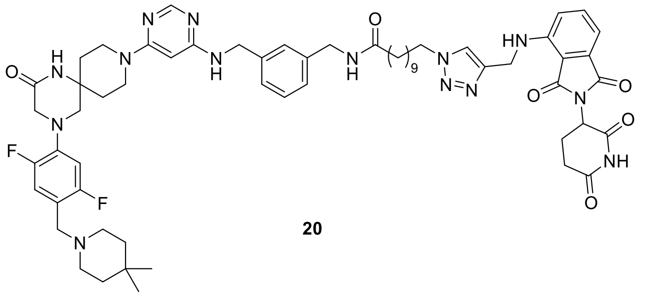

20

Compound **20** was prepared according to General procedure 3 (COMU) using the corresponding amine (17 mg, 0.03 mmol) and carboxylic acid (15 mg, 0.03 mmol). The reaction mixture was concentrated *in vacuo* and the crude product was purified using flash column chromatography (SiO_2; DCM/MeOH = from 90 : 10 to 80 : 20) providing 12 mg of desired product (37% yield). ^{1}H NMR (400 MHz, $CDCl_3$) δ 10.08 (bs, 1H), 8.16 (s, 1H), 7.50 – 7.43 (m, 2H), 7.21 (s, 1H), 7.17 (t, *J* = 7.3 Hz, 2H), 7.10 (d, *J* = 7.2 Hz, 1H), 6.97 (d, *J* = 8.5 Hz, 1H), 6.83 (s, 1H), 6.68 (t, *J* = 5.9 Hz, 1H), 6.58 (dd, *J* = 10.9, 7.1 Hz, 1H), 6.07 (s, 1H), 5.93 (bs, 1H), 5.40 – 5.32 (m, 2H), 4.90 (dd, *J* = 11.9, 5.3 Hz, 1H), 4.62 (d, *J* = 5.9 Hz, 2H), 4.42 (m, 3H), 4.31 (t, *J* = 7.0 Hz, 2H), 3.71 (s, 2H), 3.67 – 3.62 (m, 2H), 3.59 – 3.45 (m, 4H), 3.27 (s, 2H), 2.90 – 2.69 (m, 3H), 2.48 (bs, 2H), 2.18 – 2.13 (m, 2H), 2.05 – 1.97 (m, 2H), 1.93 – 1.83 (m, 4H), 1.80 – 1.71 (m, 2H), 1.65 – 1.55 (m, 4H), 1.44 – 1.39 (m, 4H), 1.28 – 1.25 (m, 12H), 0.92 (s, 6H). ^{13}C NMR (101 MHz, $CDCl_3$) δ 173.3, 171.9, 169.6, 169.4, 167.9, 167.7, 162.5, 146.3, 139.4, 138.7, 136.3, 132.5, 129.1, 126.8, 126.3, 126.0, 121.7, 117.3, 112.4, 110.9, 56.4, 53.6, 53.0, 50.6, 49.7, 49.1, 45.7, 43.4, 40.6, 38.9, 38.4, 36.7, 35.1, 32.1, 31.6, 30.2, 29.9, 29.5, 29.2, 29.1, 29.1, 28.8, 28.4, 27.4, 26.3, 25.8, 22.9, 22.8, 14.3. LRMS (ESI): m/z 1139.608 $[M + H]^+$, (calcd for $C_{61}H_{77}F_2N_{14}O_6^+$, 1139.610).

Preparation of 4-((4-(4-(((6-(4-(4-((4,4-dimethylpiperidin-1-yl)methyl)-2,5-difluorophenyl)-2-oxo-1,4,9-triazaspiro[5.5]undecan-9-yl)pyrimidin-4-yl)amino)methyl)piperidin-1-yl)-4-oxobutyl)amino)-2-(2,6-dioxopiperidin-3-yl)isoindoline-1,3-dione (22)

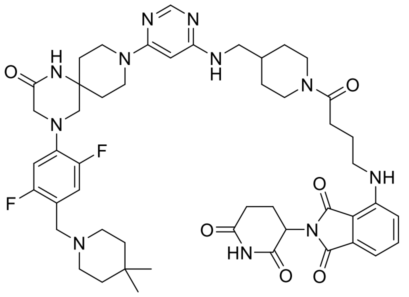

22

Compound **22** was prepared according to General procedure 3 (COMU) using the corresponding amine (53 mg, 0.08 mmol) and carboxylic acid (30 mg, 0.08 mmol). The reaction mixture was concentrated *in vacuo* and the crude product was purified using flash column chromatography (SiO_2; DCM/MeOH = from 95 : 5 to 90 : 10), followed by a second flash column chromatography (SiO_2; EtOAc/MeOH = from 90 : 10 to 80 : 20) providing 32 mg of the desired product (41% yield). ^{1}H NMR (400 MHz, MeOD-d_4) δ 7.99 (s, 1H), 7.55 (dd, J = 8.6, 7.1 Hz, 1H), 7.18 (dd, J = 12.9, 6.6 Hz, 1H), 7.10 (d, J = 8.6 Hz, 1H), 7.03 (d, J = 7.1 Hz, 1H), 6.88 (dd, J = 11.2, 7.3 Hz, 1H), 5.66 (s, 1H), 5.05 (dd, J = 12.4, 5.4 Hz, 1H), 4.53 (d, J = 13.1 Hz, 1H), 3.97 – 3.90 (m, 3H), 3.72 (s, 2H), 3.60 (s, 2H), 3.43 – 3.35 (m, 6H), 3.15 – 3.08 (m, 2H), 3.03 (t, J = 12.8 Hz, 1H), 2.91 – 2.79 (m, 1H), 2.78 – 2.67 (m, 2H), 2.63 – 2.43 (m, 7H), 2.14 – 2.06 (m, 1H), 2.02 – 1.91 (m, 4H), 1.88 – 1.72 (m, 5H), 1.43 (t, J = 5.6 Hz, 4H), 1.15 – 1.01 (m, 2H), 0.93 (s, 6H). ^{13}C NMR (101 MHz, MeOD-d_4) δ 174.7, 173.1, 171.7, 170.7, 170.3, 169.3, 164.8, 163.4, 158.3, 148.2, 137.3, 134.0, 120.1, 119.9, 118.1, 111.9, 111.2, 107.4, 107.1, 56.2, 55.3, 54.6, 53.7, 50.5, 50.2, 47.3, 46.9, 43.0, 42.8, 41.8, 39.0, 37.4, 35.9, 32.2, 31.6, 31.2, 31.0, 30.8, 29.1, 25.9, 23.8. LRMS (ESI): m/z 938.492 $[M + H]^+$, (calcd for $C_{49}H_{62}F_2N_{11}O_6^+$, 938.484).

Preparation of 4-((6-(4-(((6-(4-(4-((4,4-dimethylpiperidin-1-yl)methyl)-2,5-difluorophenyl)-2-oxo-1,4,9-triazaspiro[5.5]undecan-9-yl)pyrimidin-4-yl)amino)methyl)piperidin-1-yl)-6-oxohexyl)amino)-2-(2,6-dioxopiperidin-3-yl)isoindoline-1,3-dione (24)

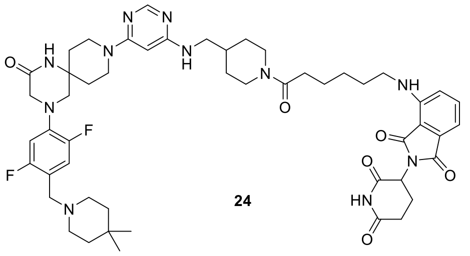

24

Compound **24** was prepared according to General procedure 3 (COMU) using the corresponding amine (26 mg, 0.042 mmol) and carboxylic acid (17 mg, 0.042 mmol). The reaction mixture was concentrated *in vacuo* and the crude product was purified using flash column chromatography (SiO_2; EtOAc/MeOH = 2 : 0.6) providing 32 mg of the desired product (79% yield). ^{1}H NMR (400 MHz, MeOD-d_4) δ 7.99 (s, 1H), 7.54 (dd, *J* = 8.5, 7.1 Hz, 1H), 7.22 (dd, *J* = 12.9, 6.7 Hz, 1H), 7.02 (d, *J* = 8.5 Hz, 1H), 6.92 (dd, *J* = 11.3, 7.2 Hz, 1H), 5.68 (s, 1H), 5.02 (dd, *J* = 13.1, 6.0 Hz, 1H), 4.52 (d, *J* = 13.3 Hz, 1H), 3.98 – 3.86 (m, 3H), 3.847 – 3.78 (bs, 2H), 3.74 (s, 2H), 3.42 – 3.38 (m, 4H), 3.34 (d, *J* = 5.6 Hz, 2H), 3.21 – 3.13 (m, 2H), 3.05 (t, *J* = 12.4 Hz, 1H), 2.81– 2.70 (m, 6H), 2.63 – 2.5744 (m, 1H), 2.44 – 2.38 (m, 2H), 2.11 – 2.06 (m, 1H), 1.98 – 1.95 (m, 2H), 1.84 – 1.78 (m, 5H), 1.71 – 1.62 (m, 5H), 1.52 – 1.49 (m, 6H), 1.20 – 1.06 (m, 2H), 0.97 (s, 6H). ^{13}C NMR (101 MHz, MeOD-d_4) δ 174.7, 173.7, 171.7, 170.8, 170.1, 169.3, 164.8, 163.4, 160.5, 158.3, 158.1, 151.0, 148.3, 137.3, 133.9, 120.2, 118.0, 111.8, 111.0, 107.4, 107.1, 56.0, 54.8, 54.6, 53.6, 50.4, 50.2, 47.3, 47.1, 43.2, 43.0, 41.7, 38.3, 37.5, 35.8, 34.0, 32.2, 31.7, 30.8, 30.0, 29.0, 27.6, 26.4, 23.8. LRMS (ESI): m/z 966.545 [M + H]$^+$, (calcd for $C_{51}H_{66}F_2N_{11}O_6^+$, 966.451).

Preparation of 4-((8-(4-(((6-(4-(4-((4,4-dimethylpiperidin-1-yl)methyl)-2,5-difluoro phenyl)-2-oxo-1,4,9-triazaspiro[5.5]undecan-9-yl)pyrimidin-4-yl)amino)methyl)piperidin-1-yl)-8-oxooctyl)amino)-2-(2,6-dioxopiperidin-3-yl)isoindoline-1,3-dione (25)

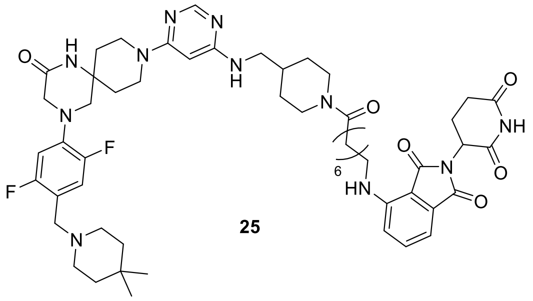

25

Compound **25** was prepared according to General procedure 3 (COMU) using the corresponding amine (50 mg, 0.08 mmol) and carboxylic acid (33 mg, 0.08 mmol). The reaction mixture was concentrated *in vacuo* and the crude product was purified using flash column chromatography (SiO_2; EtOAc/MeOH = from 85 : 15 to 80 : 20) providing 25 mg of the desired product (32% yield). ^{1}H NMR (400 MHz, MeOD-d_4) δ 7.99 (s, 1H), 7.53 (dd, *J* = 8.6, 7.1 Hz, 1H), 7.18 (dd, *J* = 13.0, 6.6 Hz, 1H), 7.03 (d, *J* = 8.6 Hz, 1H), 7.01 (d, *J* = 7.1 Hz, 1H), 6.88 (dd, *J* = 11.2, 7.2 Hz, 1H), 5.68 (s, 1H), 5.04 (dd, *J* = 12.5, 5.5 Hz, 1H), 4.53 (d, *J* = 13.2 Hz, 1H), 4.02 – 3.85 (m, 3H), 3.72 (s, 2H), 3.63 (m, 2H), 3.39 (m, 4H), 3.17 (d, *J* = 6.4 Hz, 2H), 3.05 (t, *J* = 12.8 Hz, 1H), 2.85 (ddd, *J* = 17.6, 14.2, 5.0 Hz, 1H), 2.76 (m, 1H), 2.74 – 2.68 (m, 1H), 2.58 (m, 5H), 2.38 (td, *J* = 7.4, 4.9 Hz, 2H), 2.10 (dtd, *J* = 12.9, 4.9, 2.3 Hz, 1H), 1.98 (m, 2H), 1.81 (m, 6H), 1.67 (t, *J* = 6.8 Hz, 2H), 1.60 (t, *J* = 7.1 Hz, 2H), 1.44 (t, *J* = 5.7 Hz, 6H), 1.38 (m, 5H), 1.32 – 1.28 (m, 2H), 0.94 (s, 6H). ^{13}C NMR (101 MHz, MeOD-d_4) δ 174.7, 174.0, 171.7, 170.8, 170.3, 169.3, 164.8, 163.4, 158.3, 148.3, 140.0, 137.3, 133.9, 119.9, 118.0, 111.7, 111.0, 107.4, 107.1, 56.2, 55.2, 54.6, 53.7, 50.5, 50.2, 47.3, 47.1, 43.4, 43.0, 41.7, 38.9, 37.6, 35.9, 34.1, 32.2, 31.7, 30.9, 30.2, 30.2, 30.1, 29.1, 27.8, 26.5, 23.8. LRMS (ESI): m/z 994.549 $[M + H]^+$, (calcd for $C_{53}H_{70}F_2N_{11}O_6^+$, 994.550).

Preparation of N-(3-(((6-(4-(4-((4,4-dimethylpiperidin-1-yl)methyl)-2,5-difluorophenyl)- 2-oxo-1,4,9-triazaspiro[5.5]undecan-9-yl)pyrimidin-4-yl)4-(2-aminoethyl)piperazin)-4-((2-(2,6-dioxopiperidin-3-yl)-1,3-dioxoisoindolin-4-yl)amino)butanamide (29)

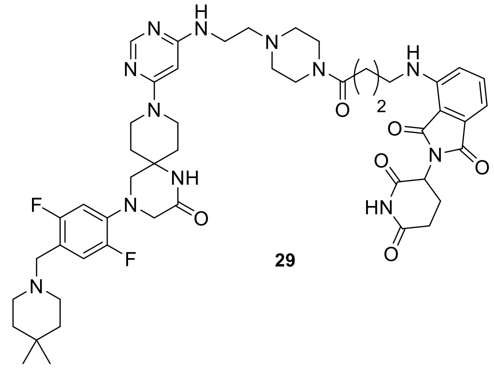

29

Compound **29** was prepared according to General procedure 3 (HATU) using the corresponding amine (74 mg, 0.11 mmol) and carboxylic acid (40 mg, 0.11 mmol). The reaction mixture was concentrated *in vacuo* and the crude product was purified using flash column chromatography (SiO_2; EtOAc/MeOH = 90 : 10) providing 61 mg of the desired product (59% yield). ^{1}H NMR (500 MHz, MeOD-d_4) δ 8.57 (s, 1H), 8.03 (s, 1H), 7.57 (dd, *J* = 8.4, 7.2 Hz, 1H), 7.22 (dd, *J* =12.9, 6.7 Hz, 1H), 7.12 (d, *J* = 8.5, 1H), 7.06 (d, *J* = 7.0, 1H), 6.92 (dd, *J* =11.1, 7.3 Hz, 1H),

5.73 (s, 1H), 5.07 (dd, *J* =12.5, 5.4 Hz, 1H), 3.96 (m, 2H), 3.76 (s, 2H), 3.71 (s, 2H), 3.66-3.53 (m, 4H), 3.50-3.37 (m, 9H), 2.91-2.61 (m, 8H), 2.59 (t, *J* = 6.2, 2H), 2.53 (t, *J* = 6.8, 1H), 2.48 (m, 4H), 2.12 (m, 1H), 1.99 (m, 4H), 1.84 (m, 2H), 1.49 (t, *J* = 5.6, 4H), 1.21 (s, 3H), 0.97 (s, 6H). ^{13}C NMR (101 MHz, MeOD-*d$_4$*) δ 173.2, 171.8, 170.2, 169.3, 168.8, 167.8, 163.0, 162.1, 158.9, 156.8, 156.5, 152.1, 149.7, 148.5, 146.7, 138.9, 138.8, 135.8, 132.5, 118.7, 118.5, 116.7, 110.5, 109.8, 106.0, 105.7, 81.23, 56.2, 54.7, 53.7, 53.1, 52.7, 52.3, 52.3, 49.0, 48.8, 45.2, 41.3, 41.3, 40.3, 37.6, 37.3, 34.4, 30.8, 29.47, 27.6, 24.4, 22.4. LRMS (ESI): m/z 953.584 [M + H]$^+$, (calcd for $C_{49}H_{63}F_2N_{12}O_6^+$, 953.469).

Preparation of N-(3-(((6-(4-(4-((4,4-dimethylpiperidin-1-yl)methyl)-2,5-difluorophenyl)- 2-oxo-1,4,9-triazaspiro[5.5]undecan-9-yl)pyrimidin-4-yl)4-(2-aminoethyl)piperazin)-6-((2-(2,6-dioxopiperidin-3-yl)-1,3-dioxoisoindolin-4-yl)amino)hexanamide (30)

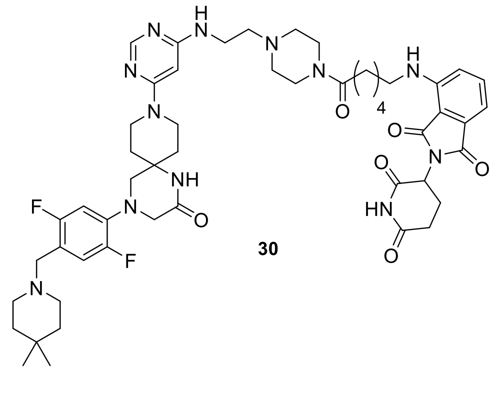

30

Compound **30** was prepared according to General procedure 3 (HATU), the corresponding amine (74 mg, 0.11 mmol) and carboxylic acid (42 mg, 0.11 mmol). The reaction mixture was concentrated *in vacuo* and the crude product was purified using flash column chromatography (SiO_2; EtOAc/MeOH = 90 : 10) providing 53 mg of desired product (50% yield). ^{1}H NMR (500 MHz, MeOD-*d$_4$*) δ 8.58 (s, 1H), 8.02 (s, 1H), 7.92 (s, 1H), 7.56 (dd, *J* = 8.2, 7.3 Hz, 1H), 7.20 (dd, *J* =12.9, 6.5 Hz, 1H), 7.06 (dd, *J* =10.4, 8.7 Hz, 2H), 6.90 (dd, *J* =11.1, 7.2 Hz, 1H), 5.74 (s, 1H), 5.06 (dd, *J* =12.4, 5.4 Hz, 1H), 3.97 (m, 2H), 3.75 (s, 2H), 3.66-3.54 (m, 6H), 3.49-3.36 (m, 8H), 2.91-2.67 (m, 3H), 2.60 (t, *J* =6.1, 2H); 2.59-2.46 (m, 7H), 2.44 (t, *J* =7.0, 2H), 2.12 (m, 1H), 2.01 (m, 2H), 1.84 (m, 2H), 1.70 (m, 4H), 1.54-1.42 (m, 6H), 1.31 (s, 3H), 0.93 (s, 6H). ^{13}C NMR (101 MHz, MeOD-*d$_4$*) δ 173.2, 172.5, 170.2, 169.4, 168.8, 167.9, 163.0, 162.1, 158.8, 156.8, 156.4, 146.9, 138.6, 135.8, 132.5, 118.6, 118.4, 116.6, 110.3, 109.6, 105.9, 105.7, 78.0, 56.2, 54.7, 53.8, 53.1, 52.9, 52.4, 52.3, 49.1, 48.7, 45.4, 41.7, 41.3, 40.3, 37.6, 37.6, 34.4, 32.3, 30.8, 29.3, 28.5, 27.6, 26.1, 24.8, 22.4. LRMS (ESI): m/z 981.584 [M + H]$^+$, (calcd for $C_{51}H_{66}F_2N_{12}O_6^+$, 980.520).

Preparation of N-(3-(((6-(4-(4-((4,4-dimethylpiperidin-1-yl)methyl)-2,5-difluorophenyl)- 2-oxo-1,4,9-triazaspiro[5.5]undecan-9-yl)pyrimidin-4-yl)4-(2-aminoethyl)piperazin)-8-((2-(2,6-dioxopiperidin-3-yl)-1,3-dioxoisoindolin-4-yl)amino)octanamide (31)

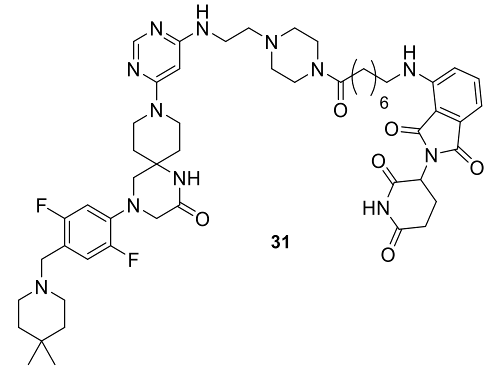

31

Compound **31** was prepared according to General procedure 3 (HATU) using the corresponding amine (74 mg, 0.11 mmol) and carboxylic acid (45 mg, 0.11 mmol). The reaction mixture was concentrated *in vacuo* and the crude product was purified using flash column chromatography (SiO_2; EtOAc/MeOH = 90 : 10) providing 73 mg of desired product (66% yield). ^{1}H NMR (500 MHz, DMSO-d_6) δ 8.56 (s, 1H), 8.03 (s, 1H), 7.56 (dd, *J* = 8.3, 7.0 Hz, 1H), 7.27 (dd, *J* =12.6, 6.4 Hz, 1H), 7.05 (dd, *J* = 8.5, 6.3 Hz, 2H), 6.95 (dd, *J* =11.1, 7.2 Hz, 1H), 5.74 (s, 1H), 5.07 (dd, *J* =12.6, 5.5 Hz, 1H), 3.95 (m, 2H), 3.90 (s, 2H), 3.77 (s, 2H), 3.70-3.55 (m, 4H), 3.50-3.39 (m, 8H), 2.89-2.71 (m, 6H), 2.66 (s, 1H), 2.63 (t, *J* = 6.2, 2H), 2.53 (m, 4H), 2.40 (t, *J* = 7.4, 1H), 2.13 (m, 1H), 2.00 (m, 2H), 1.85 (m, 2H), 1.70 (m, 2H), 1.62 (m, 2H), 1.55 (t, *J* = 5.3, 4H), 1.50-1.37 (m, 9H), 1.31 (s, 6H), 1.01 (s, 6H). ^{13}C NMR (125 MHz, DMSO-d_6) δ 173.2, 172.7, 170.2, 169.4, 168.7, 167.9, 163.0, 162.1, 158.8, 156.8, 156.5, 151.8, 146.9, 135.8, 132.5, 119.0, 116.6, 110.3, 109.6, 106.0, 105.8, 56.5, 54.6, 53.3, 53.1, 52.9, 52.4, 52.2, 48.9, 48.8, 45.4, 41.9, 41.2, 40.2, 39.0, 37.6, 36.7, 34.4, 32.5, 30.8, 29.3, 28.7, 27.5, 26.3, 25.0, 22.4. LRMS (ESI): m/z 1009.712 $[M + H]^+$, (calcd for $C_{53}H_{71}F_2N_{12}O_6^+$, 1009.559).

Preparation of N-(3-(((6-(4-(4-((4,4-dimethylpiperidin-1-yl)methyl)-2,5-difluorophenyl)- 2-oxo-1,4,9-triazaspiro[5.5]undecan-9-yl)pyrimidin-4-yl)4-(2-aminoethyl)piperazin)4-(5-bromopyrimidin-2-yl)piperazine-4-((2-(2,6-dioxopiperidin-3-yl)-1,3-dioxoisoindolin-4-yl)amino)butanamide (33)

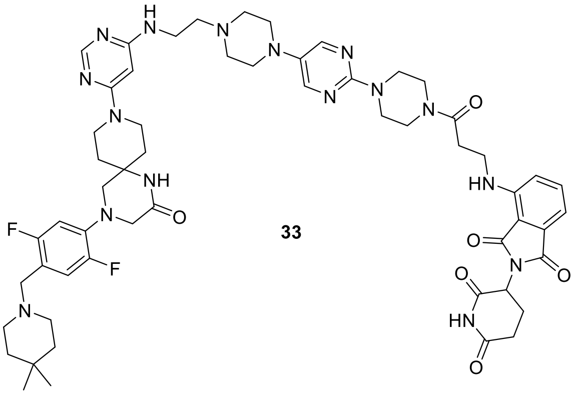

33

Compound **33** was prepared according to General procedure 3 (HATU) using the corresponding deprotected amine (31 mg, 0.04 mmol) and carboxylic acid (14 mg, 0.04 mmol). The reaction mixture was concentrated *in vacuo* and the crude product was purified using flash column chromatography (SiO_2; EtOAc/MeOH = 90 : 10) providing 23 mg of the desired product (53% yield). ^{1}H NMR (400 MHz, $CDCl_3$) δ 8.13 (s, 3H), 7.63-7.47 (m, 2H), 7.09 (d, *J* = 7.0 Hz, 1H), 6.99 (d, *J* = 8.5 Hz, 1H), 6.61 (dd, *J* =11.3, 7.1 Hz, 1H), 6.47 (s, 1H), 6.32 (t, *J* = 5.6 Hz, 1H), 5.51 (s, 1H), 4.90 (dd, *J* =12.2, 5.5 Hz, 1H), 3.95 (s, 2H), 3.82-3.61 (m, 9H), 3.57-3.49 (m, 2H), 3.46-3.32 (m, 4H), 3.11 (m, 3H), 2.98-2.93 (m, 4H), 2.82-2.68 (m, 6H), 2.49 (t, *J* = 6.6 Hz, 1H), 2.39-1.89 (m, 18H), 1.88-1.77 (m, 3H), 1.68 (s, 4H), 1.01 (s, 6H). ^{13}C NMR (176 MHz, DMSO-d_6) δ 172.8, 170.4, 170.2, 170.1, 168.8, 167.3, 166.4, 166.5, 163.2, 157.5, 157.3, 155.9, 151.2, 147.0, 146.4, 138.0, 136.3, 132.2, 117.8, 117.3, 110.4, 109.1, 106.5, 59.8, 54.9, 54.8, 54.1, 53.0, 52.8, 52.7, 52.5, 49.2, 49.1, 48.6, 48.5, 44.8, 41.6, 41.1, 40.0, 38.3, 34.6, 31.0, 29.4, 29.0, 28.1, 24.2, 22.1. LRMS (ESI): m/z 1115.654 $[M + H]^+$, (calcd for $C_{57}H_{73}F_2N_{16}O_6^+$, 1115.582).

Preparation of N-(3-(((6-(4-(4-((4,4-dimethylpiperidin-1-yl)methyl)-2,5-difluorophenyl)-2-oxo-1,4,9-triazaspiro[5.5]undecan-9-yl)pyrimidin-4-yl)amino)methyl)benzyl)-4-((2-(1-methyl-2,6-dioxopiperidin-3-yl)-1,3-dioxoisoindolin-4-yl)amino)butanamide (me-14)

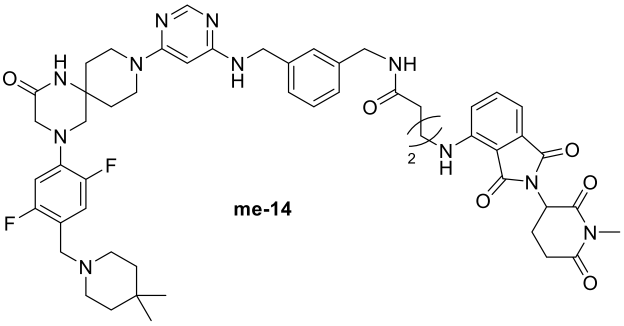

me-14

Compound **me-14** was prepared according to General procedure 3 (COMU) using the corresponding amine (56 mg, 0.09 mmol) and carboxylic acid (40 mg, 0.1 mmol). The reaction mixture was concentrated *in vacuo* and the crude product was purified using flash column chromatography (SiO_2; EtOAc/MeOH = 90 : 10) providing 43 mg of desired product (41% yield).^{1}H NMR (400 MHz, MeOD-d_4) δ 7.98 (s, 1H), 7.49 (dd, J = 8.6, 7.1 Hz, 1H), 7.30 – 7.13 (m, 6H), 7.03 – 6.96 (m, 2H), 6.90 (dd, J = 11.3, 7.2 Hz, 1H), 5.61 (s, 1H), 5.06 (dd, J = 12.8, 5.4 Hz, 1H), 4.45 (s, 2H), 4.34 (s, 2H), 3.86 – 3.79 (m, 4H), 3.72 (s, 2H), 3.39 – 3.35 (m, 4H), 3.11 (s, 3H), 2.86 (dd, J = 7.1, 3.7 Hz, 2H), 2.76 (bs, 4H), 2.72 – 2.60 (m, 1H), 2.33 (t, J = 7.2 Hz, 2H), 2.10 – 2.03 (m, 1H), 1.97 – 1.86 (m, 5H), 1.79 – 1.69 (m, 2H), 1.50 (d, J = 7.2 Hz, 5H), 0.97 (s, 6H). ^{13}C NMR (101 MHz, MeOD-d_4) δ 175.1, 173.7, 171.4, 170.7, 170.1, 169.3, 164.5, 163.4, 163.0, 158.3, 148.1, 140.6, 137.3, 133.9, 129.8, 127.4, 127.1, 127.1, 119.9, 118.0, 111.9, 111.1, 107.2, 54.6, 53.6, 50.8, 50.4, 49.6, 49.5, 49.4, 49.3, 49.2, 49.1, 49.0, 48.9, 48.8, 48.6, 48.4, 44.0, 42.8, 41.7, 38.3, 35.7, 34.1, 32.5, 30.8, 29.0, 27.3, 26.4, 23.0. LRMS (ESI): m/z 974.485 $[M + H]^+$, (calcd for $C_{52}H_{62}F_2N_{11}O_6^+$, 974.485).

Preparation of 4-((6-(4-(((6-(4-(4-((4,4-dimethylpiperidin-1-yl)methyl)-2,5-difluorophenyl)-2-oxo-1,4,9-triazaspiro[5.5]undecan-9-yl)pyrimidin-4-yl)amino)methyl)piperidin-1-yl)-6-oxohexyl)amino)-2-(1-methyl-2,6-dioxopiperidin-3-yl)isoindoline-1,3-dione (me-24)

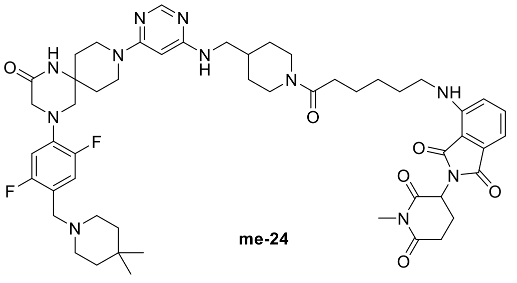

me-24

Compound **me-24** was prepared according to General procedure 3 (COMU) using the corresponding amine (25 mg, 0.040 mmol) and carboxylic acid (17 mg, 0.04 mmol). The reaction mixture was concentrated *in vacuo* and the crude product was purified using flash column chromatography (SiO_2; DCM/MeOH = from 100:5 to 100:10) providing 6 mg of desired product (15% yield). ^{1}H NMR (400 MHz, $CDCl_3$) δ 8.14 (s, 1H), 7.48 (t, *J* = 7.8 Hz, 1H), 7.17 – 7.13 (m, 1H), 7.07 (d, *J* = 7.1 Hz, 1H), 6.87 (d, *J* = 8.5 Hz, 1H), 6.59 (dd, *J* = 10.9, 7.0 Hz, 1H), 6.38 (s, 1H), 6.23 – 6.21 (m, 1H), 5.44 (s, 1H), 4.93 – 4.89 (m, 1H), 4.66 (d, *J* = 13.3 Hz, 1H), 3.88 (d, *J* = 13.0 Hz, 1H), 3.76 – 3.51 (m, 4H), 3.31 – 3.13 (m, 6H), 3.04 – 2.95 (m, 2H), 2.78 – 2.73 (m, 2H), 2.55 – 2.42 (m, 4H), 2.34 (t, *J* = 7.5 Hz, 2H), 2.09 – 2.07 (m, 1H), 2.01 – 1.92 (m, 2H), 1.87 – 1.77 (m, 4H), 1.72 – 1.62 (m, 4H), 1.50 – 1.39 (m, 6H), 1.18 – 1.10 (m, 2H), 0.92 (s, 6H), 0.86 – 0.77 (m, 2H). ^{13}C NMR (101 MHz, $CDCl_3$) δ 171.4, 171.2, 169.9, 169.2, 167.9, 167.8, 163.3, 163.1, 162.5, 157.9, 147.1, 136.3, 132.7, 118.8, 116.7, 111.5, 110.1, 105.9, 56.6, 54.7, 53.6, 53.1, 49.8, 49.8, 47.1, 45.6, 42.6, 41.7, 40.4, 38.4, 36.6, 35.5, 33.2, 32.1, 30.8, 29.9, 29.8, 29.3, 28.4, 27.4, 26.9, 25.1, 22.3. LRMS (ESI): m/z 980.531 $[M + H]^+$, (calcd for $C_{52}H_{68}F_2N_{11}O_6^+$, 980.532).

Preparation of N-(3-(((6-(4-(4-((4,4-dimethylpiperidin-1-yl)methyl)-2,5-difluorophenyl)- 2-oxo-1,4,9-triazaspiro[5.5]undecan-9-yl)pyrimidin-4-yl)4-(2-aminoethyl)piperazin)-(2-(2,6-dioxopiperidin-3-yl)-1,3-dioxoisoindolin-4-yl)dione (32)

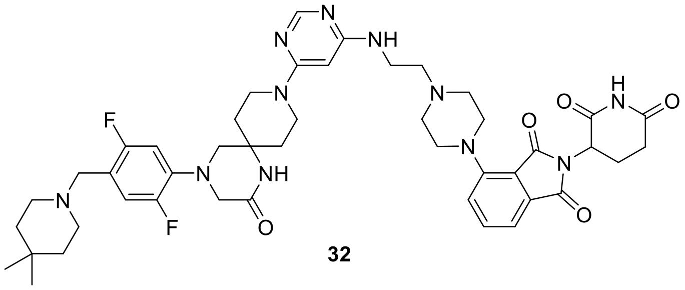

32

Compound **32** was prepared according to General procedure 1 using compounds **57** (86 mg, 0.22 mmol), **39** (75 mg, 0.15 mmol) and DIPEA (0.6 mmol). The reaction mixture was concentrated *in vacuo* and the crude product was purified using flash column chromatography (SiO_2; EtOAc/MeOH = 90 : 10) providing 57 mg of the desired product (44% yield). ^{1}H NMR (400 MHz, $CDCl_3$) δ 8.17 (s, 1H), 7.5 (t, *J* = 7.7 Hz, 1H), 7.38 (d, *J* = 7.0 Hz, 1H), 7.21 - 7.09 (m, 4H), 6.96 (s, 1H), 6.58 (dd, *J* =10.7, 7.3 Hz, 1H), 5.55 (s, 1H), 5.45 (s, 1H), 4.95 (m, 1H), 3.72 (m, 4H), 3.64 (m, 2H), 3.52 (m, 2H), 3.34 (m, 6H), 3.28 (m, 2H), 2.91-2.65 (m, 9H), 2.45 (s, 4H), 2.09 (m, 1H), 1.89 (m, 4H), 1.41 (s, 4H), 0.91 (s, 6H).^{13}C NMR (101 MHz, $CDCl_3$) δ 171.4, 168.7, 167.9, 167.3, 166.7, 163.0, 162.4, 158.5, 157.7, 156.1, 152.1, 150.2, 149.7, 138.0,

135.6, 134.1, 123.3, 118.8, 118.6, 117.4, 115.7, 105.7, 105.4, 80.7, 56.5, 56.3, 54.3, 53.4, 52.9, 52.7, 50.9, 49.6, 40.1, 38.0, 35.3, 31.4, 28.2, 22.7. LRMS (ESI): m/z 868.554 $[M + H]^+$, (calcd for $C_{45}H_{55}F_2N_{11}O_5^+$, 868.439).

Preparation of 5-((4-(4-(((6-(4-(4-((4,4-dimethylpiperidin-1-yl)methyl)-2,5-difluorophenyl)-2-oxo-1,4,9-triazaspiro[5.5]undecan-9-yl)pyrimidin-4-yl)amino)methyl)piperidin-1-yl)-4-oxobutyl)amino)-2-(2,6-dioxopiperidin-3-yl)isoindoline-1,3-dione (23)

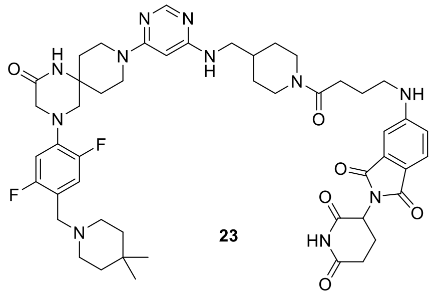

23

The reaction sequence started with General procedure 6 using 5-fluoro-thalidomide (813 mg, 2.95 mmol) and the corresponding *tert*-butyl ester (469 mg, 2.95 mmol). The volatiles were then removed *in vacuo* and the crude product was used in the next synthetic step without further purification. To a stirred solution of the *tert*-butyl ester (1 eq) in DCM (0.5 M) was added TFA (10 eq). The resulting reaction mixture was stirred at rt until full completion. The free carboxylic acid was used in the next step without further purification. (Monitored by TLC). Compound **23** was prepared according to General procedure 3 (COMU) using the corresponding amine (79 mg, 0.125 mmol) and carboxylic acid (44 mg, 0.125 mmol). The reaction mixture was concentrated *in vacuo* and the crude product was purified using flash column chromatography (SiO_2; DCM/MeOH = 2 : 0.1 to 2 : 0.2), followed by a second flash column chromatography (SiO_2; EtOAc/MeOH = 2 : 0.4) providing 7 mg of the desired product (0.2% yield after three steps). ^{1}H NMR (400 MHz, MeOD-d_4) δ 7.99 – 7.96 (bs, 1H), 7.56 (d, J = 8.4 Hz, 1H), 7.21 (dd, J = 12.9, 6.6 Hz, 1H), 6.98 (d, J = 2.2 Hz, 1H), 6.91 (dd, J = 11.3, 7.2 Hz, 1H), 6.86 (dd, J = 8.4, 2.2 Hz, 1H), 5.67 (s, 1H), 5.03 (dd, J = 12.8, 5.5 Hz, 1H), 4.60 (s, 4H), 4.54 (d, J = 13.7 Hz, 1H), 3.97 – 3.90 (m, 4H), 3.74 – 3.72 (m, 4H), 3.48 – 3.43 (m, 4H), 3.30 – 3.26 (m, 1H), 3.15 – 3.12 (m, 2H), 3.06 – 3.01 (m, 1H), 2.88 – 2.80 (m, 2H), 2.75 – 2.59 (m, 8H), 2.55 – 2.46 (m, 2H), 2.08 (ddt, J = 13.0, 5.6, 2.8 Hz, 1H), 2.00 – 1.91 (m, 3H), 1.86 – 1.79 (m, 3H), 1.50 – 1.46 (m, 4H), 1.14 – 1.06 (m, 2H), 0.96 (s, 6H). ^{13}C NMR (151 MHz, MeOD-d_4) δ 174.7, 173.2, 171.8, 170.2, 169.6, 169.3, 164.9, 164.7, 163.2, 160.0, 158.4, 158.3, 156.1, 153.1, 151.8, 140.6, 136.0, 126.6, 120.2, 118.2, 116.8, 107.4, 107.2, 106.7, 56.1, 55.0, 54.6, 53.6, 50.4, 50.3, 49.6,

47.3, 46.9, 43.4, 43.0, 41.7, 38.5, 37.5, 40.0, 35.8, 32.2, 31.6, 31.3, 30.8, 29.0, 25.6, 23.8. LRMS (ESI): m/z 938.495 $[M + H]^+$, (calcd for $C_{49}H_{62}F_2N_{11}O_6^+$, 938.485).

Preparation of 4-((2-(4-(((6-(4-(4-((4,4-dimethylpiperidin-1-yl)methyl)-2,5-difluorophenyl)-2-oxo-1,4,9-triazaspiro[5.5]undecan-9-yl)pyrimidin-4-yl)amino)methyl)piperidin-1-yl)-2-oxoethyl)amino)-2-(2,6-dioxopiperidin-3-yl)isoindoline-1,3-dione (21)

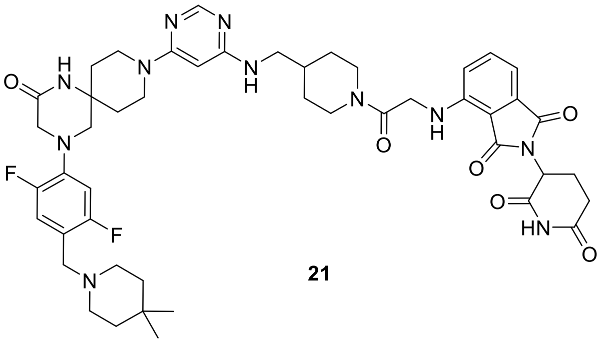

21

Compound **21** was prepared according to General procedure 6 with 4-fluoro-thalidomide (14 mg, 0.052 mmol, 1 eq) and the corresponding amine (40 mg, 0.052 mmol, 1 eq). The reaction was stirred at 80°C for 24 hours, evaporated and the crude product was purified using flash column chromatography (SiO_2; DCM/MeOH = from 90 : 10 to 87 : 13), followed by a second flash column chromatography (SiO_2; EtOAc/MeOH = from 90 : 10 to 80 : 20) providing 23 mg of the desired product (48% yield). ^{1}H NMR (400 MHz, DMSO-d_6) δ 11.09 (s, 1H), 8.17 (s, 1H), 7.98 (s, 1H), 7.60 (t, J = 7.8 Hz, 1H), 7.19 – 7.08 (m, 3H), 7.06 (d, J = 7.0 Hz, 1H), 6.93 (dd, J = 11.5, 7.4 Hz, 1H), 6.78 (bs, 1H), 5.66 (s, 1H), 5.06 (dd, J = 12.9, 5.4 Hz, 1H), 4.38 (d, J = 12.8 Hz, 1H), 4.16 (dd, J = 9.5, 4.5 Hz, 1H), 3.86 (d, J = 15.9 Hz, 3H), 3.59 (s, 2H), 3.43 (s, 2H), 3.26 (s, 3H), 3.18 – 3.08 (m, 2H), 3.00 (t, J = 12.4 Hz, 1H), 2.95 – 2.83 (m, 1H), 2.69 – 2.56 (m, 2H), 2.34 (s, 4H), 2.08 – 1.97 (m, 1H), 1.85 – 1.60 (m, 9H), 1.22 – 1.08 (m, 5H), 1.07 – 0.96 (m, 2H), 0.87 (s, 6H). ^{13}C NMR (126 MHz, DMSO-d_6) δ 172.9, 170.1, 168.8, 167.4, 166.6, 166.0, 163.5, 158.0, 157.8, 157.3, 151.3, 149.4, 145.4, 136.2, 132.0, 118.5, 118.3, 116.2, 110.8, 109.5, 106.5, 106.5, 106.3, 106.3, 69.8, 54.7, 52.8, 52.7, 49.0, 48.6, 45.5, 43.7, 41.6, 40.4, 37.9, 35.7, 35.5, 34.6, 31.0, 29.9, 29.3, 29.1, 29.0, 28.1, 22.2, 14.0. LRMS (ESI): m/z 910.454 $[M + H]^+$, (calcd for $C_{47}H_{57}F_2N_{11}O_6^+$, 910.453).

Preparation of 4-(4-(4-(((6-(4-(4-((4,4-dimethylpiperidin-1-yl)methyl)-2,5-difluorophenyl)-2-oxo-1,4,9-triazaspiro[5.5]undecan-9-yl)pyrimidin-4-yl)amino)methyl)phenyl)piperazin-1-yl)-2-(2,6-dioxopiperidin-3-yl)isoindoline-1,3-dione (34)

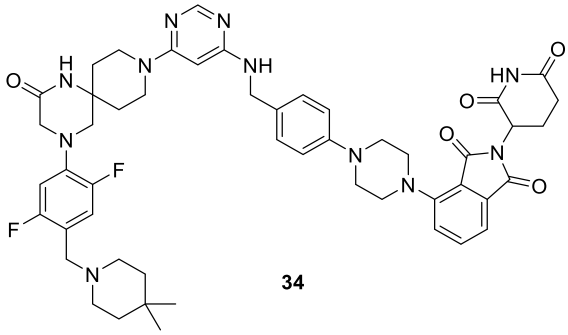

34

To a stirred solution of compound **47a** (150 mg, 0.193 mmol) in DCM (1.9 mL) was added TFA (0.147 mL, 1.9 mmol). The reaction mixture was stirred at rt for two hours. The volatiles were removed *in vacuo* and compound **47** was used in the next step without further purification. The final compound **34** was prepared using General procedure 6 using **47** (57 mg, 0.086 mmol) and 4-fluoro thalidomide (0.024 g, 0.083 mmol). The crude product was purified was purified using flash column chromatography (SiO_2; DCM/MeOH = from 100 : 5 to 100 : 10) followed by preparative TLC (EtOAc/MeOH 2 : 0.3) providing 5 mg of desired product (0.01% yield). ^{1}H NMR (400 MHz, $CDCl_3$) δ 8.16 (s, 1H), 7.63 (dd, *J* = 8.4, 7.2 Hz, 1H), 7.44 (d, *J* = 7.3 Hz, 1H), 7.27 – 7.25 (m, 1H), 7.22 (dd, *J* = 8.4 Hz, 2H), 6.96 (d, *J* = 8.7 Hz, 2H), 6.60 (dd, *J* = 10.9, 7.0 Hz, 1H), 6.24 (s, 1H), 5.43 (s, 1H), 4.97 (dd, *J* = 12.4, 5.4 Hz, 1H), 4.38 (d, *J* = 5.6 Hz, 2H), 3.74 (s, 2H), 3.69 – 3.63 (m, 2H), 3.60 – 3.55 (m, 2H), 3.53 – 3.49 (m, 3H), 3.39 (t, *J* = 5.1 Hz, 3H), 2.91 – 2.70 (m, 4H), 2.14 – 2.12 (m, 1H), 1.96 – 1.92 (m, 2H), 1.80 – 1.76 (m, 2H), 0.96 (s, 6H), 0.89 – 0.83 (m, 5H). ^{13}C NMR (151 MHz, $CDCl_3$) δ 171.2, 168.4, 167.4, 162.4, 150.8, 150.4, 150.2, 135.9, 134.3, 129.0, 128.5, 123.5, 117.9, 116.8, 116.2, 105.6, 81.0, 56.4, 53.6, 52.9, 51.2, 49.6, 49.3, 45.5, 41.1, 40.4, 37.2, 35.4, 32.1, 31.6, 29.9, 29.8, 29.5, 28.2, 27.4, 22.9, 14.3, 1.2. LRMS (ESI): m/z 930.461 $[M + H]^+$, (calcd for $C_{50}H_{58}F_2N_{11}O_5^+$, 930.486).

Preparation of 4-(4-(2-(4-(((6-(4-(4-((4,4-dimethylpiperidin-1-yl)methyl)-2,5-difluorophenyl)-2-oxo-1,4,9-triazaspiro[5.5]undecan-9-yl)pyrimidin-4-yl)amino)methyl)piperidin-1-yl)-2-oxoethyl)piperazin-1-yl)-2-(2,6-dioxopiperidin-3-yl)isoindoline-1,3-dione (35)

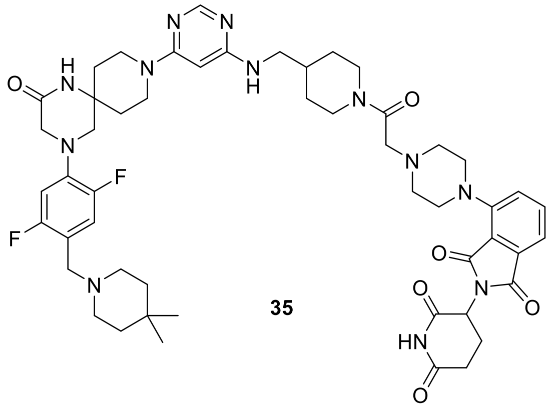

Compound **50** (61 mg, 0.09 mmol, 1 eq) and *tert*-butyl piperazine-1-carboxylate (25 mg, 0.135 mmol, 1.5 eq) were dissolved in 1 mL of DMSO. TEA (3 eq) was added to the mixture and the reaction was stirred at 50°C overnight. The volatiles were then removed *in vacuo* and the crude product was used in the next synthetic step without further purification. To a stirred solution of the Boc-protected amine (1 eq) in DCM (0.5 M) was added TFA (10 eq). The resulting reaction mixture was stirred at rt until full completion. The free amine **51** was used in the next step without further purification (Monitored by TLC). Compound **35** was prepared according to General procedure 6 using **51**, 4-fluoro thalidomide (9 mg, 0.09 mmol, 1 eq) and DIPEA (3 eq). The reaction mixture was stirred at 110 °C for 1 hour. The volatiles were then removed *in vacuo* and the mixture was purified with flash column chromatography twice (SiO_2; DMC/MeOH = 90:10m, then EtOAc/MeOH = 2:1). Affording 5 mg of desired product (5% yield after three steps). ^{1}H NMR (400 MHz, MeOD-*d4*) δ 7.98 (s, 1H), 7.66 (t, *J* = 7.8 Hz, 1H), 7.43 (s, 1H), 7.37 (d, *J* = 7.2 Hz, 1H), 7.30 (d, *J* = 8.4 Hz, 1H), 7.18 (dd, *J* = 13.0, 6.6 Hz, 1H), 6.87 (dd, *J* = 11.2, 7.2 Hz, 1H), 5.69 (s, 1H), 5.09 (dd, *J* = 12.5, 5.4 Hz, 1H), 4.55 – 4.50 (m, , 1H), 4.18 (d, *J* = 11.5 Hz, 1H), 3.91 – 3.88 (m, 2H), 3.71 (s, 2H), 3.60 (s, 2H), 3.44 – 3.33 (m, 8H), 3.21 – 3.17 (m, 3H), 3.07 (t, *J* = 12.6 Hz, 1H), 2.91 – 2.63 (m, 8H), 2.57 – 2.47 (m, 4H), 2.13 – 2.09 (m, 1H), 1.97 – 1.76 (m, 7H), 1.21 – 1.11 (m, 2H), 0.93 (s, 6H). ^{13}C NMR (126 MHz, MeOD-*d4*) δ 174.6, 171.6, 170.2, 169.9, 168.9, 168.0, 164.9, 163.4, 160.1, 158.3, 158.2, 153.3, 151.5, 136.9, 135.5, 130.9, 130.2, 130.2, 129.9, 124.7, 120.2, 119.9, 118.8, 116.3, 107.4, 107.2, 61.7, 56.1, 55.1, 54.6, 54.2, 53.7, 51.9, 50.5, 47.2, 47.0, 43.3, 41.7, 38.7, 37.7, 36.5, 35.8, 33.1, 32.2, 31.8, 30.9, 30.8, 30.3, 29.0, 23.7, 14.4. LRMS (ESI): m/z 979.512 $[M + H]^+$, (calcd for $C_{51}H_{65}F_2N_{12}O_6^+$, 979.511).

Preparation of 9-(4-(((6-(4-(4-((4,4-dimethylpiperidin-1-yl)methyl)-2,5-difluorophenyl)-2-oxo-1,4,9-triazaspiro[5.5]undecan-9-yl)pyrimidin-4-yl)amino)methyl)-1H-1,2,3-triazol-1-yl)-N-(2-(2,6-dioxopiperidin-3-yl)-1-oxoisoindolin-4-yl)nonanamide (26)

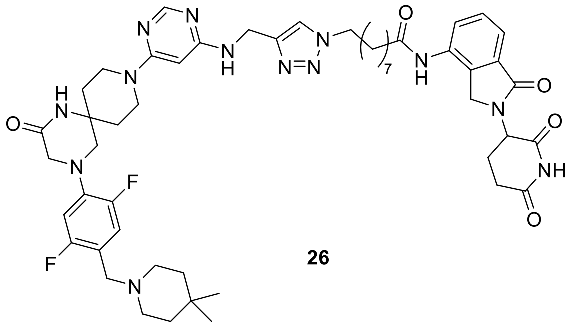

26

Intermediate **54** was prepared according to General procedure 9 starting from **52** (40 mg, 83 µmol, 1 eq) and sodium azide (6.5 mg, 100 µmol, 1.2 eq). The desired product was obtained in quantitative yield and used as such without further purification (37 mg).

20 mg of compound **54** (0.065 mmol, 1 eq) were dissolved in 1 mL of THF, followed by the addition of **40** (25 mg, 0.065 mmol, 1 eq), anhydrous $CuSO_4$ (4 mg, 0.5 eq) and sodium ascorbate (14 mg, 1.1 eq). The resulting reaction mixture was stirred at 70°C (oil bath temperature) for 48h. The volatiles were then removed *in vacuo* and the crude product was purified using flash column chromatography (SiO_2; DCM/MeOH = 90 : 10). 27 mg of the resulting product were dissolved in DCM and stirred overnight with 500 mg of SiliaMetS® TAAcONa. The mixture was then filtered and washed with DCM. The solution was evaporated affording 20 mg of desired product (45% yield). ^{1}H NMR (400 MHz, DMSO-*d6*) δ 11.02 (s, 1H), 9.76 (s, 1H), 8.18 (s, 1H), 8.02 (s, 1H), 7.91 (s, 1H), 7.81 (dd, *J* = 7.1, 1.9 Hz, 1H), 7.53 – 7.43 (m, 3H), 7.22 – 7.11 (m, 1H), 7.08 (t, *J* = 5.9 Hz, 1H), 6.93 (dd, *J* = 11.5, 7.3 Hz, 1H), 5.71 (s, 1H), 5.14 (dd, *J* = 13.3, 5.1 Hz, 1H), 4.45 (d, *J* = 5.8 Hz, 2H), 4.35 (dd, *J* = 27.0, 17.5 Hz, 2H), 4.29 (t, *J* = 7.1 Hz, 2H), 3.84 (d, *J* = 13.5 Hz, 2H), 3.59 (s, 2H), 3.44 (s, 2H), 3.31 (s, 2H), 3.25 (s, 2H), 2.92 (ddd, *J* = 17.2, 13.5, 5.3 Hz, 1H), 2.67 – 2.58 (m, 1H), 2.41 – 2.27 (m, 6H), 2.08 – 1.96 (m, 1H), 1.81 – 1.72 (m, 4H), 1.69 – 1.53 (m, 4H), 1.36 – 1.25 (m, 10H), 0.87 (s, 6H). ^{13}C NMR (101 MHz, DMSO-*d6)* δ 172.9, 171.4, 171.1, 167.8, 166.5, 163.0, 161.6, 157.4, 155.7, 145.3, 133.8, 133.67, 132.7, 128.6, 125.2, 122.6, 119.0, 106.5, 103.4, 69.8, 54.8, 52.8, 52.6, 51.5, 49.2, 49.04, 46.5, 35.8, 34.6, 31.2, 29.8, 28.6, 28.6, 28.3, 28.1, 25.8, 25.0, 22.6. LRMS (ESI): m/z 978.528 [M + H]$^+$, (calcd for $C_{51}H_{66}F_2N_{13}O_5^+$, 978.527).

Preparation of 11-(4-(((6-(4-(4-((4,4-dimethylpiperidin-1-yl)methyl)-2,5-difluorophenyl)-2-oxo-1,4,9-triazaspiro[5.5]undecan-9-yl)pyrimidin-4-yl)amino)methyl)-1H-1,2,3-triazol-1-yl)-N-(2-(2,6-dioxopiperidin-3-yl)-1-oxoisoindolin-4-yl)undecanamide (27)

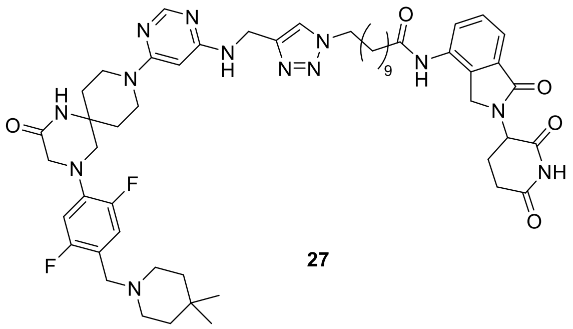

27

Compound **55** was prepared according to General procedure 9 starting from **53** (40 mg, 79 µmol, 1 eq) and sodium azide (6.2 mg, 95 µmol, 1.2 eq). The desired product was obtained in quantitative yield and used as such without further purification (37 mg).

30 mg of compound **58** (0.065 mmol, 1 eq) were dissolved in 1 mL of THF, followed by the addition of **40** (35 mg, 0.065 mmol, 1 eq), anhydrous $CuSO_4$ (5 mg, 0.5 eq) and sodium ascorbate (20 mg, 1.1 eq). The resulting reaction mixture was stirred at 40°C (oil bath temperature) for 48h. The volatiles were then removed *in vacuo* and the crude product was purified using flash column chromatography (SiO_2; DCM/MeOH = 90 : 10). 20 mg of the resulting product were dissolved in 2 mL of DCM and stirred overnight with 400 mg of SiliaMetS® TAAcONa. The mixture was then filtered and washed with DCM. The solution was evaporated affording 16 mg of desired product (25% yield). ^{1}H NMR (400 MHz, MeOD-*d*$_4$) δ 8.02 (d, *J* = 0.8 Hz, 1H), 7.85 (s, 1H), 7.70 (dd, *J* = 8.0, 1.0 Hz, 1H), 7.63 (d, *J* = 7.6 Hz, 1H), 7.50 (t, *J* = 7.7 Hz, 1H), 7.21 (dd, *J* = 12.9, 6.7 Hz, 1H), 6.90 (dd, *J* = 11.3, 7.3 Hz, 1H), 5.74 (d, *J* = 1.0 Hz, 1H), 5.16 (dd, *J* = 13.3, 5.2 Hz, 1H), 4.54 (s, 2H), 4.47 (d, *J* = 2.3 Hz, 2H), 4.35 (t, *J* = 6.9 Hz, 2H), 3.94 – 3.85 (m, 2H), 3.76 (s, 2H), 3.73 (s, 2H), 3.47 – 3.33 (m, 2H), 2.91 (ddd, *J* = 18.3, 13.4, 5.3 Hz, 1H), 2.83 – 2.72 (m, 1H), 2.70 (s, 4H), 2.42 (t, *J* = 7.5 Hz, 2H), 2.24 – 2.13 (m, 1H), 1.99 – 1.90 (m, 2H), 1.91 – 1.73 (m, 4H), 1.70 (p, *J* = 7.4 Hz, 2H), 1.48 (t, *J* = 5.7 Hz, 4H), 1.29 (q, *J* = 4.8 Hz, 13H), 0.96 (s, 6H). ^{13}C NMR (101 MHz, MeOD-*d*$_4$) δ 174.7, 174.6, 172.1, 171.1, 170.1, 164.3, 163.5, 158.4, 146.9, 136.3, 134.7, 134.0, 130.1, 127.8, 124.1, 121.4, 107.5, 107.3, 56.0, 54.9, 54.6, 53.6, 51.3, 50.44, 41.7, 38.4, 37.5, 37.3, 35.8, 32.4, 31.2, 31.0, 30.8, 30.3, 30.3, 30.3, 30.2, 29.9, 29.0, 28.1, 27.31, 26.7, 24.2, 23.7. LRMS (ESI): m/z 1006.558 [M + H]$^+$, (calcd for $C_{53}H_{70}F_2N_{13}O_5^+$, 1006.559).

Preparation of 4-(((1-(12-((6-(4-(4-((4,4-dimethylpiperidin-1-yl)methyl)-2,5-difluorophenyl)-2-oxo-1,4,9-triazaspiro[5.5]undecan-9-yl)pyrimidin-4-yl)amino)dodecyl)-1H-1,2,3-triazol-4-yl)methyl)amino)-2-(2,6-dioxopiperidin-3-yl)isoindoline-1,3-dione (28)

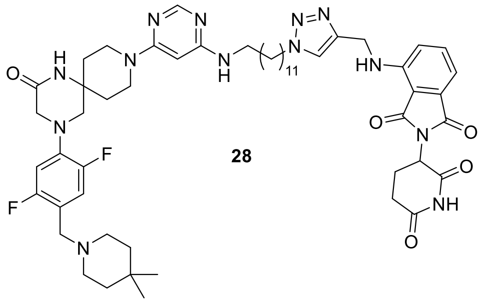

28

For the synthesis of **28**, intermediate **41** was prepared following General procedure 1 using compound **39** (37 mg, 0.073 mmol) and 12-azidododecan-1-amine (35 mg, 0.15 mmol, 2 eq). The volatiles were removed *in vacuo* and the crude product was purified using flash column chromatography (SiO_2; DCM/MeOH = 90 : 10) providing 20 mg of compound **41**. 18 mg of compound **41** (0.025 mmol, 1 eq) were dissolved in 1 mL of THF, followed by the addition of **56** (16 mg, 0.05 mmol, 2 eq), anhydrous $CuSO_4$ (4 mg) and sodium ascorbate (20 mg, 1.1 eq). The resulting reaction mixture was stirred at 40°C (oil bath temperature) for 48h. The volatiles were then removed *in vacuo* and the crude product was purified using flash column chromatography (SiO_2; EtOAc/MeOH = from 90:10 to 80:20), followed by preparative TLC (DCM/MeOH 90 : 10). 14 mg of the resulting product were dissolved in and stirred overnight with 200 mg of SiliaMetS® TAAcONa. The mixture was then filtered and washed with DCM. The yellow solution was evaporated affording 10 mg of the desired product (13% yield after 2 steps). ^{1}H NMR (400 MHz, DMSO-d_6) δ 11.10 (s, 1H), 8.20 (bs, 1H), 8.00 (s, 1H), 7.97 (s, 1H), 7.56 (dd, J = 8.6, 7.1 Hz, 1H), 7.19 (bs, 1H), 7.15 (d, J = 8.6 Hz, 1H), 7.10 – 7.02 (m, 2H), 6.96 (bs, 1H), 6.61 (t, J = 5.6 Hz, 1H), 5.60 (s, 1H), 5.06 (dd, J = 12.9, 5.3 Hz, 1H), 4.58 (d, J = 6.0 Hz, 2H), 4.29 (t, J = 7.0 Hz, 2H), 3.89 – 3.81 (m, 2H), 3.61 (s, 2H), 3.28 (s, 2H), 3.20 – 3.09 (m, 2H), 2.95 – 2.81 (m, 1H), 2.56 (s, 3H), 2.07 – 1.96 (m, 2H), 1.82 – 1.70 (m, 4H), 1.70 – 1.59 (m, 3H), 1.50 – 1.41 (m, 4H), 1.38 – 1.29 (m, 4H), 1.27 – 1.06 (m, 19H), 0.89 (s, 6H). ^{13}C NMR (101 MHz, DMSO-d_6) δ 172.8, 170.1, 168.8, 167.3, 163.3, 145.8, 144.4, 136.1, 132.1, 122.7, 117.6, 110.9, 109.7, 69.8, 52.6, 49.3, 48.6, 37.7, 34.6, 31.3, 31.0, 29.7, 29.0, 29.0, 28.9, 28.9, 28.8, 28.3, 26.5, 25.8, 22.2, 14.0. LRMS (ESI): m/z 1020.575 $[M + H]^+$, (calcd for $C_{54}H_{72}F_2N_{13}O_5^+$, 1020.574).

References

(1) Dolbois, A.; Bedi, R. K.; Bochenkova, E.; Müller, A.; Moroz-Omori, E. V; Huang, D.; Caflisch, A. 1,4,9-Triazaspiro[5.5]Undecan-2-One Derivatives as Potent and Selective METTL3 Inhibitors. *J Med Chem* **2021**, *64* (17), 12738–12760. DOI: 10.1021/acs.jmedchem.1c00773

(2) Hwang, J. Y.; Ha, J. D.; Cho, S. Y.; Kim, P.; Yun, C. S.; Kim, H. J.; Park, S. G.; Park, B. C.; Kim, J. H.; Kim, S. Target Protein EED Degradation-Inducing Degraducer, Preparation Method Thereof, and Pharmaceutical Composition for Preventing or Treating Diseases Related to EED, EZH2, or PRC2, Comprising Same as Active Ingredient. WO 2020 162725 A1, 2020.

(3) Sicheri, F.; Posternak, G.; Poda, G. Amide-Based Proteolysis Modulators of B-Rapidly Accelerated Fibrosarcoma (BRAF) and Associated Uses. WO 2020 176983 A1, 2020.

(4) Gray, N.; Bradner, J.; Tan, L.; Huang H.-T.; Buckley, D.; Winter, G.; Ishoey M. Degradation of Protein Kinases by Conjugation of Protein Kinase Inhibitors with E3 Ligase Ligand and Methods of Use. WO 2018 089736 A1, 2018.

^{1}H and ^{13}C NMR spectra of target compounds ***1-35****,* ***me-14*** *and* ***me-24***

N-(3-((6-(4-(4-((4,4-dimethylpiperidin-1-yl)methyl)phenyl)-2-oxo-1,4,9-triazaspiro[5.5]undecan-9-yl)pyrimidin-4-yl)amino)propyl)-3-(2-(2-((2-(2,6-dioxopiperidin-3-yl)-1,3-dioxoisoindolin-4-yl)amino)ethoxy)ethoxy)propenamide (1)

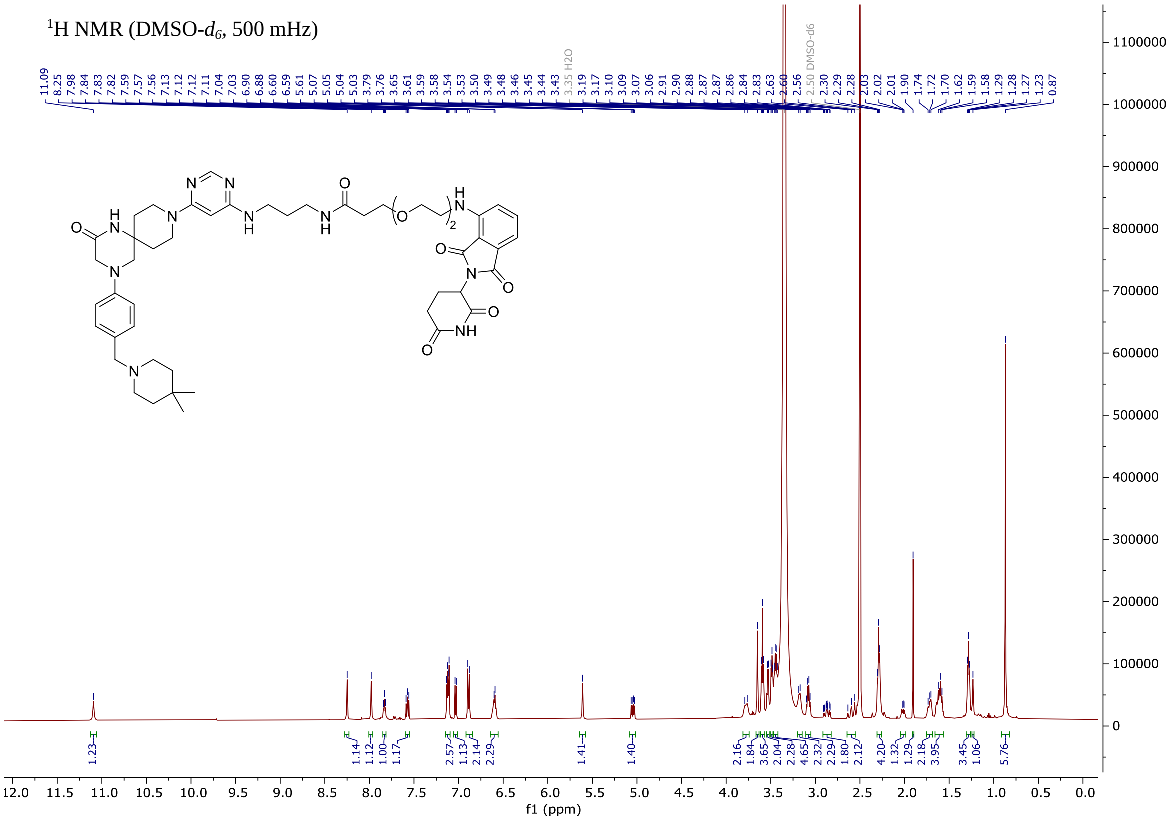

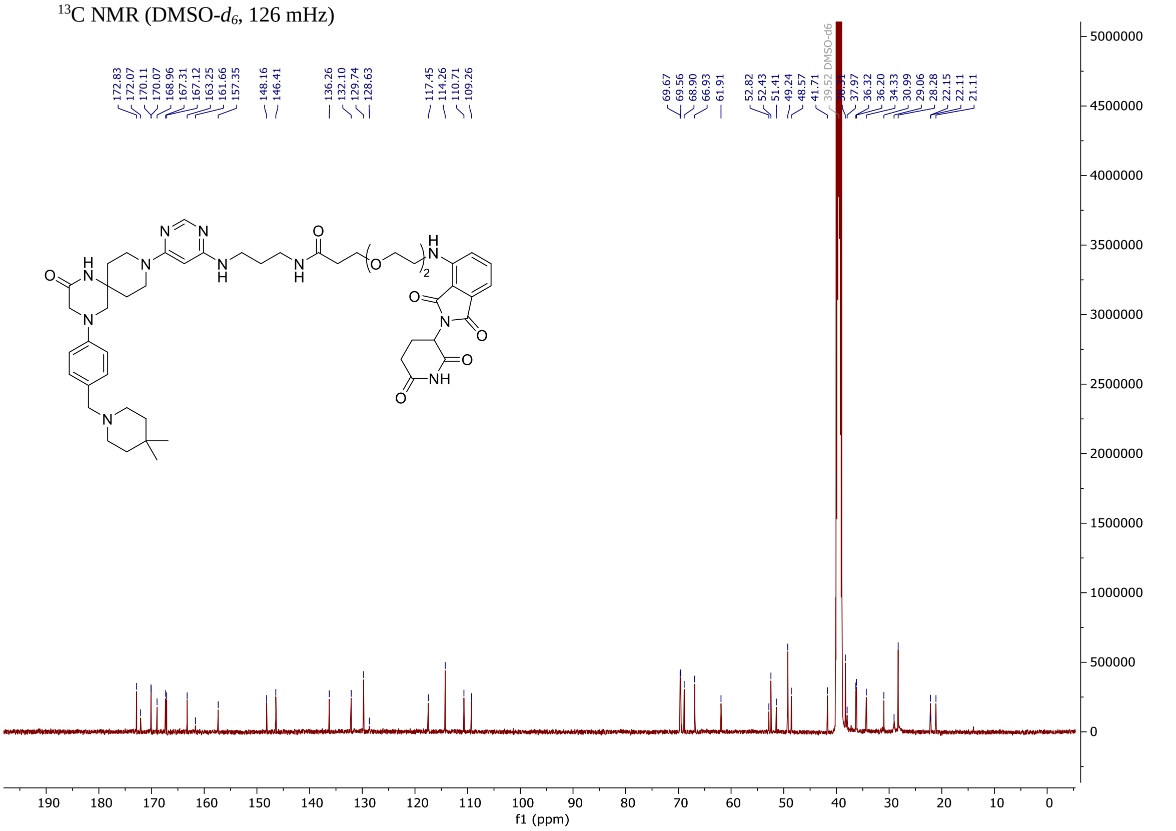

N-(3-((6-(4-(4-((4,4-dimethylpiperidin-1-yl)methyl)phenyl)- 2-oxo-1,4,9-triazaspiro[5.5]undecan-9-yl)pyrimidin-4-yl)amino)propyl)-3-(2-(2-(2-((2-(2,6-dioxopiperidin-3-yl)-1,3-dioxoisoindolin-4-yl)amino)ethoxy)ethoxy)ethoxy)propenamide (2)

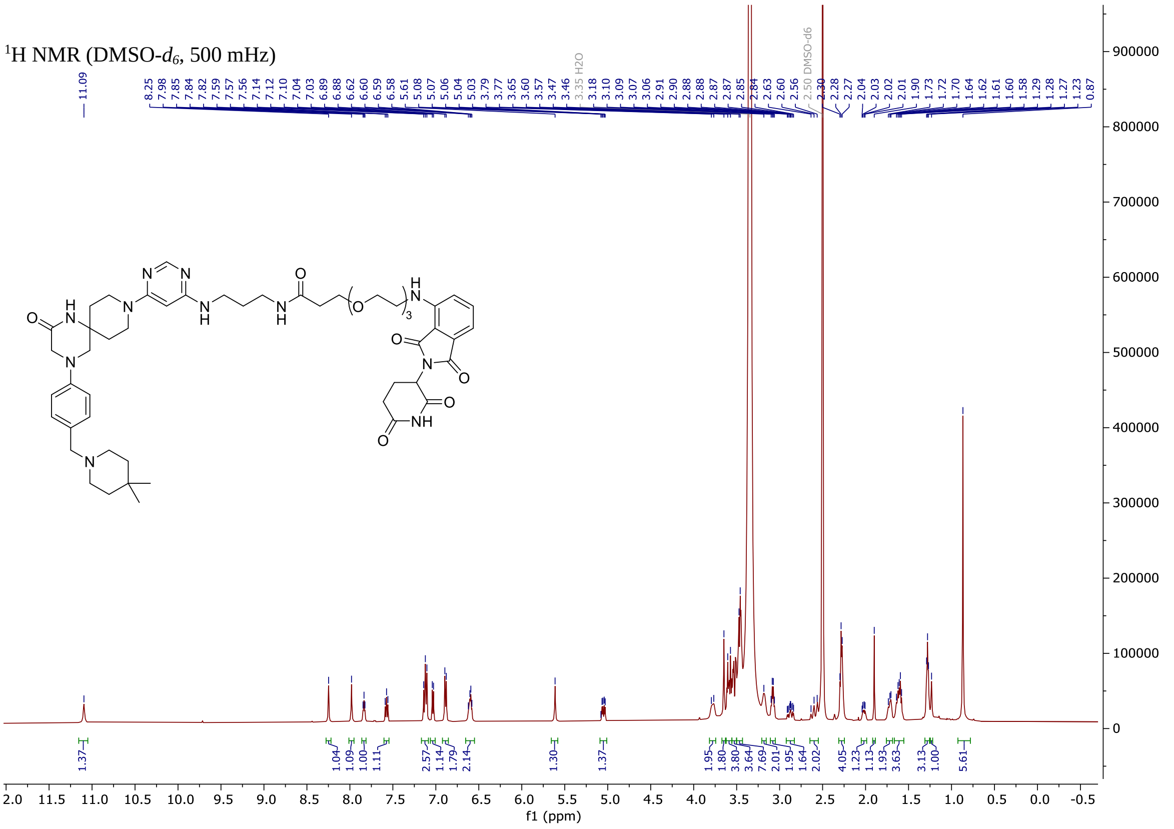

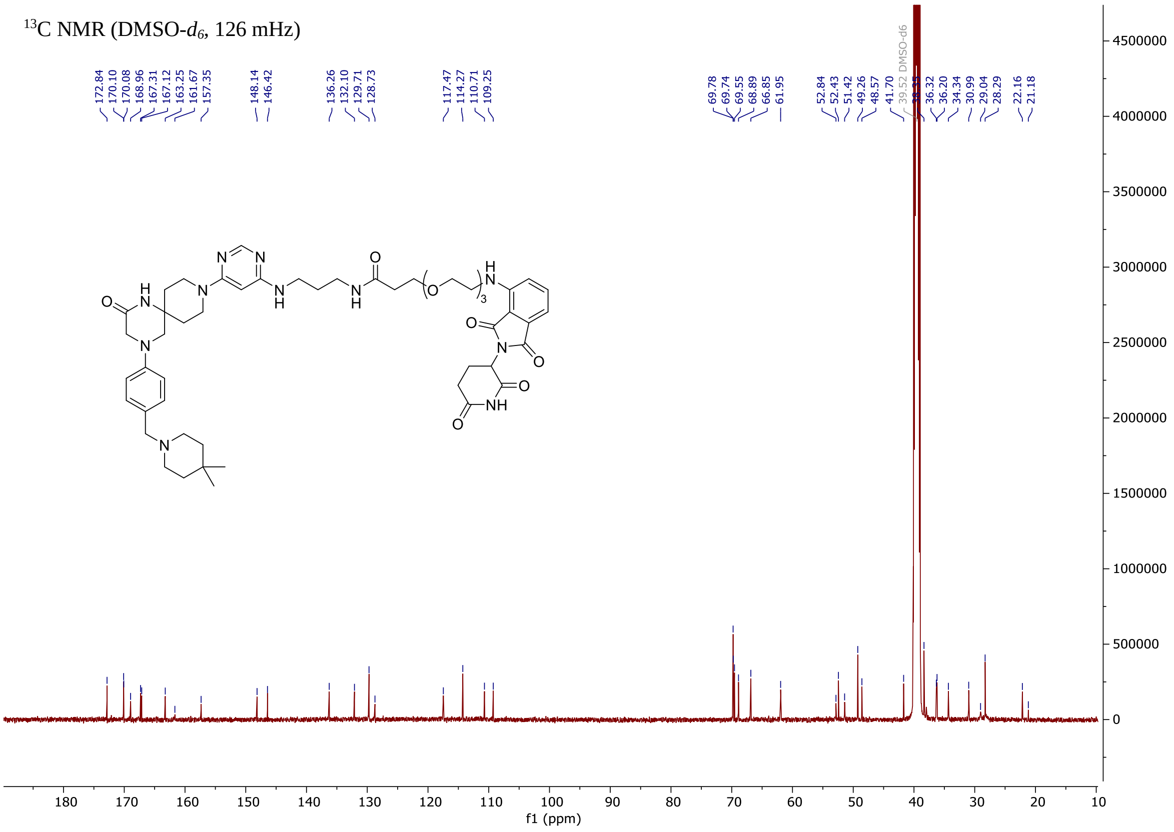

N-(3-((6-(4-(4-((4,4-dimethylpiperidin-1-yl)methyl)phenyl)- 2-oxo-1,4,9-triazaspiro[5.5]undecan-9-yl)pyrimidin-4-yl)amino)propyl)-1-((2-(2,6-dioxopiperidin-3-yl)-1,3-dioxoisoindolin-4-yl)amino)-3,6,9,12-tetraoxapentadecan-15-amide (3)

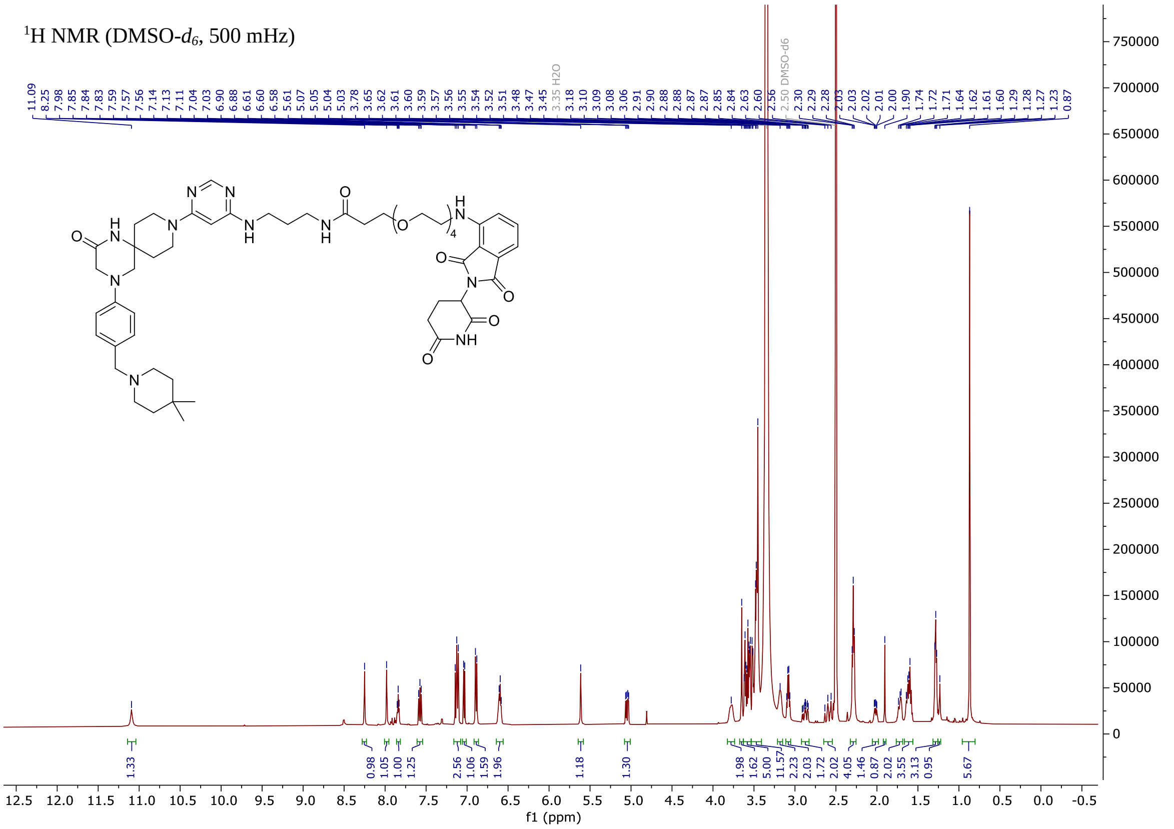

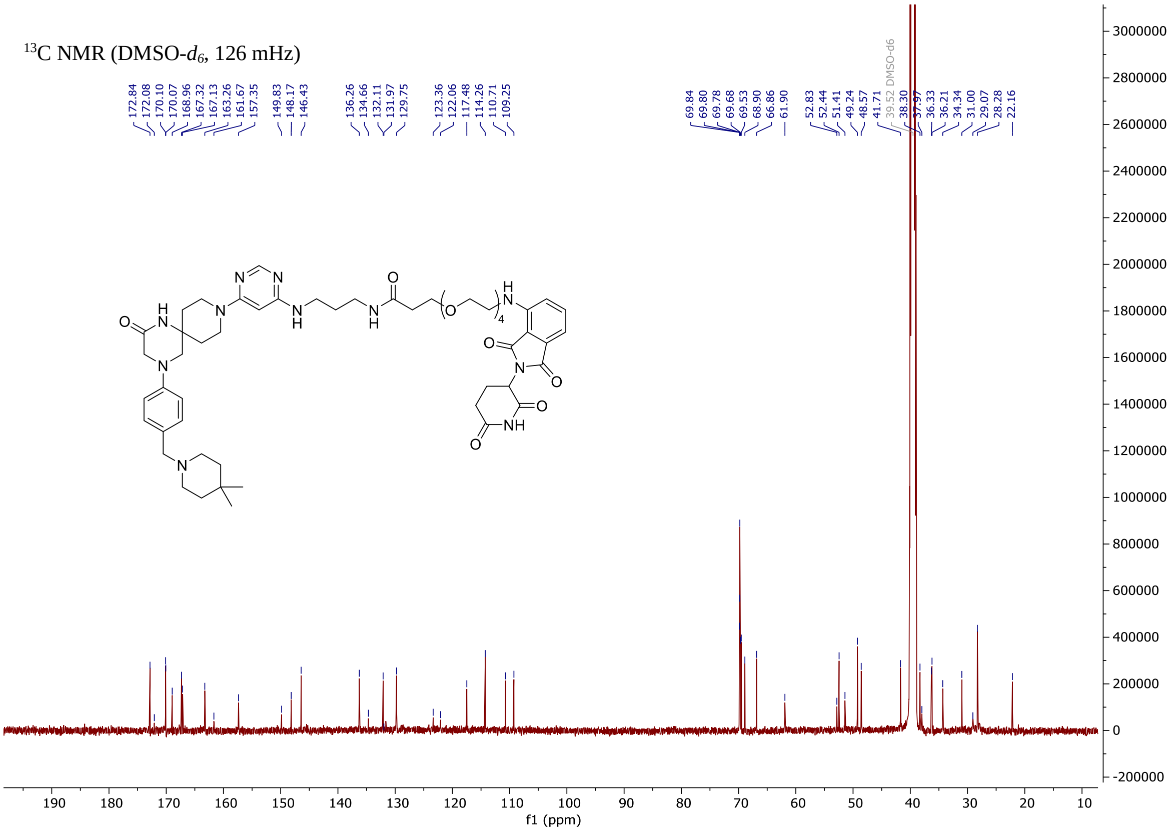

N-(3-((6-(4-(4-((4,4-dimethylpiperidin-1-yl)methyl)phenyl)- 2-oxo-1,4,9-triazaspiro[5.5]undecan-9-yl)pyrimidin-4-yl)amino)propyl)-1-((2-(2,6-dioxopiperidin-3-yl)-1,3-dioxoisoindolin-4-yl)amino)-3,6,9,12,15-pentaoxaoctadecan-18-amide (4)

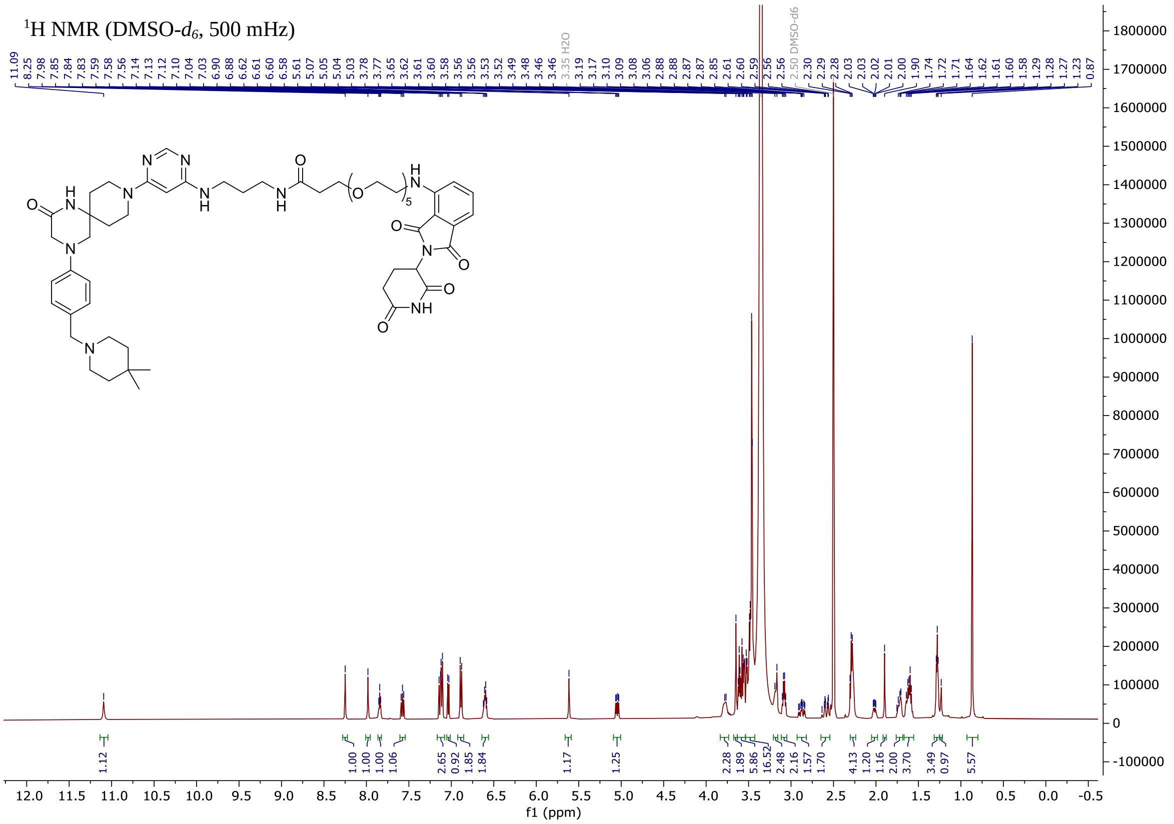

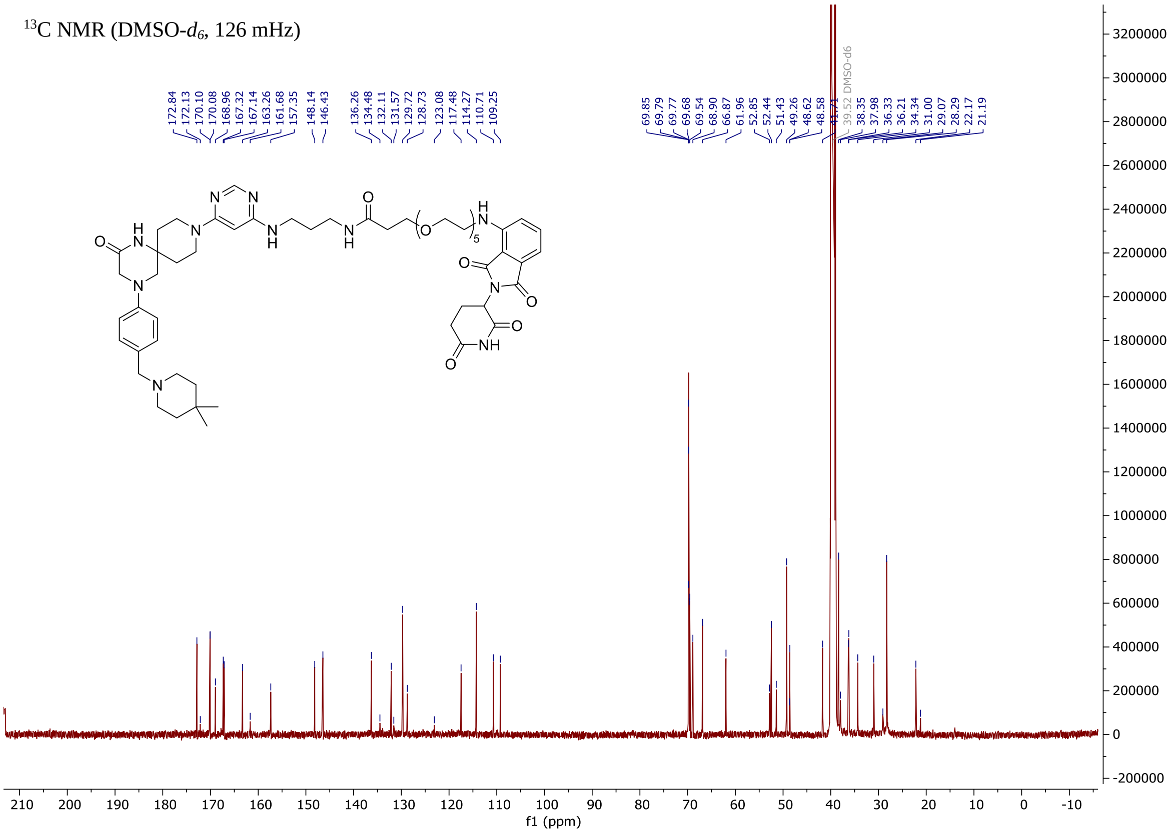

N-(3-((6-(4-(4-((4,4-dimethylpiperidin-1-yl)methyl)phenyl)-2-oxo-1,4,9-triazaspiro[5.5]undecan-9-yl)pyrimidin-4-yl)amino)propyl)-9-((2-(2,6-dioxopiperidin-3-yl)-1-oxoisoindolin-4-yl)amino)nonanamide (5)

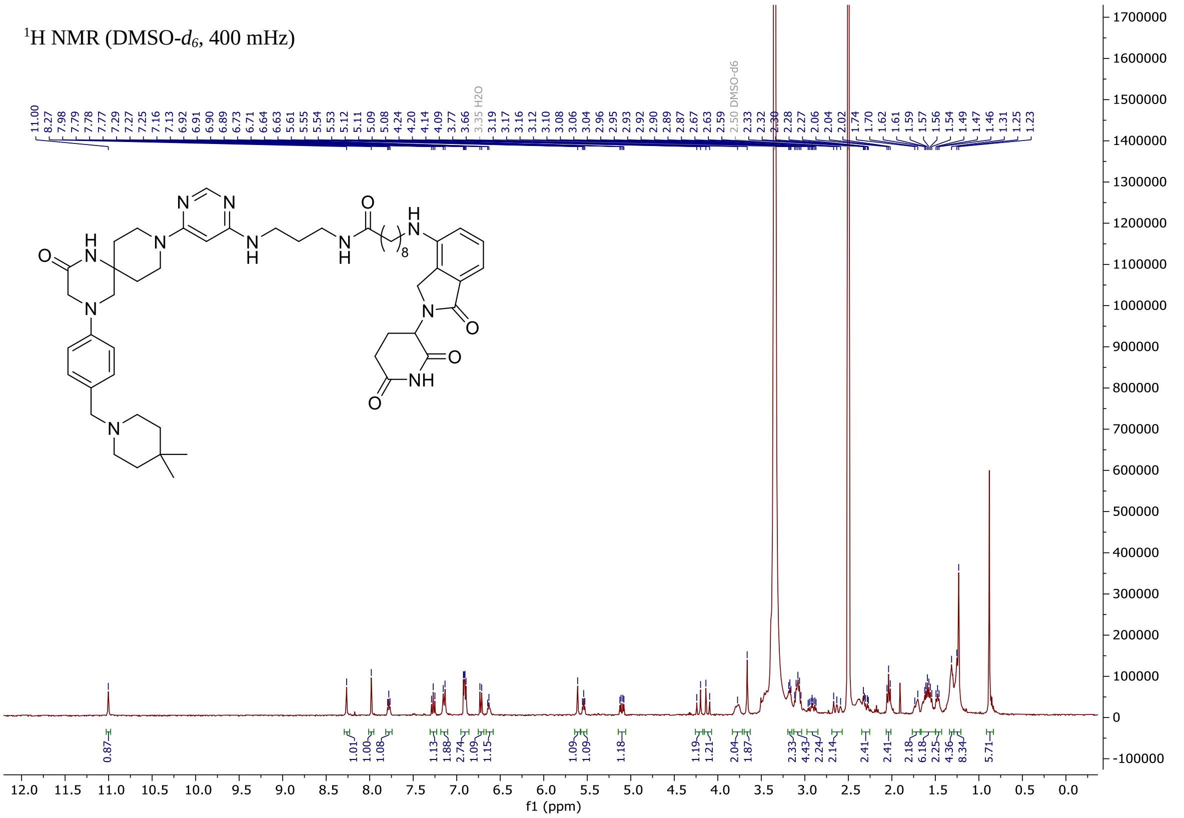

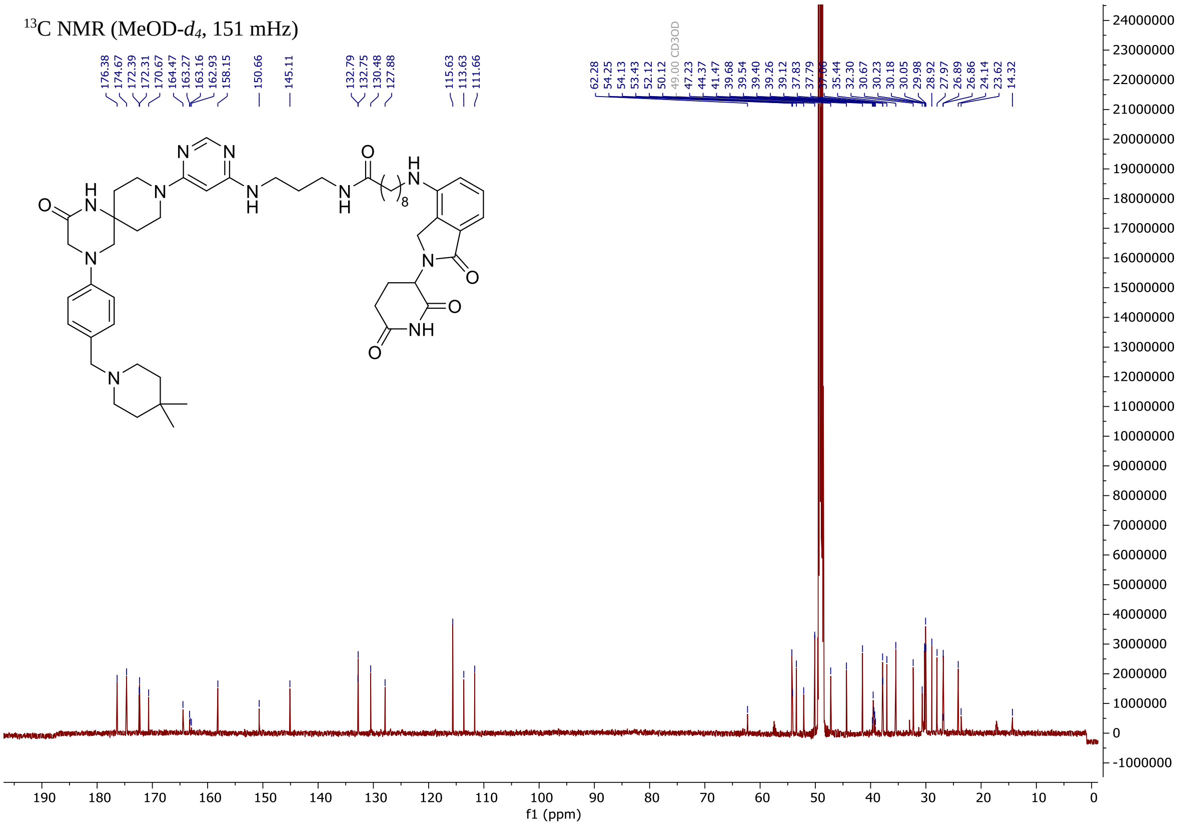

***N*-(3-((6-(4-(4-((4,4-dimethylpiperidin-1-yl)methyl)phenyl)-2-oxo -1,4,9-triazaspiro[5.5]undecan-9-yl)pyrimidin-4-yl)amino)propyl)-11-((2-(2,6-dioxopiperidin-3-yl)-1-oxoisoindolin-4-yl)amino)undecanamide (6)**

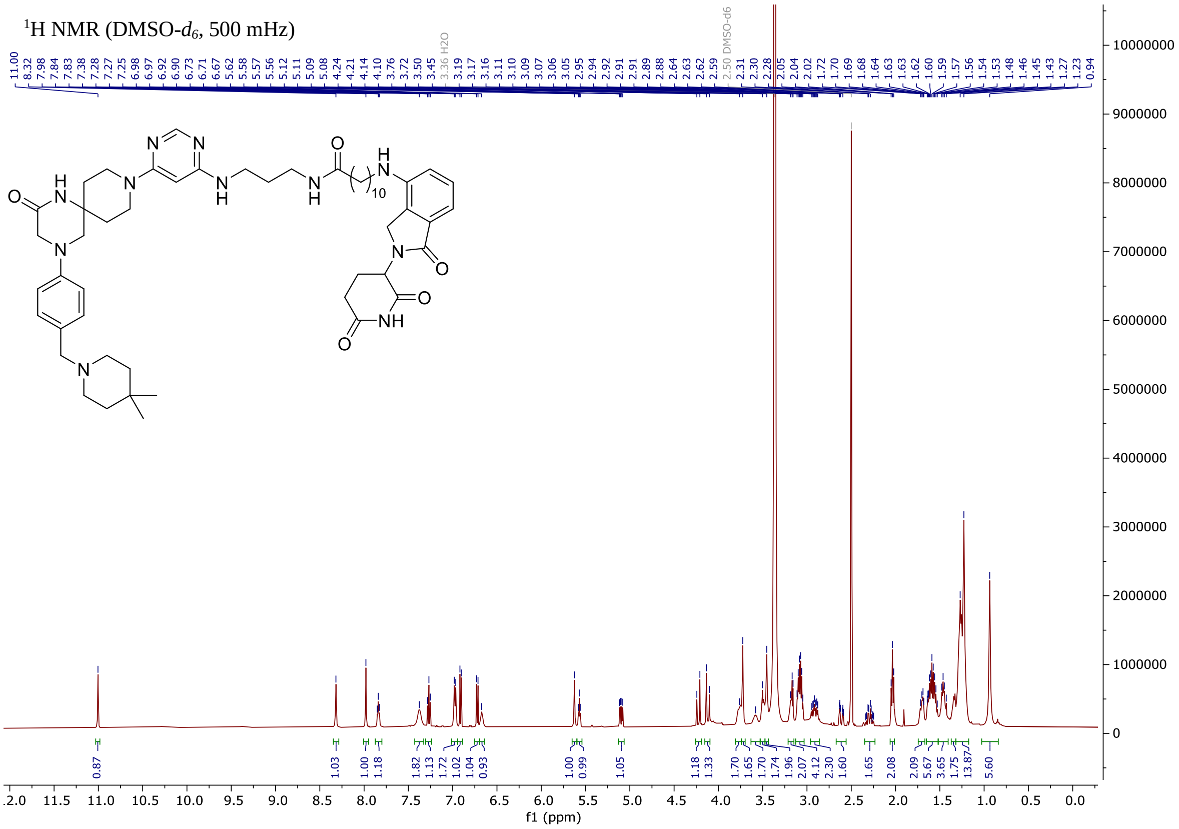

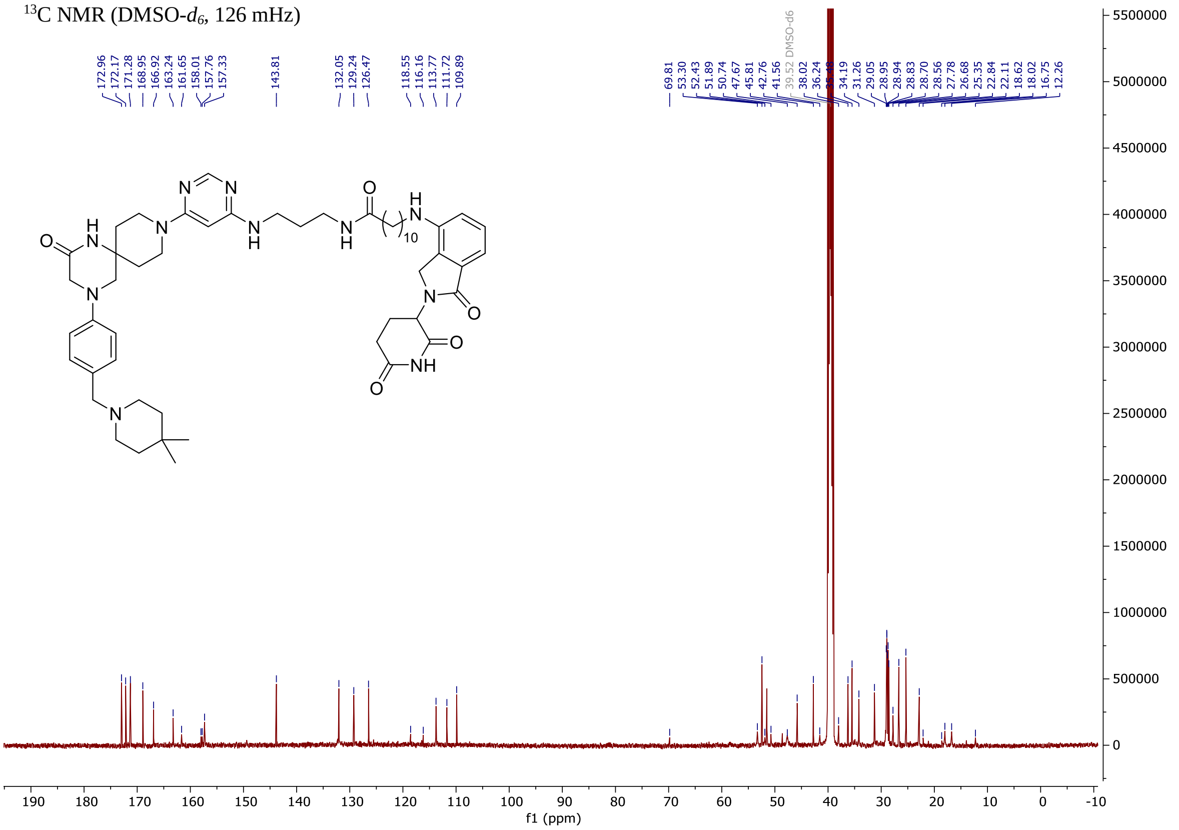

N*-(3-((6-(4-(4-((4,4-dimethylpiperidin-1-yl)methyl)phenyl)-2-oxo-1,4,9-triaza-spiro[5.5]undecan-9-yl)pyrimidin-4-yl)amino)propyl)-15-((2-(2,6-dioxopiperidin-3-yl)-1-oxoisoindolin-4-yl)amino)pentadecanamide (7)

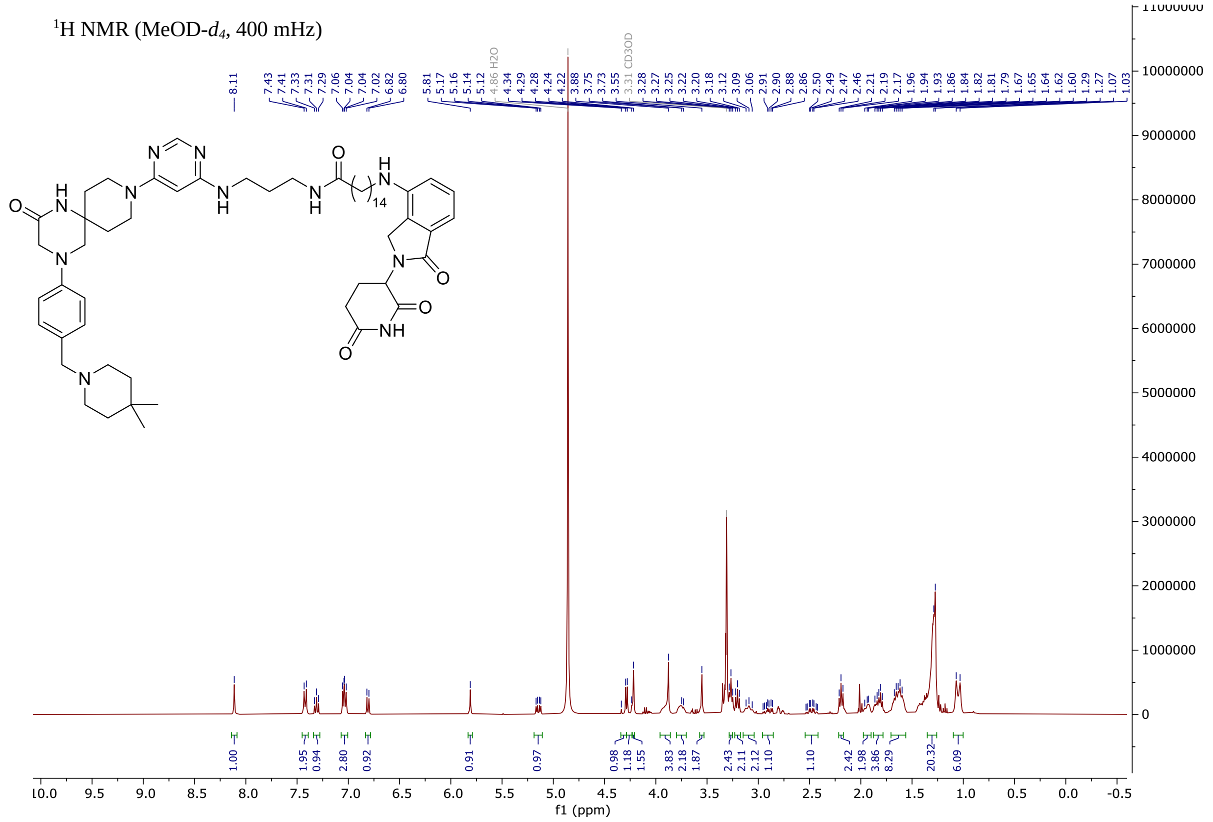

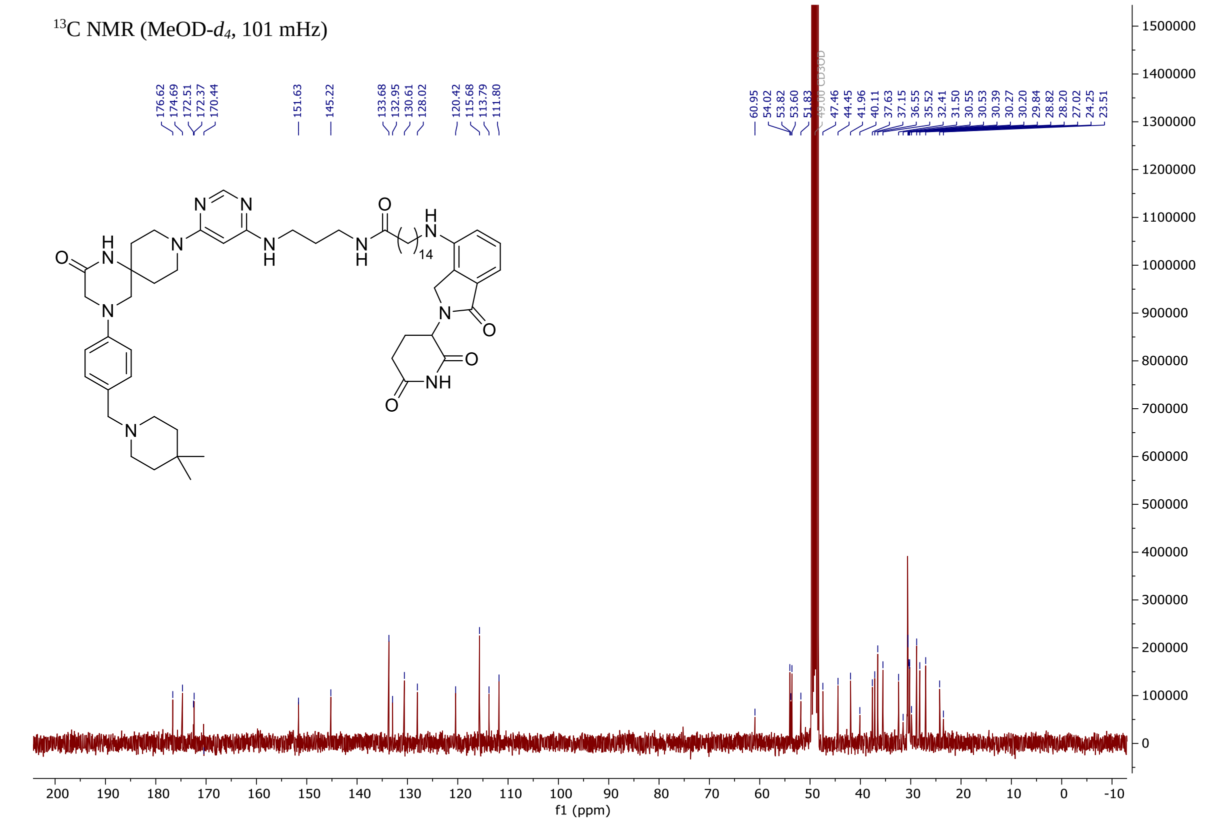

N*-(3-((6-(4-(4-((4,4-dimethylpiperidin-1-yl)methyl)phenyl)-2-oxo-1,4,9-triazaspiro[5.5]undecan-9-yl)pyrimidin-4-yl)amino)propyl)-11-(4-(((2-(2,6-dioxopiperidin-3-yl)-1,3-dioxoisoindolin-4-yl)amino)methyl)-1*H*-1,2,3-triazol-1-yl)undecanamide (8)

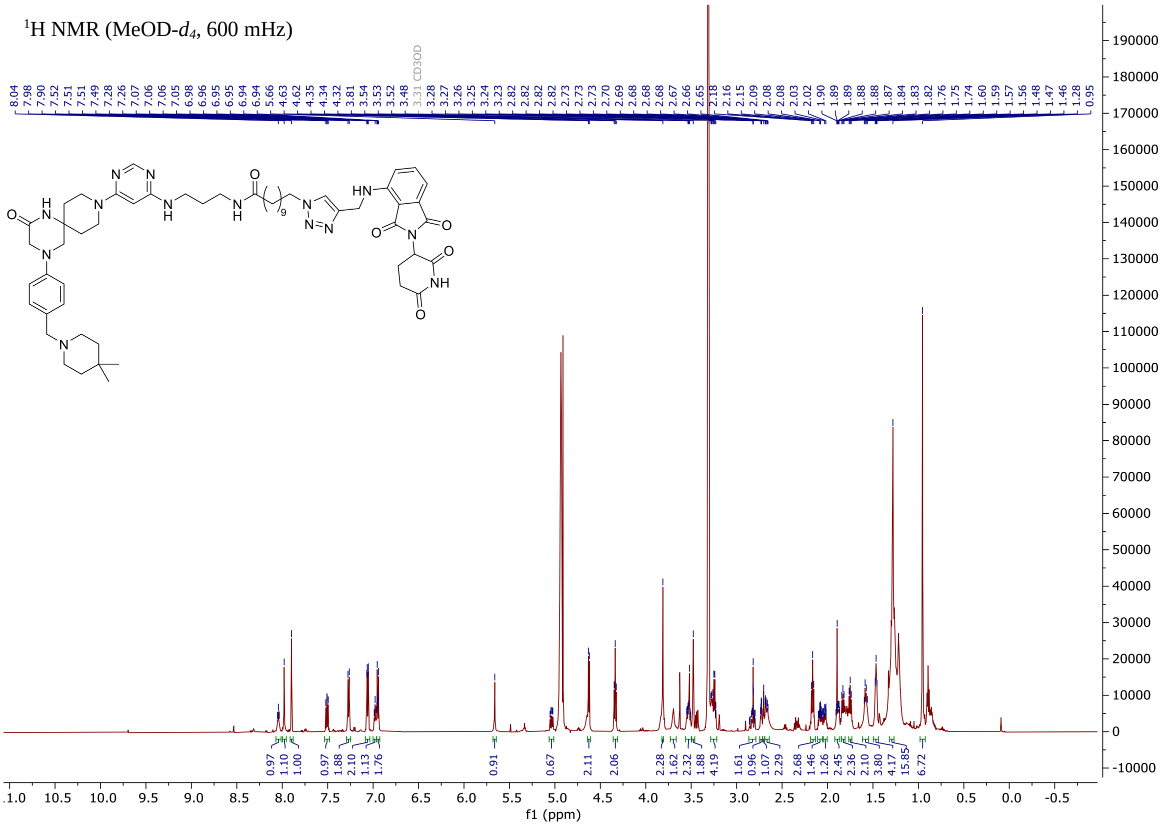

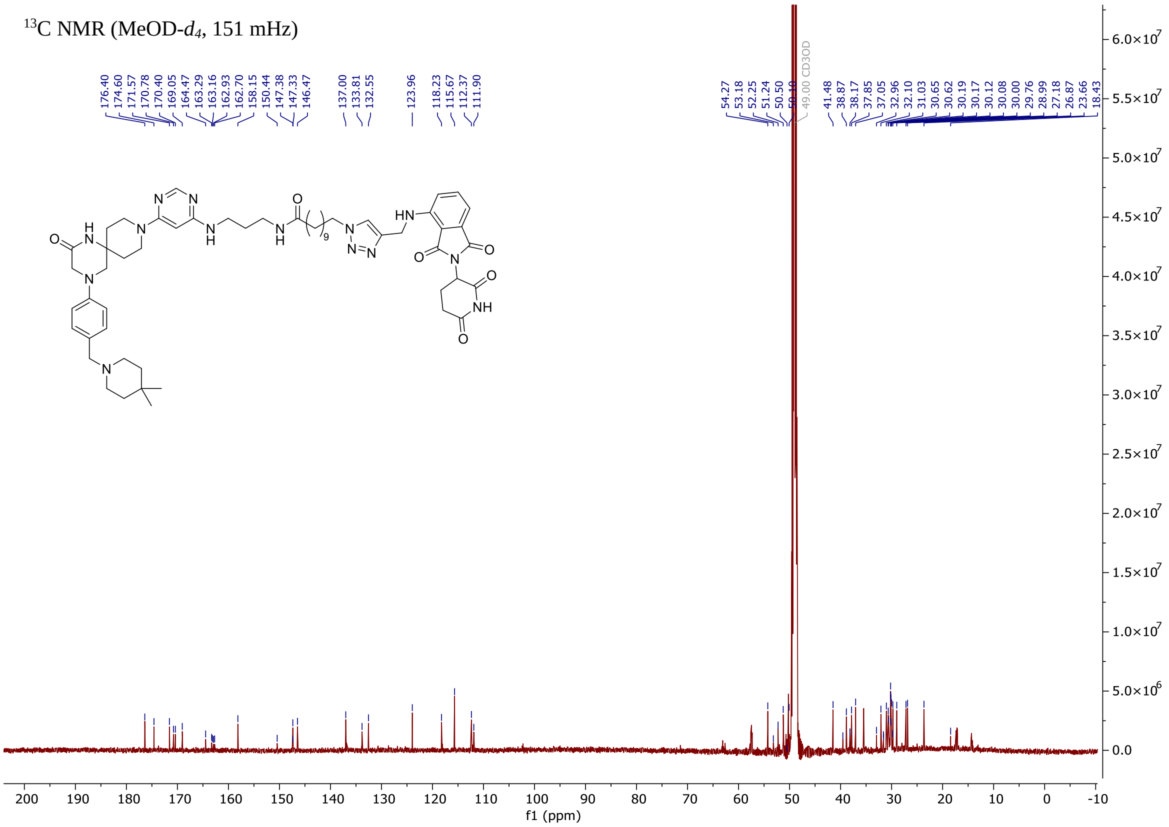

N-(3-((6-(4-(4-((4,4-dimethylpiperidin-1-yl)methyl)phenyl)-2-oxo-1,4,9-triazaspiro[5.5]undecan-9-yl)pyrimidin-4-yl)amino)propyl)-9-(4-(((2-(2,6-dioxopiperidin-3-yl)-1,3-dioxoisoindolin-4-yl)amino)methyl)-1H-1,2,3-triazol-1-yl)nonanamide (9)

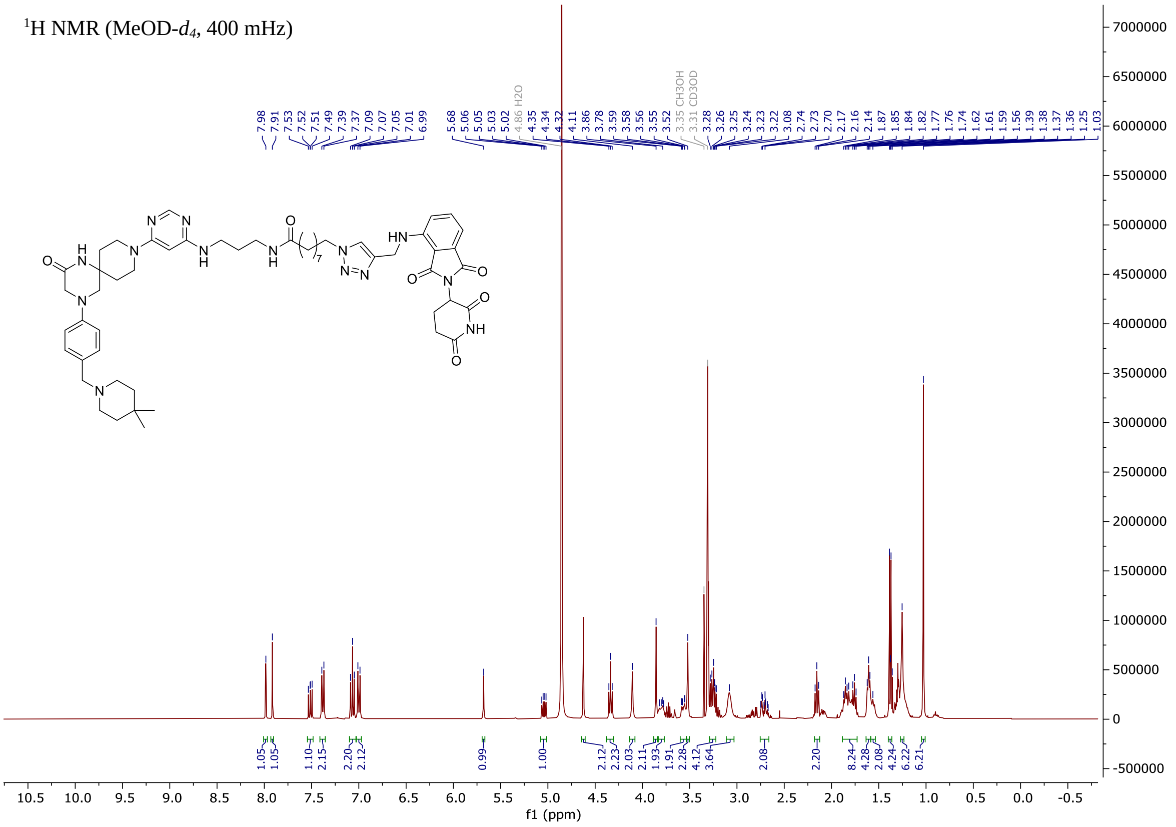

^{1}H NMR (MeOD-d_4, 400 mHz)

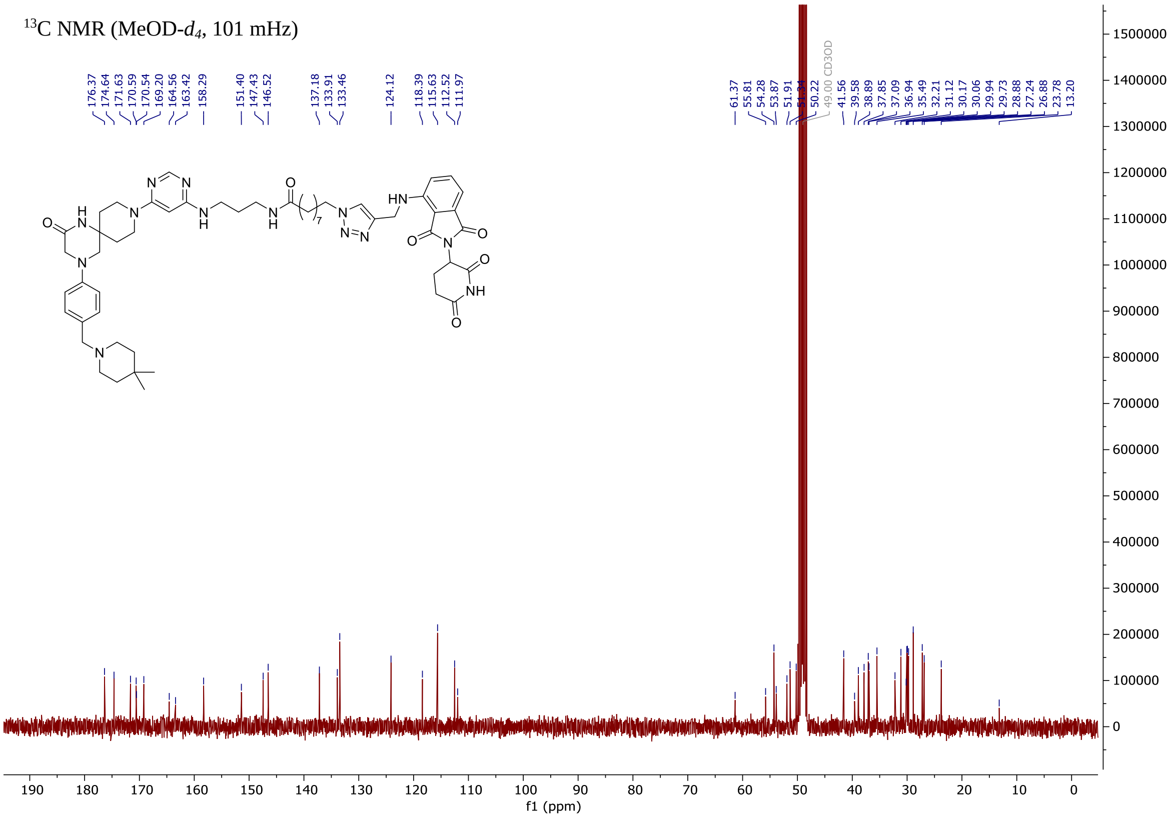

^{13}C NMR (MeOD-d_4, 101 mHz)

N-(3-((6-(4-(4-((4,4-dimethylpiperidin-1-yl)methyl)-2,5-difluorophenyl)- 2-oxo-1,4,9-triazaspiro[5.5]undecan-9-yl)pyrimidin-4-yl)amino)propyl)-9-((2-(2,6-dioxopiperidin-3-yl)-1-oxoisoindolin-4-yl)amino)nonanamide (10)

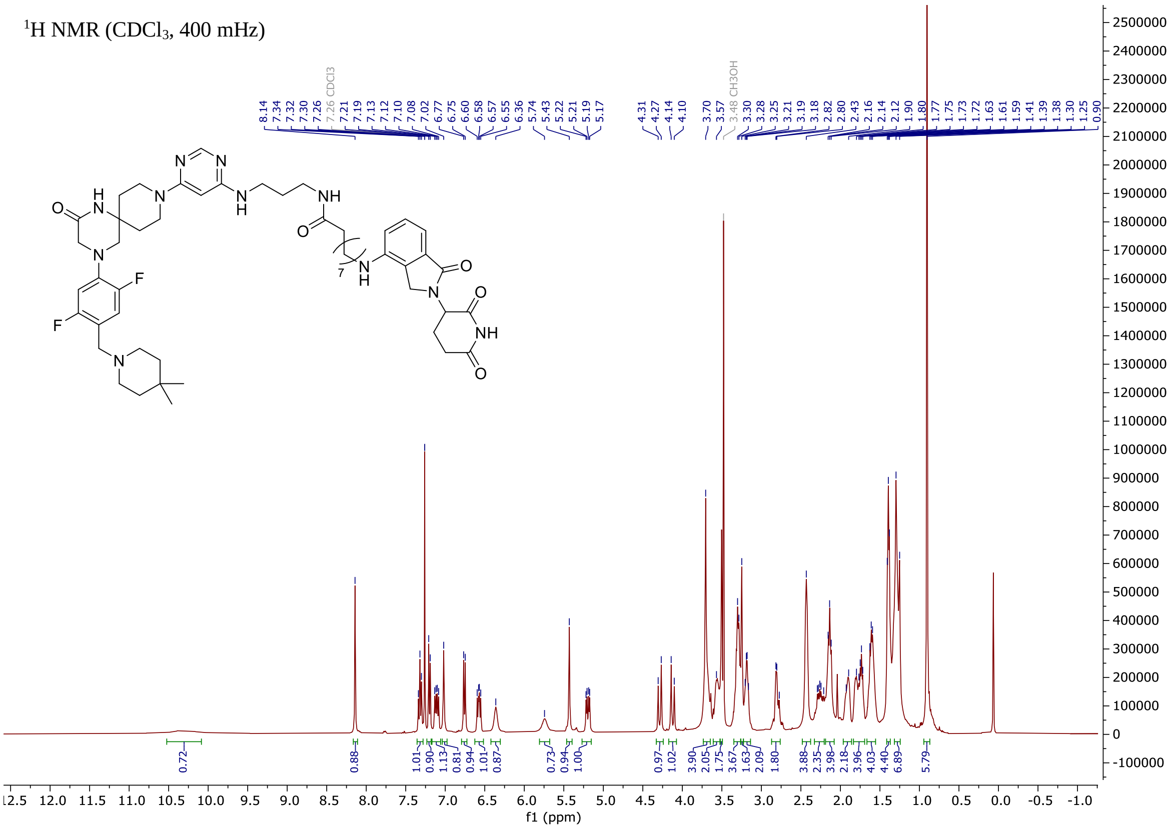

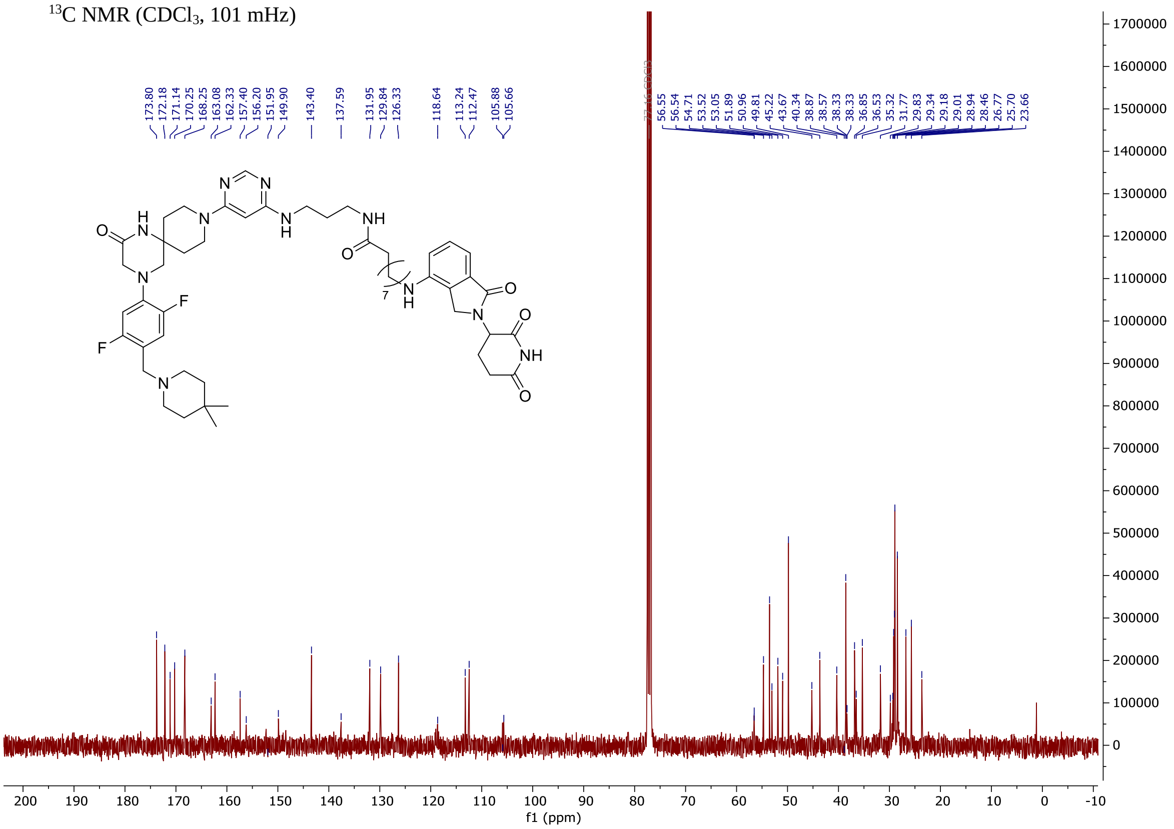

N-(3-((6-(4-(4-((4,4-dimethylpiperidin-1-yl)methyl)-2,5-difluorophenyl)- 2-oxo-1,4,9-triazaspiro[5.5]undecan-9-yl)pyrimidin-4-yl)amino)propyl)-11-((2-(2,6-dioxopiperidin-3-yl)-1-oxoisoindolin-4-yl)amino)undecanamide (11)

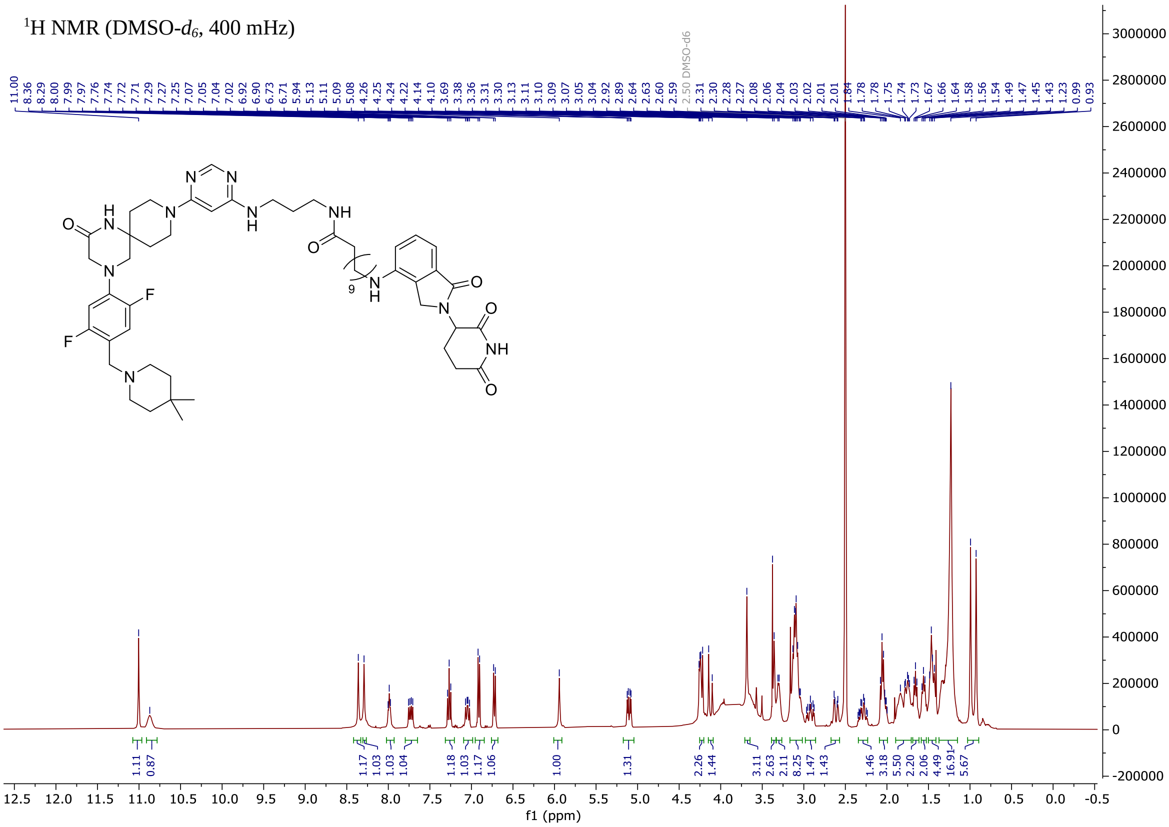

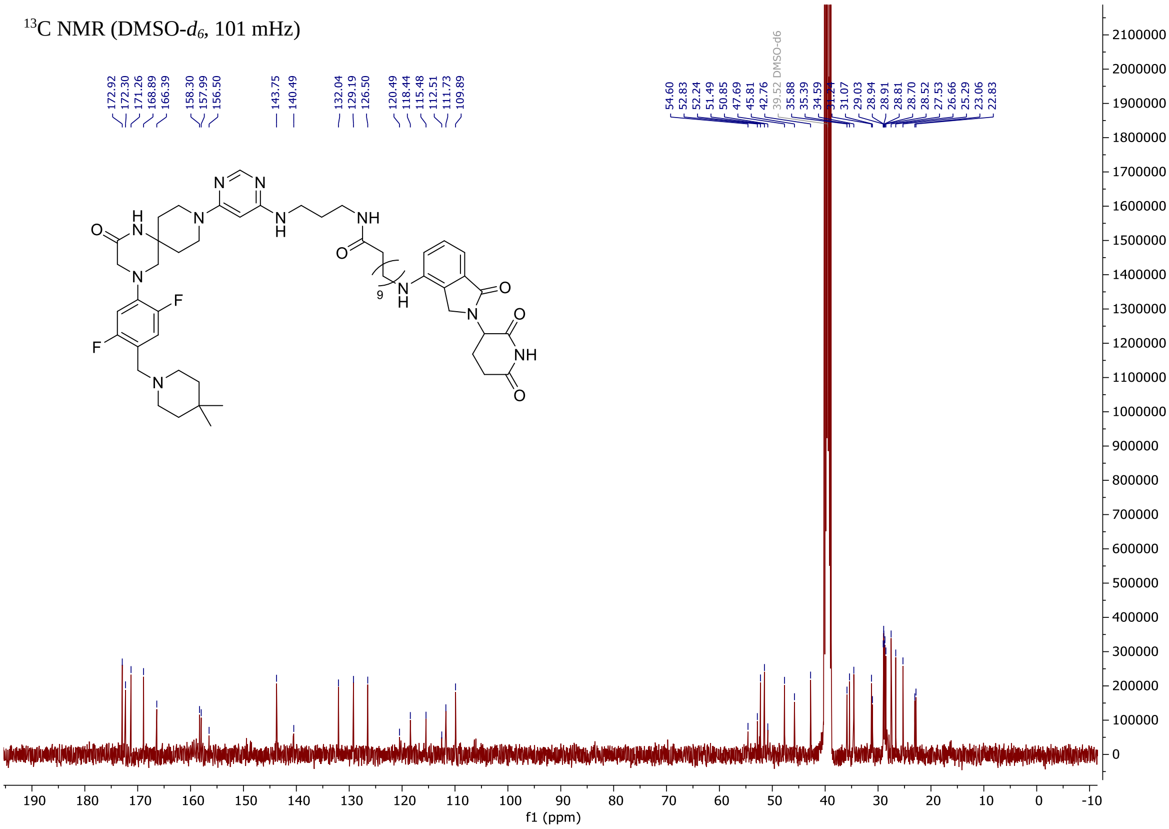

N-(3-((6-(4-(4-((4,4-dimethylpiperidin-1-yl)methyl)-2,5-difluorophenyl)-2-oxo-1,4,9-triazaspiro[5.5]undecan-9-yl)pyrimidin-4-yl)amino)propyl)-9-(4-(((2-(2,6-dioxopiperidin-3-yl)-1,3-dioxoisoindolin-4-yl)amino)methyl)-1H-1,2,3-triazol-1-yl)nonanamide (12)

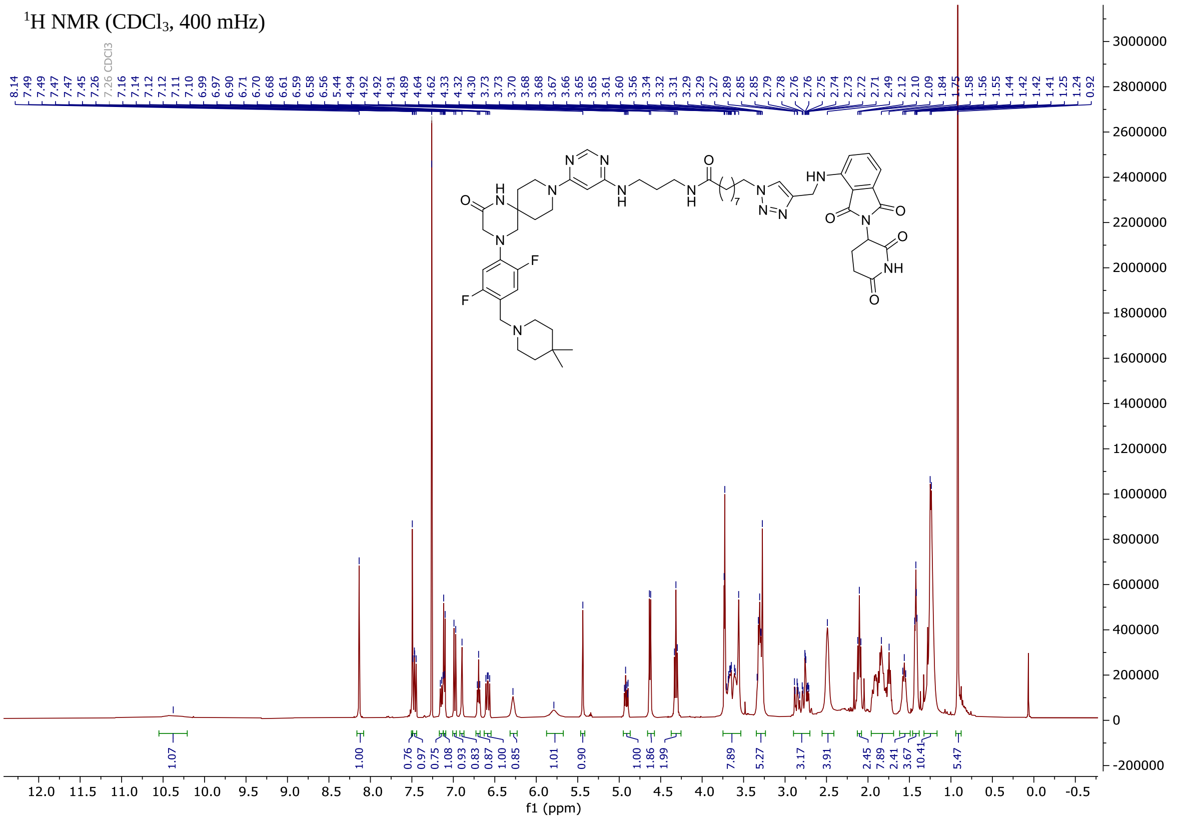

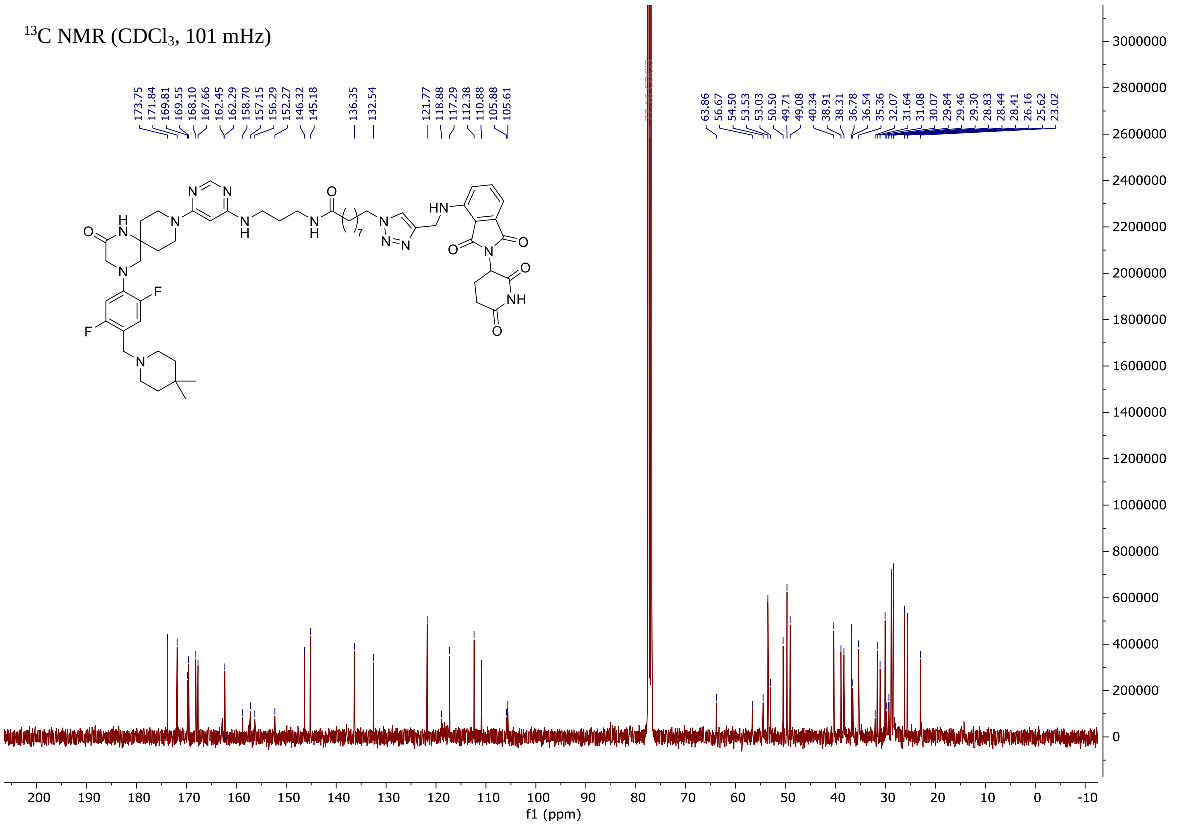

***N*-(3-((6-(4-(4-((4,4-dimethylpiperidin-1-yl)methyl)-2,5-difluorophenyl)-2-oxo-1,4,9-triazaspiro[5.5]undecan-9-yl)pyrimidin-4-yl)amino)propyl)-11-(4-(((2-(2,6-dioxopiperidin-3-yl)-1,3-dioxoisoindolin-4-yl)amino)methyl)-1*H*-1,2,3-triazol-1-yl)undecanamide (13)**

^{1}H NMR ($CDCl_3$, 400 mHz)

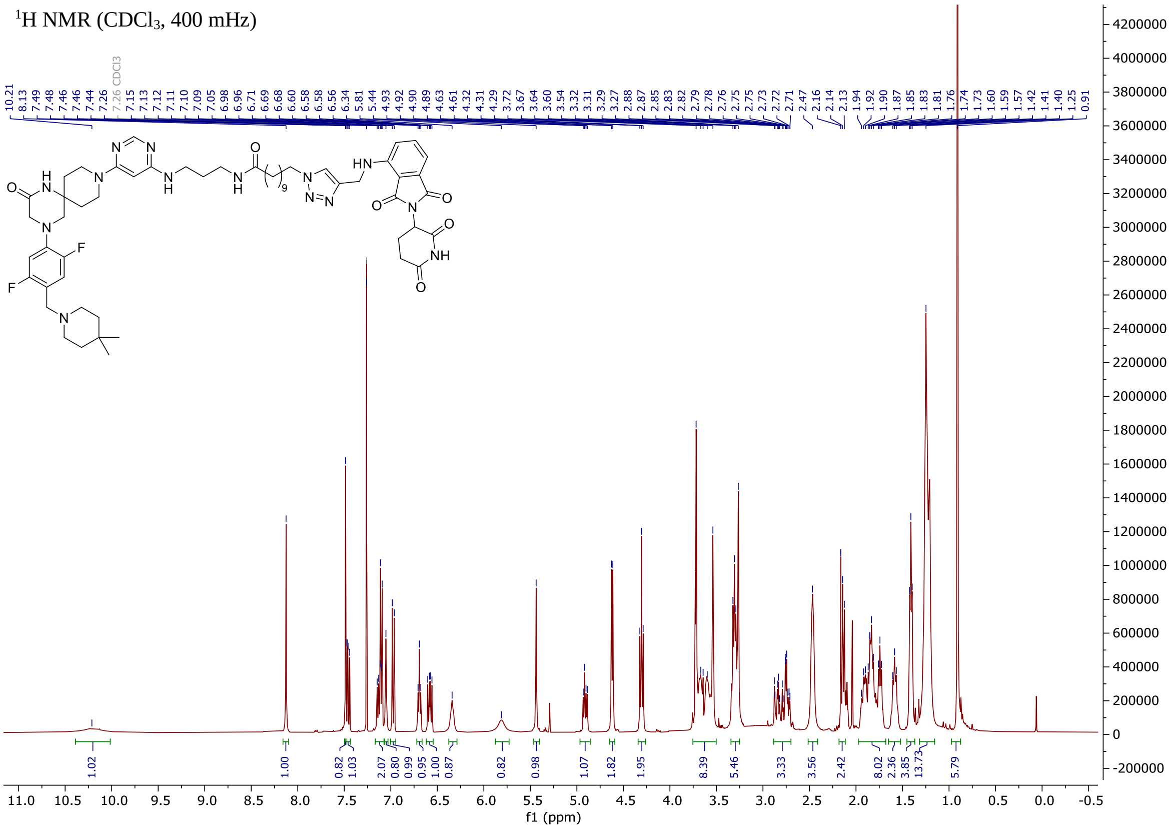

^{13}C NMR ($CDCl_3$, 101 mHz)

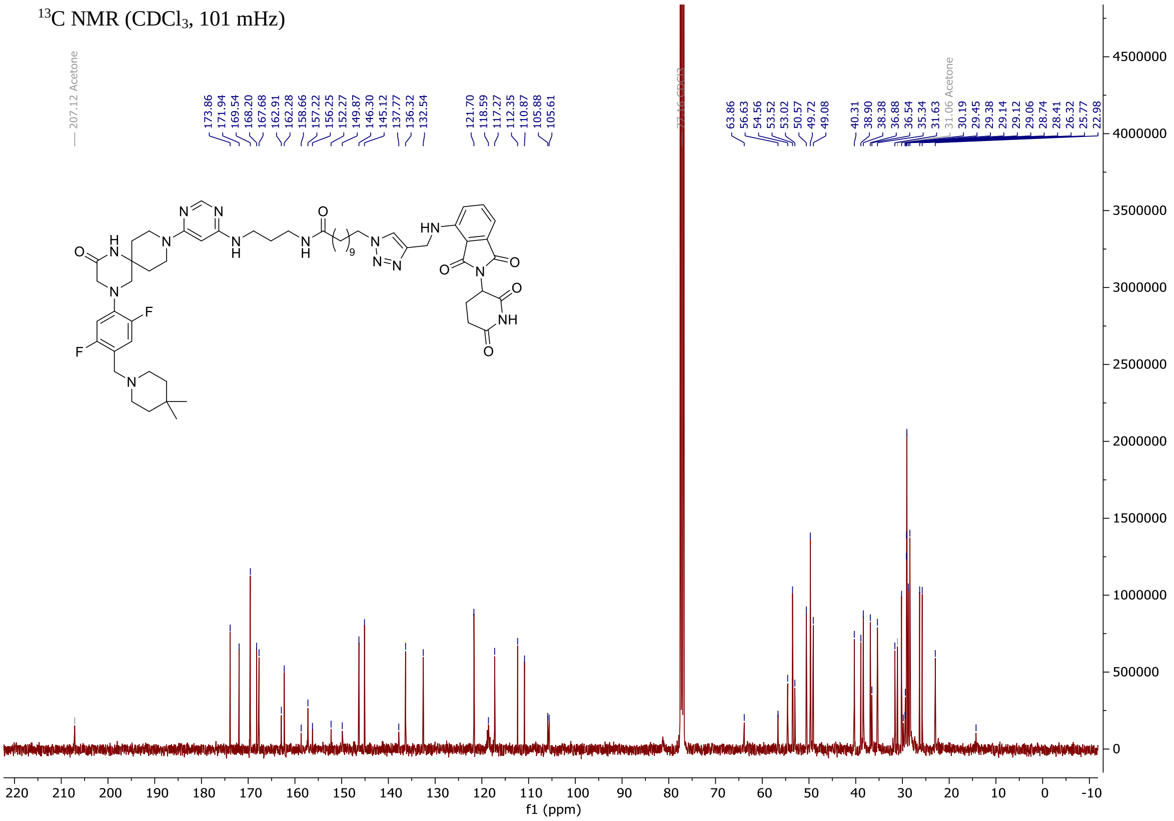

N-(3-(((6-(4-(4-((4,4-dimethylpiperidin-1-yl)methyl)-2,5-difluorophenyl)- 2-oxo-1,4,9-triazaspiro[5.5]undecan-9-yl)pyrimidin-4-yl)amino)methyl)benzyl)-4-((2-(2,6-dioxopiperidin-3-yl)-1,3-dioxoisoindolin-4-yl)amino)butanamide (14)

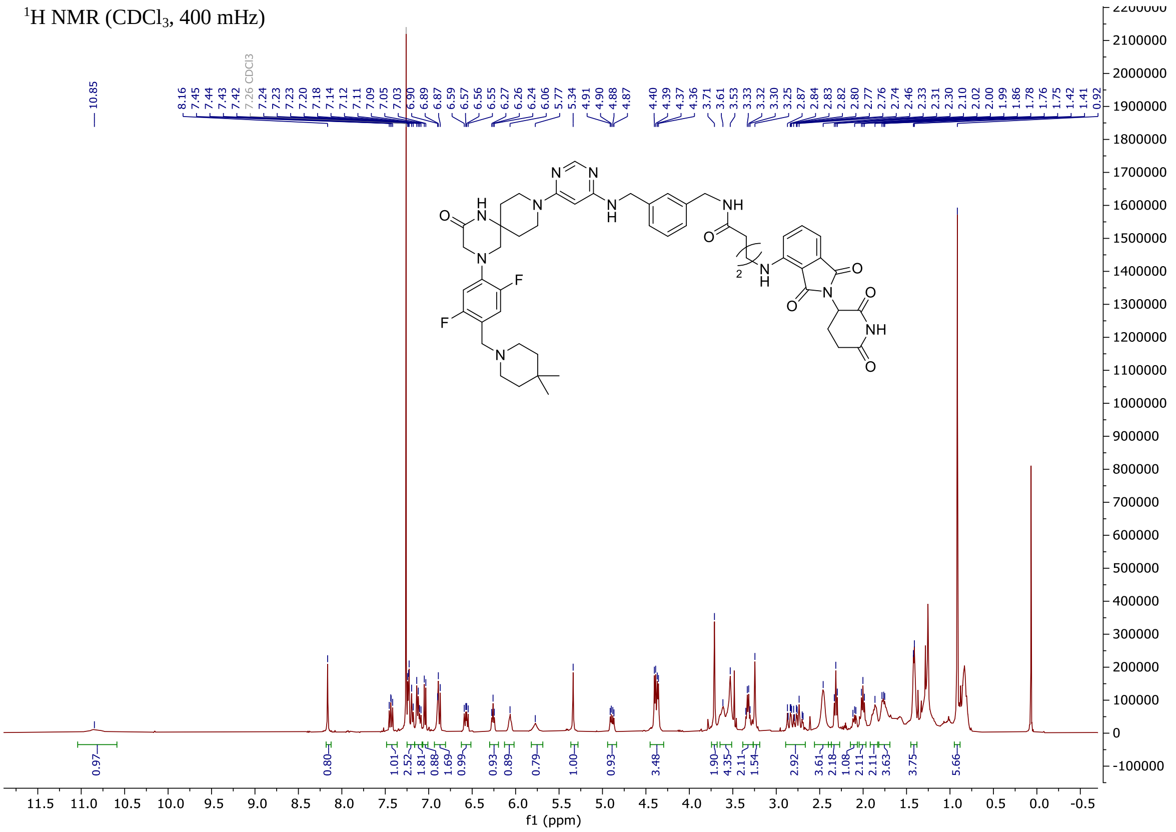

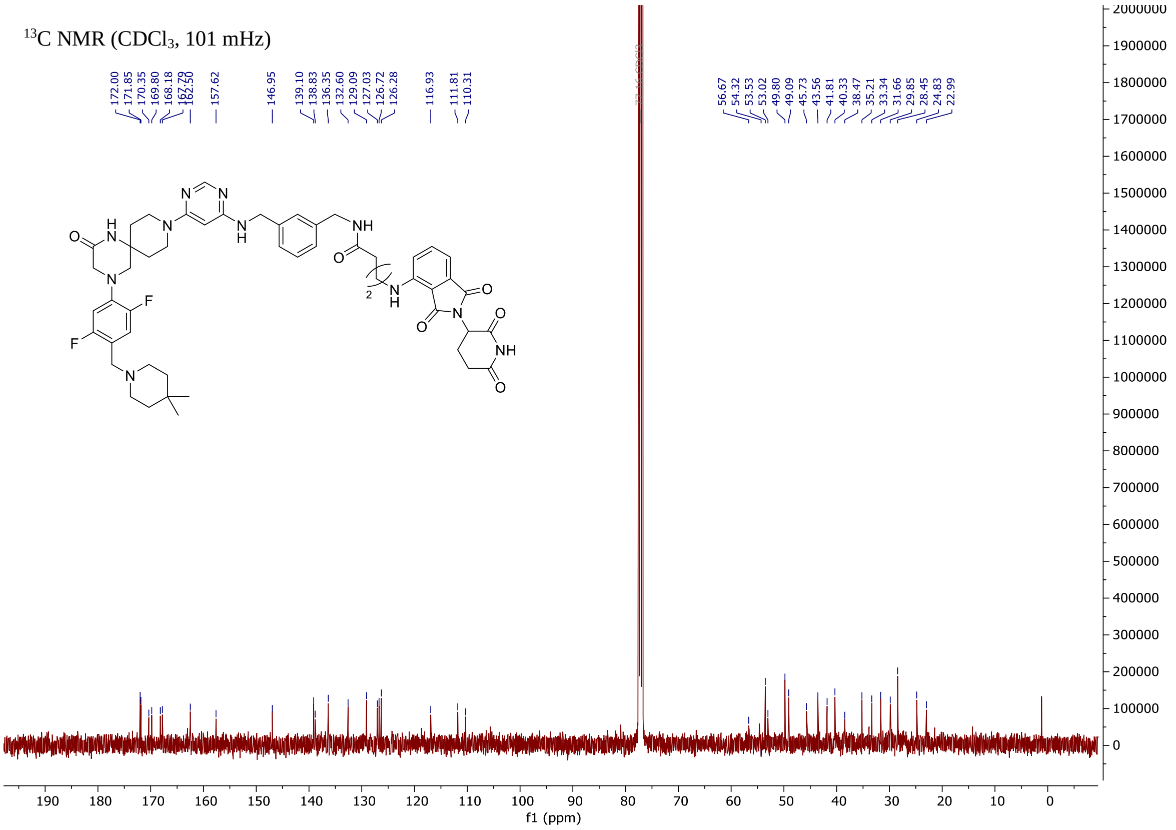

N-(3-(((6-(4-(4-((4,4-dimethylpiperidin-1-yl)methyl)-2,5-difluorophenyl)-2-oxo-1,4,9-triazaspiro[5.5]undecan-9-yl)pyrimidin-4-yl)amino)methyl)benzyl)-6-((2-(2,6-dioxopiperidin-3-yl)-1,3-dioxoisoindolin-4-yl)amino)hexanamide (15)

^{1}H NMR (MeOD-d_4, 400 mHz)

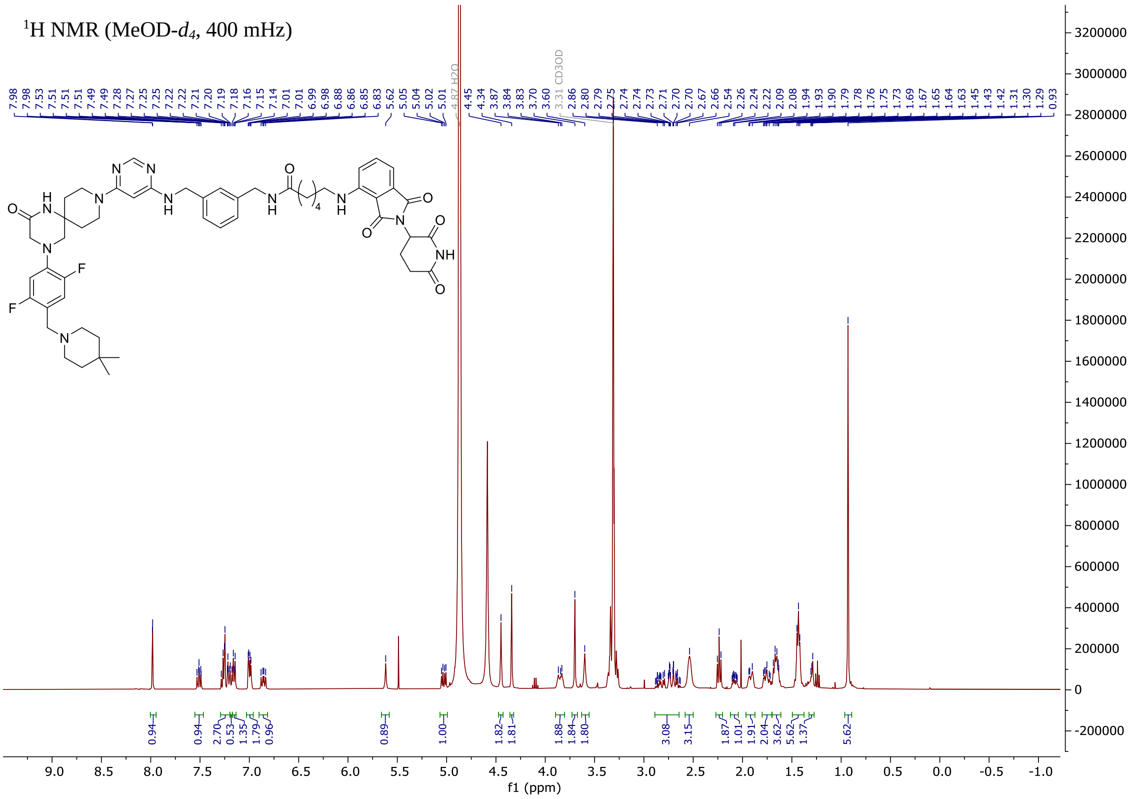

^{13}C NMR (MeOD-d_4, 101 mHz)

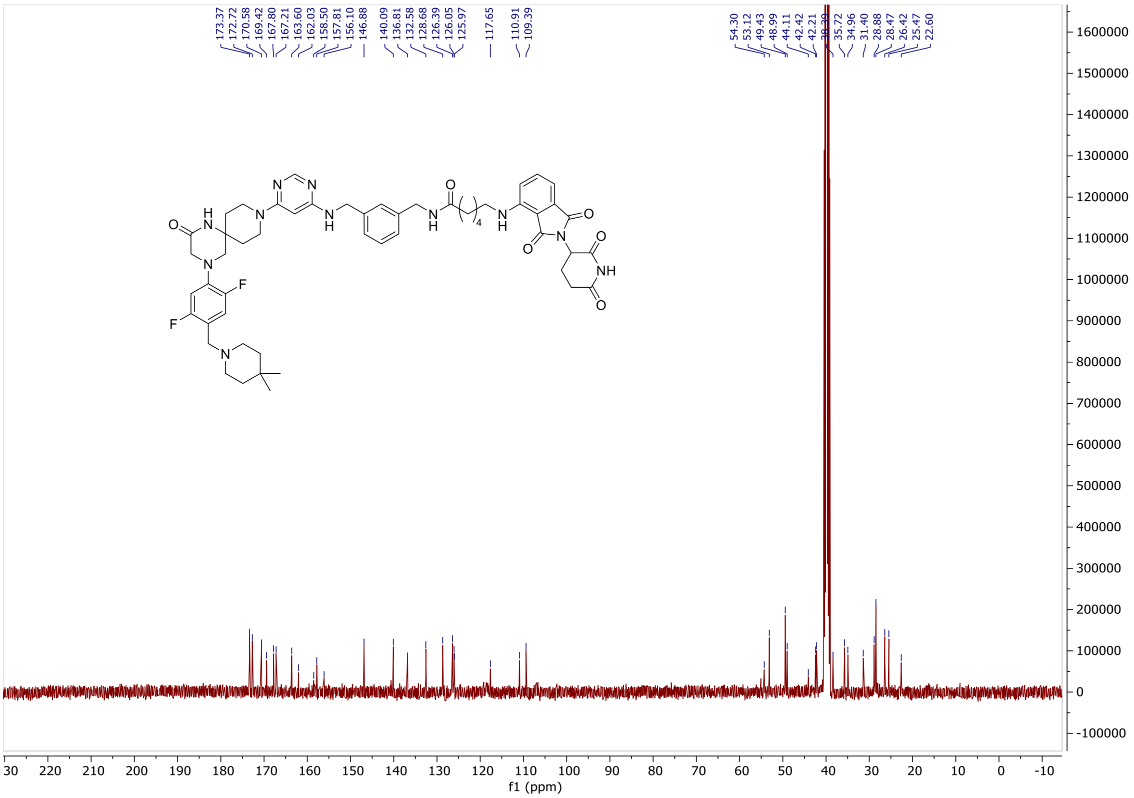

N*-(3-(((6-(4-(4-((4,4-dimethylpiperidin-1-yl)methyl)-2,5-difluorophenyl)-2- oxo-1,4,9-triazaspiro[5.5]undecan-9-yl)pyrimidin-4-yl)amino)methyl)benzyl)-9-((2-(2,6-dioxopiperidin-3-yl)-1-oxoisoindolin-4-yl)amino)nonanamide (16)

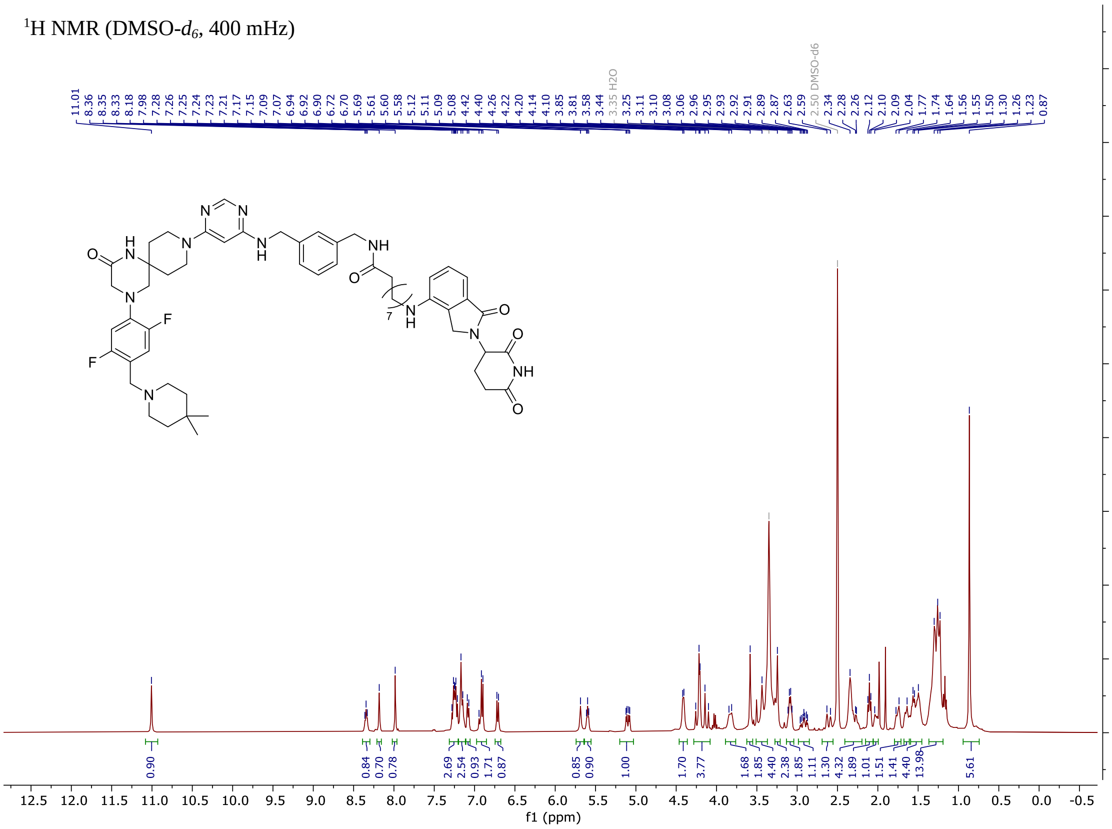

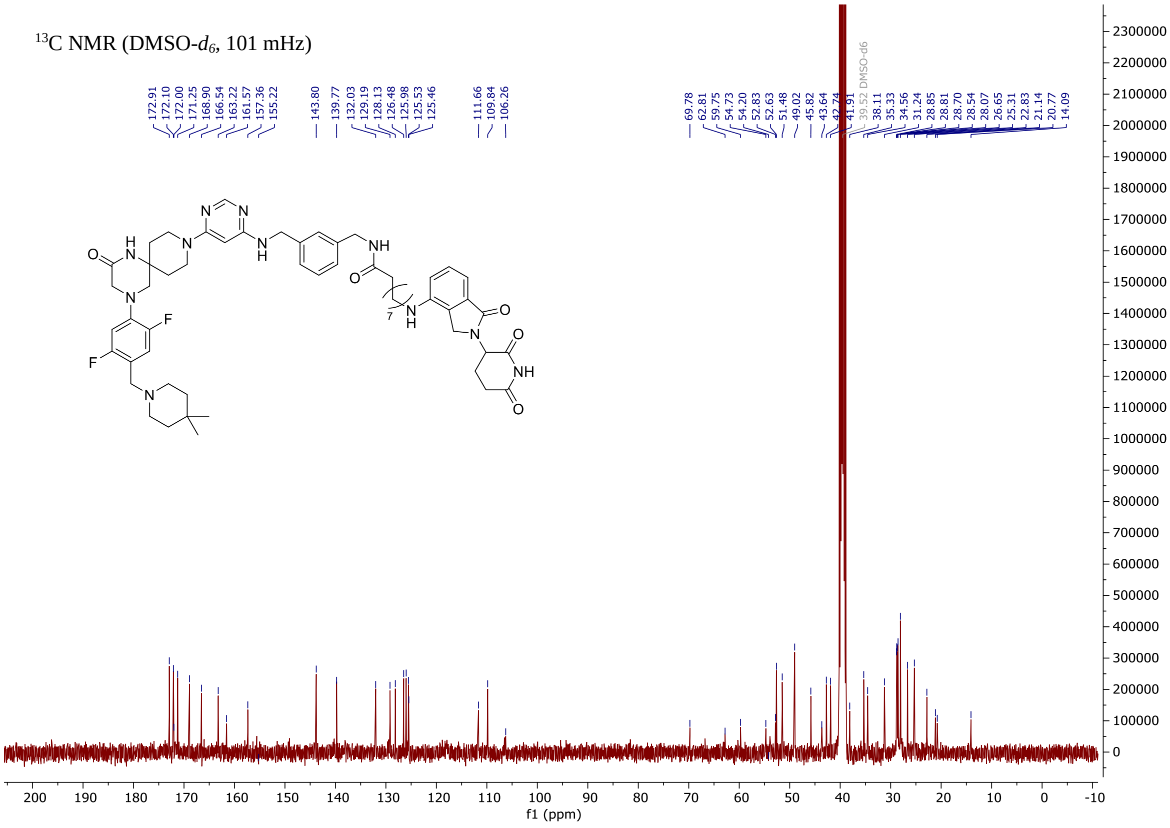

N-(3-(((6-(4-(4-((4,4-dimethylpiperidin-1-yl)methyl)-2,5-difluorophenyl)-2-oxo-1,4,9-triazaspiro[5.5]undecan-9-yl)pyrimidin-4-yl)amino)methyl)benzyl)-11-((2-(2,6-dioxopiperidin-3-yl)-1-oxoisoindolin-4-yl)amino)undecanamide (17)

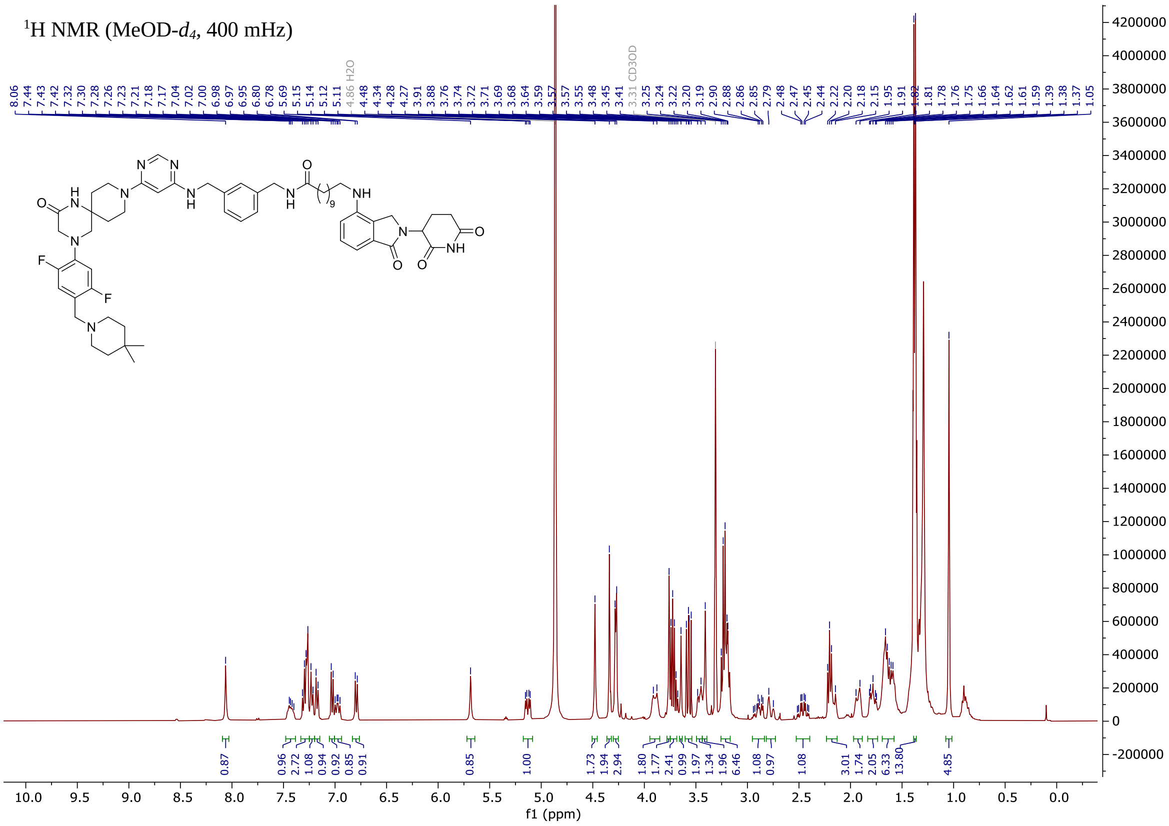

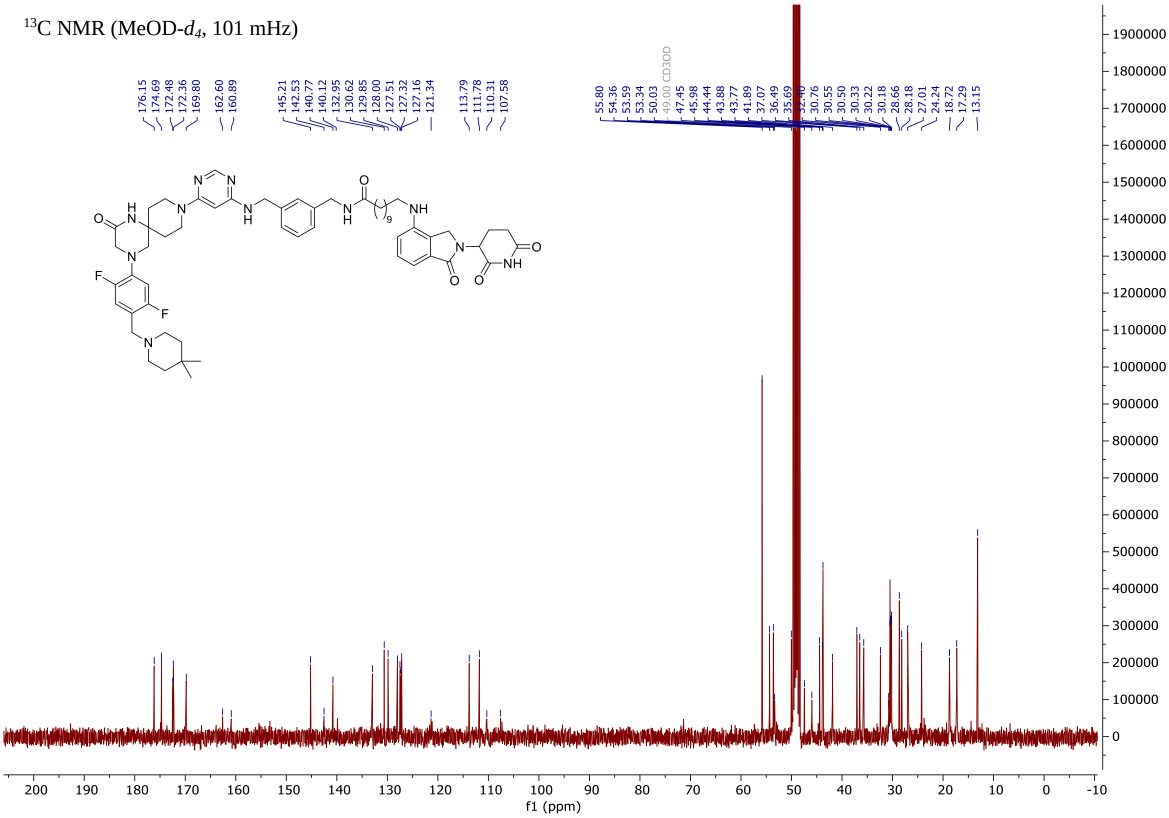

N-(3-(((6-(4-(4-((4,4-dimethylpiperidin-1-yl)methyl)-2,5-difluorophenyl)-2-oxo-1,4,9-triazaspiro[5.5]undecan-9-yl)pyrimidin-4-yl)amino)methyl)benzyl)-3-(4-(((2-(2,6-dioxopiperidin-3-yl)-1,3-dioxoisoindolin-4-yl)amino)methyl)-1H-1,2,3-triazol-1-yl)propenamide (18)

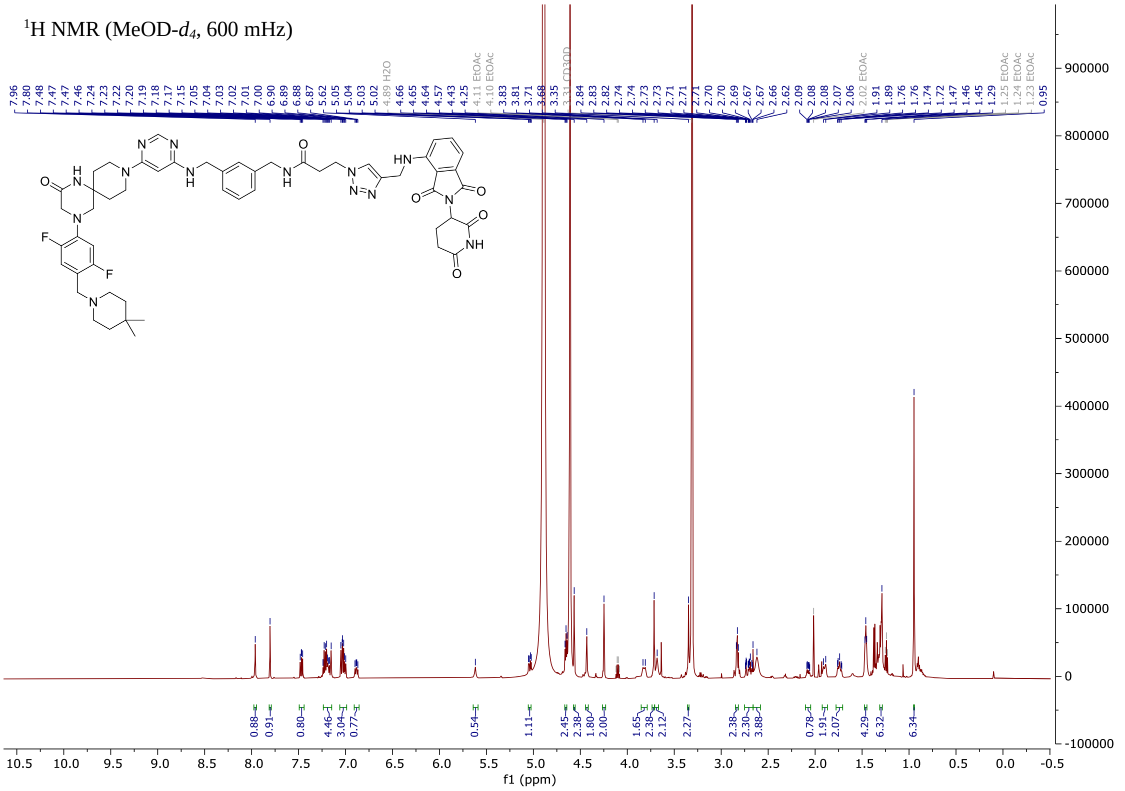

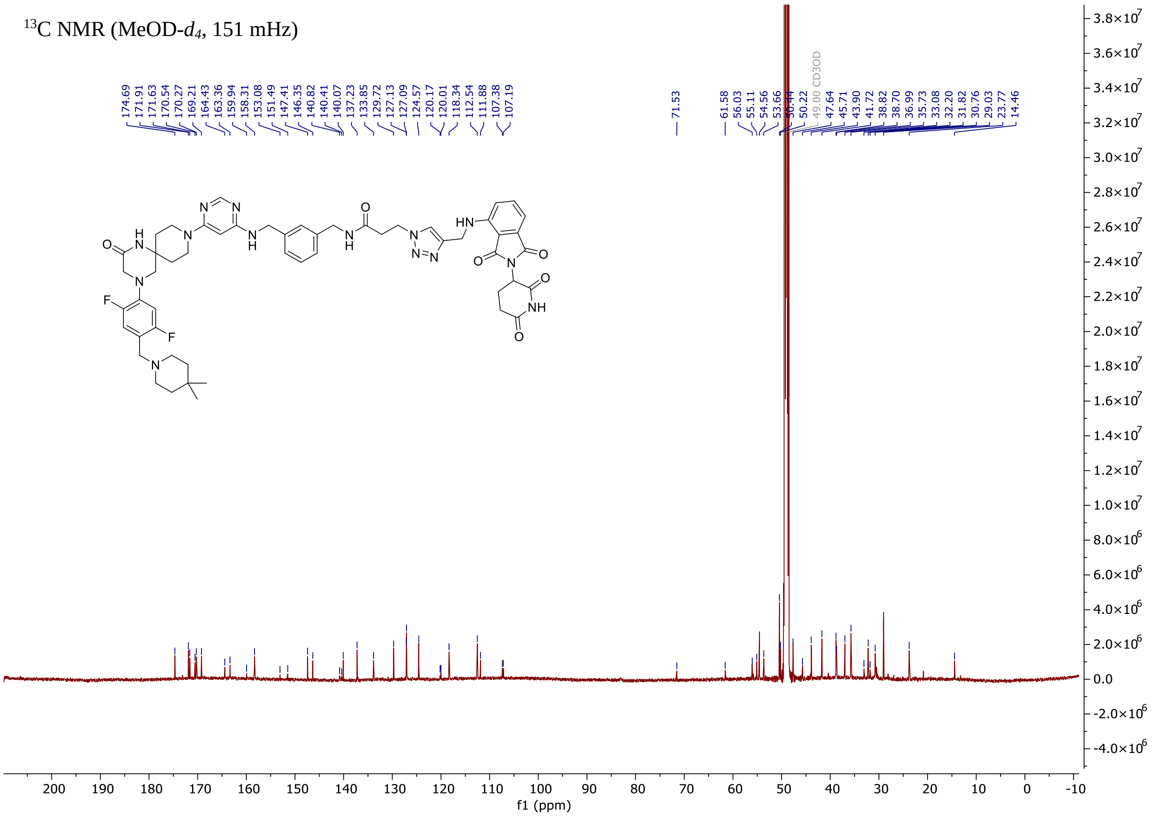

N-(3-(((6-(4-(4-((4,4-dimethylpiperidin-1-yl)methyl)-2,5-difluorophenyl)-2-oxo-1,4,9-triazaspiro[5.5]undecan-9-yl)pyrimidin-4-yl)amino)methyl)benzyl)-9-(4-(((2-(2,6-dioxopiperidin-3-yl)-1,3-dioxoisoindolin-4-yl)amino)methyl)-1H-1,2,3-triazol-1-yl)nonanamide (19)

^{1}H NMR ($CDCl_3$, 400 mHz)

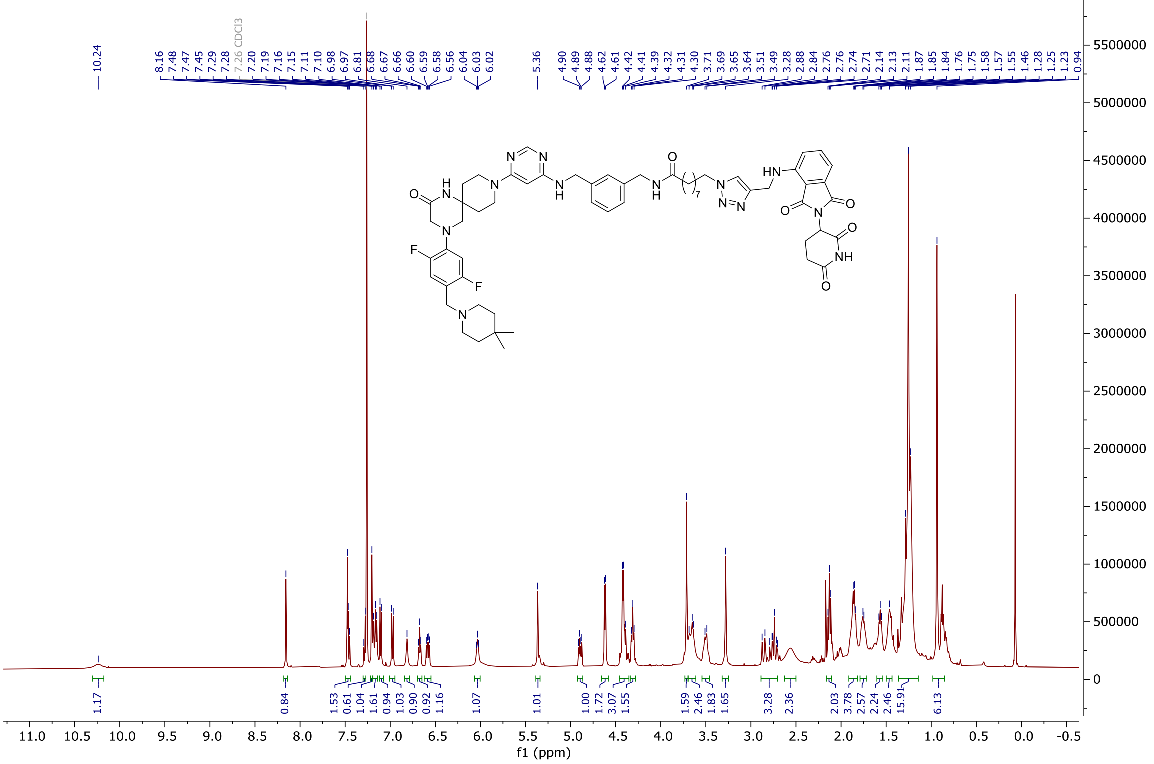

^{13}C NMR ($CDCl_3$, 101 mHz)

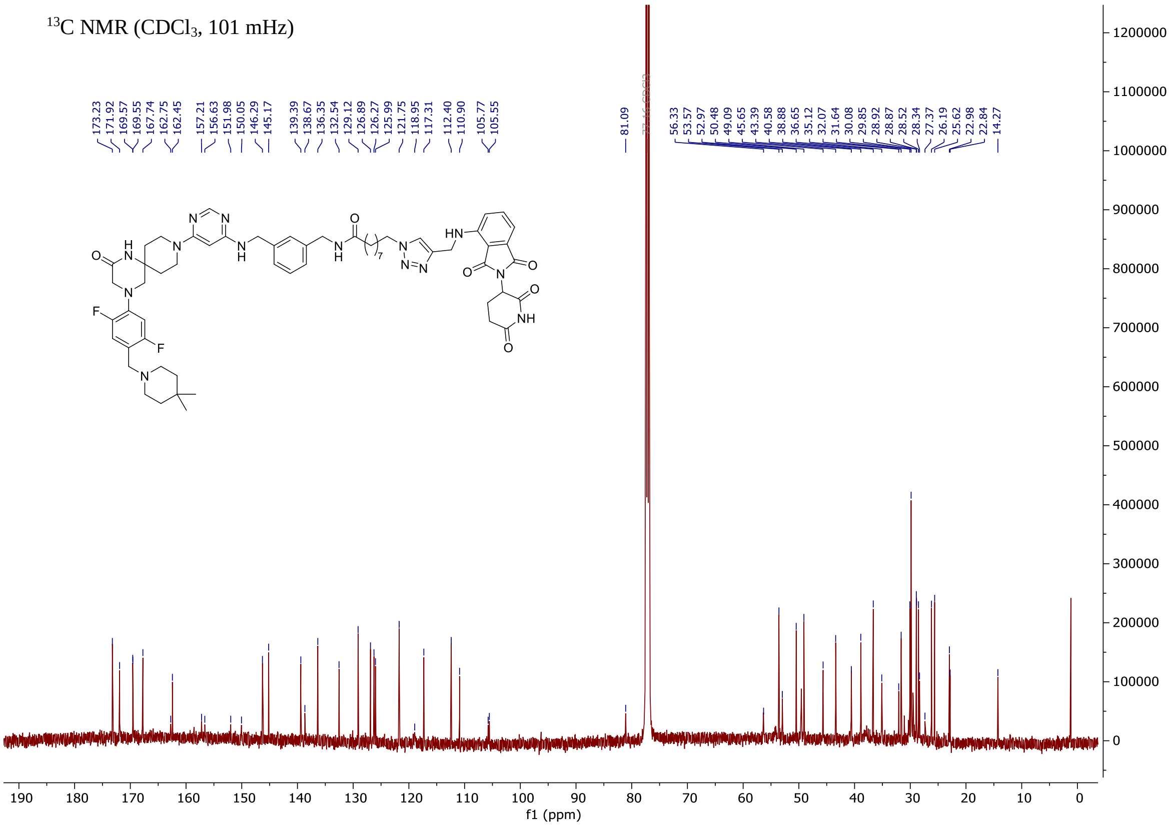

N-(3-(((6-(4-(4-((4,4-dimethylpiperidin-1-yl)methyl)-2,5-difluorophenyl)- 2-oxo-1,4,9-triazaspiro[5.5]undecan-9-yl)pyrimidin-4-yl)amino)methyl)benzyl)-11-(4-(((2-(2,6-dioxopiperidin-3-yl)-1,3-dioxoisoindolin-4-yl)amino)methyl)-1H-1,2,3-triazol-1-yl)undecanamide (20)

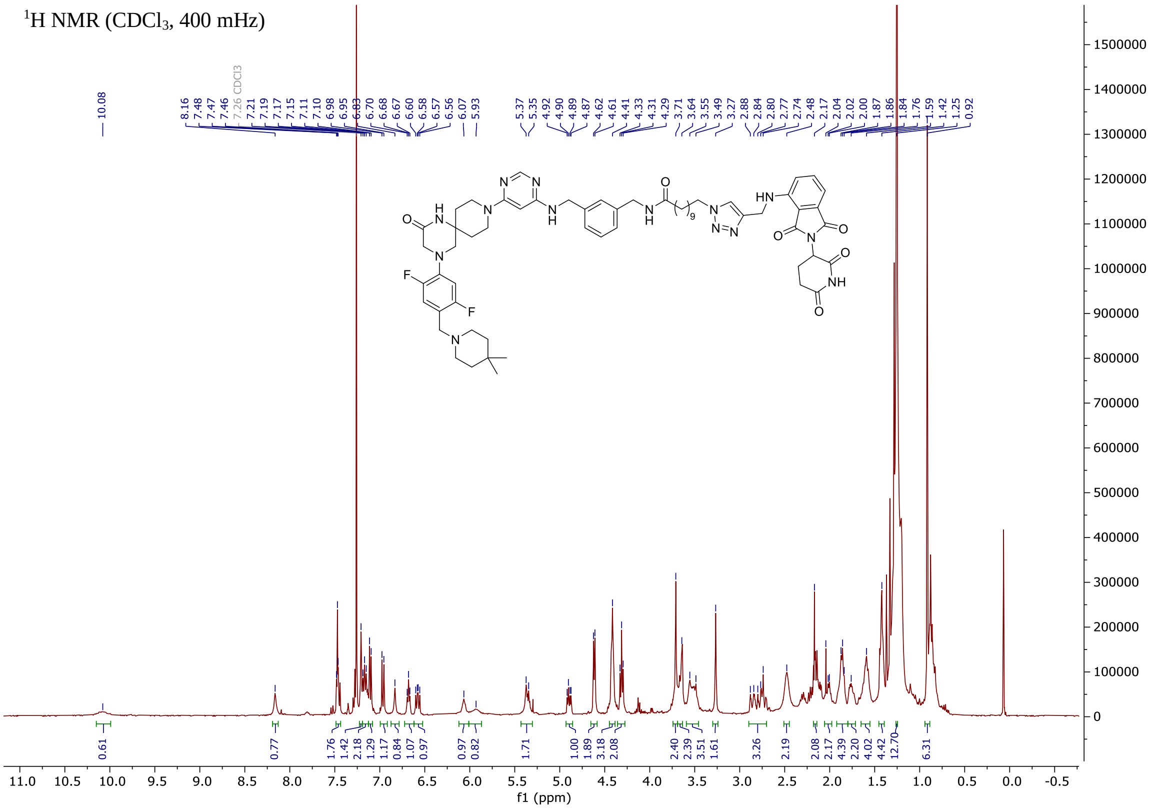

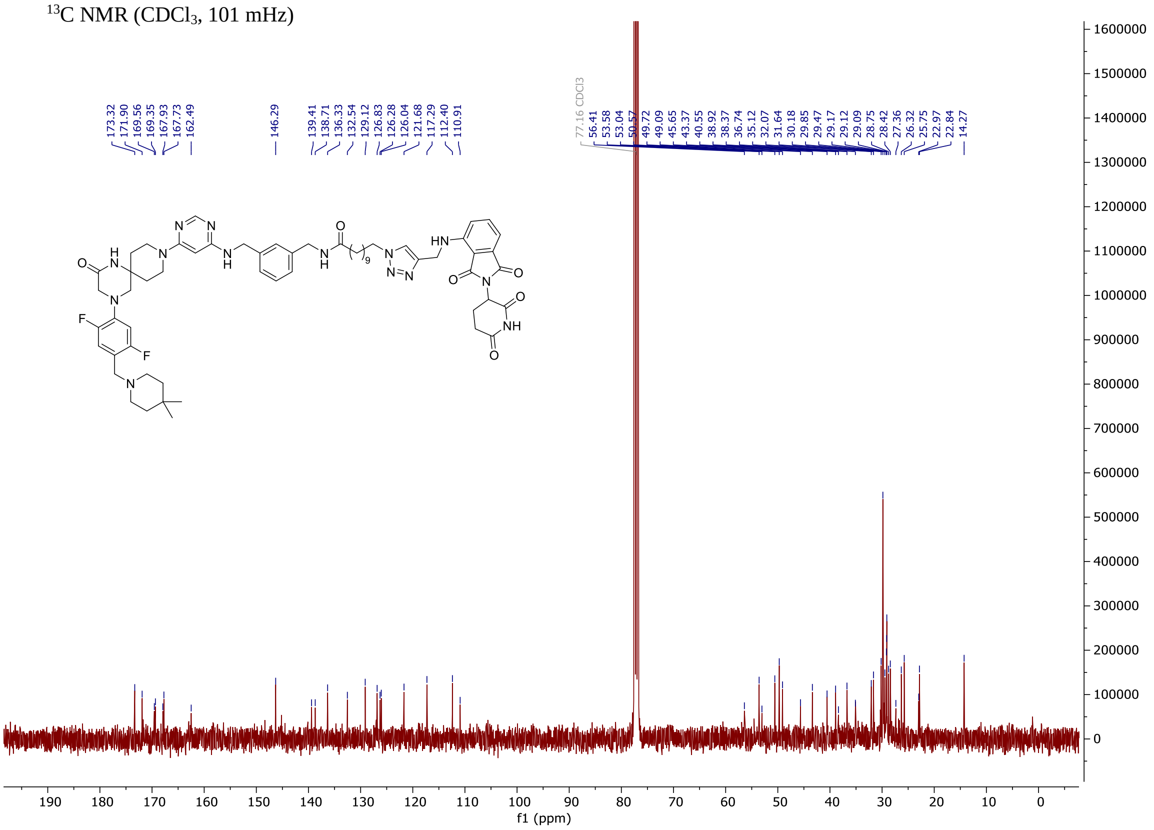

4-((2-(4-(((6-(4-(4-((4,4-dimethylpiperidin-1-yl)methyl)-2,5-difluorophenyl)-2-oxo-1,4,9-triazaspiro[5.5]undecan-9-yl)pyrimidin-4-yl)amino)methyl)piperidin-1-yl)-2-oxoethyl)amino)-2-(2,6-dioxopiperidin-3-yl)isoindoline-1,3-dione (21)

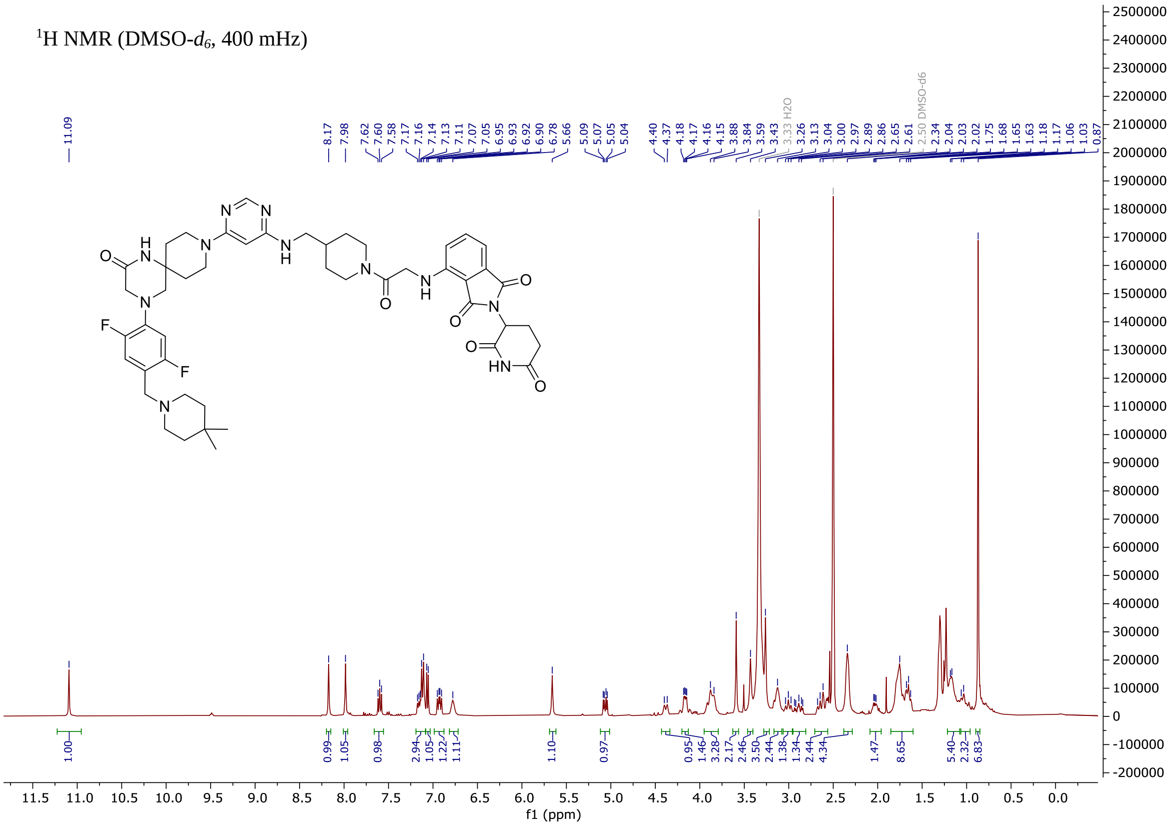

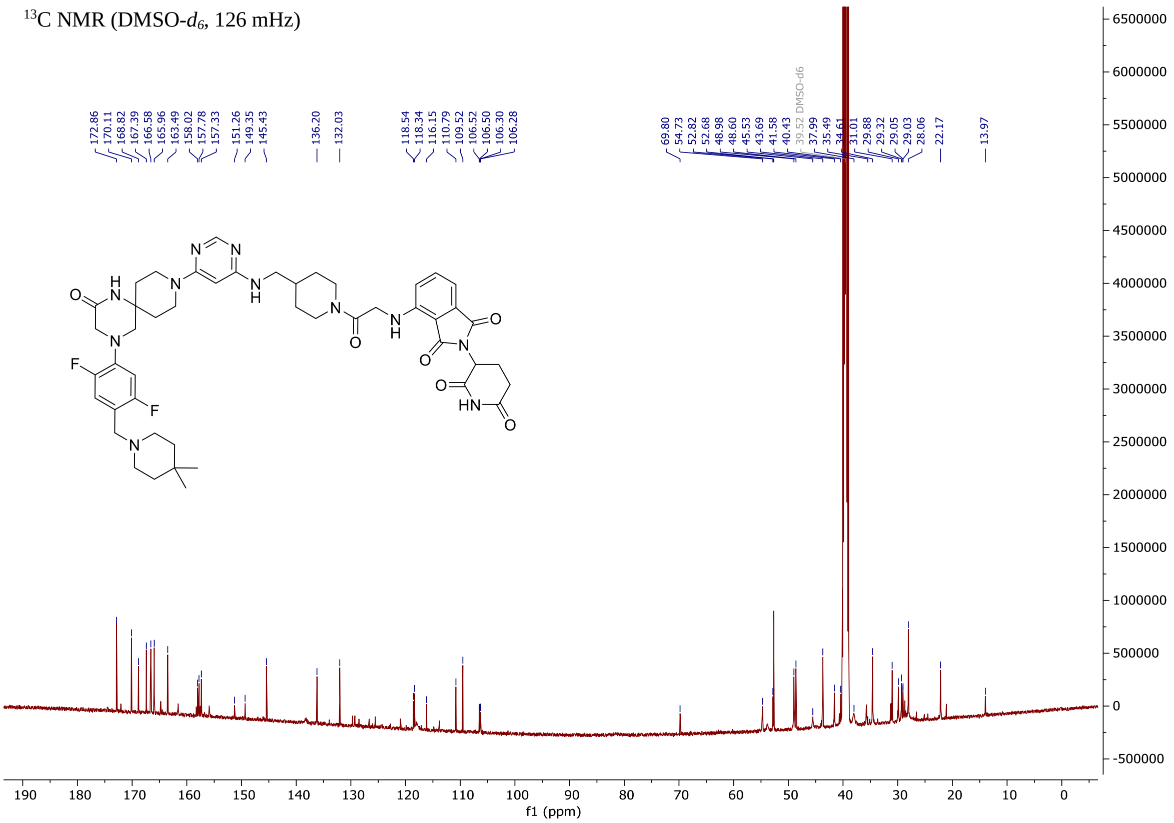

4-((4-(4-(((6-(4-(4-((4,4-dimethylpiperidin-1-yl)methyl)-2,5-difluorophenyl)- 2-oxo-1,4,9-triazaspiro[5.5]undecan-9-yl)pyrimidin-4-yl)amino)methyl)piperidin-1-yl)-4-oxobutyl)amino)-2-(2,6-dioxopiperidin-3-yl)isoindoline-1,3-dione (22)

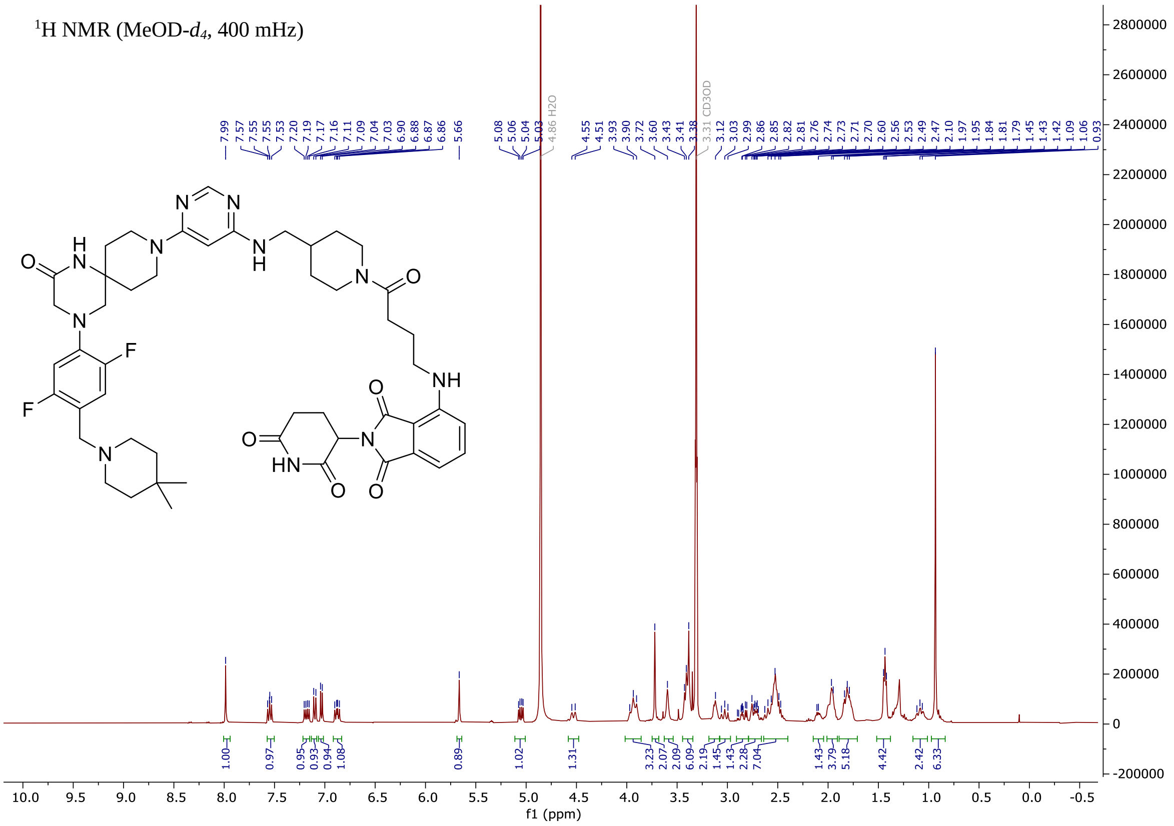

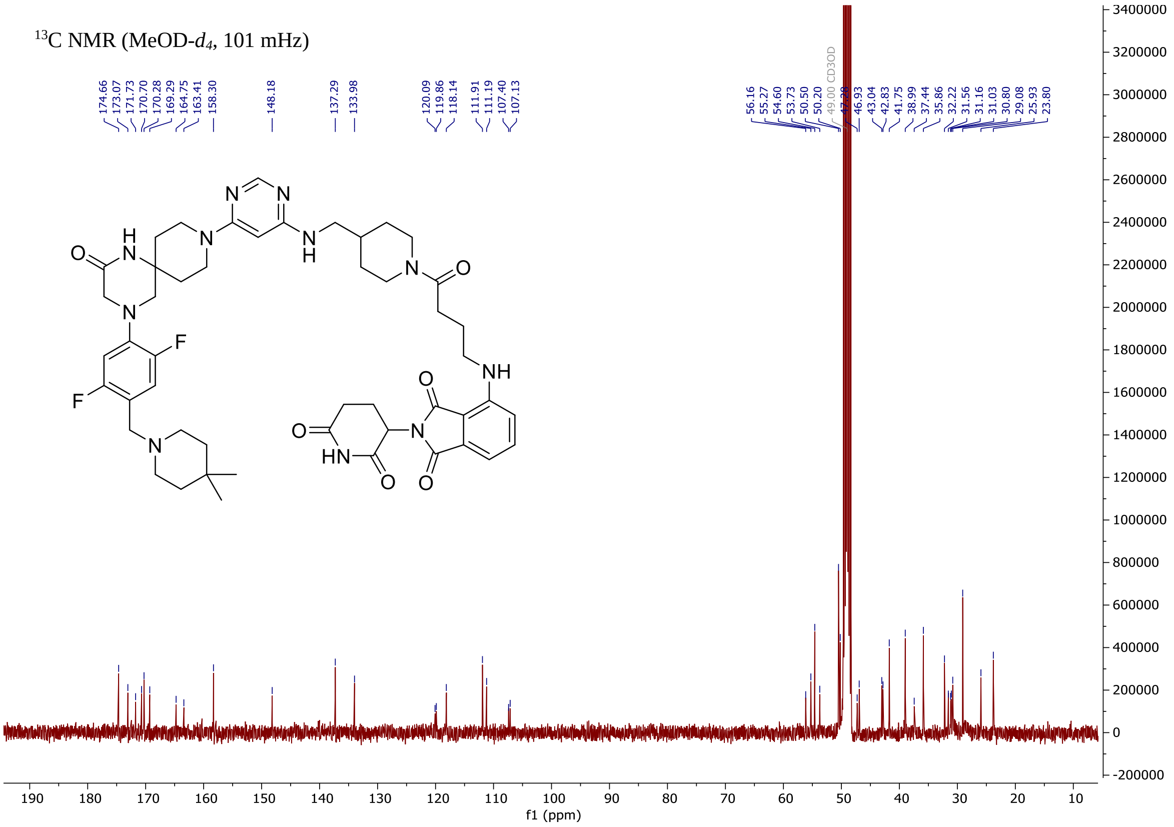

5-((4-(4-(((6-(4-(4-((4,4-dimethylpiperidin-1-yl)methyl)-2,5-difluorophenyl)-2-oxo-1,4,9-triazaspiro[5.5]undecan-9-yl)pyrimidin-4-yl)amino)methyl)piperidin-1-yl)-4-oxobutyl)amino)-2-(2,6-dioxopiperidin-3-yl)isoindoline-1,3-dione (23)

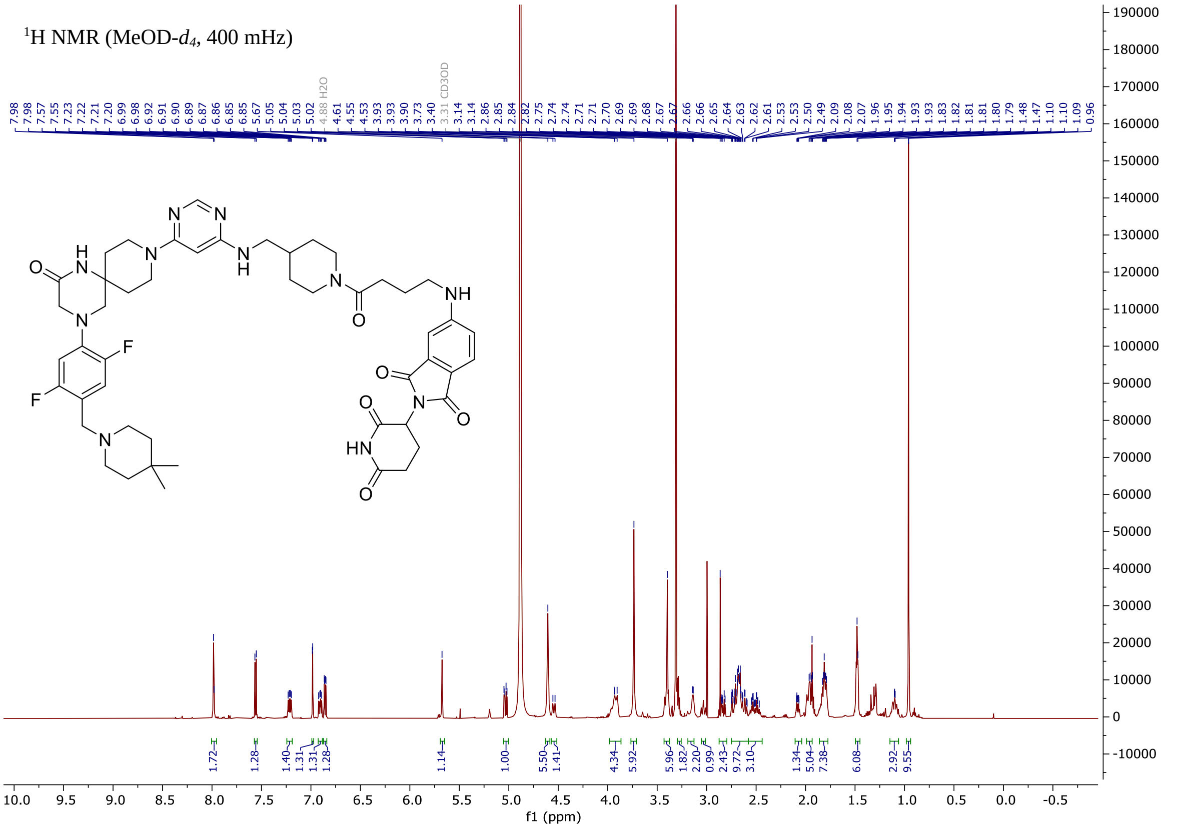

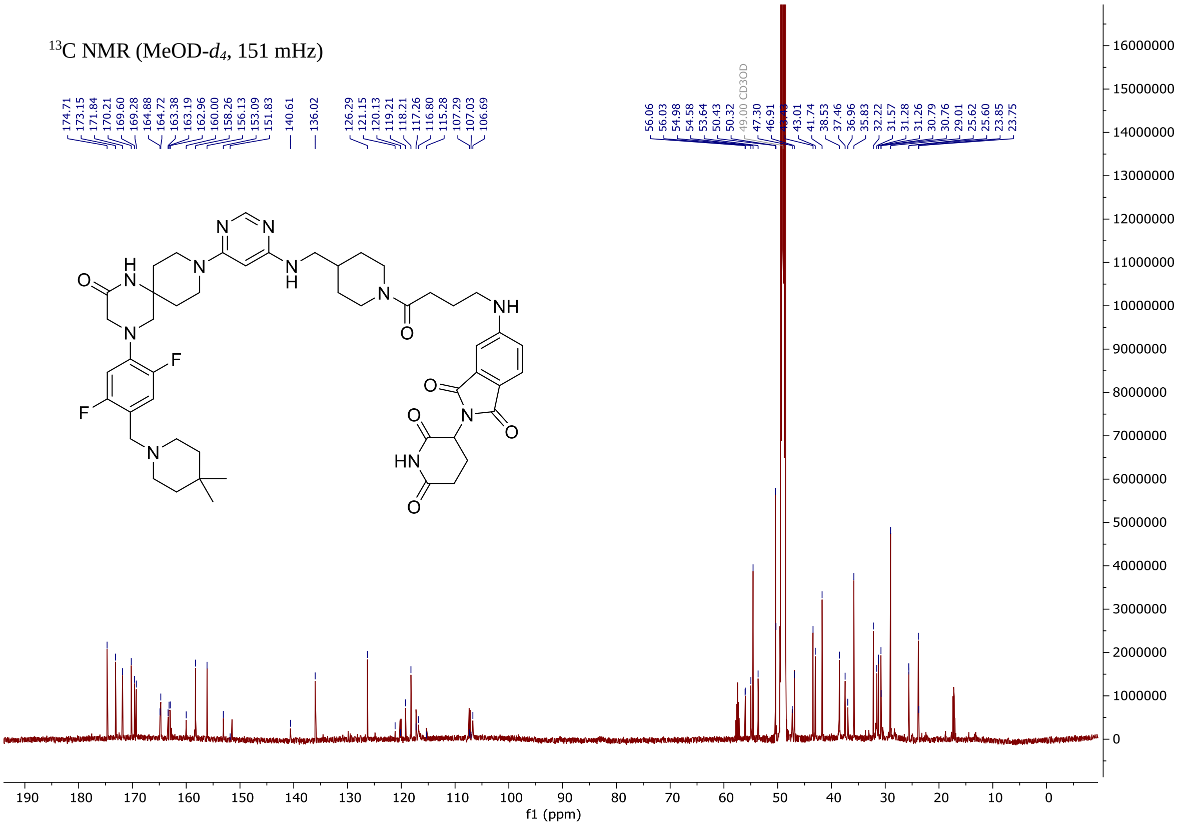

4-((6-(4-(((6-(4-(4-((4,4-dimethylpiperidin-1-yl)methyl)-2,5-difluorophenyl)-2-oxo-1,4,9-triazaspiro[5.5]undecan-9-yl)pyrimidin-4-yl)amino)methyl)piperidin-1-yl)-6-oxohexyl)amino)-2-(2,6-dioxopiperidin-3-yl)isoindoline-1,3-dione (24)

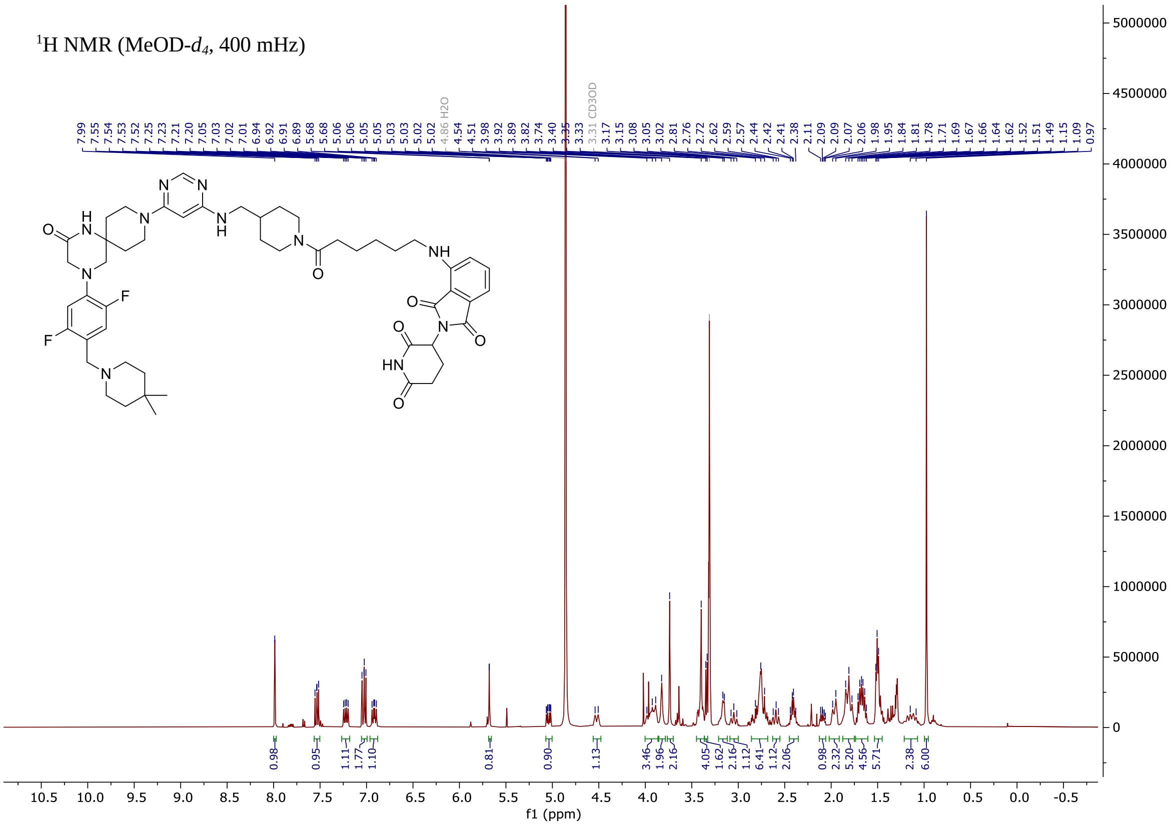

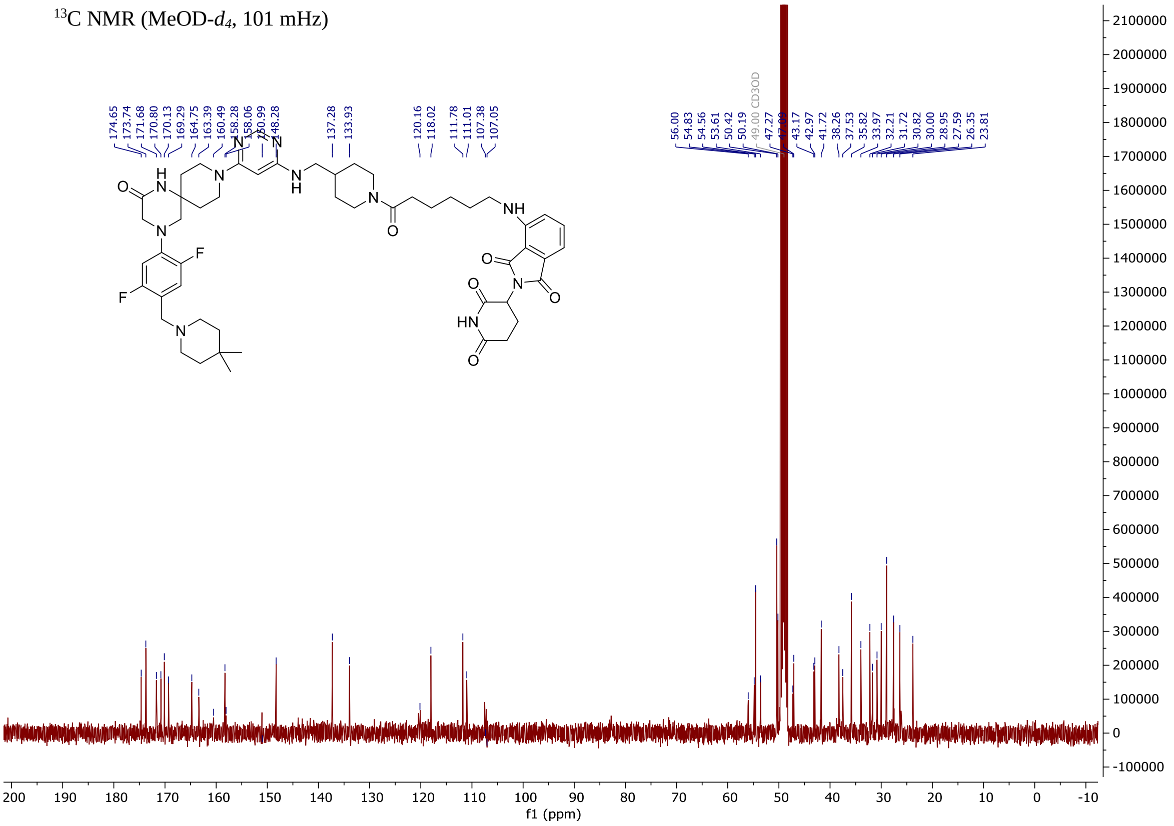

4-((8-(4-(((6-(4-(4-((4,4-dimethylpiperidin-1-yl)methyl)-2,5-difluoro phenyl)-2-oxo-1,4,9-triazaspiro[5.5]undecan-9-yl)pyrimidin-4-yl)amino)methyl)piperidin-1-yl)-8-oxooctyl)amino)-2-(2,6-dioxopiperidin-3-yl)isoindoline-1,3-dione (25)

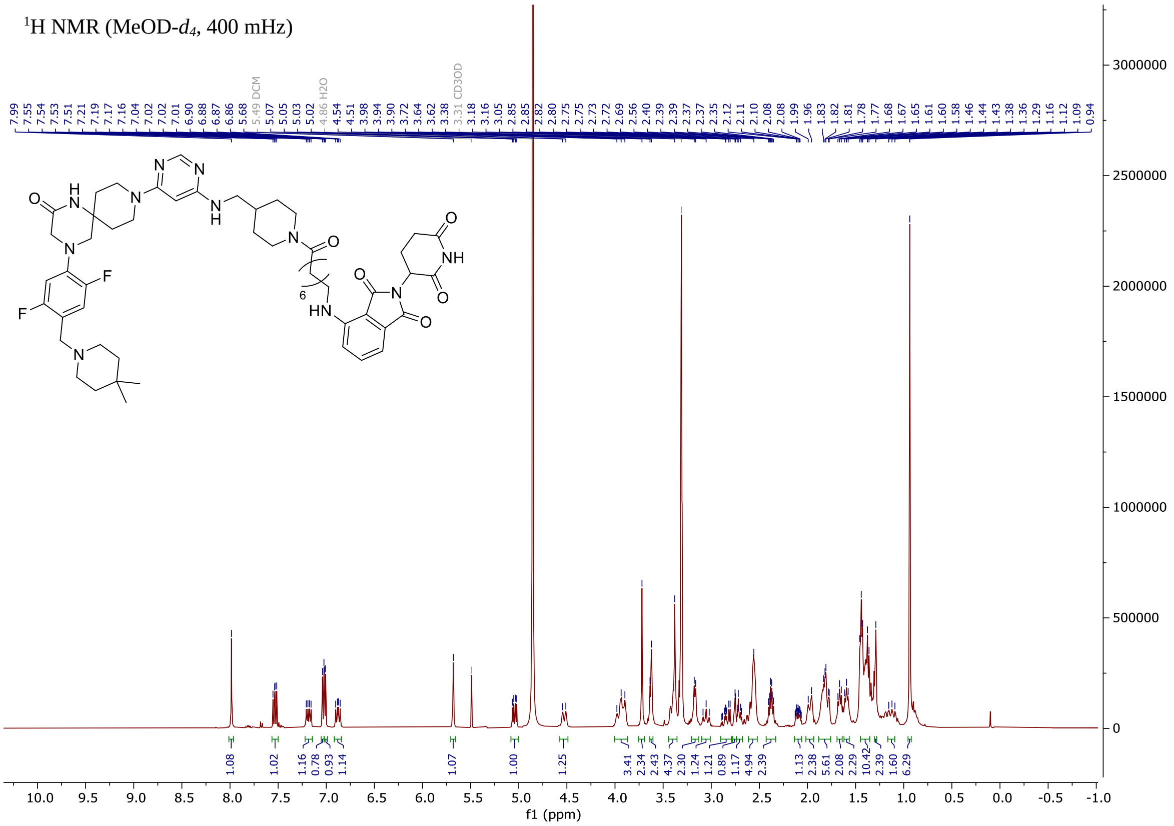

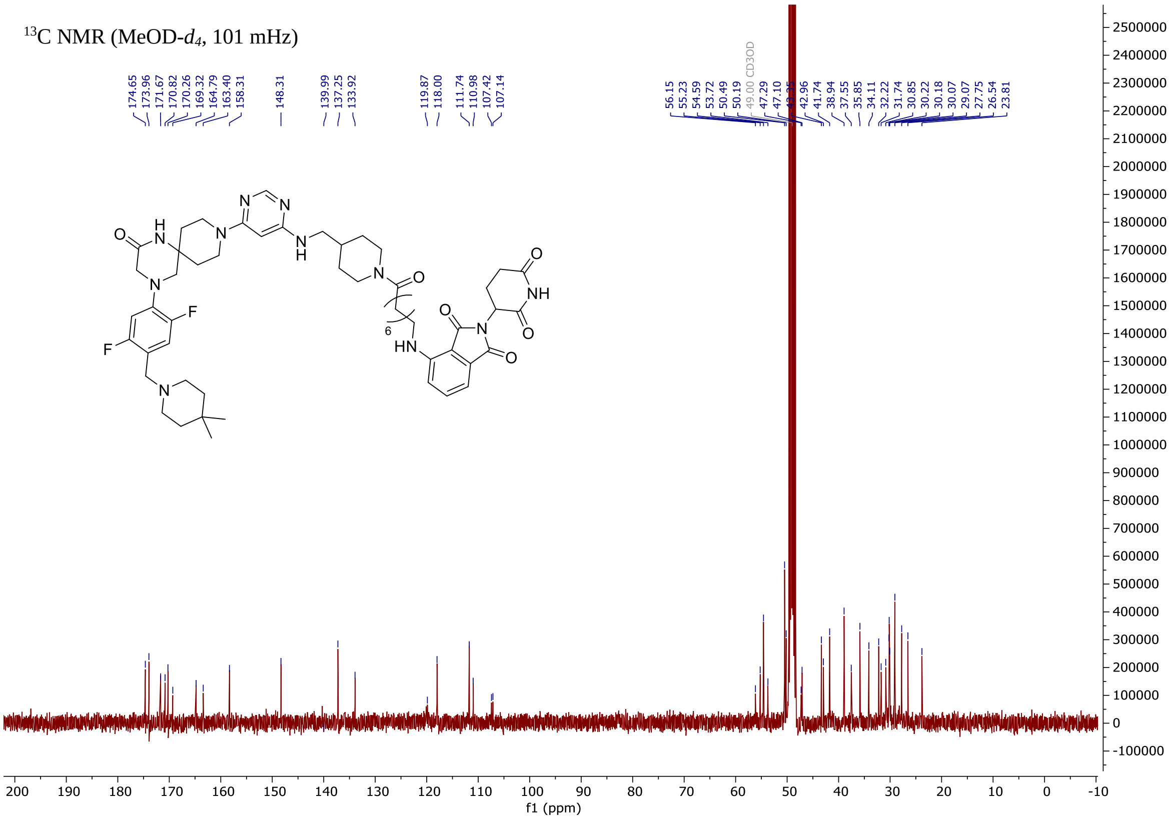

9-(4-(((6-(4-(4-((4,4-dimethylpiperidin-1-yl)methyl)-2,5-difluorophenyl)-2-oxo-1,4,9-triazaspiro[5.5]undecan-9-yl)pyrimidin-4-yl)amino)methyl)-1H-1,2,3-triazol-1-yl)-N-(2-(2,6-dioxopiperidin-3-yl)-1-oxoisoindolin-4-yl)nonanamide (26)

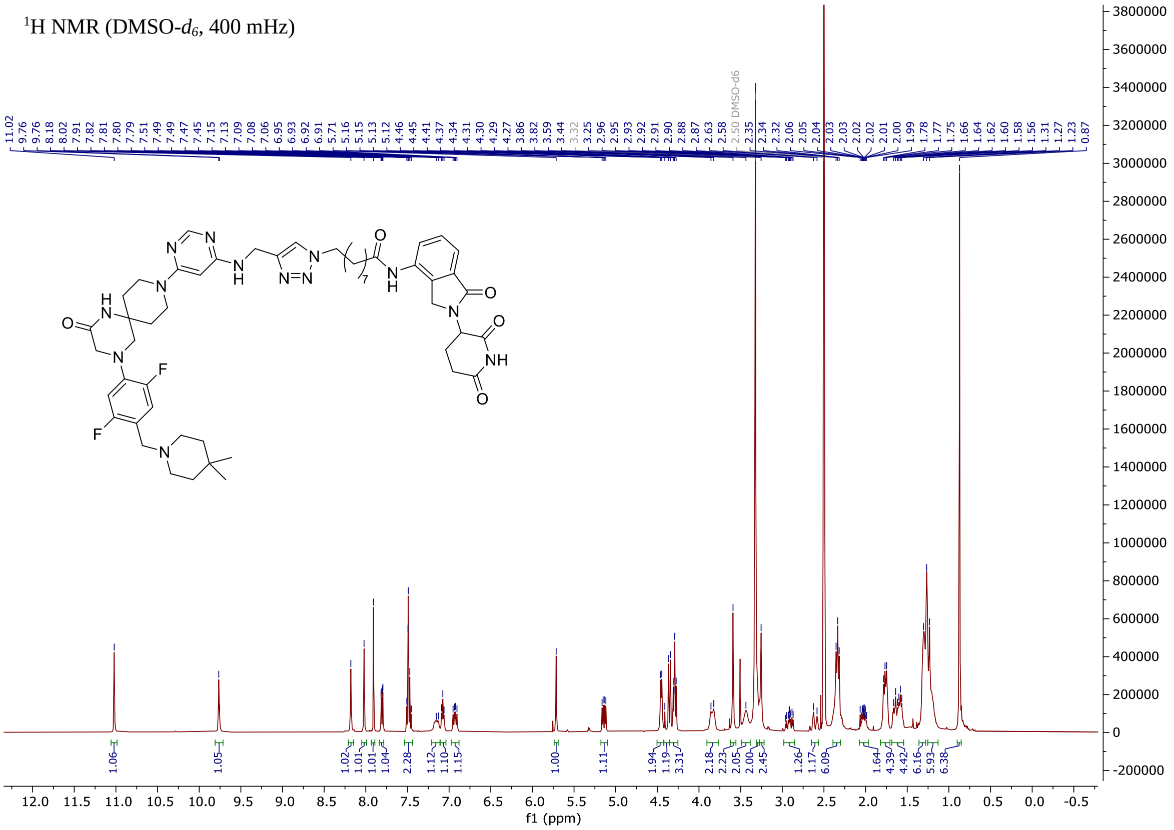

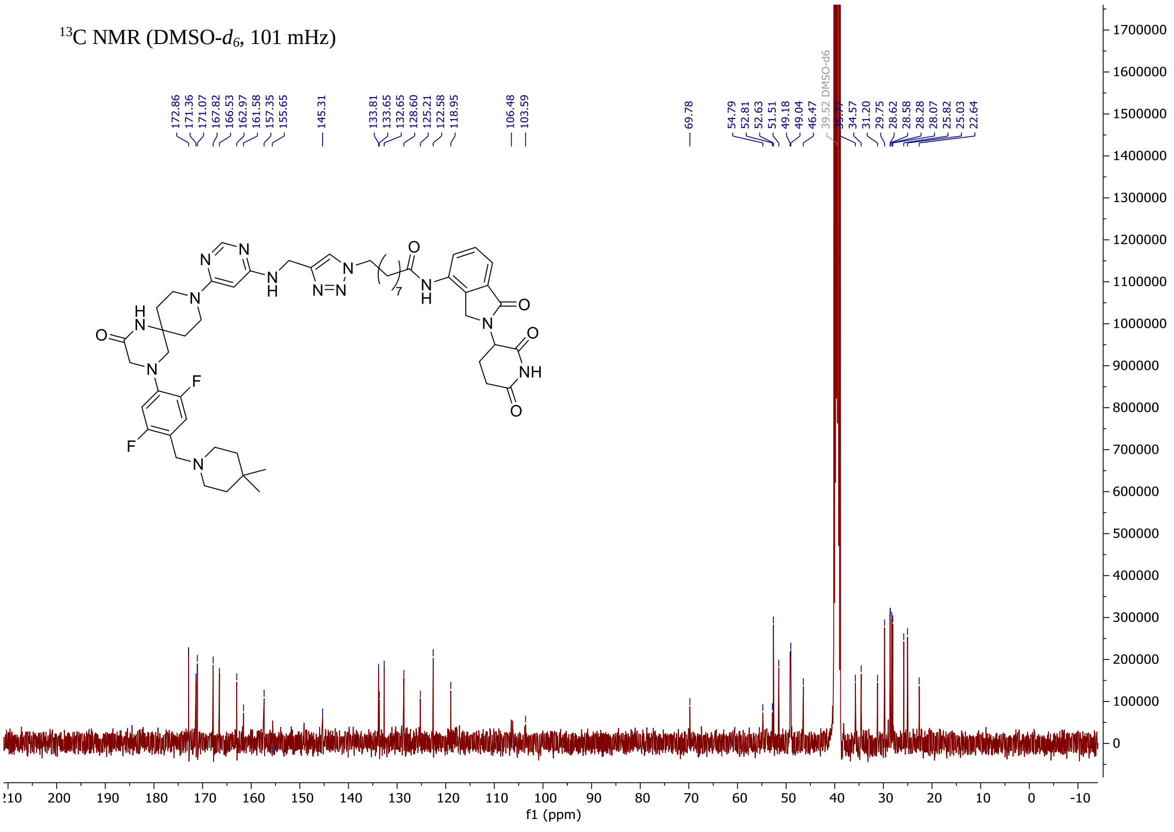

11-(4-(((6-(4-(4-((4,4-dimethylpiperidin-1-yl)methyl)-2,5-difluorophenyl)-2-oxo-1,4,9-triazaspiro[5.5]undecan-9-yl)pyrimidin-4-yl)amino)methyl)-1H-1,2,3-triazol-1-yl)-N-(2-(2,6-dioxopiperidin-3-yl)-3-oxoisoindolin-5-yl)undecanamide (27)

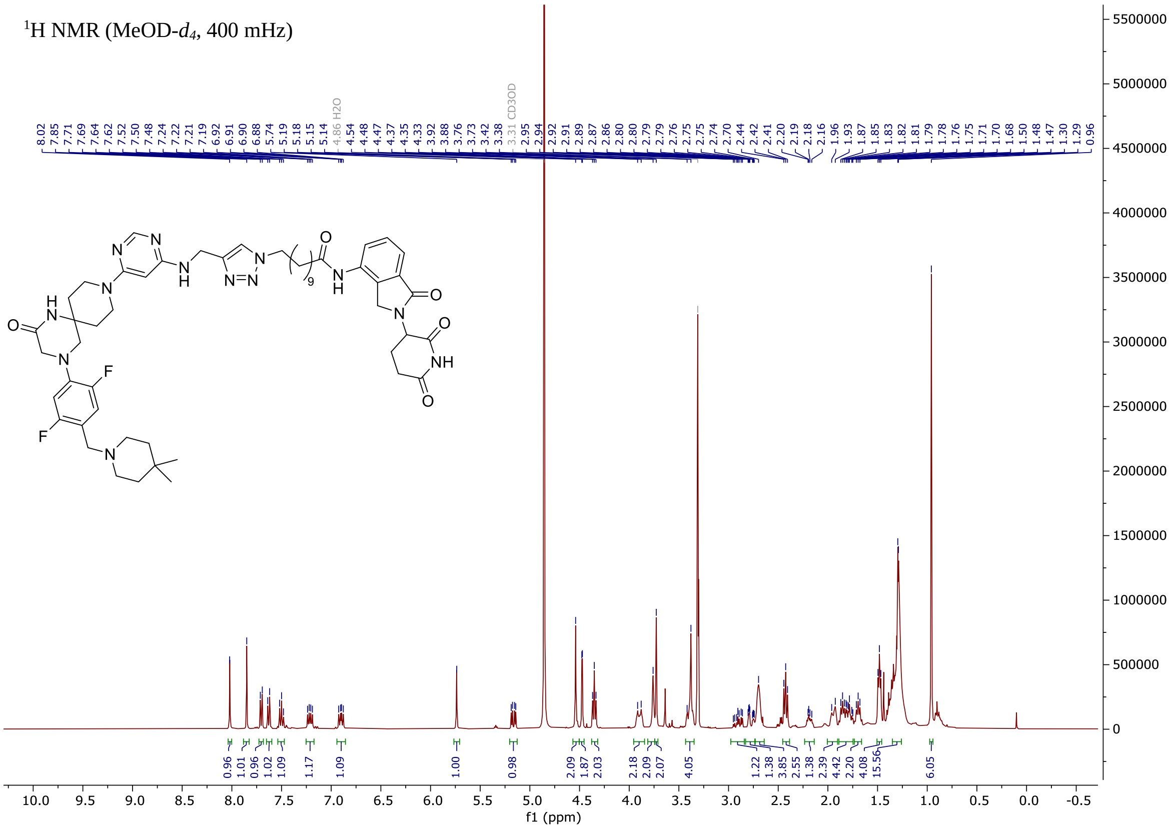

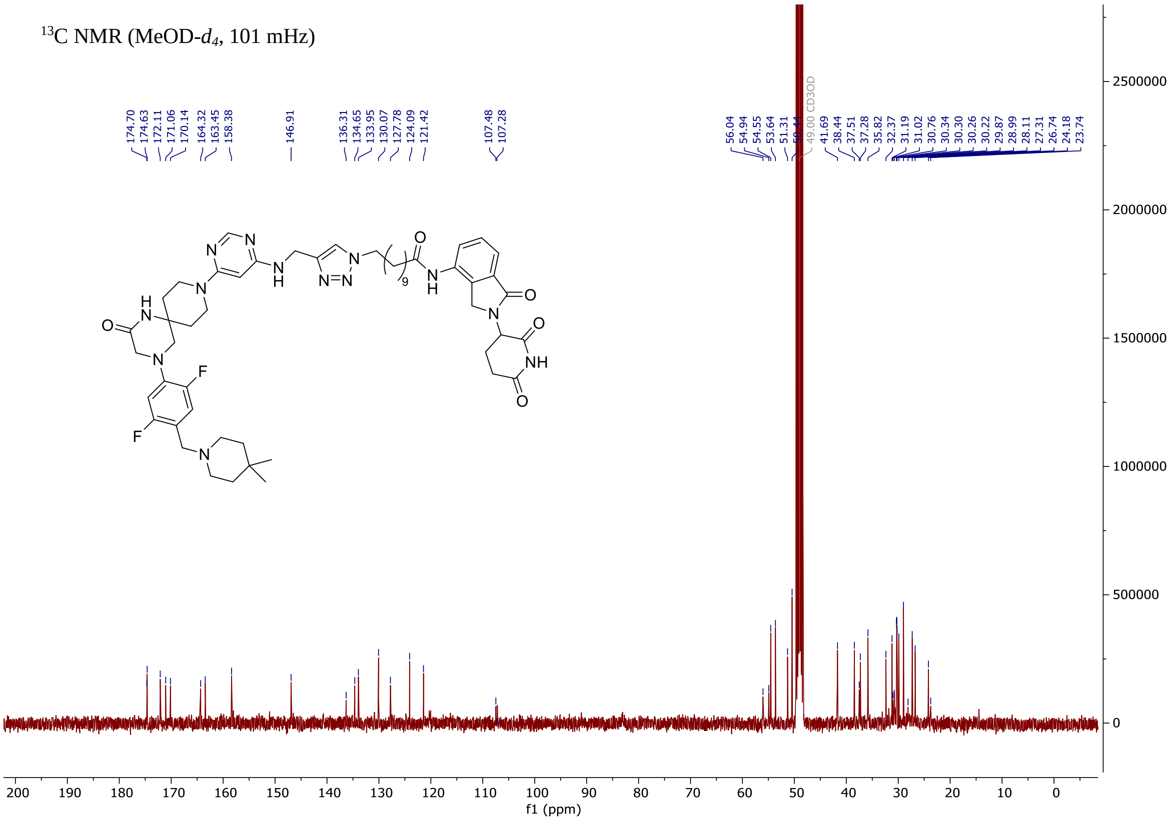

4-(((1-(12-((6-(4-(4-((4,4-dimethylpiperidin-1-yl)methyl)-2,5-difluorophenyl)-2-oxo-1,4,9-triazaspiro[5.5]undecan-9-yl)pyrimidin-4-yl)amino)dodecyl)-1H-1,2,3-triazol-4-yl)methyl)amino)-2-(2,6-dioxopiperidin-3-yl)isoindoline-1,3-dione (28)

^{1}H NMR (DMSO-d_6, 400 mHz)

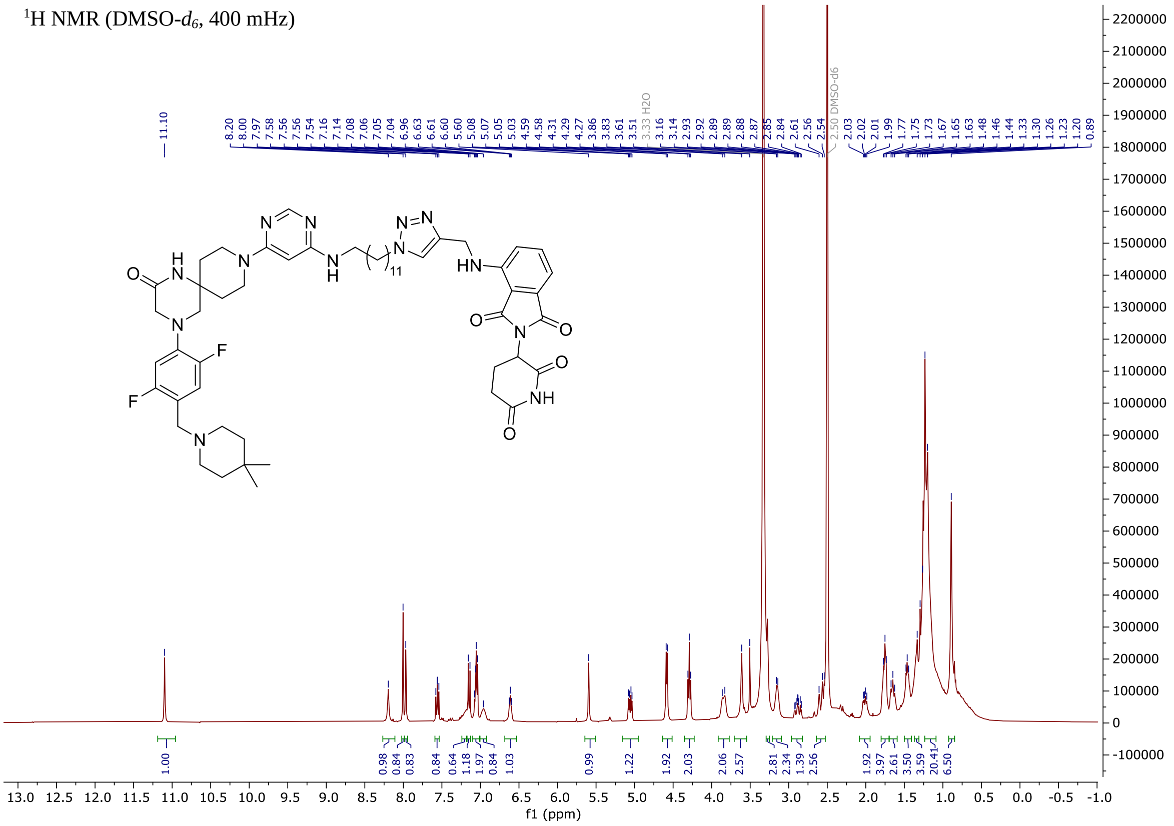

^{13}C NMR (DMSO-d_6, 101 mHz)

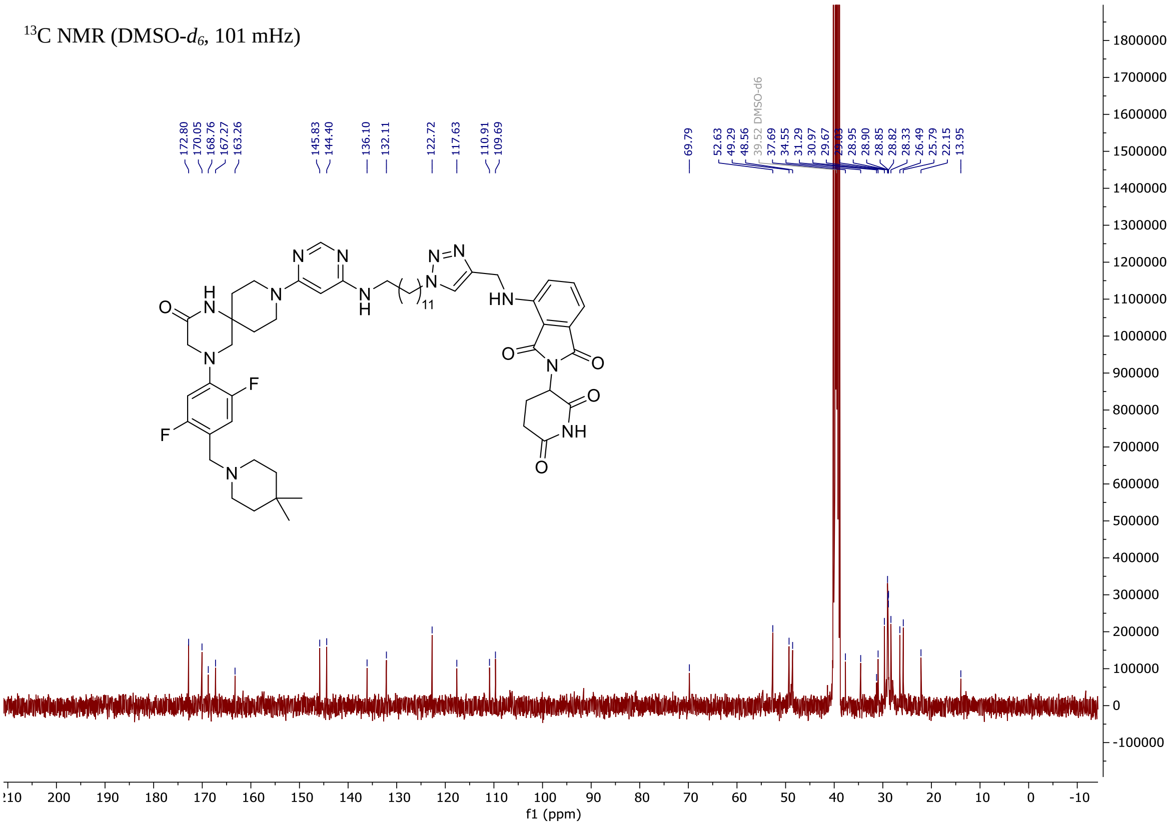

N*-(3-(((6-(4-(4-((4,4-dimethylpiperidin-1-yl)methyl)-2,5-difluorophenyl)- 2-oxo-1,4,9-triazaspiro[5.5]undecan-9-yl)pyrimidin-4-yl)4-(2-aminoethyl)piperazin)-4-((2-(2,6-dioxopiperidin-3-yl)-1,3-dioxoisoindolin-4-yl)amino)butanamide (29)

^{1}H NMR (MeOD-*d*$_4$, 500 MHz)

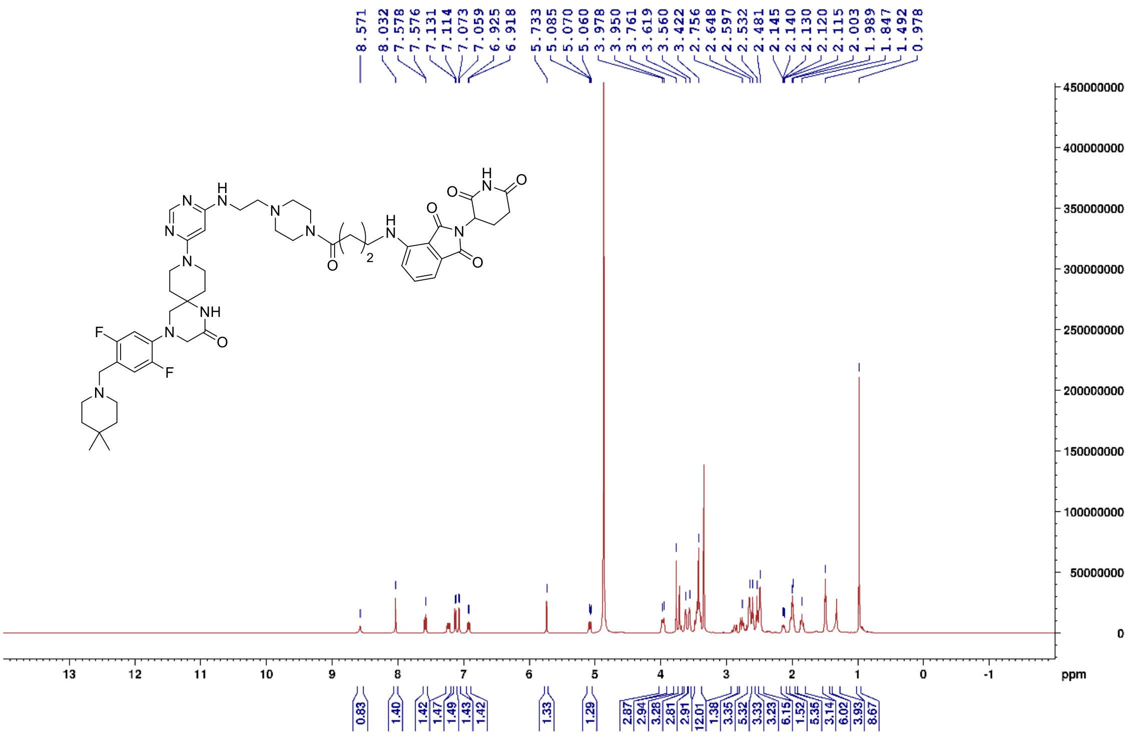

^{13}C NMR (MeOD-*d*$_4$, 101 MHz)

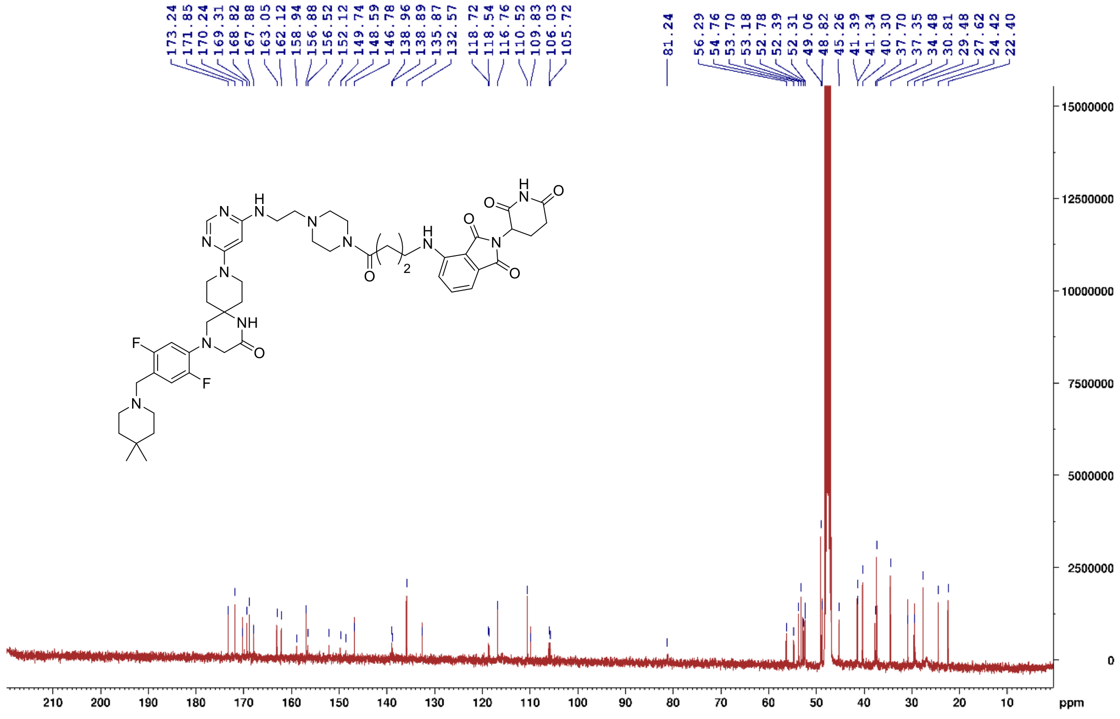

N-(3-(((6-(4-(4-((4,4-dimethylpiperidin-1-yl)methyl)-2,5-difluorophenyl)- 2-oxo-1,4,9-triazaspiro[5.5]undecan-9-yl)pyrimidin-4-yl)4-(2-aminoethyl)piperazin)-6-((2-(2,6-dioxopiperidin-3-yl)-1,3-dioxoisoindolin-4-yl)amino)hexanamide (30)

^{1}H NMR (MeOD-d_4, 500 MHz)

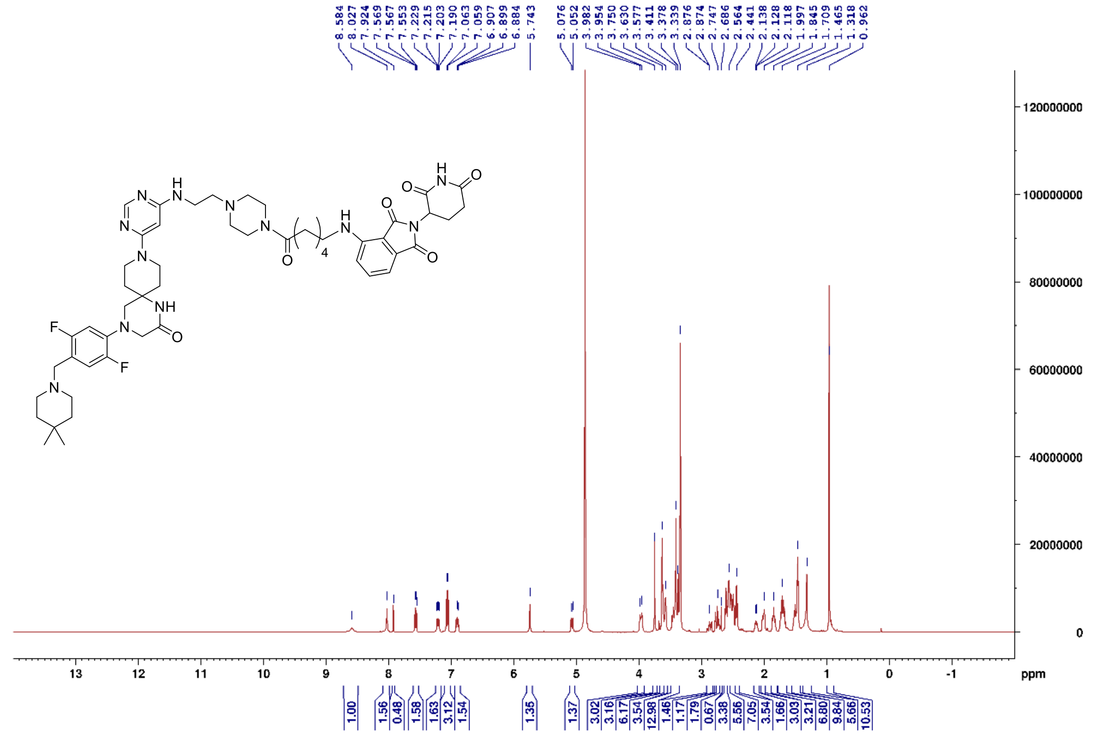

^{13}C NMR (MeOD-d_4, 400 MHz)

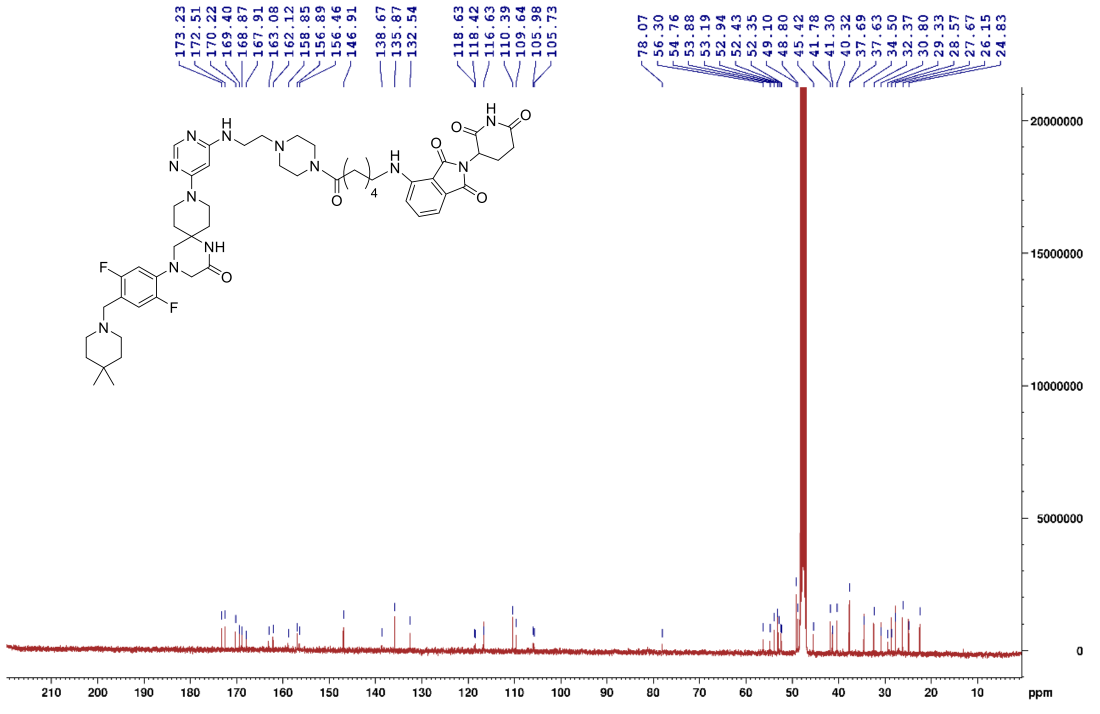

N-(3-(((6-(4-(4-((4,4-dimethylpiperidin-1-yl)methyl)-2,5-difluorophenyl)- 2-oxo-1,4,9-triazaspiro[5.5]undecan-9-yl)pyrimidin-4-yl)4-(2-aminoethyl)piperazin)-8-((2-(2,6-dioxopiperidin-3-yl)-1,3-dioxoisoindolin-4-yl)amino)octanamide (31)

^{1}H NMR (MeOD-*d*$_4$, 500 MHz)

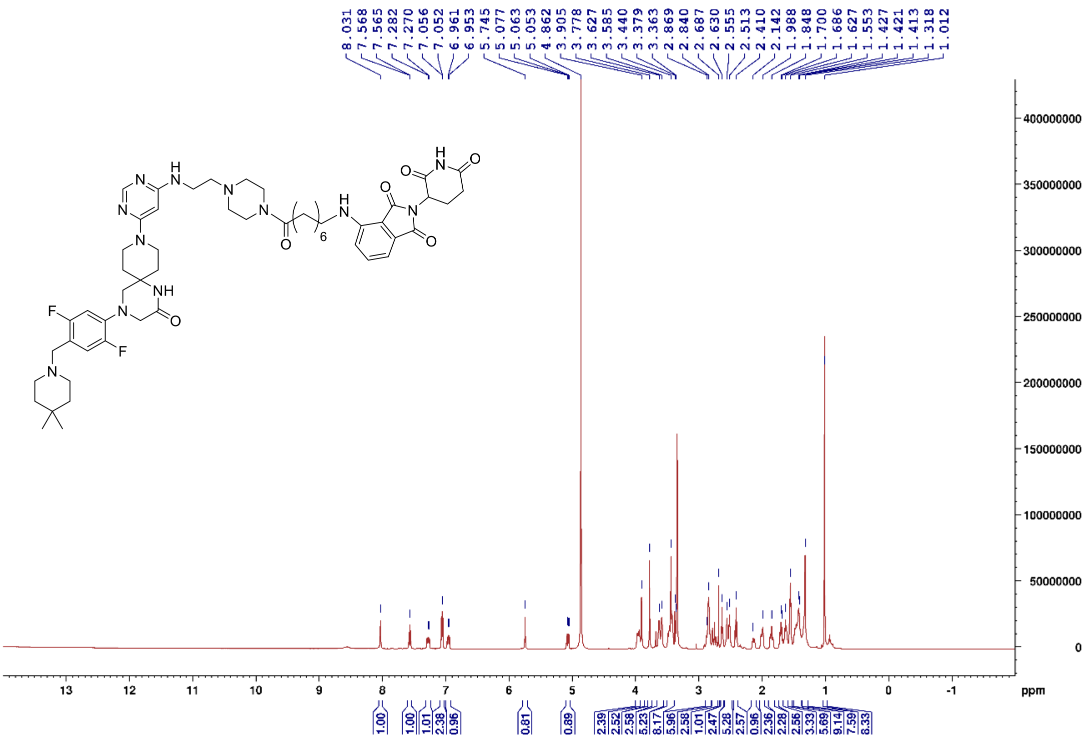

^{13}C NMR (MeOD-*d*$_4$, 125 MHz)

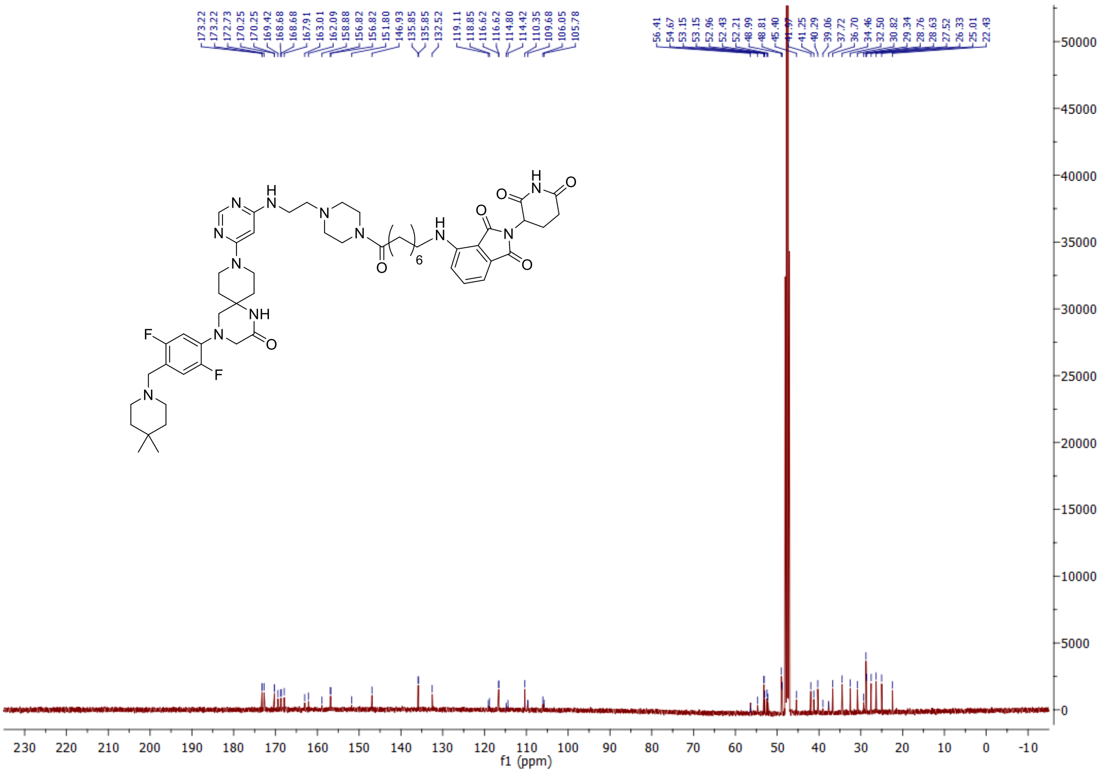

4-(4-(2-((6-(4-(4-((4,4-dimethylpiperidin-1-yl)methyl)-2,5-difluorophenyl)-2-oxo-1,4,9-triazaspiro[5.5]undecan-9-yl)pyrimidin-4-yl)amino)ethyl)piperazin-1-yl)-2-(2,6-dioxopiperidin-3-yl)isoindoline-1,3-dione (32)

^{1}H NMR ($CDCl_3$, 400 MHz)

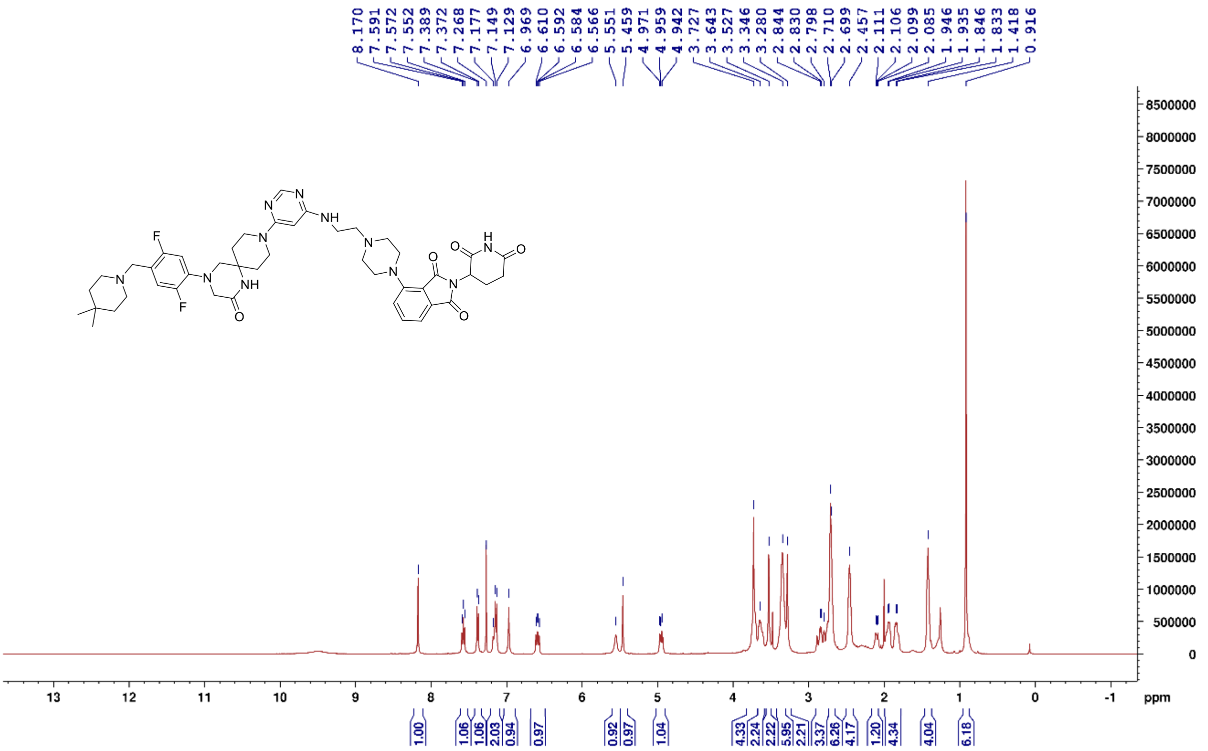

^{13}C NMR ($CDCl_3$, 101 MHz)

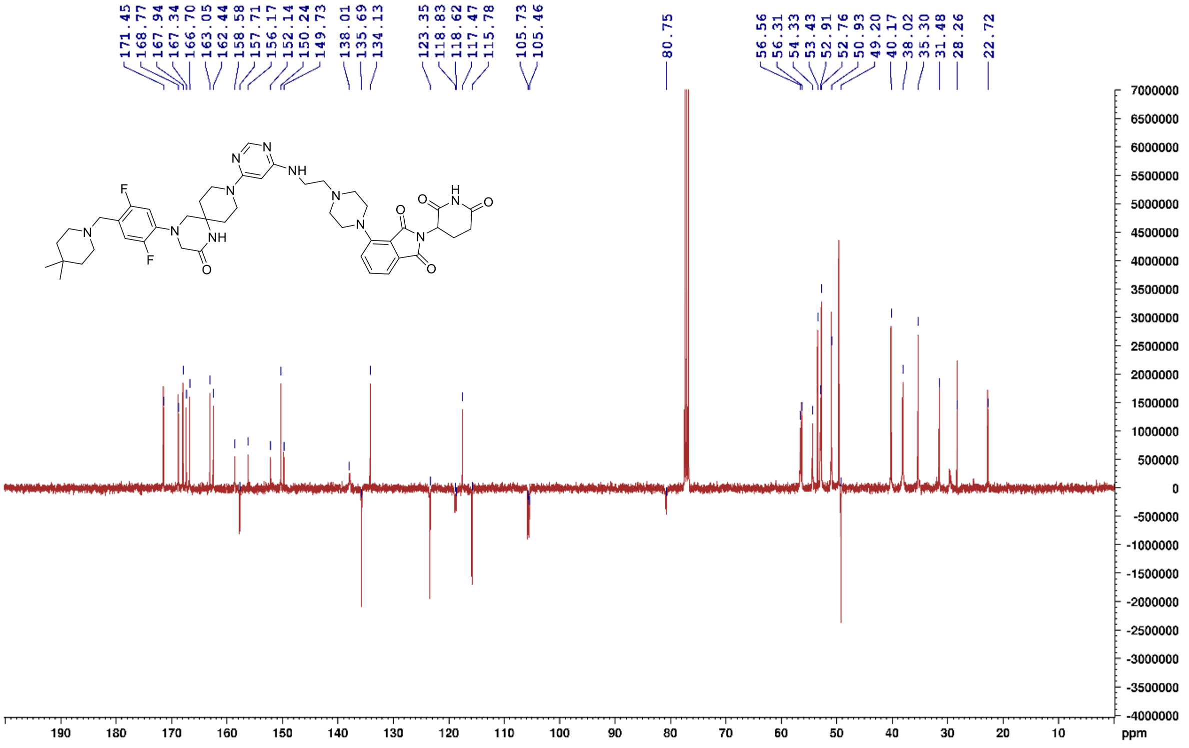

4-((3-(4-(5-(4-(2-((6-(4-(4-((4,4-dimethylpiperidin-1-yl)methyl)-2,5-difluorophenyl)-2-oxo-1,4,9-triazaspiro[5.5]undecan-9-yl)pyrimidin-4-yl)amino)ethyl)piperazin-1-yl)pyrimidin-2-yl)piperazin-1-yl)-3-oxopropyl)amino)-2-(2,6-dioxopiperidin-3-yl)isoindoline-1,3-dione (33)

^{1}H NMR ($CDCl_3$, 400 MHz)

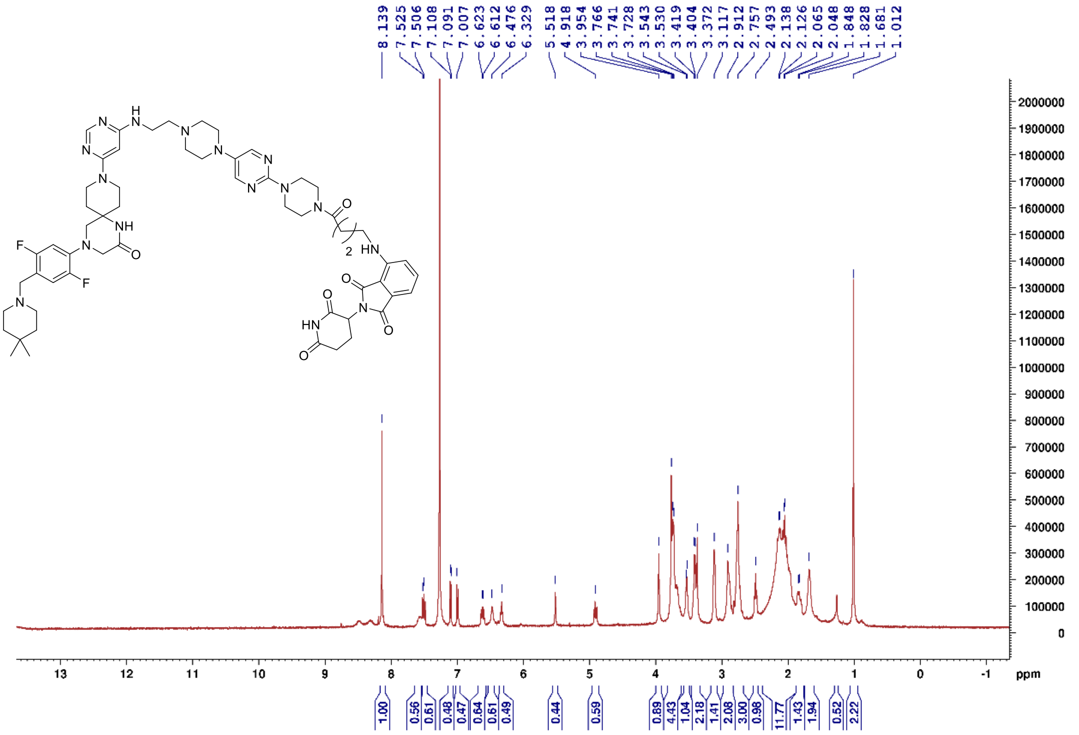

^{13}C NMR (DMSO-d_6, 176 MHz)

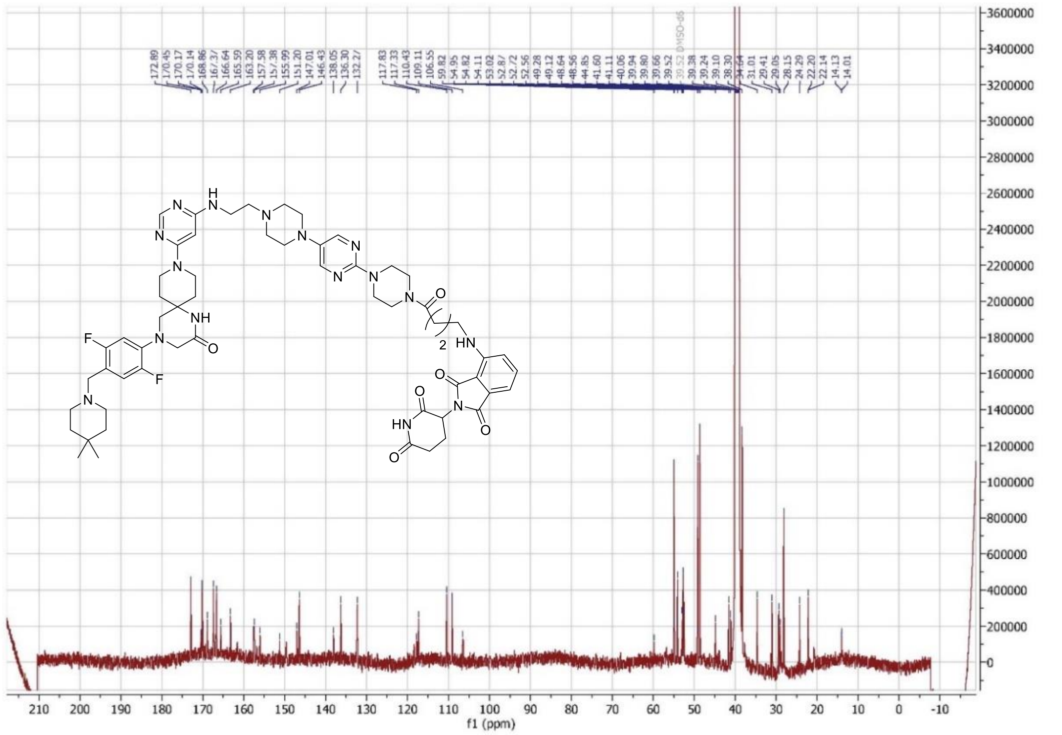

4-(4-(4-(((6-(4-(4-((4,4-dimethylpiperidin-1-yl)methyl)-2,5-difluorophenyl)-2-oxo-1,4,9-triazaspiro[5.5]undecan-9-yl)pyrimidin-4-yl)amino)methyl)phenyl)piperazin-1-yl)-2-(2,6-dioxopiperidin-3-yl)isoindoline-1,3-dione (34)

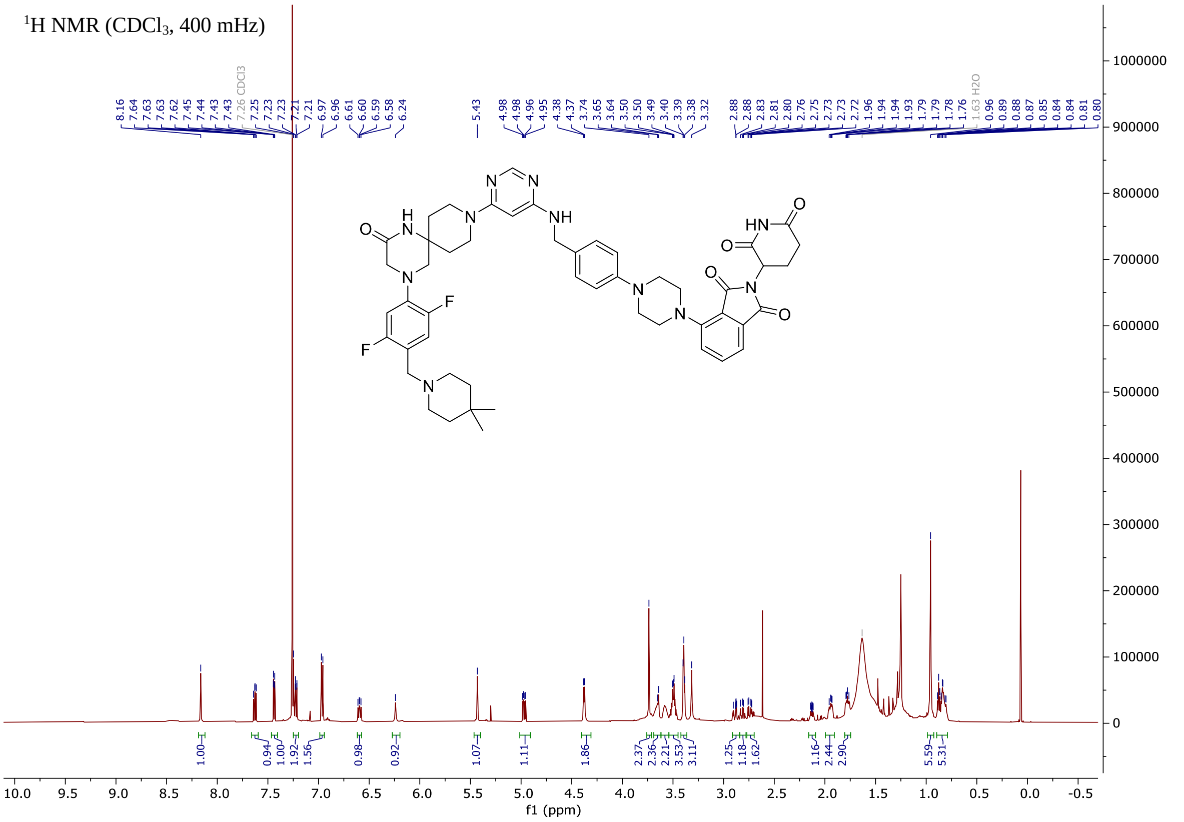

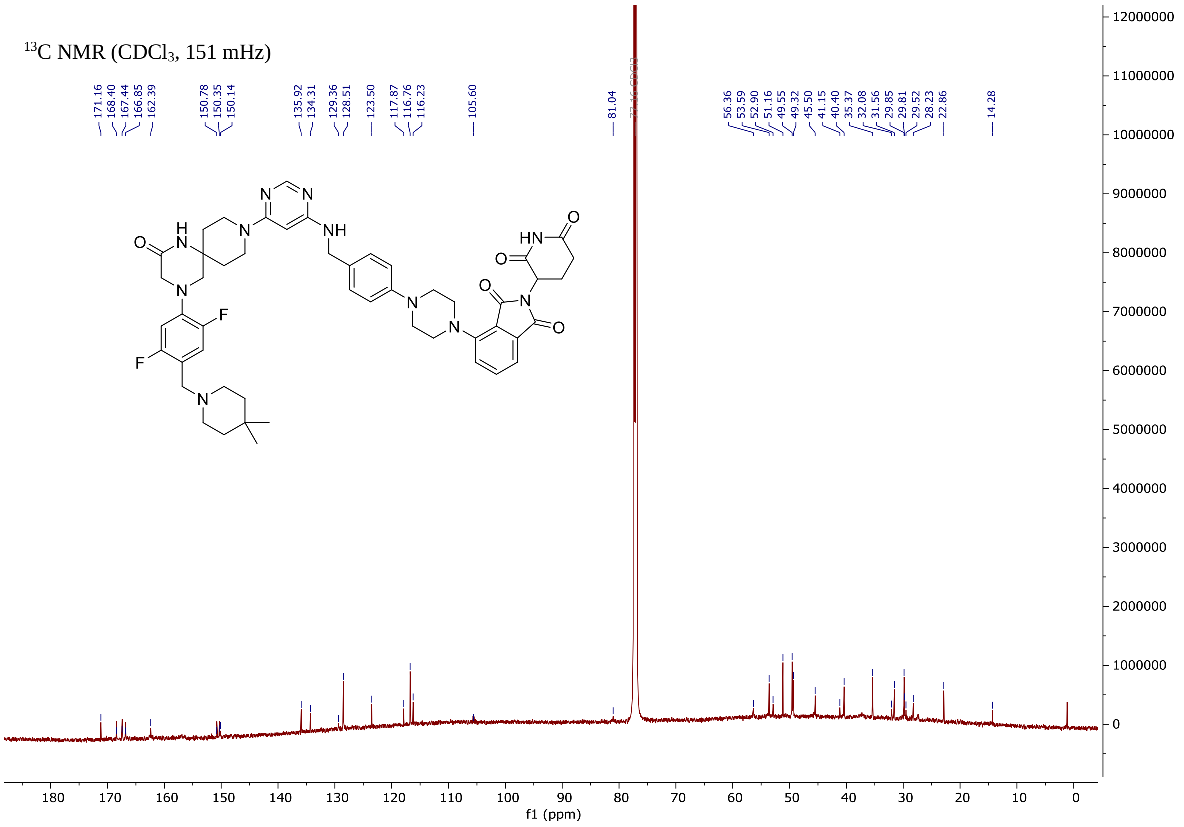

4-(4-(2-(4-(((6-(4-(4-((4,4-dimethylpiperidin-1-yl)methyl)-2,5-difluorophenyl)-2-oxo-1,4,9-triazaspiro[5.5]undecan-9-yl)pyrimidin-4-yl)amino)methyl)piperidin-1-yl)-2-oxoethyl)piperazin-1-yl)-2-(2,6-dioxopiperidin-3-yl)isoindoline-1,3-dione (35)

^{1}H NMR (MeOD-d_4, 400 mHz)

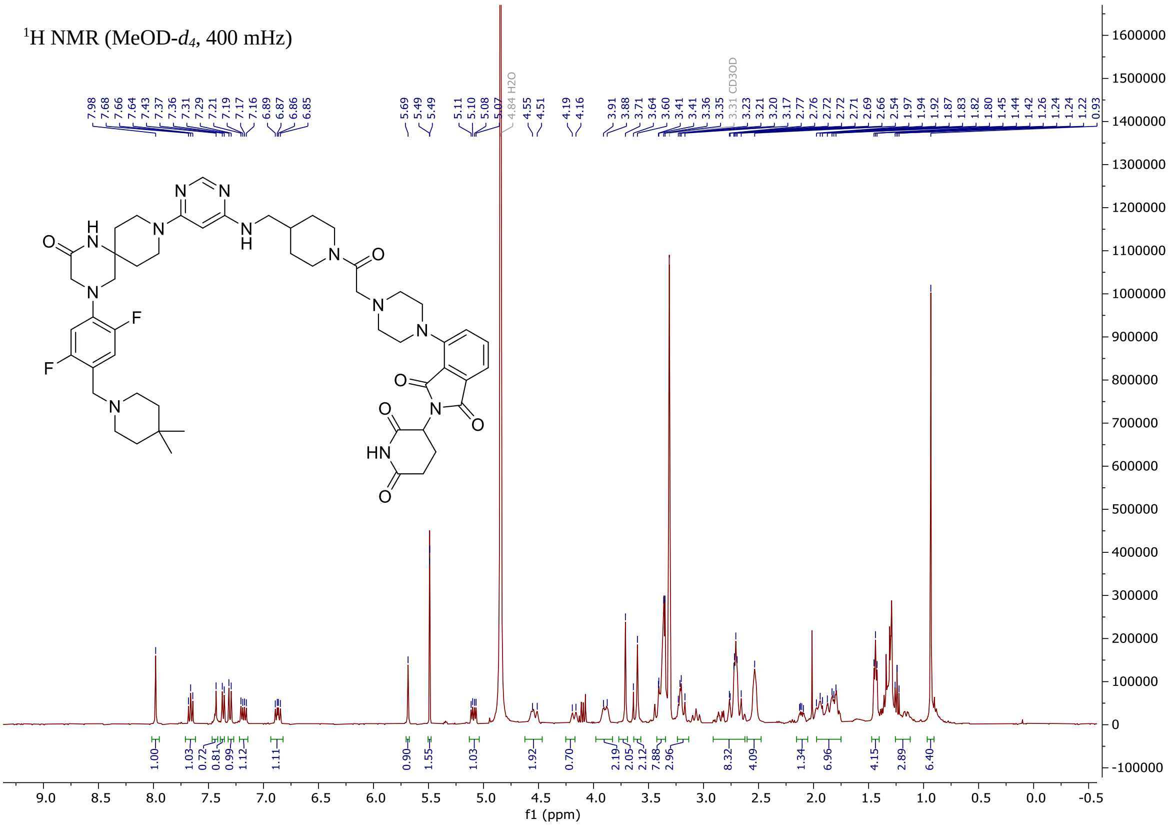

^{13}C NMR (MeOD-d_4, 126 mHz)

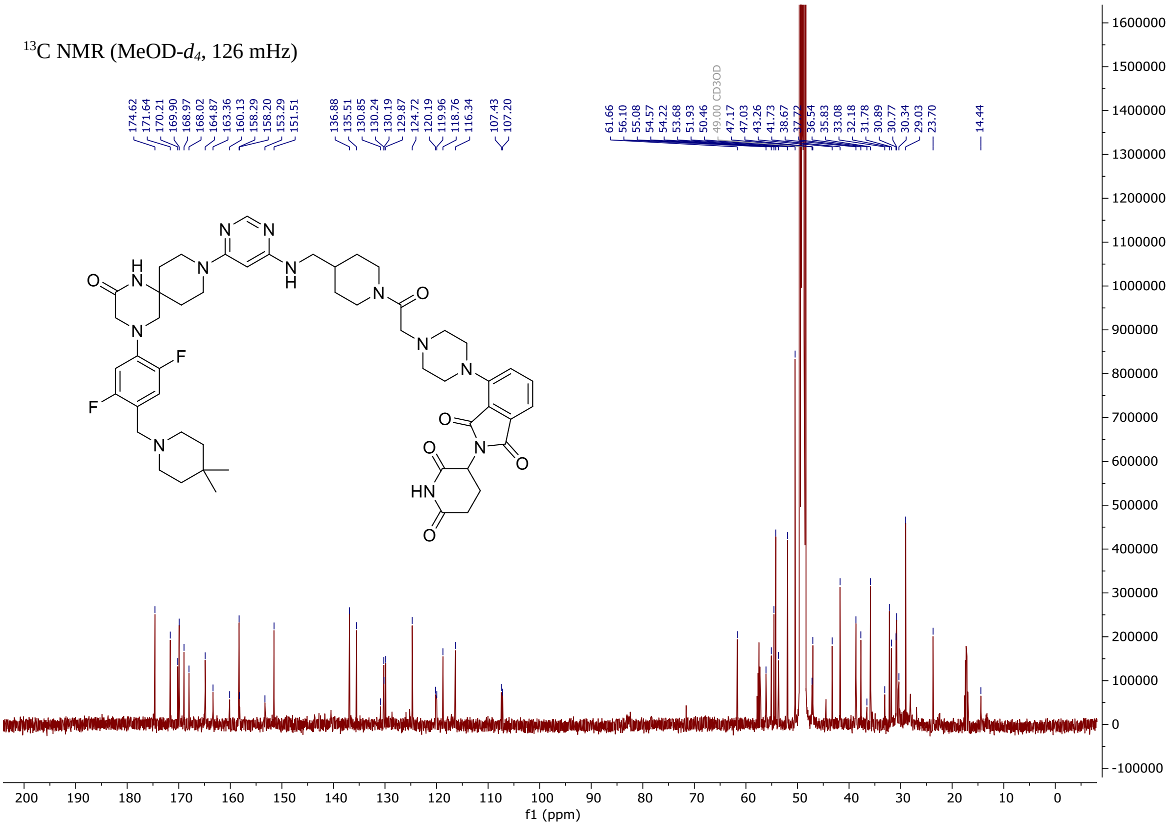

N-(3-(((6-(4-(4-((4,4-dimethylpiperidin-1-yl)methyl)-2,5-difluorophenyl)-2-oxo-1,4,9-triazaspiro[5.5]undecan-9-yl)pyrimidin-4-yl)amino)methyl)benzyl)-4-((2-(1-methyl-2,6-dioxopiperidin-3-yl)-1,3-dioxoisoindolin-4-yl)amino)butanamide (me-14)

^{1}H NMR (MeOD-d_4, 400 mHz)

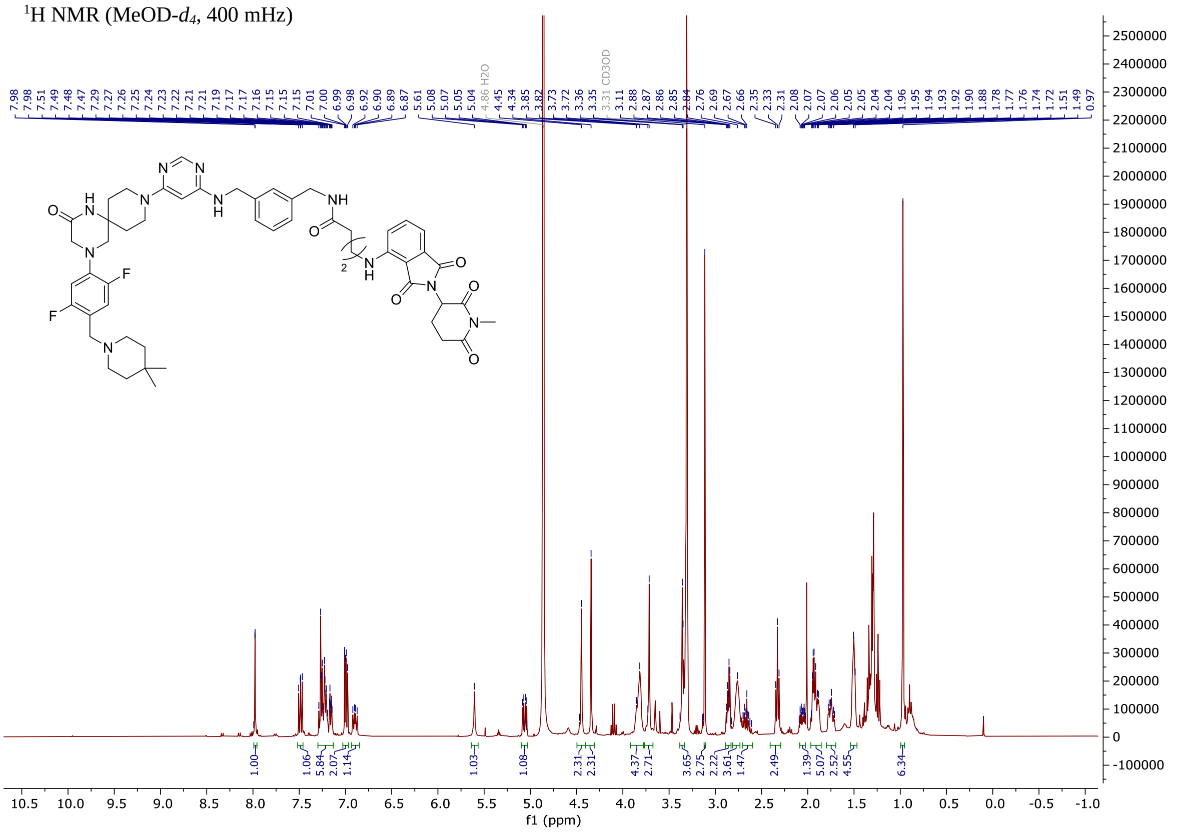

^{13}C NMR (MeOD-d_4, 101 mHz)

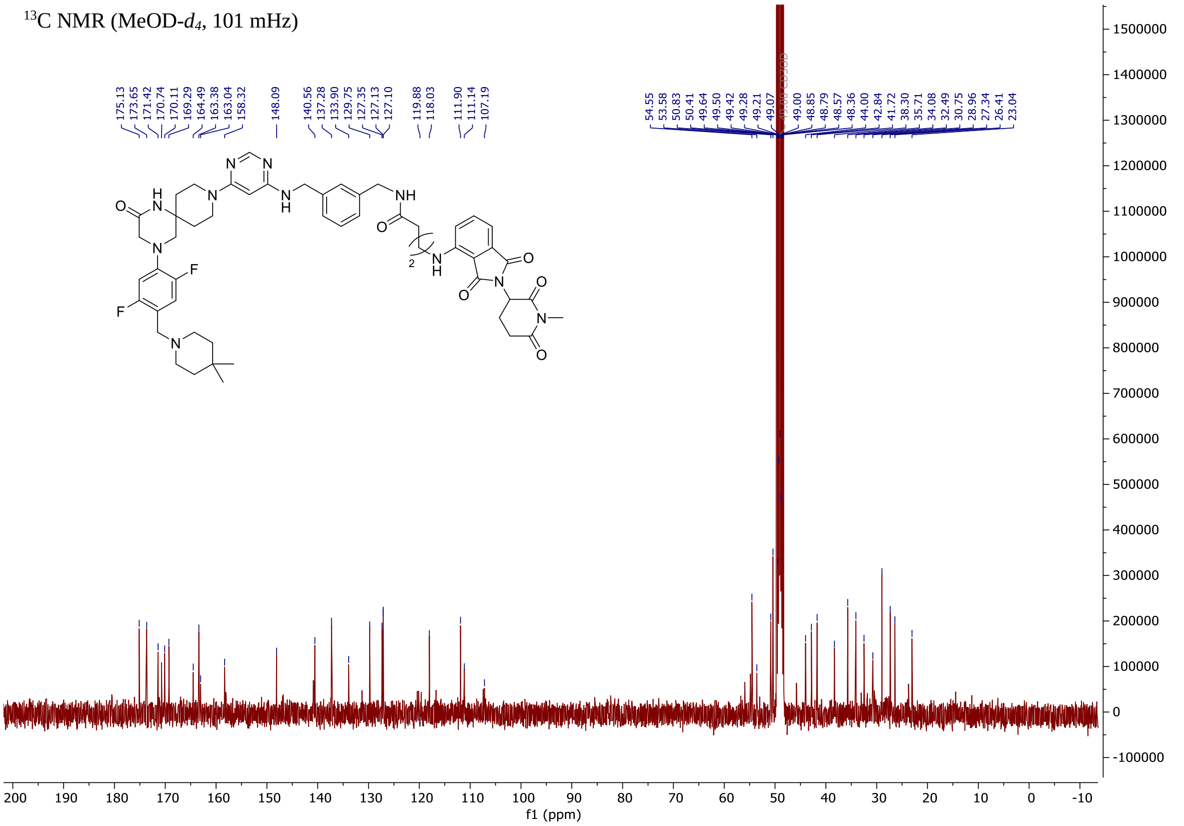

4-((6-(4-(((6-(4-(4-((4,4-dimethylpiperidin-1-yl)methyl)-2,5-difluorophenyl)-2-oxo-1,4,9-triazaspiro[5.5]undecan-9-yl)pyrimidin-4-yl)amino)methyl)piperidin-1-yl)-6-oxohexyl)amino)-2-(1-methyl-2,6-dioxopiperidin-3-yl)isoindoline-1,3-dione (me-24)

^{1}H NMR ($CDCl_3$, 400 mHz)

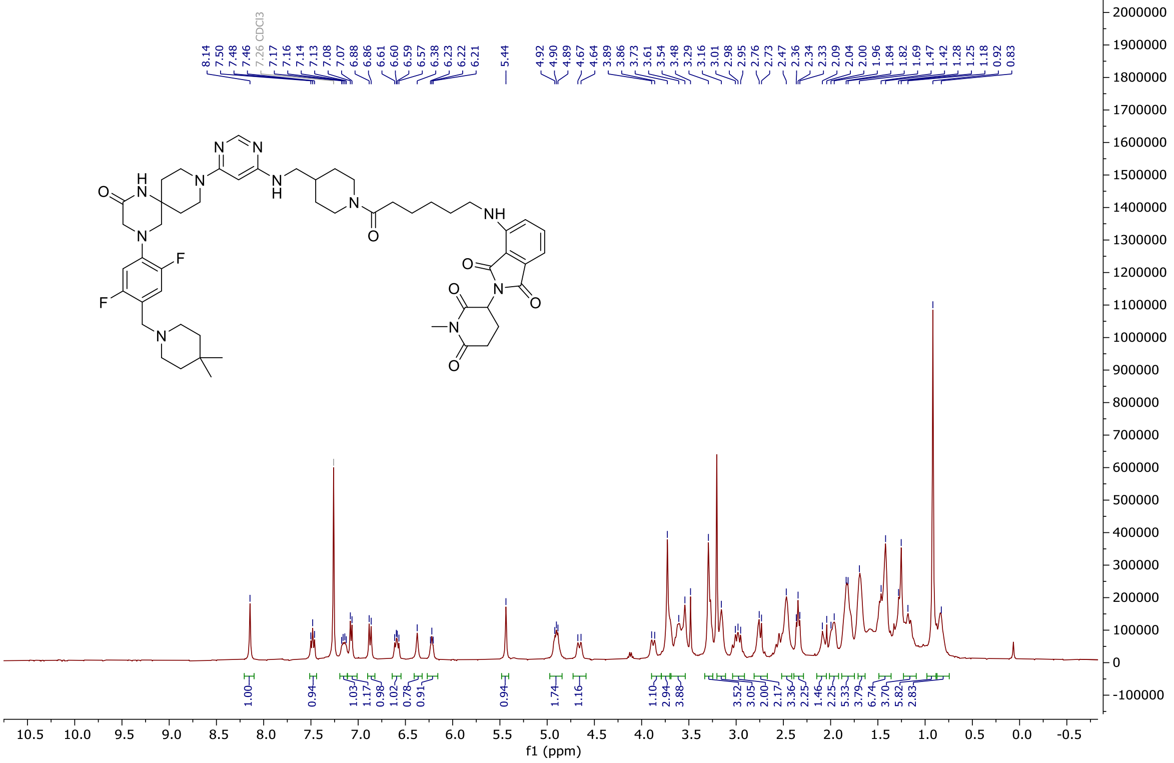

^{13}C NMR ($CDCl_3$, 101 mHz)

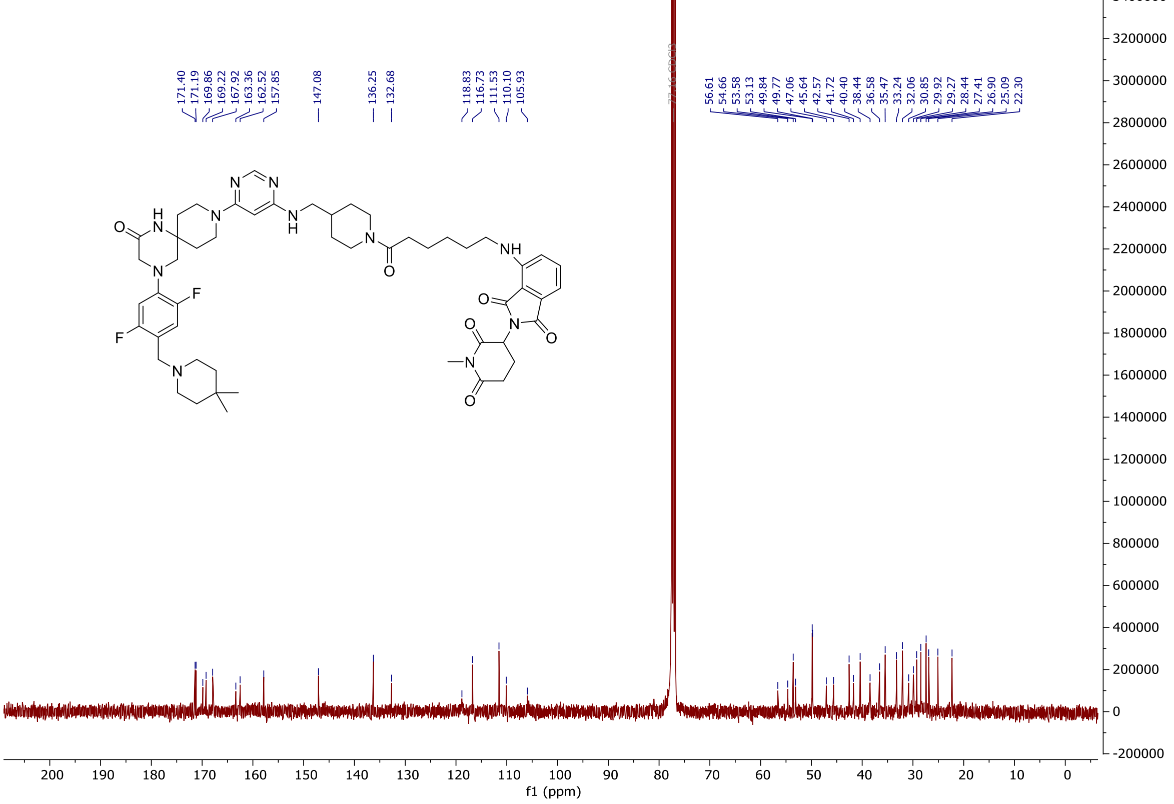

*HPLC traces of target compounds **1-35**, **me-14** and **me-24**.*

N-(3-((6-(4-(4-((4,4-dimethylpiperidin-1-yl)methyl)phenyl)-2-oxo-1,4,9-triazaspiro[5.5]undecan-9-yl)pyrimidin-4-yl)amino)propyl)-3-(2-(2-((2-(2,6-dioxopiperidin-3-yl)-1,3-dioxoisoindolin-4-yl)amino)ethoxy)ethoxy)propenamide (1)

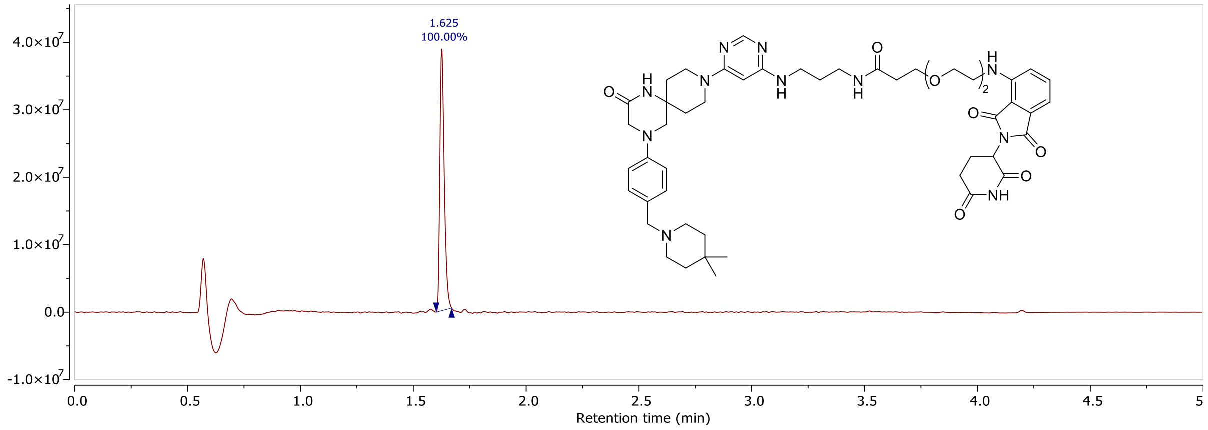

N-(3-((6-(4-(4-((4,4-dimethylpiperidin-1-yl)methyl)phenyl)-2-oxo-1,4,9-triazaspiro[5.5]undecan-9-yl)pyrimidin-4-yl)amino)propyl)-3-(2-(2-(2-((2-(2,6-dioxopiperidin-3-yl)-1,3-dioxoisoindolin-4-yl)amino)ethoxy)ethoxy)ethoxy)propenamide (2)

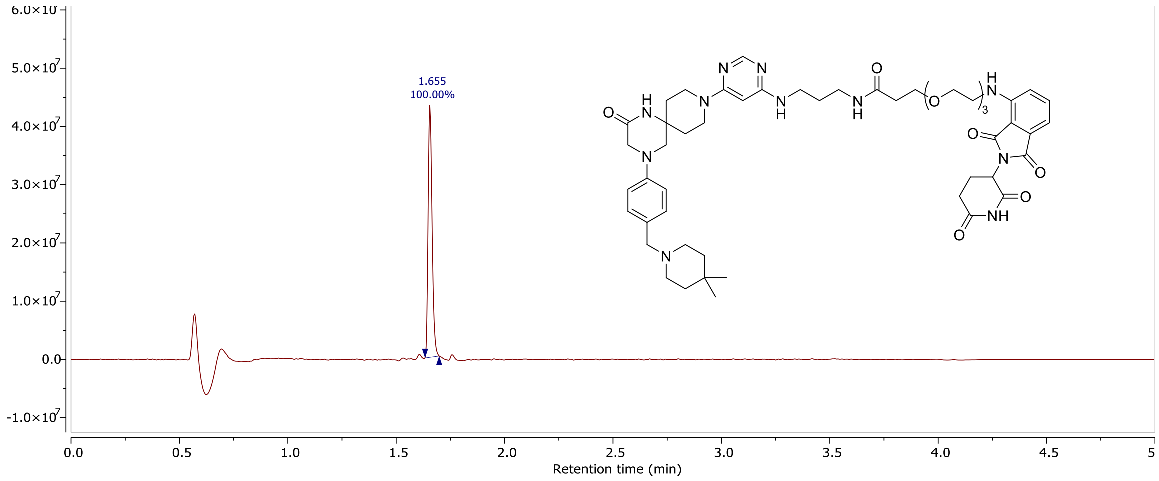

N-(3-((6-(4-(4-((4,4-dimethylpiperidin-1-yl)methyl)phenyl)-2-oxo-1,4,9-triazaspiro[5.5]undecan-9-yl)pyrimidin-4-yl)amino)propyl)-1-((2-(2,6-dioxopiperidin-3-yl)-1,3-dioxoisoindolin-4-yl)amino)-3,6,9,12-tetraoxapentadecan-15-amide (3)

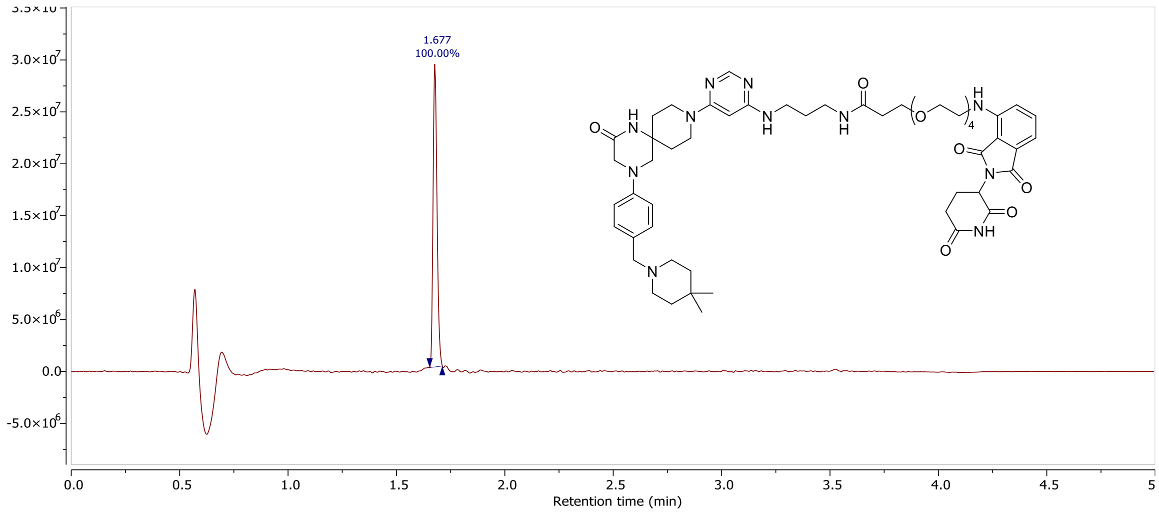

N-(3-((6-(4-(4-((4,4-dimethylpiperidin-1-yl)methyl)phenyl)-2-oxo-1,4,9-triazaspiro[5.5]undecan-9-yl)pyrimidin-4-yl)amino)propyl)-1-((2-(2,6-dioxopiperidin-3-yl)-1,3-dioxoisoindolin-4-yl)amino)-3,6,9,12,15-pentaoxaoctadecan-18-amide (4)

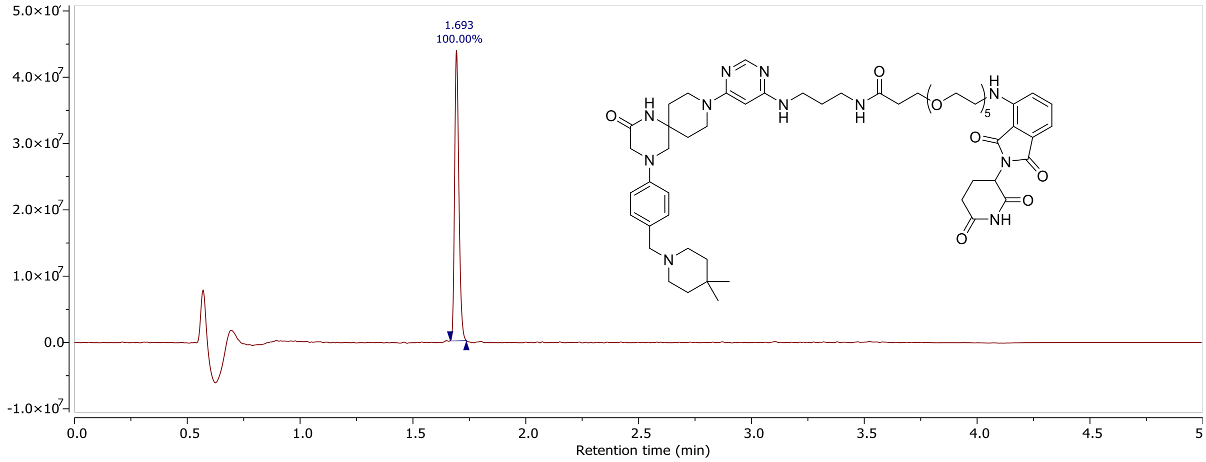

N-(3-((6-(4-(4-((4,4-dimethylpiperidin-1-yl)methyl)phenyl)-2-oxo-1,4,9-triazaspiro[5.5]undecan-9-yl)pyrimidin-4-yl)amino)propyl)-9-((2-(2,6-dioxopiperidin-3-yl)-1-oxoisoindolin-4-yl)amino)nonanamide(5)

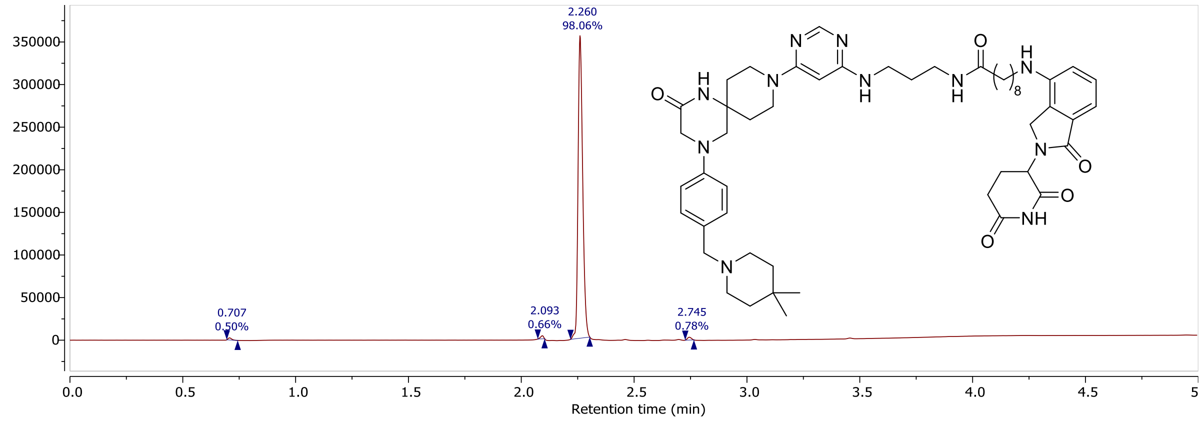

N-(3-((6-(4-(4-((4,4-dimethylpiperidin-1-yl)methyl)phenyl)-2-oxo-1,4,9-triazaspiro[5.5]undecan-9-yl)pyrimidin-4-yl)amino)propyl)-11-((2-(2,6-dioxopiperidin-3-yl)-1-oxoisoindolin-4-yl)amino)undecanamide(6)

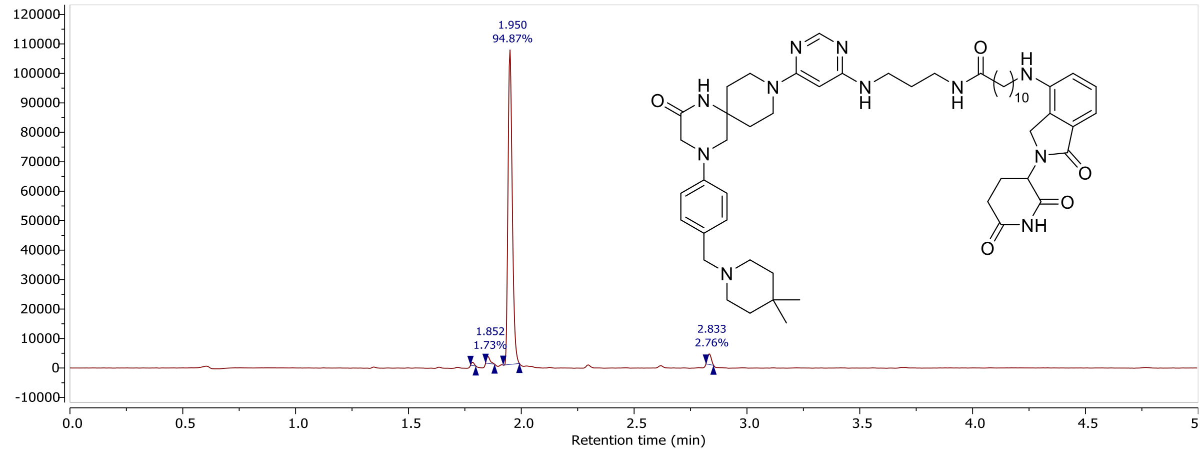

N-(3-((6-(4-(4-((4,4-dimethylpiperidin-1-yl)methyl)phenyl)-2-oxo-1,4,9-triazaspiro[5.5]undecan-9-yl)pyrimidin-4-yl)amino)propyl)-15-((2-(2,6-dioxopiperidin-3-yl)-1-oxoisoindolin-4-yl)amino)pentadecanamide(7)

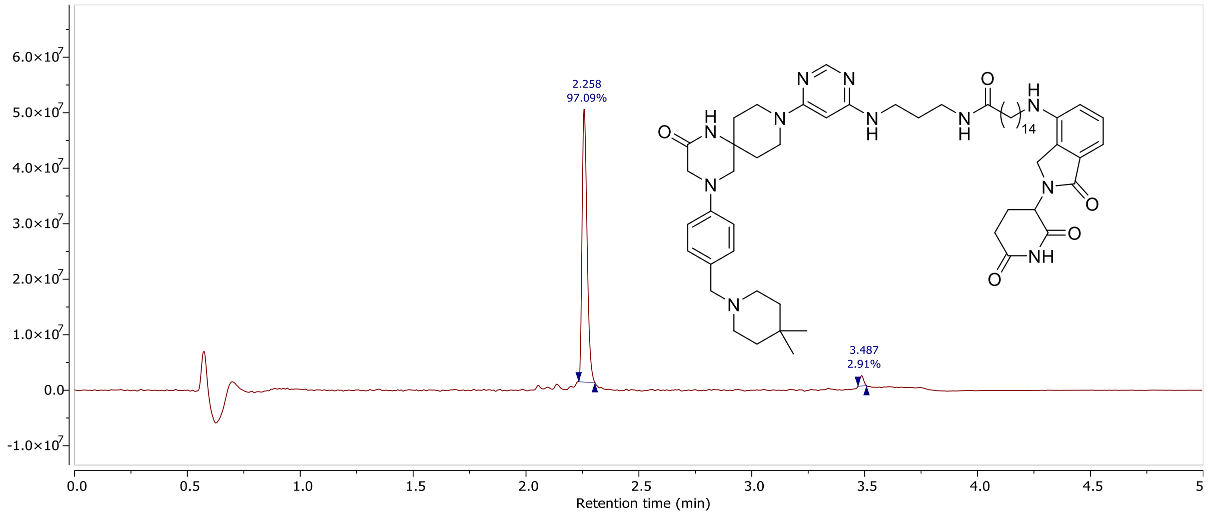

N-(3-((6-(4-(4-((4,4-dimethylpiperidin-1-yl)methyl)phenyl)-2-oxo-1,4,9-triazaspiro[5.5]undecan-9-yl)pyrimidin-4-yl)amino)propyl)-11-(4-(((2-(2,6-dioxopiperidin-3-yl)-1,3-dioxoisoindolin-4-yl)amino)methyl)-1H-1,2,3-triazol-1-yl)undecanamide(8)

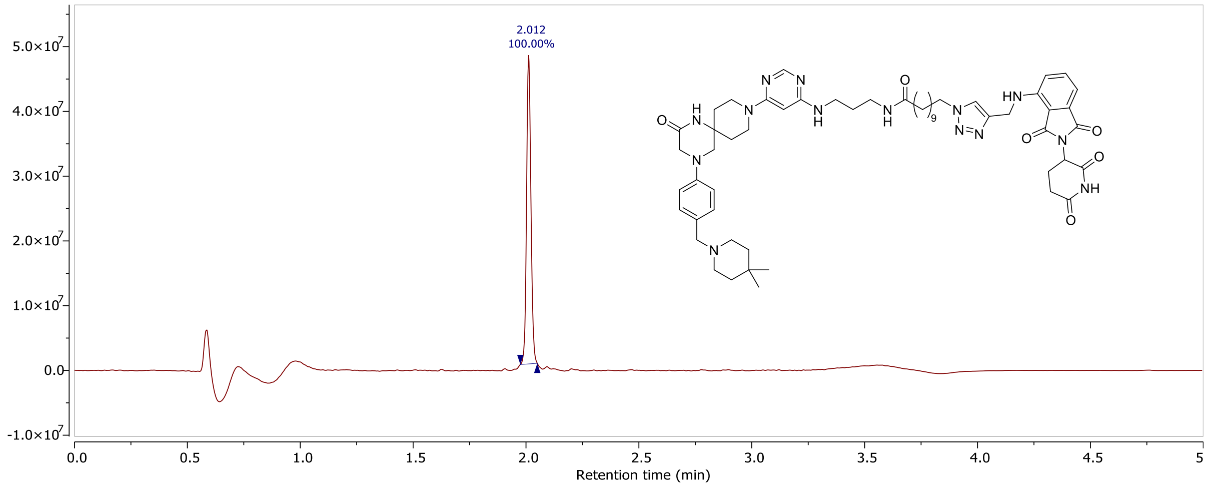

N-(3-((6-(4-(4-((4,4-dimethylpiperidin-1-yl)methyl)phenyl)-2-oxo-1,4,9-triazaspiro[5.5]undecan-9-yl)pyrimidin-4-yl)amino)propyl)-9-(4-(((2-(2,6-dioxopiperidin-3-yl)-1,3-dioxoisoindolin-4-yl)amino)methyl)-1H-1,2,3-triazol-1-yl)nonanamide (9)

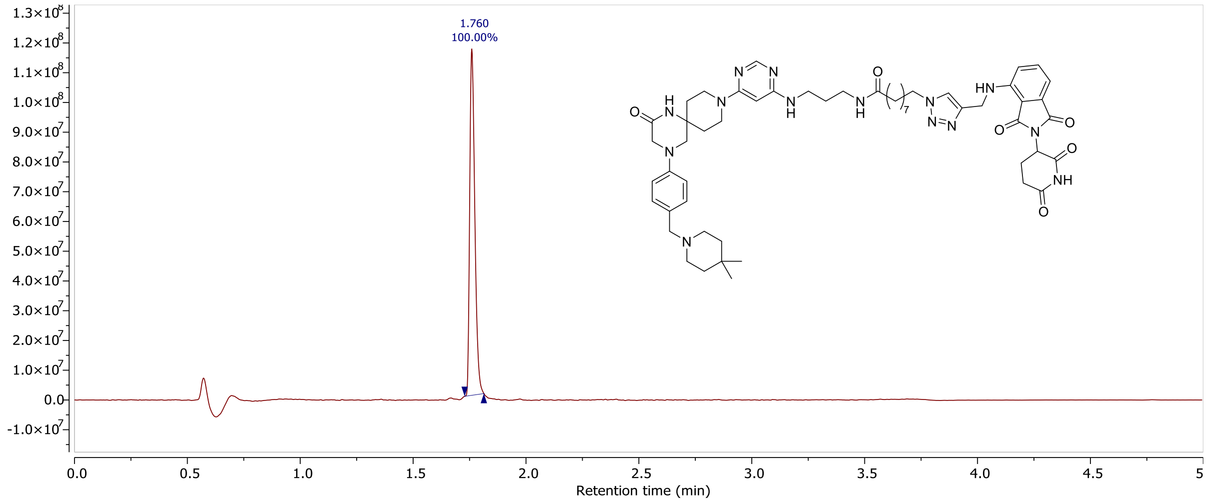

N-(3-((6-(4-(4-((4,4-dimethylpiperidin-1-yl)methyl)-2,5-difluorophenyl)-2-oxo-1,4,9-triazaspiro[5.5]undecan-9-yl)pyrimidin-4-yl)amino)propyl)-9-((2-(2,6-dioxopiperidin-3-yl)-1-oxoisoindolin-4-yl)amino)nonanamide (10)

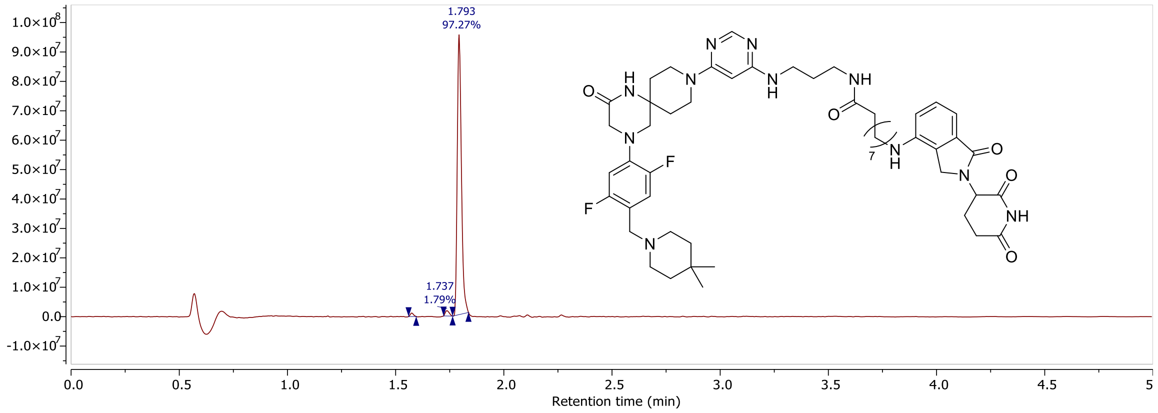

N-(3-((6-(4-(4-((4,4-dimethylpiperidin-1-yl)methyl)-2,5-difluorophenyl)-2-oxo-1,4,9-triazaspiro[5.5]undecan-9-yl)pyrimidin-4-yl)amino)propyl)-11-((2-(2,6-dioxopiperidin-3-yl)-1-oxoisoindolin-4-yl)amino)undecanamide (11)

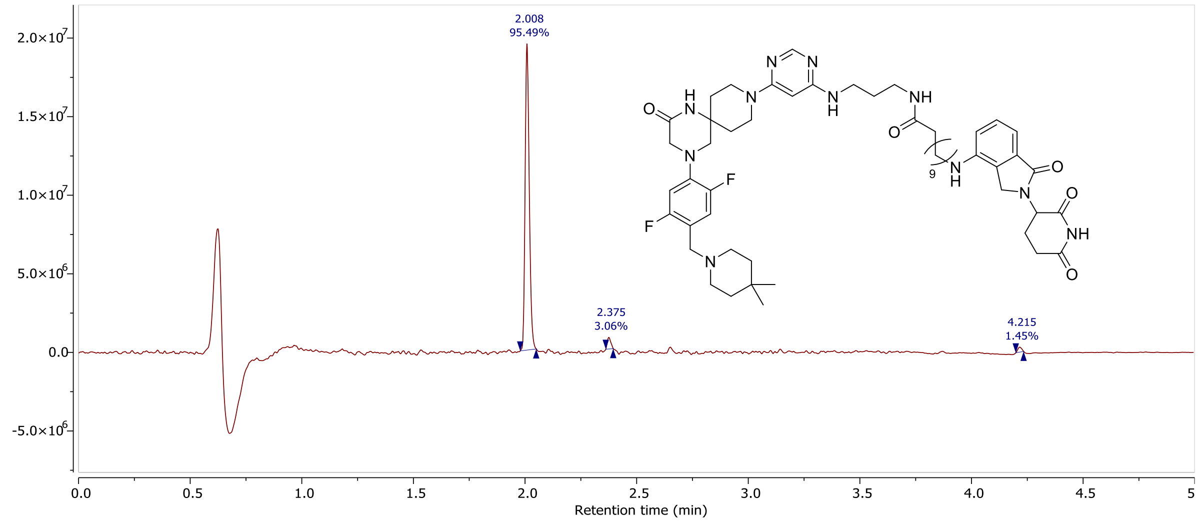

N-(3-((6-(4-(4-((4,4-dimethylpiperidin-1-yl)methyl)-2,5-difluorophenyl)-2-oxo-1,4,9-triazaspiro[5.5]undecan-9-yl)pyrimidin-4-yl)amino)propyl)-9-(4-(((2-(2,6-dioxopiperidin-3-yl)-1,3-dioxoisoindolin-4-yl)amino)methyl)-1H-1,2,3-triazol-1-yl)nonanamide (12)

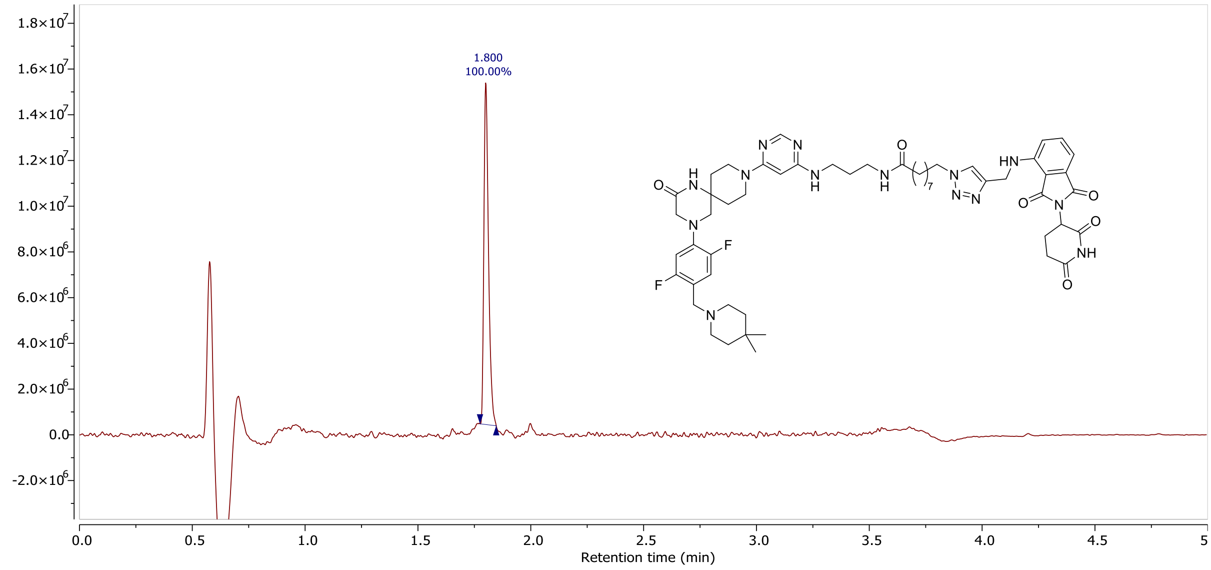

4-((6-(4-(((6-(4-(4-((4,4-dimethylpiperidin-1-yl)methyl)-2,5-difluorophenyl)-2-oxo-1,4,9-triazaspiro[5.5]undecan-9-yl)pyrimidin-4-yl)amino)methyl)piperidin-1-yl)-6-oxohexyl)amino)-2-(2,6-dioxopiperidin-3-yl)isoindoline-1,3-dione (13)

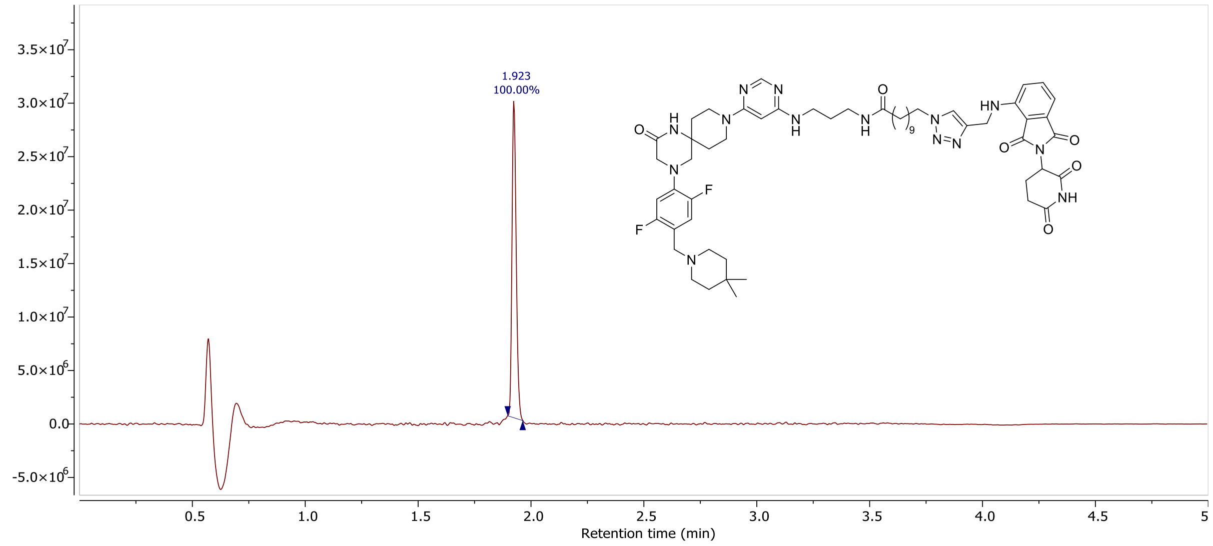

N-(3-(((6-(4-(4-((4,4-dimethylpiperidin-1-yl)methyl)-2,5-difluorophenyl)-2-oxo-1,4,9-triazaspiro[5.5]undecan-9-yl)pyrimidin-4-yl)amino)methyl)benzyl)-4-((2-(2,6-dioxopiperidin-3-yl)-1,3-dioxoisoindolin-4-yl)amino)butanamide (14)

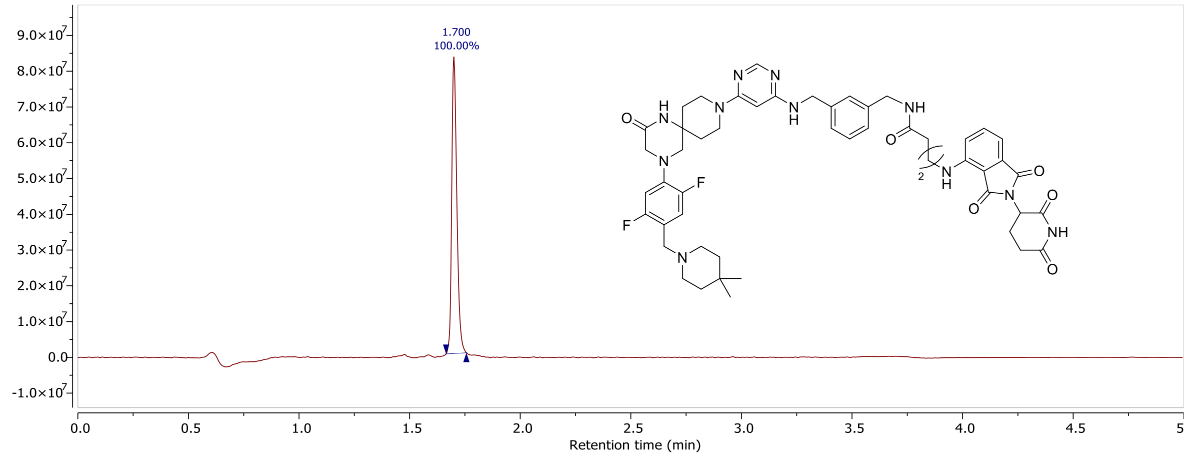

N-(3-(((6-(4-(4-((4,4-dimethylpiperidin-1-yl)methyl)-2,5-difluorophenyl)-2-oxo-1,4,9-triazaspiro[5.5]undecan-9-yl)pyrimidin-4-yl)amino)methyl)benzyl)-6-((2-(2,6-dioxopiperidin-3-yl)-1,3-dioxoisoindolin-4-yl)amino)hexanamide (15)

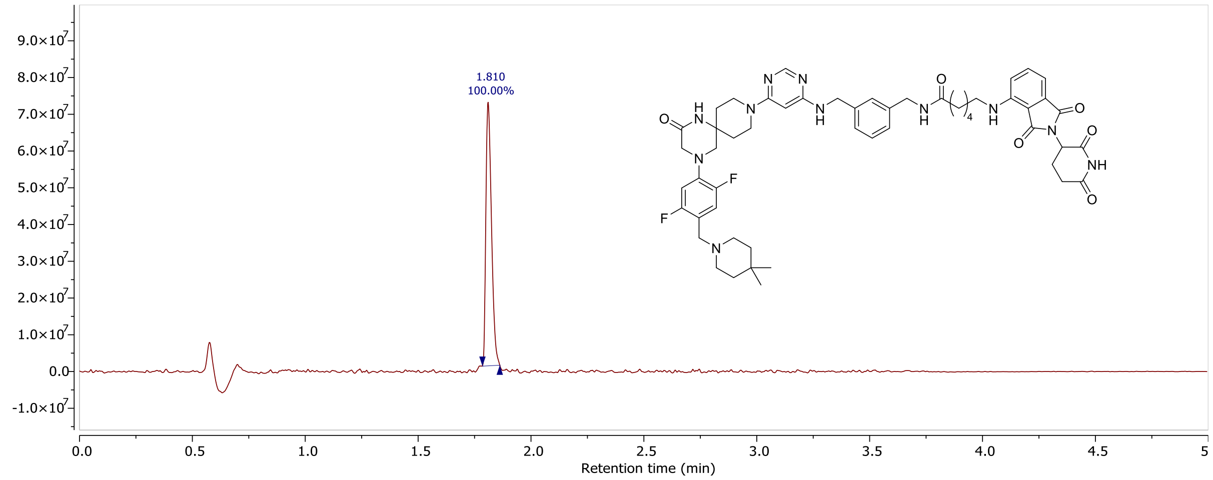

N-(3-(((6-(4-(4-((4,4-dimethylpiperidin-1-yl)methyl)-2,5-difluorophenyl)-2-oxo-1,4,9-triazaspiro[5.5]undecan-9-yl)pyrimidin-4-yl)amino)methyl)benzyl)-9-((2-(2,6-dioxopiperidin-3-yl)-1-oxoisoindolin-4-yl)amino)nonanamide (16)

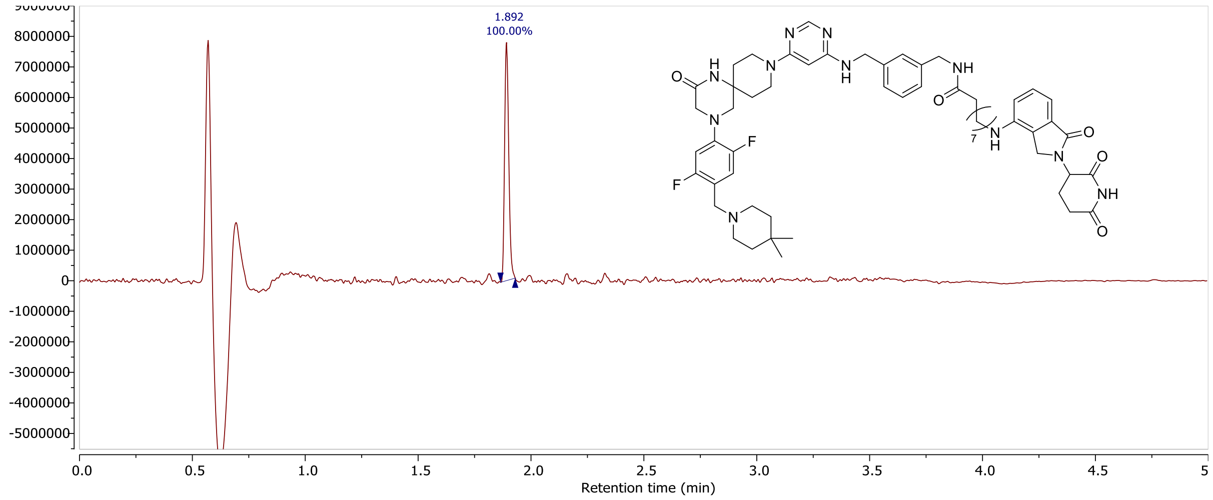

N-(3-(((6-(4-(4-((4,4-dimethylpiperidin-1-yl)methyl)-2,5-difluorophenyl)-2-oxo-1,4,9-triazaspiro[5.5]undecan-9-yl)pyrimidin-4-yl)amino)methyl)benzyl)-11-(4-(((2-(2,6-dioxopiperidin-3-yl)-1,3-dioxoisoindolin-4-yl)amino)methyl)-1H-1,2,3-triazol-1-yl)undecanamide (17)

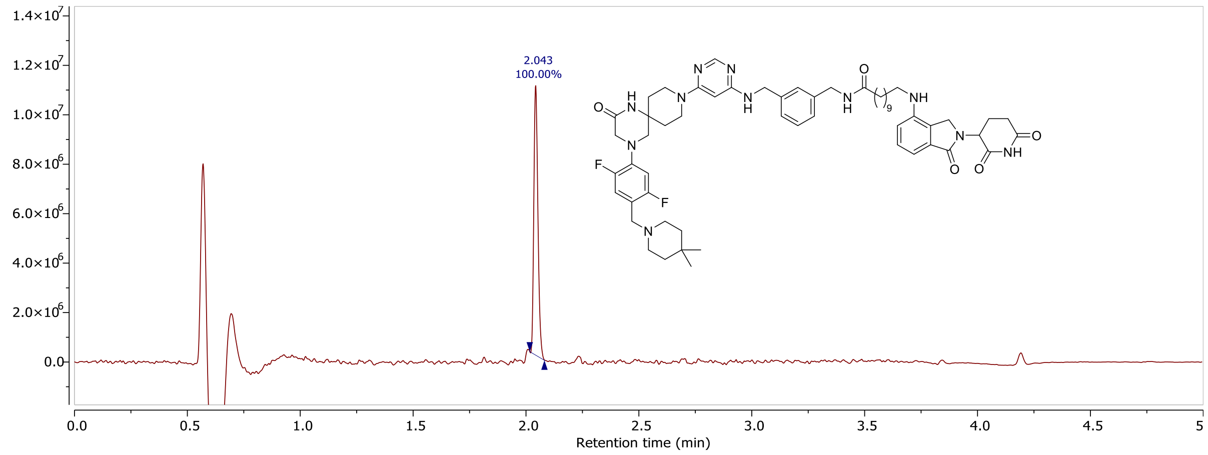

N-(3-(((6-(4-(4-((4,4-dimethylpiperidin-1-yl)methyl)-2,5-difluorophenyl)-2-oxo-1,4,9-triazaspiro[5.5]undecan-9-yl)pyrimidin-4-yl)amino)methyl)benzyl)-3-(4-(((2-(2,6-dioxopiperidin-3-yl)-1,3-dioxoisoindolin-4-yl)amino)methyl)-1H-1,2,3-triazol-1-yl)propenamide (18)

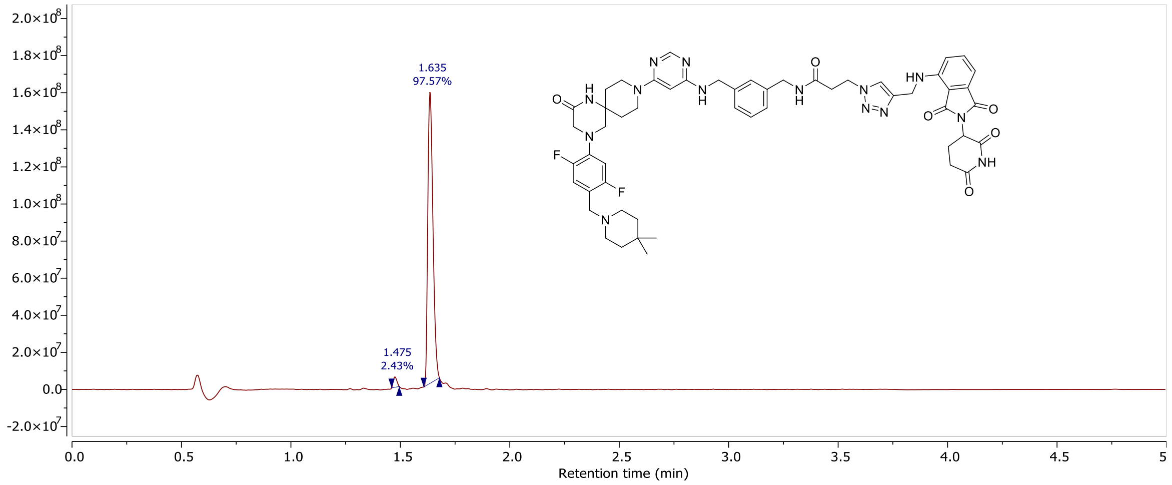

N-(3-(((6-(4-(4-((4,4-dimethylpiperidin-1-yl)methyl)-2,5-difluorophenyl)-2-oxo-1,4,9-triazaspiro[5.5]undecan-9-yl)pyrimidin-4-yl)amino)methyl)benzyl)-9-(4-(((2-(2,6-dioxopiperidin-3-yl)-1,3-dioxoisoindolin-4-yl)amino)methyl)-1H-1,2,3-triazol-1-yl)nonanamide (19)

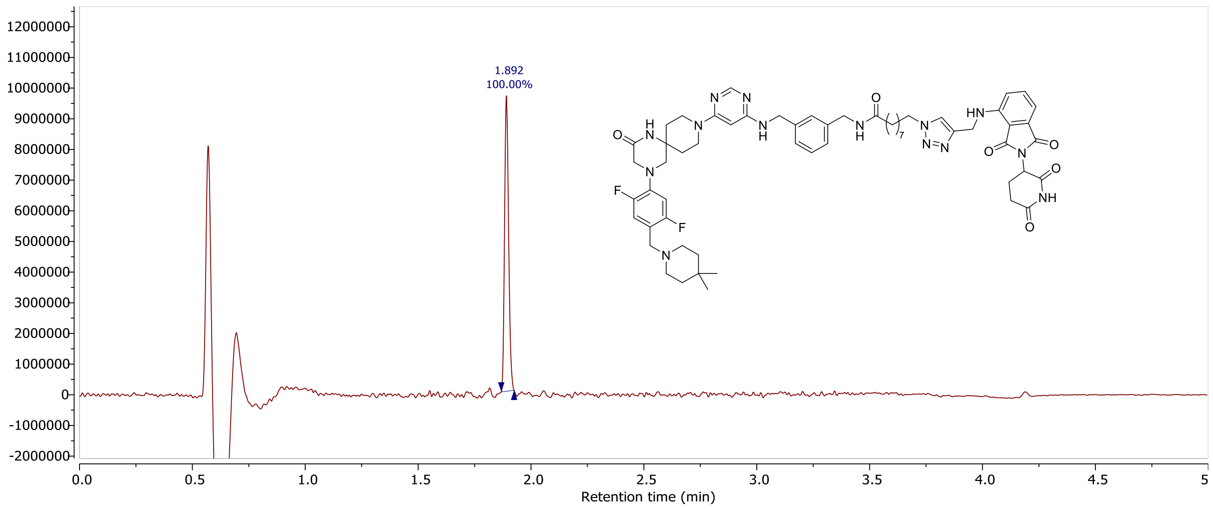

N-(3-(((6-(4-(4-((4,4-dimethylpiperidin-1-yl)methyl)-2,5-difluorophenyl)-2-oxo-1,4,9-triazaspiro[5.5]undecan-9-yl)pyrimidin-4-yl)amino)methyl)benzyl)-11-(4-(((2-(2,6-dioxopiperidin-3-yl)-1,3-dioxoisoindolin-4-yl)amino)methyl)-1H-1,2,3-triazol-1-yl)undecanamide (20)

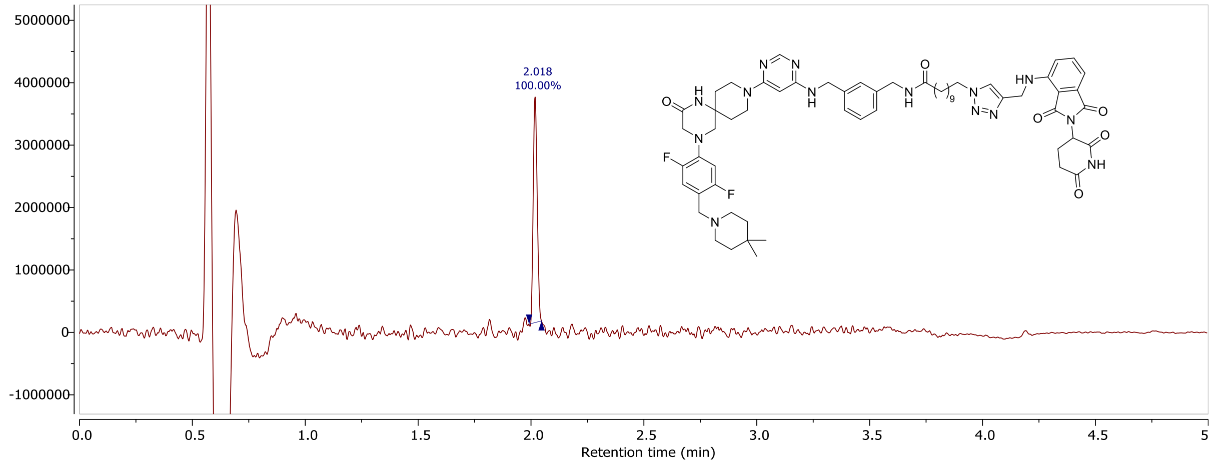

4-((2-(4-(((6-(4-(4-((4,4-dimethylpiperidin-1-yl)methyl)-2,5-difluorophenyl)-2-oxo-1,4,9-triazaspiro[5.5]undecan-9-yl)pyrimidin-4-yl)amino)methyl)piperidin-1-yl)-2-oxoethyl)amino)-2-(2,6-dioxopiperidin-3-yl)isoindoline-1,3-dione (21)

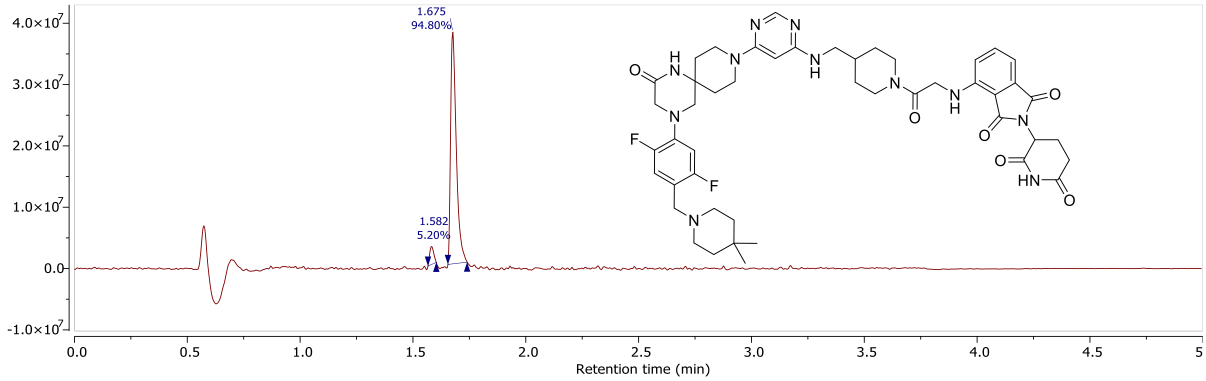

4-((4-(4-(((6-(4-(4-((4,4-dimethylpiperidin-1-yl)methyl)-2,5-difluorophenyl)-2-oxo-1,4,9-triazaspiro[5.5]undecan-9-yl)pyrimidin-4-yl)amino)methyl)piperidin-1-yl)-4-oxobutyl)amino)-2-(2,6-dioxopiperidin-3-yl)isoindoline-1,3-dione (22)

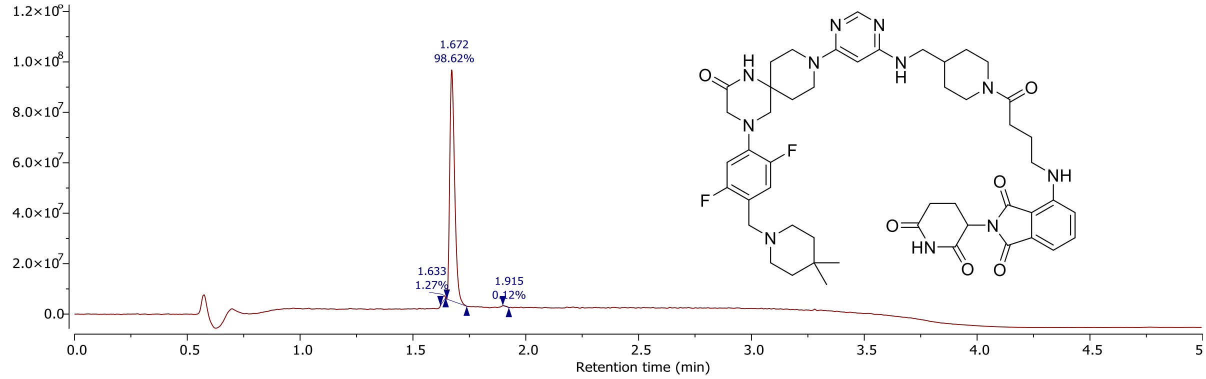

5-((4-(4-(((6-(4-(4-((4,4-dimethylpiperidin-1-yl)methyl)-2,5-difluorophenyl)-2-oxo-1,4,9-triazaspiro[5.5]undecan-9-yl)pyrimidin-4-yl)amino)methyl)piperidin-1-yl)-4-oxobutyl)amino)-2-(2,6-dioxopiperidin-3-yl)isoindoline-1,3-dione (23)

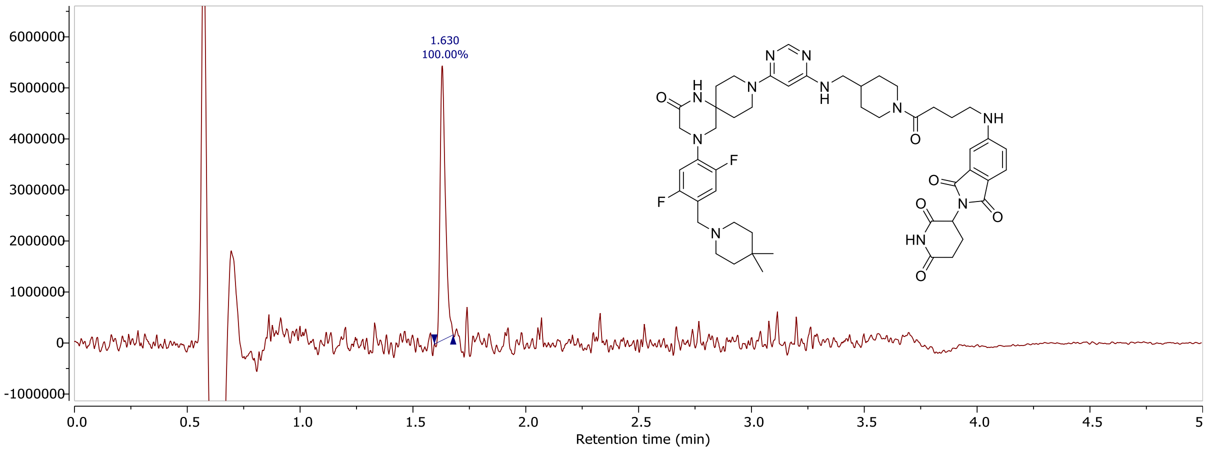

4-((6-(4-(((6-(4-(4-((4,4-dimethylpiperidin-1-yl)methyl)-2,5-difluorophenyl)-2-oxo-1,4,9-triazaspiro[5.5]undecan-9-yl)pyrimidin-4-yl)amino)methyl)piperidin-1-yl)-6-oxohexyl)amino)-2-(2,6-dioxopiperidin-3-yl)isoindoline-1,3-dione (24)

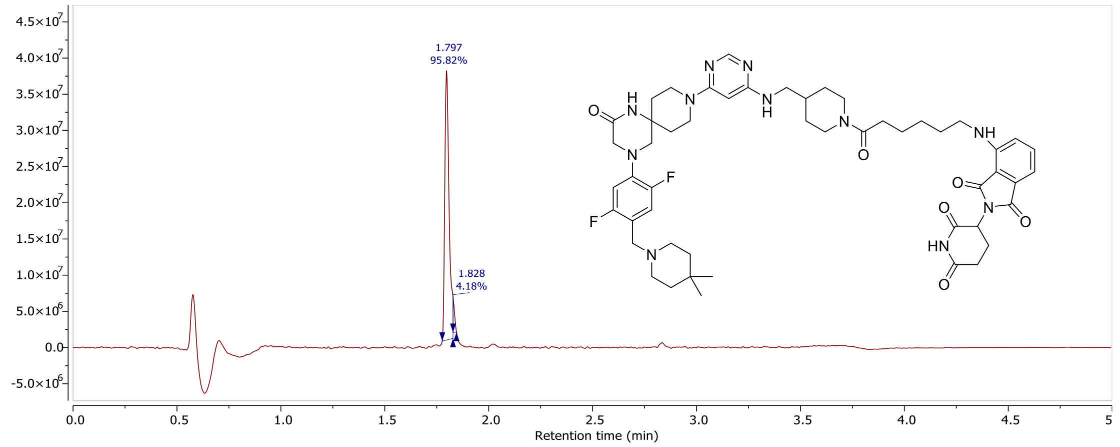

4-((8-(4-(((6-(4-(4-((4,4-dimethylpiperidin-1-yl)methyl)-2,5-difluorophenyl)-2-oxo-1,4,9-triazaspiro[5.5]undecan-9-yl)pyrimidin-4-yl)amino)methyl)piperidin-1-yl)-8-oxooctyl)amino)-2-(2,6-dioxopiperidin-3-yl)isoindoline-1,3-dione (25)

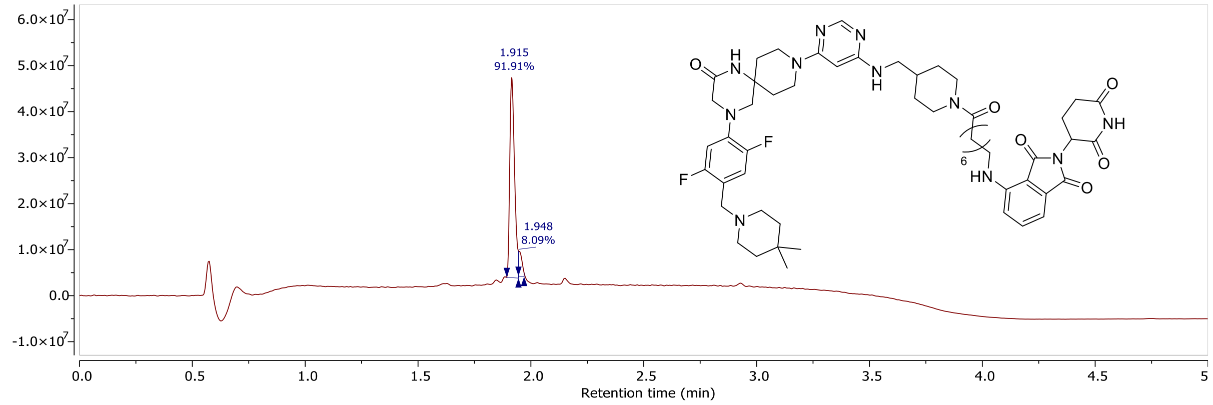

9-(4-(((6-(4-(4-((4,4-dimethylpiperidin-1-yl)methyl)-2,5-difluorophenyl)-2-oxo-1,4,9-triazaspiro[5.5]undecan-9-yl)pyrimidin-4-yl)amino)methyl)-1H-1,2,3-triazol-1-yl)-N-(2-(2,6-dioxopiperidin-3-yl)-1-oxoisoindolin-4-yl)nonanamide (26)

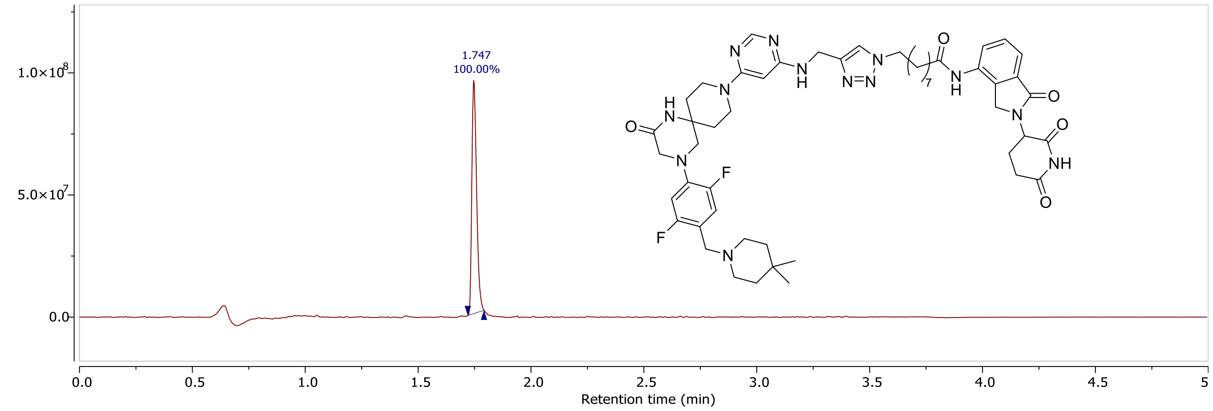

11-(4-(((6-(4-(4-((4,4-dimethylpiperidin-1-yl)methyl)-2,5-difluorophenyl)-2-oxo-1,4,9-triazaspiro[5.5]undecan-9-yl)pyrimidin-4-yl)amino)methyl)-1H-1,2,3-triazol-1-yl)-N-(2-(2,6-dioxopiperidin-3-yl)-1-oxoisoindolin-4-yl)undecanamide (27)

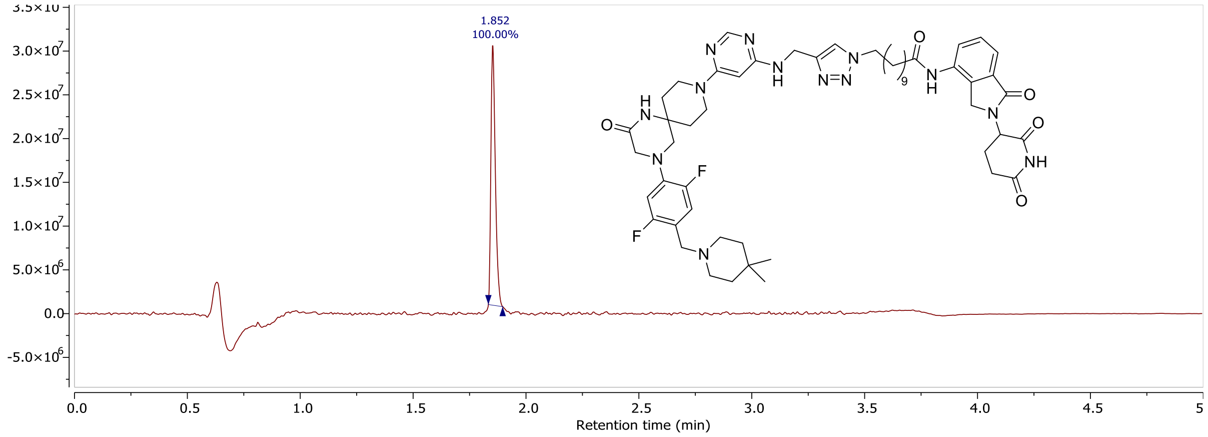

4-(((1-(12-((6-(4-(4-((4,4-dimethylpiperidin-1-yl)methyl)-2,5-difluorophenyl)-2-oxo-1,4,9-triazaspiro[5.5]undecan-9-yl)pyrimidin-4-yl)amino)dodecyl)-1H-1,2,3-triazol-4-yl)methyl)amino)-2-(2,6-dioxopiperidin-3-yl)isoindoline-1,3-dione (28)

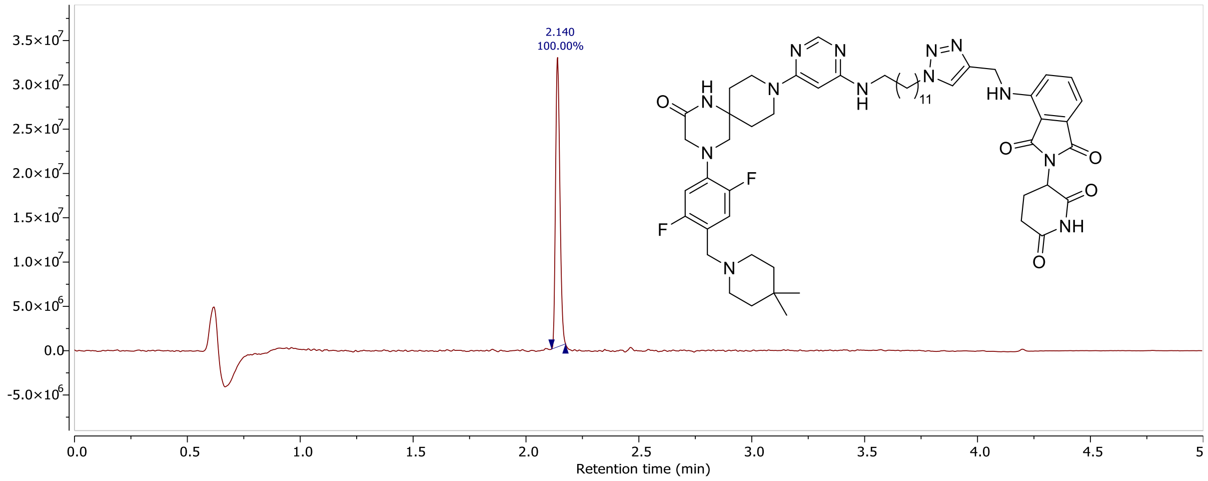

N-(3-(((6-(4-(4-((4,4-dimethylpiperidin-1-yl)methyl)-2,5-difluorophenyl)- 2-oxo-1,4,9-triazaspiro[5.5]undecan-9-yl)pyrimidin-4-yl)4-(2-aminoethyl)piperazin)-4-((2-(2,6-dioxopiperidin-3-yl)-1,3-dioxoisoindolin-4-yl)amino)butanamide (29)

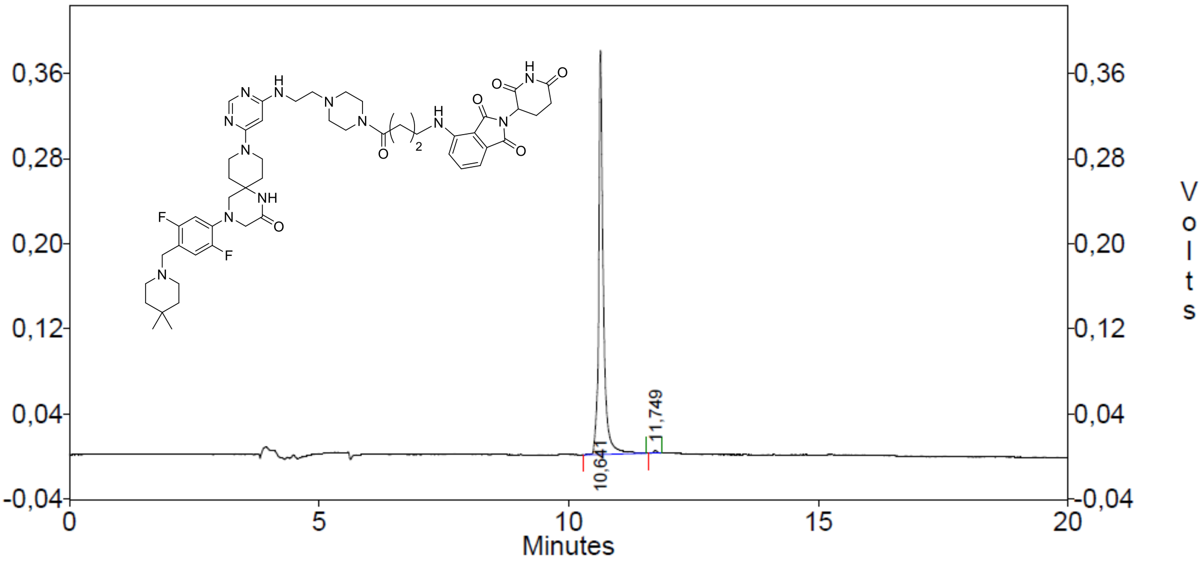

HPLC profile of **29** (95% H_2O 0.1% HCOOH to 95% MeCN in 20 min), *t*R = 10.64 min, 99% purity, detection at 254 nm.

N-(3-(((6-(4-(4-((4,4-dimethylpiperidin-1-yl)methyl)-2,5-difluorophenyl)- 2-oxo-1,4,9-triazaspiro[5.5]undecan-9-yl)pyrimidin-4-yl)4-(2-aminoethyl)piperazin)-6-((2-(2,6-dioxopiperidin-3-yl)-1,3-dioxoisoindolin-4-yl)amino)hexanamide (30)

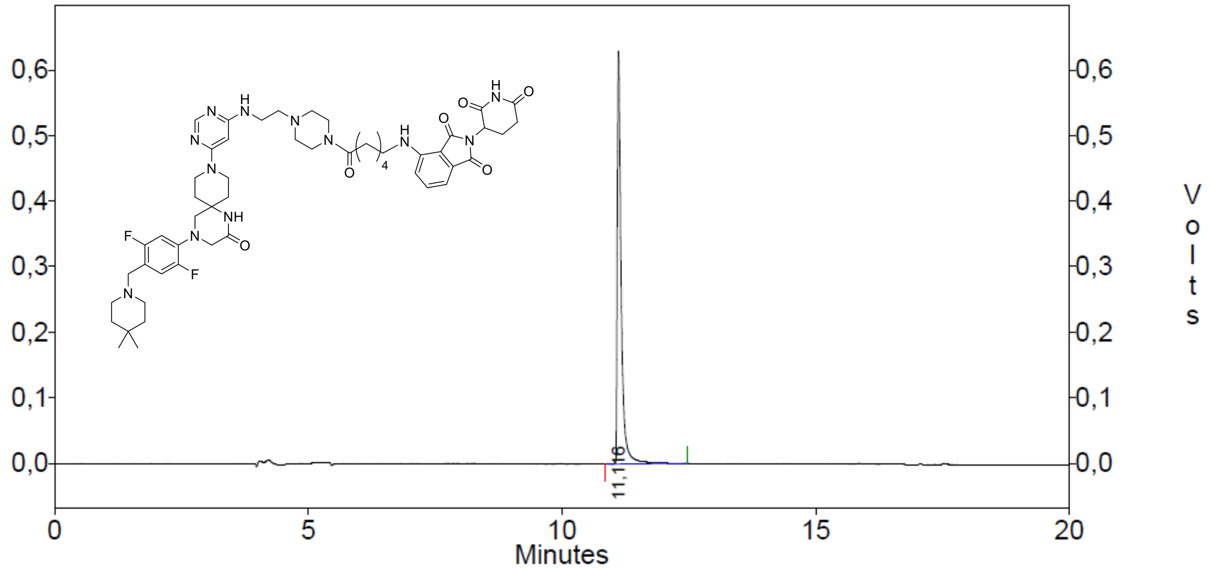

HPLC profile of **30** (95% H_2O 0.1% HCOOH to 95% MeCN in 20 min), *t*R = 11.11 min, 99% purity, detection at 254 nm.

N-(3-(((6-(4-(4-((4,4-dimethylpiperidin-1-yl)methyl)-2,5-difluorophenyl)- 2-oxo-1,4,9-triazaspiro[5.5]undecan-9-yl)pyrimidin-4-yl)4-(2-aminoethyl)piperazin)-8-((2-(2,6-dioxopiperidin-3-yl)-1,3-dioxoisoindolin-4-yl)amino)octanamide (31)

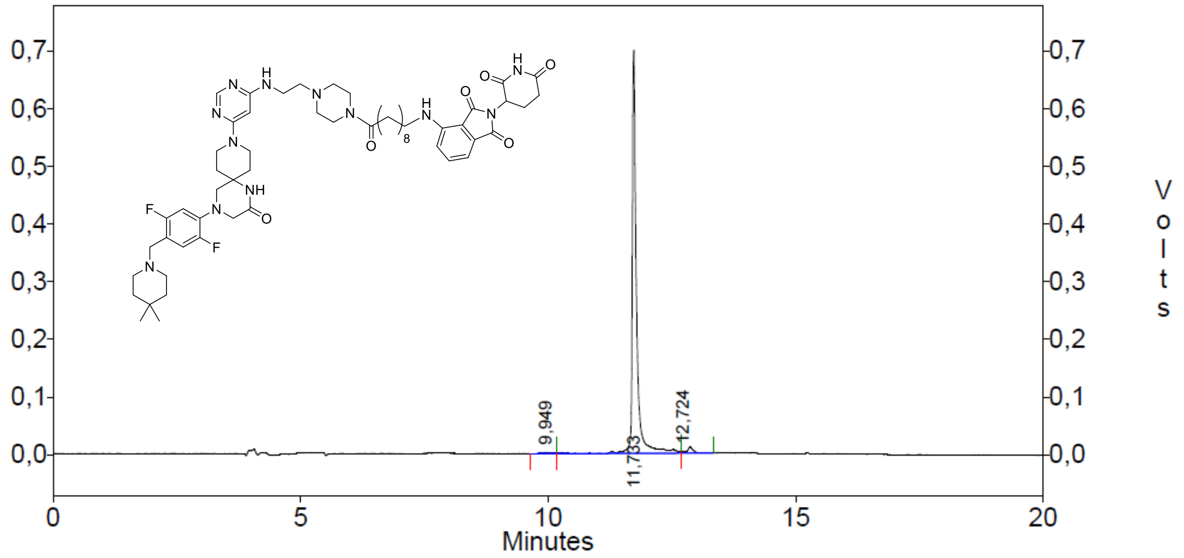

HPLC profile of **31** (95% H_2O 0.1% HCOOH to 95% MeCN in 20 min), *t*R = 11.73 min, 98% purity, detection at 254 nm.

4-(4-(2-((6-(4-(4-((4,4-dimethylpiperidin-1-yl)methyl)-2,5-difluorophenyl)-2-oxo-1,4,9-triazaspiro[5.5]undecan-9-yl)pyrimidin-4-yl)amino)ethyl)piperazin-1-yl)-2-(2,6-dioxopiperidin-3-yl)isoindoline-1,3-dione (32)

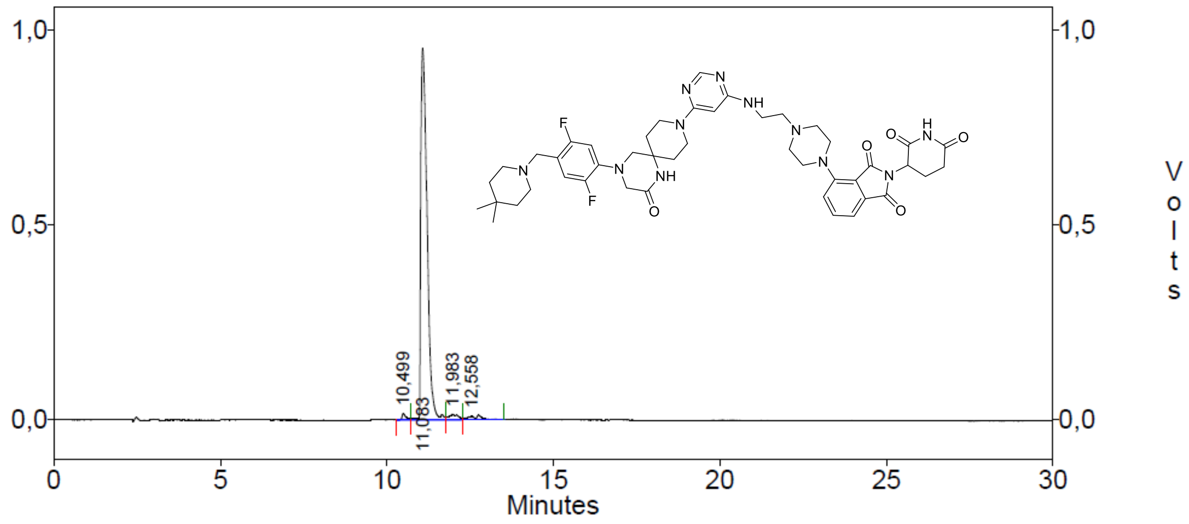

HPLC profile of **32** (95% H_2O 0.1% HCOOH to 95% MeCN in 30 min), *t*R = 11.08 min, 97% purity, detection at 254 nm.

4-((3-(4-(5-(4-(2-((6-(4-(4-((4,4-dimethylpiperidin-1-yl)methyl)-2,5-difluorophenyl)-2-oxo-1,4,9-triazaspiro[5.5]undecan-9-yl)pyrimidin-4-yl)amino)ethyl)piperazin-1-yl)pyrimidin-2-yl)piperazin-1-yl)-3-oxopropyl)amino)-2-(2,6-dioxopiperidin-3-yl)isoindoline-1,3-dione (33)

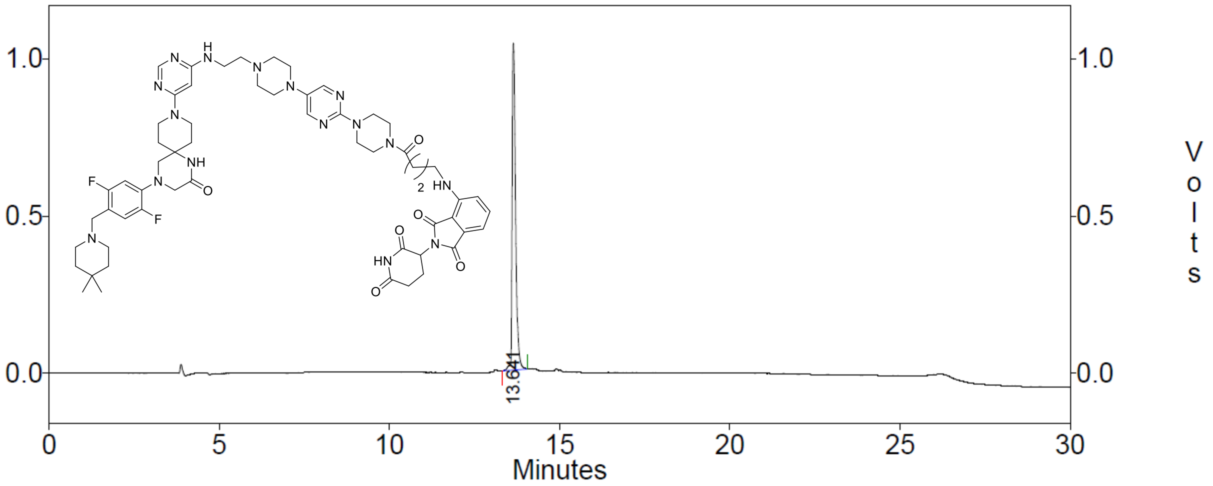

HPLC profile of **33** (95% H_2O 0.1% HCOOH to 95% MeCN in 30 min), *t*R = 13.64 min, 98% purity, detection at 254 nm.

4-(4-(4-(((6-(4-(4-((4,4-dimethylpiperidin-1-yl)methyl)-2,5-difluorophenyl)-2-oxo-1,4,9-triazaspiro[5.5]undecan-9-yl)pyrimidin-4-yl)amino)methyl)phenyl)piperazin-1-yl)-2-(2,6-dioxopiperidin-3-yl)isoindoline-1,3-dione (34)

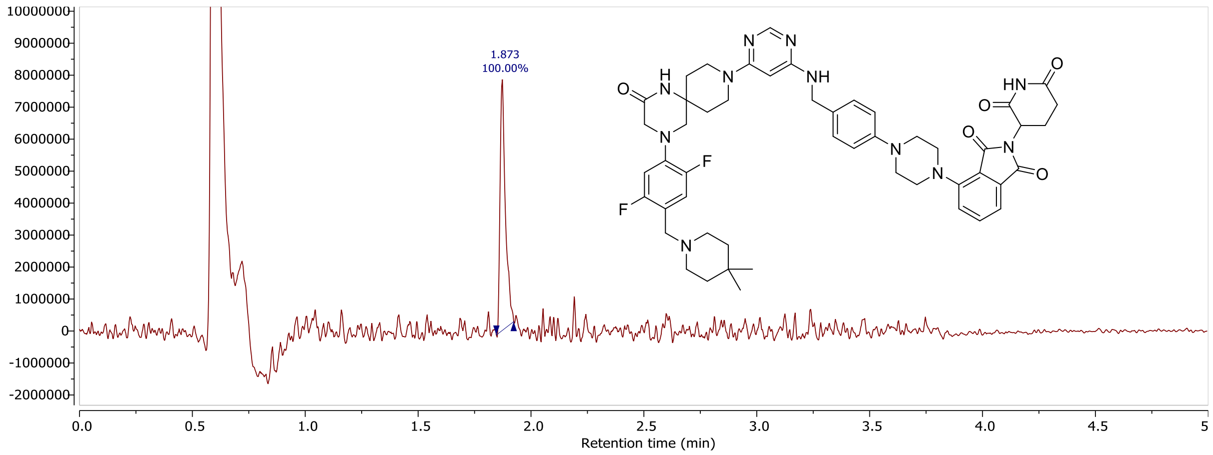

4-(4-(2-(4-(((6-(4-(4-((4,4-dimethylpiperidin-1-yl)methyl)-2,5-difluorophenyl)-2-oxo-1,4,9-triazaspiro[5.5]undecan-9-yl)pyrimidin-4-yl)amino)methyl)piperidin-1-yl)-2-oxoethyl)piperazin-1-yl)-2-(2,6-dioxopiperidin-3-yl)isoindoline-1,3-dione (35)

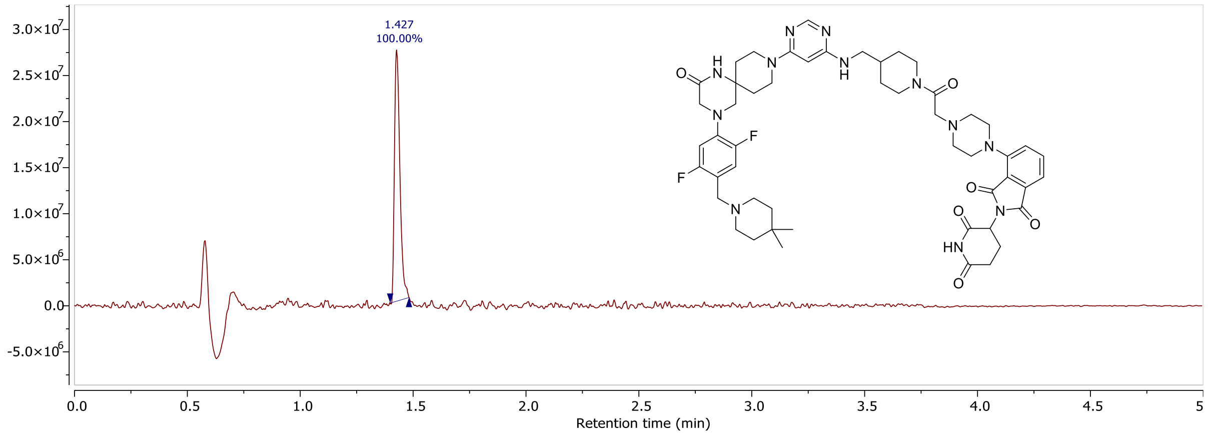

N-(3-(((6-(4-(4-((4,4-dimethylpiperidin-1-yl)methyl)-2,5-difluorophenyl)-2-oxo-1,4,9-triazaspiro[5.5]undecan-9-yl)pyrimidin-4-yl)amino)methyl)benzyl)-4-((2-(1-methyl-2,6-dioxopiperidin-3-yl)-1,3-dioxoisoindolin-4-yl)amino)butanamide (me-14)

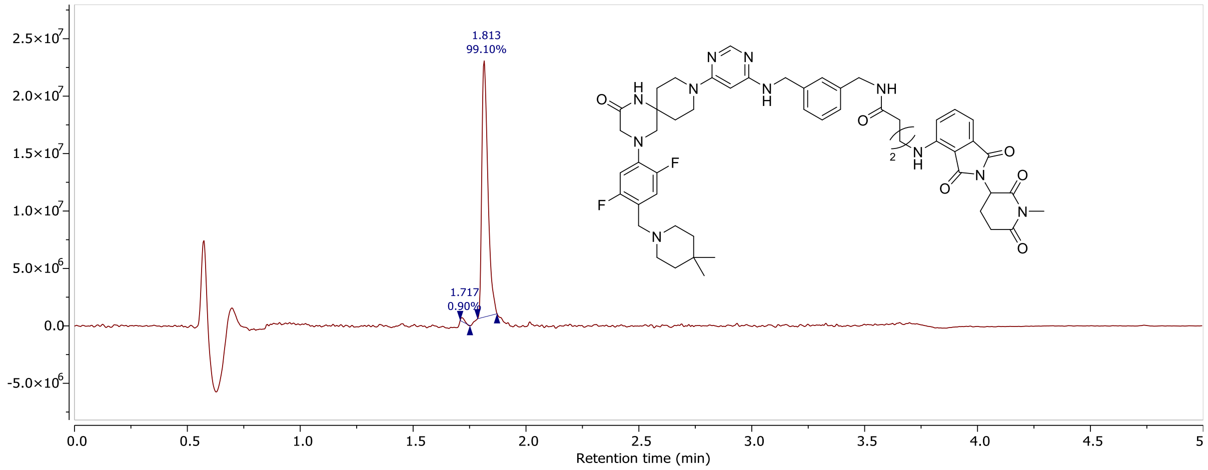

4-((6-(4-(((6-(4-(4-((4,4-dimethylpiperidin-1-yl)methyl)-2,5-difluorophenyl)-2-oxo-1,4,9-triazaspiro[5.5]undecan-9-yl)pyrimidin-4-yl)amino)methyl)piperidin-1-yl)-6-oxohexyl)amino)-2-(1-methyl-2,6-dioxopiperidin-3-yl)isoindoline-1,3-dione (me-24)

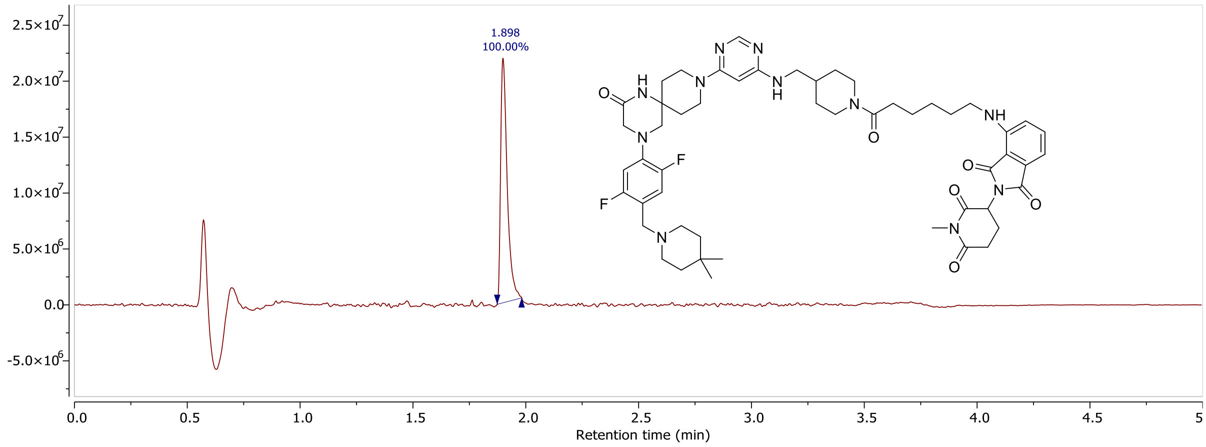

Supplement: Supplementary file 1 — au4c00040_si_001.pdf [file au4c00040_si_001.pdf]
